# Causal interplay between lactose intolerance and gut microbiota: a combined bidirectional Mendelian randomization and *in vivo* validation study

**DOI:** 10.3389/fnut.2026.1803337

**Published:** 2026-06-01

**Authors:** Mengxiong Lu, Zheng Wang, ChunFeng Mei, Meiling She, Hong Xue, Xudong Tang

**Affiliations:** 1 Institute of Digestive Diseases, Peking University Traditional Chinese Medicine Clinical Medical School (Xiyuan), Beijing, China; 2 Institute of Digestive Diseases, Xiyuan Hospital of China Academy of Chinese Medical Sciences, Beijing, China

**Keywords:** genetic variations, gut microbiota, lactose intolerance, Mendelian randomization, microbial pathways

## Abstract

**Objectives:**

The causal relationship between gut microbiota and lactose intolerance (LI) remains elusive due to confounding factors in observational studies. This study aims to decipher the bidirectional causal link between specific gut bacterial taxa and LI by integrating genetic inference with experimental validation.

**Methods:**

We employed a two-sample bidirectional Mendelian Randomization (MR) analysis using summary statistics from the MiBioGen Consortium (gut microbiota) and the FinnGen study (LI). Robustness was assessed via inverse-variance weighted (IVW), MR-Egger, and sensitivity analyses. To validate the genomic findings, we established a lactose-intolerant rat model induced by a high-lactose diet and analyzed cecal microbiota composition using 16S rRNA high-throughput sequencing.

**Results:**

MR analysis identified significant causal associations: the class Deltaproteobacteria, genus Bilophila were identified as potential risk factors for LI, whereas the genus Paraprevotella and Blautia exhibited protective effects. Notably, reverse MR analysis suggested that LI genetically influences host gut microbiota, particularly suppressing carbohydrate metabolism and blooming Bifidobacterium. Experimental sequencing in rats corroborated these findings, showing a distinctive alteration in microbial structure, specifically an increased abundance of Bifidobacterium and a depletion of Blautia in the high-lactose group, consistent with the genetic inference.

**Conclusion:**

This study provides robust evidence for a causal interplay between gut microbiota and LI. The convergence of genetic and experimental data highlights specific taxa, particularly Bifidobacterium and Blautia, as potential biomarkers or therapeutic targets. These findings offer new insights into the microbial etiology of metabolic disorders and suggest microbiota-targeted strategies for LI management.

## 1 Introduction

Lactose intolerance (LI) is a common gastrointestinal condition affecting approximately 68% of the global population, with significant variations observed across different ethnic groups. For instance, it is estimated that around 70% of adults in East Asia and up to 90% in some African regions experience lactose malabsorption, leading to symptoms such as abdominal pain, bloating, and diarrhea upon lactose consumption. The prevalence of LI is particularly high in populations with a historically low consumption of dairy products, indicating a strong evolutionary component to this condition. In the United States, about 25% of adults report symptoms of LI, which significantly impacts their quality of life and dietary choices. Furthermore, the economic burden associated with treating LI-related symptoms and complications underscores the need for effective management strategies. Understanding the underlying mechanisms that contribute to LI, particularly the role of gut microbiota, is crucial for developing targeted interventions and dietary recommendations (1, 2).

Traditional statistical methods, such as observational studies, often face limitations due to confounding factors and reverse causation, which complicate the establishment of causal relationships between dietary habits and health outcomes like LI. These studies may fail to adequately control for variables that influence both exposure and outcomes, leading to biased results. For instance, dietary habits and gut microbiota composition can be influenced by a multitude of factors, including genetics, lifestyle, and environmental conditions, making it difficult to ascertain direct associations. Moreover, the reliance on self-reported data can introduce measurement errors that further cloud the findings. Thus, conventional approaches may not fully elucidate the complex interplay between lactose consumption and the gut microbiome. This highlights the necessity for more robust methodologies to investigate these relationships.

Mendelian randomization (MR) offers a promising alternative to traditional observational methods by using genetic variants as instrumental variables to infer causality. This method capitalizes on the random allocation of genetic variants at conception, which minimizes confounding and reduces the risk of reverse causation. By leveraging genetic data, MR can provide more reliable estimates of the causal effects of exposures on health outcomes. In the context of LI, MR can elucidate how variations in gut microbiota influence lactose metabolism and tolerance. The application of MR in this field is particularly important given the complexities of dietary influences and the need for strong evidence to support interventions aimed at managing LI. MR can help identify potential microbial targets for therapeutic strategies, thereby enhancing the understanding of LI and its management (3, 4).

In this study, we aim to investigate the causal relationship between gut microbiota composition and the risk of developing LI using a two-sample MR approach. We will utilize genetic variants associated with gut microbiota from genome-wide association studies (GWAS) to assess their impact on LI risk. Previous research has indicated that certain gut microbiota, such as Bifidobacterium and Lactobacillus, may play protective roles against LI, while others, like Eubacterium, have been associated with increased risk. This study will also examine how dietary factors, such as lactose intake, and host genetics interact to influence gut microbiota composition and, consequently, LI risk. By employing multiple MR methodologies, including inverse-variance weighted (IVW) and MR-Egger regression, we aim to ensure the robustness and reliability of our findings. Furthermore, sensitivity analyses will be conducted to evaluate the stability of the results, providing insights into the causal pathways that link gut microbiota and LI (5, 6).

This study is expected to yield valuable insights that may guide clinical practices and future research directions. By elucidating the complex relationship between gut microbiota and LI, this research could lead to the development of microbiota-targeted therapies, which may offer new avenues for managing this prevalent condition effectively (7, 8).

## 2 Methods

### 2.1 Study design

**Figure 1:**
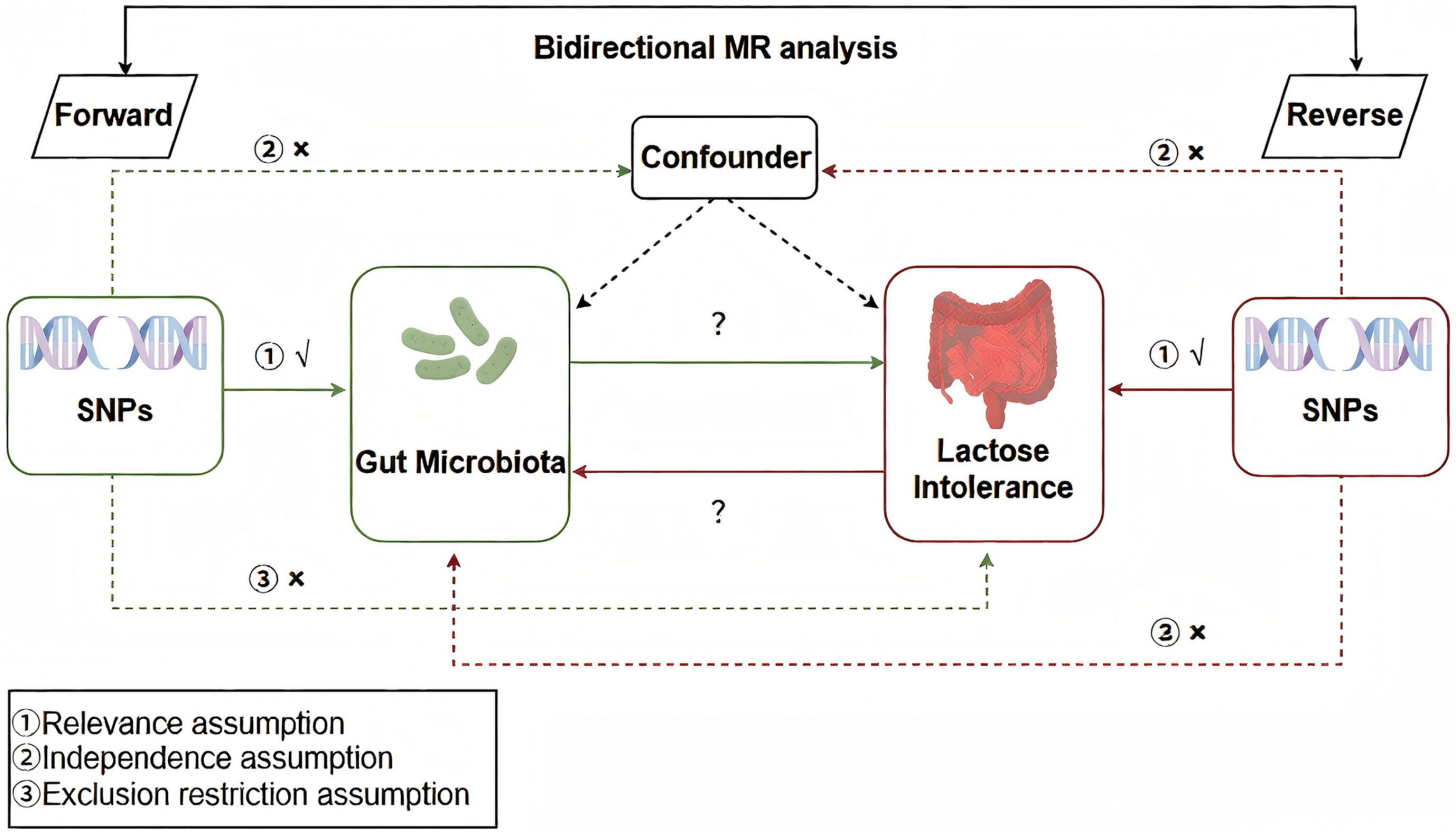
Mendelian randomization (MR) study flowchart (SNPs: Single nucleotide polymorphisms).

This study employed a two-sample bidirectional MR analysis design to systematically assess the potential causal relationship between gut microbiota and LI (as shown in Figure 1). The MR method utilizes genetic variations as instrumental variables, effectively inferring the causal association between exposure (gut microbiota) and outcome (LI), while avoiding common confounding biases in traditional observational studies.

### 2.2 Data sources

The exposure data for this study were sourced from the GWAS of the MiBioGen Consortium, which conducted 16S rRNA sequencing on 7,738 independent individuals primarily from Europe, identifying and quantifying 412 types of gut microbiota abundance, covering five taxonomic levels: phylum, class, order, family, and genus, with advantages such as a wide sample source and clear definition of microbiota characteristics. The outcome data were obtained from the latest R12 version of the FinnGen study, with ID finngen_R12_E4_LACTONAS. This data is publicly available,^1^ and the study included 1,470 cases and 420,066 controls, comprising approximately 20,111,796 single nucleotide polymorphisms (SNPs), as part of a large-scale project based on the Finnish isolated population. In this study, the exposure and outcome data came from different sample sets, achieving a true two-sample MR design.

### 2.3 Instrumental variables

To avoid bias caused by linkage disequilibrium and weak instrumental variables, this study employed a strict screening process for candidate genetic instrumental variables. First, a significance threshold of *p* < 1 × 10^−5^ was set for SNPs associated with gut microbiota and LI. Subsequently, the Clumping strategy was used to control for linkage disequilibrium, with *r*^2^ < 0.001 and a 10,000 kb window, ensuring that each SNP was independent. The F-statistic was calculated by F = (beta/se)^2, and weak instrumental variables with *F* < 10 were excluded to minimize weak instrument bias. The exposure and outcome data were then harmonized for alleles, removing palindromic SNPs and variants with inconsistent effect directions. Before formal inference, MR-PRESSO was used to test for horizontal pleiotropy, identifying and excluding potential outlier SNPs to ensure the reliability of the inference results.

### 2.4 Experimental animals and diets

For the experimental component, 16 male Sprague–Dawley rats (260 ± 20 g body weight, specific pathogen-free status) were acquired from Sipeifu (Beijing) Biotechnology Co., Ltd. The animals were kept under controlled conditions, following a 7-day acclimatization period on standard chow.

The experimental diets containing 40, 45, and 50% lactose, along with the standard AIN-93G formulation, were sourced from Keao Xieli Feed Co., Ltd. (Beijing). These high-lactose diets were created by isocalorically substituting corn starch with pharmaceutical-grade lactose, ensuring that the micronutrient composition remained consistent across all formulations.

### 2.5 Grouping and model establishment

**Figure 2:**
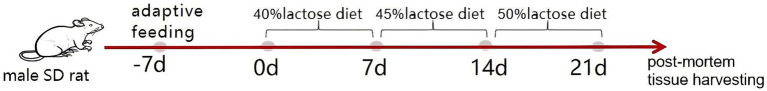
Animal model establishment method.

During the grouping and model establishment phase, 16 rats were randomly divided into two groups: the control group (Group C, *n* = 5), receiving the standard AIN-93G diet, and the model group (Group M, *n* = 5), subjected to a lactose-loading regimen. The model group was provided with a lactose-enriched diet, with a progressive increase of 5% in lactose concentration each week, commencing with a 40% lactose concentration in the first week, followed by 45 and 50% in the subsequent weeks (refer to Figure 2).

### 2.6 Cecal contents collection and microbiota 16S rRNA sequencing

The rats were euthanized using carbon dioxide, and the cecal contents were immediately placed in sterile tubes and stored at −80 °C. High-throughput sequencing of the microbiota 16S rRNA was performed using the Illumina Nextseq2000 platform.

### 2.7 Statistical analysis

In statistical inference, the IVW method was primarily used as the main effect analysis model, which has high statistical power and robust estimation under the premise that all instrumental variables are valid. Additionally, MR-Egger regression, weighted median, weighted mode, and simple mode analyses were employed to test the consistency of results and enhance the credibility of causal inference. Effect sizes were expressed as odds ratios (OR) and their 95% confidence intervals (CI), with a two-tailed test and a statistical significance threshold set at *p* < 0.05. Statistical work was completed using R software (version 4.4.1), primarily utilizing the TwoSampleMR and MR-PRESSO analysis packages.

All high-throughput sequencing data analyses were conducted on the Majorbio Cloud Platform,^2^ specifically as follows: the Wilcoxon test in R software was used to assess the significance of differences between the two groups based on the mean of differential values. Further combined with Linear Discriminant Analysis Effect Size (LEfSe) analysis, species with significant impacts on inter-group differences were screened. At the functional level, 16S functional prediction analysis was performed using PICRUSt2 (version 2.2.0).

## 3 Results

### 3.1 Instrumental variable selection

To conduct MR analysis, this study based on the Dutch Microbiome Project data, selected 207 microbial taxa and 205 microbial-related pathways as exposure factors, extracting SNPs significantly associated with them (*p* < 1 × 10^−5^) as instrumental variables, ultimately obtaining 4,146 SNPs for MR analysis, while also collecting key information such as effect alleles, βvalues, standard errors (SE), *p* values, and sample sizes to ensure the accuracy and reliability of the analysis. The selection of reverse MR instrumental variables followed the above standards, obtaining 9 SNPs from the summary statistics of LI in the FinnGen database for subsequent MR analysis (as shown in Supplementary material).

### 3.2 MR analysis results of gut microbiota on LI risk

**Figure 3:**
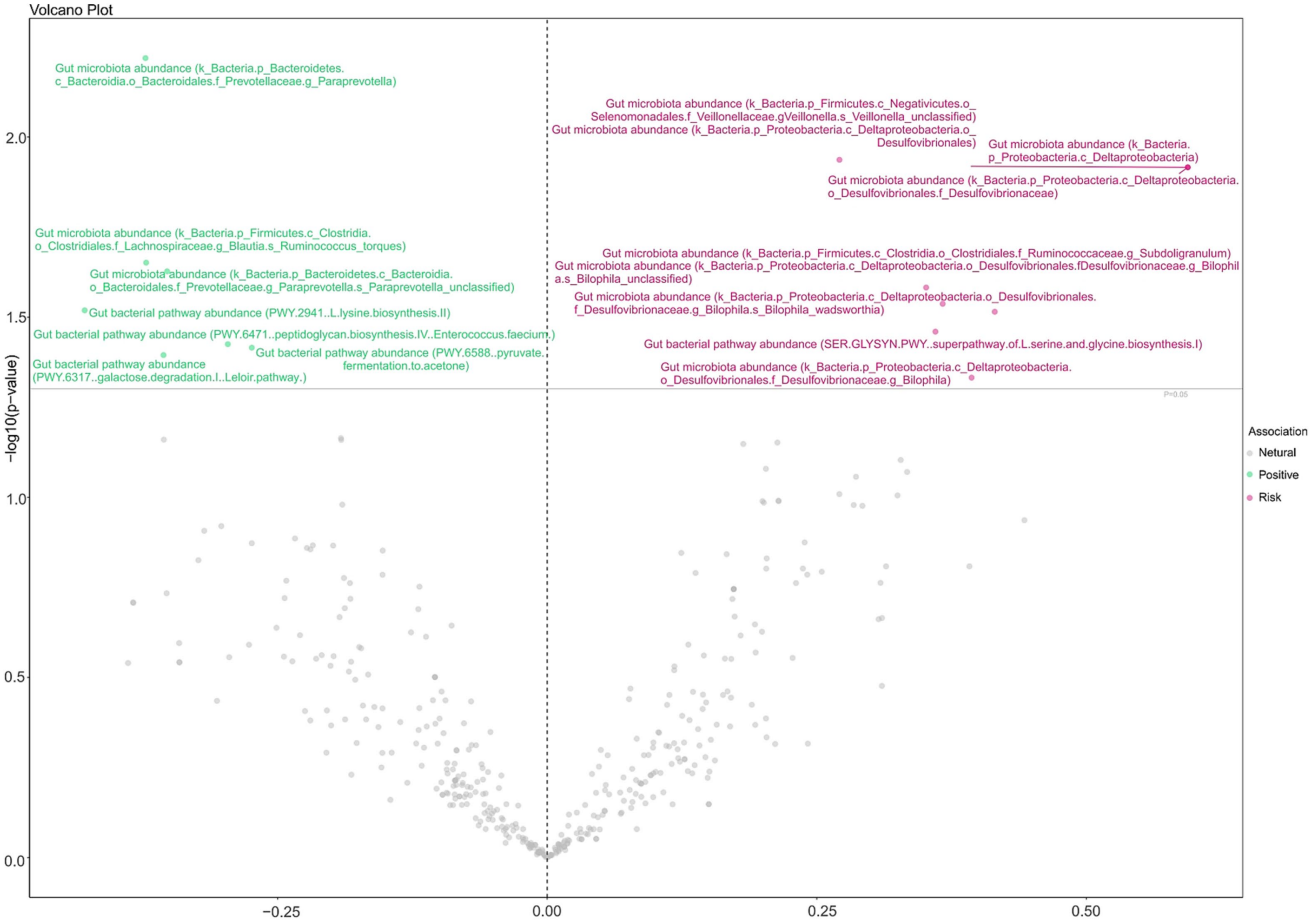
Mendelian randomization results volcano plot with threshold of *p* < 0.05.

**Figure 4:**
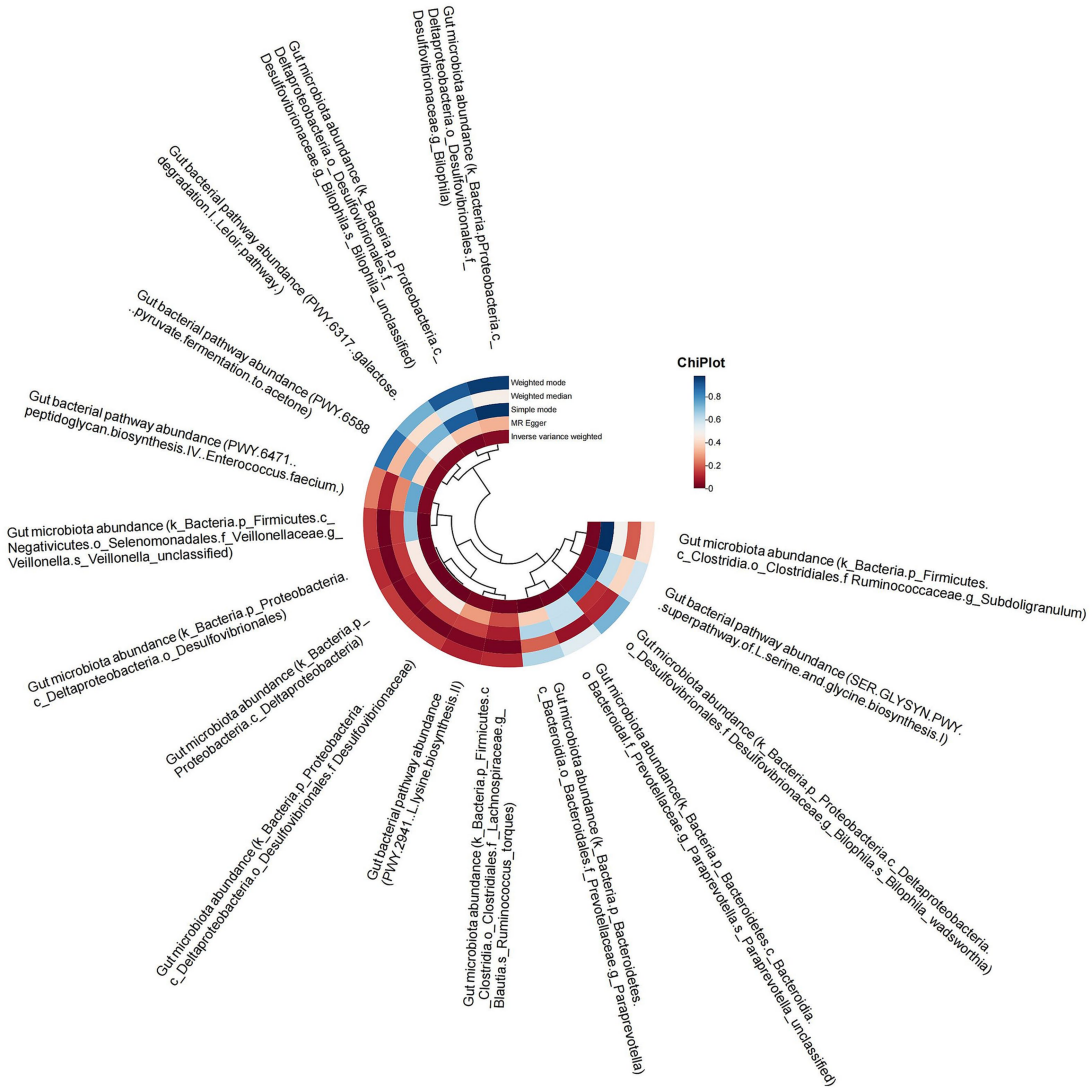
Mendelian randomization positive results heatmap with threshold of *p* < 0.05.

Multilevel regression analysis identified causal relationships between five levels of microbial taxa and LI. A total of 12 gut microbial taxa and functional pathways with causal associations with LI were identified (as shown in Figures 3, 4). In the IVW analysis, Deltaproteobacteria class (OR: 1.811, 95% CI 1.139–2.882, *p* = 0.012), Desulfovibrionales order (OR: 1.811, 95% CI 1.139–2.882, *p* = 0.012), Desulfovibrionaceae family (OR: 1.811, 95% CI 1.139–2.882, *p* = 0.012), Bilophila genus (OR: 1.482, 95% CI 1.006–2.184, *p* = 0.047), *Bilophila wadsworthia* species (OR: 1.515, 95% CI 1.040–2.206, *p* = 0.031), Bilophila_unclassified species (OR: 1.443, 95% CI 1.038–2.006, *p* = 0.029), and Veillonella_unclassified species (OR: 1.311, 95% CI 1.063–1.618, *p* = 0.012) were identified as risk factors for LI. Notably, the analysis results for the Paraprevotella genus showed a protective effect (OR: 0.689, 95% CI 0.528–0.899, *p* = 0.006), while the Paraprevotella_unclassified species under this genus exhibited a risk effect (OR:1.421, 95% CI 1.043–1.937, *p* = 0.026), suggesting heterogeneity in the effects of different species within this genus on the disease. On the other hand, several taxa were identified as protective factors, including Subdoligranulum genus (OR: 0.703, 95% CI 0.518–0.954, *p* = 0.024) and Ruminococcus_torques species under the Blautia genus (OR: 0.689, 95% CI 0.501–0.948, *p* = 0.022). At the level of microbial functional pathways, PWY-6588 (acetone fermentation pathway, OR: 0.760, 95% CI 0.587–0.986, *p* = 0.038), PWY-2941 (L-lysine biosynthesis pathway II, OR: 0.651, 95% CI 0.442–0.960, *p* = 0.030), PWY-6317 (galactose degradation pathway I, OR: 0.701, 95% CI 0.499–0.984, *p* = 0.040), and PWY-6471 (peptidoglycan biosynthesis pathway IV, OR: 0.744, 95% CI 0.562–0.983, *p* = 0.038) all exhibited protective effects. However, the analysis results for SER. GLYSYN. PWY (L-serine and glycine biosynthesis superpathway I) suggested it may be a risk factor (OR:1.433, 95% CI 1.026–2.002, *p* = 0.035).

### 3.3 Sensitivity analysis, pleiotropy test, and heterogeneity test results

Using the MR-Egger regression intercept method, it was shown that the intercept *p* values corresponding to all gut microbial taxa and functional pathways significantly associated with LI were greater than 0.05, indicating that there was no significant horizontal pleiotropy among the instrumental variables (SNPs). Scatter plot results indicated that for risk factors such as Deltaproteobacteria class, Bilophila genus and its species, and Veillonella_unclassified, the SNP effect points exhibited a positive association trend (slope > 0), suggesting an increased risk of LI. In contrast, for protective pathways such as PWY-6588, PWY-2941, and protective species like Ruminococcus_torques, the SNP effect points showed a negative association trend (slope < 0), indicating a reduced risk of LI, which is consistent with the findings of the IVW method. Heterogeneity testing (Cochran’s Q test) results indicated no significant heterogeneity among the instrumental variables (as shown in Supplementary material).

### 3.4 Reverse MR results

**Figure 5:**
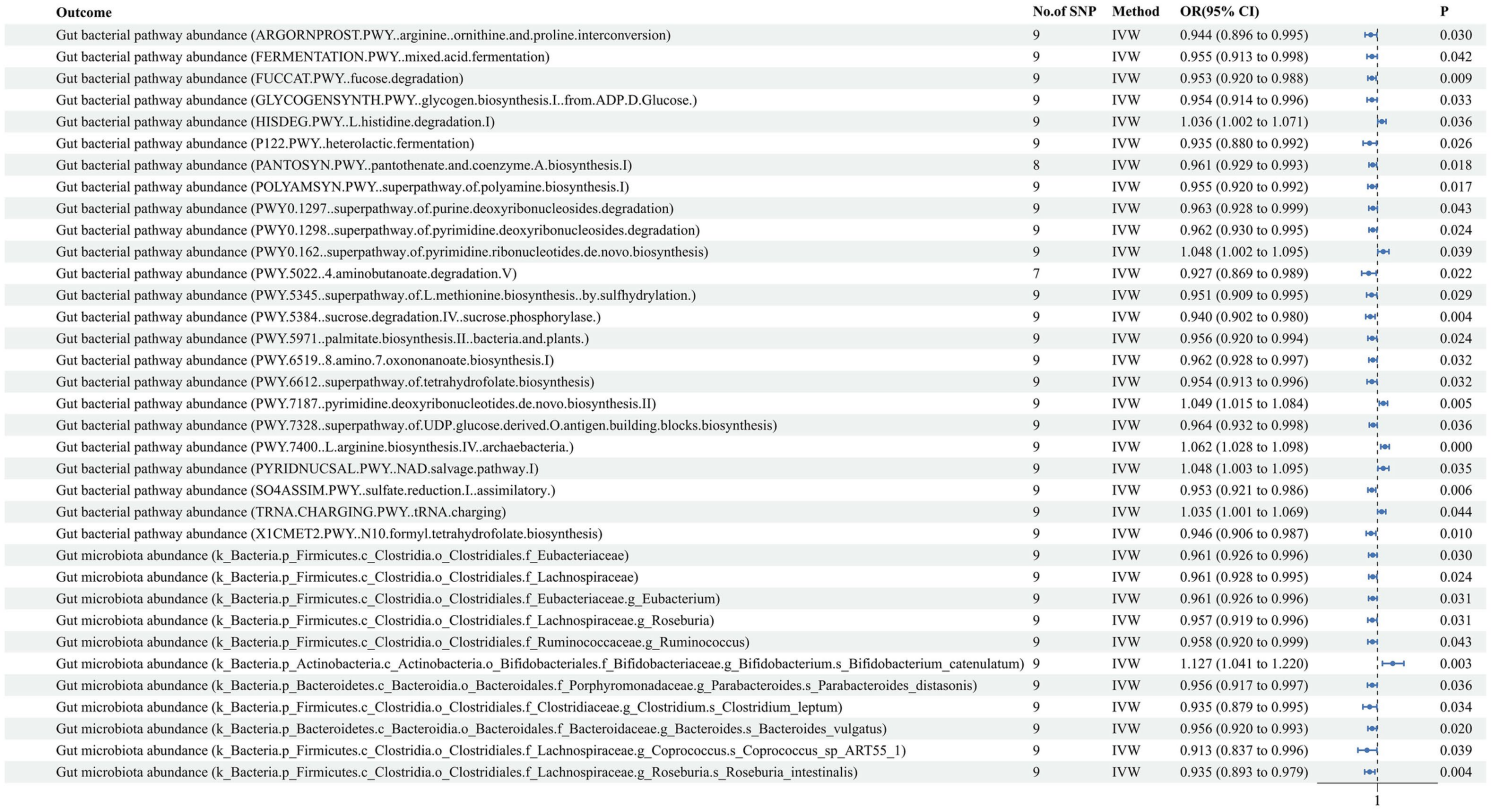
Reverse Mendelian randomization results with threshold of *p* < 0.05.

In the reverse MR analysis, a multilevel regression model was used to systematically assess the potential reverse causal effects between five major levels of gut microbial taxa and functional pathways with LI. The results showed that LI was significantly associated with multiple microbial metabolic pathways and several taxonomic units, with the vast majority of pathways exhibiting negative influence, while only a few pathways presented positive influence (Figure 5).

At the functional pathway level, LI showed significant negative associations with several core metabolic pathways, encompass key biological processes such as carbohydrate metabolism, amino acid conversion, nucleotide degradation, short-chain fatty acid (SCFA)-related metabolism, coenzyme and energy synthesis (e.g., ARGORNPROST. PWY: OR = 0.944, 95% CI 0.896–0.995, *p* = 0.030; FERMENTATION. PWY: OR = 0.955, 95% CI 0.913–0.998, *p* = 0.042). LI demonstrated significant positive associations with only a few pathways, primarily involving nucleotide synthesis, amino acid metabolism, and protein translation processes (e.g., HISDEG. PWY: OR = 1.036, 95% CI 1.002–1.071, *p* = 0.036; PWY0-162: OR = 1.048, 95% CI 1.002–1.096, *p* = 0.039).

At the taxonomic level, a broad negative correlation was similarly observed. LI was negatively correlated with several core microbial communities, widely involved in SCFA synthesis, complex carbohydrate degradation, and intestinal mucosal immune regulation, being important components for maintaining gut homeostasis, including Eubacteriaceae (OR = 0.961, 95% CI 0.926–0.996, *p* = 0.030), Lachnospiraceae (OR = 0.961, 95% CI 0.928–0.995, *p* = 0.024), Eubacterium (OR = 0.961, 95% CI 0.926–0.996, *p* = 0.030). Notably, only *Bifidobacterium catenulatum* was identified as a significant positive correlation factor influenced by LI (OR: 1.127, 95% CI 1.041–1.220, *p* = 0.003), with its increased abundance in LI group, indicating its unique role in disease occurrence.

In summary, reverse MR results show that genetic susceptibility of LI has a weak but significant effect in gut microbiota, may have cumulative significance at the population level. LI has systematically negatively correlated with multiple gut metabolic pathways, while a few pathways involved in nucleotide synthesis and amino acid metabolism exhibit positive correlation; at the microbial taxonomic level, the vast majority of microbial communities have negative correlation, with only *Bifidobacterium catenulatum* showing the opposite trend, suggesting significant differences in the effects of LI on different gut bacteria.

### 3.5 Microbiota 16S rRNA sequencing results

**Figure 6:**
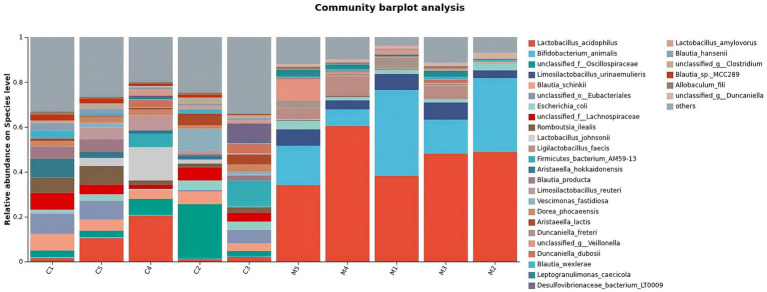
Species composition analysis at the species level.

We further explored the relationship between gut microbiota and LI. Figure 6 shows the microbial composition at the species level in Groups C and M. At the species level, the main dominant species were Lactobacillus_acidophilus and Bifidobacterium_animalis, accounting for 46 and 22.2% of the total bacterial abundance, respectively. This finding further indicates that LI affects the composition of the gut microbiota.

**Figure 7:**
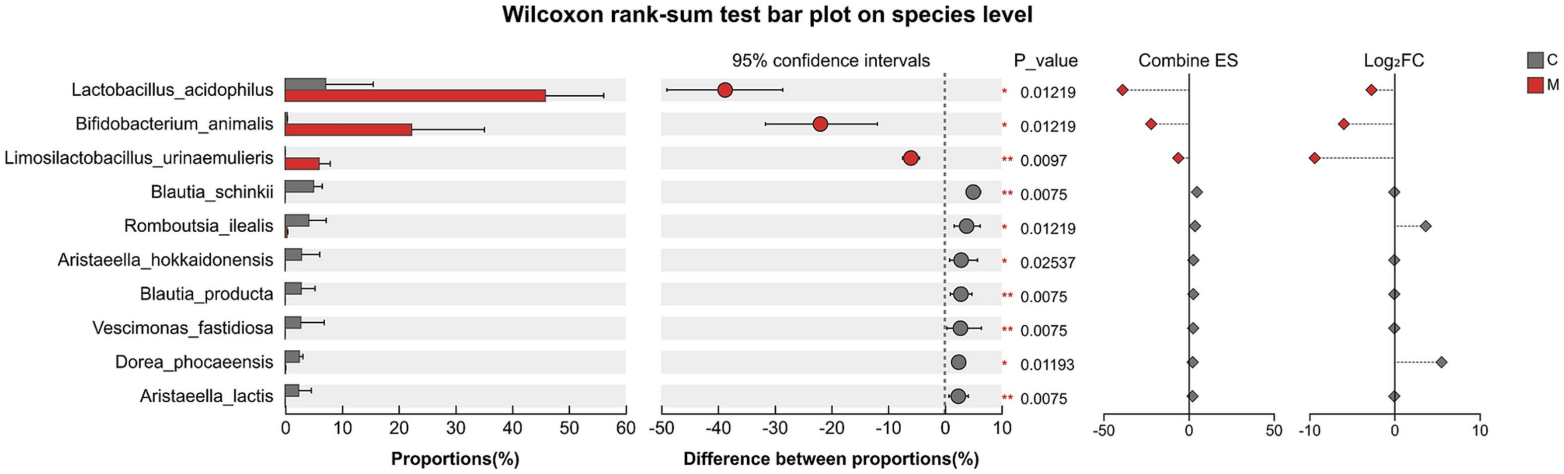
Inter-group difference analysis at the genus level with threshold of *p* < 0.05.

To identify microbial genera potentially involved in disease occurrence, we compared the differences between the two groups and screened for differentially abundant genera potentially related to the disease process (e.g., with potential pathogenicity). Inter-group difference analysis showed significant differences in several genera in Group M compared to Group C, including Lactobacillus, Bifidobacterium, Blautia, and Aristaeella (Figure 7). Notably, the 16S rRNA sequencing data in this study indicated an abnormal increase in Bifidobacterium abundance and decrease in Blautia abundance in Group M, highly consistent with the effects observed in the MR analysis, further reinforcing its credibility as a potential pathogenic-related strain from an independent data source.

**Figure 8:**
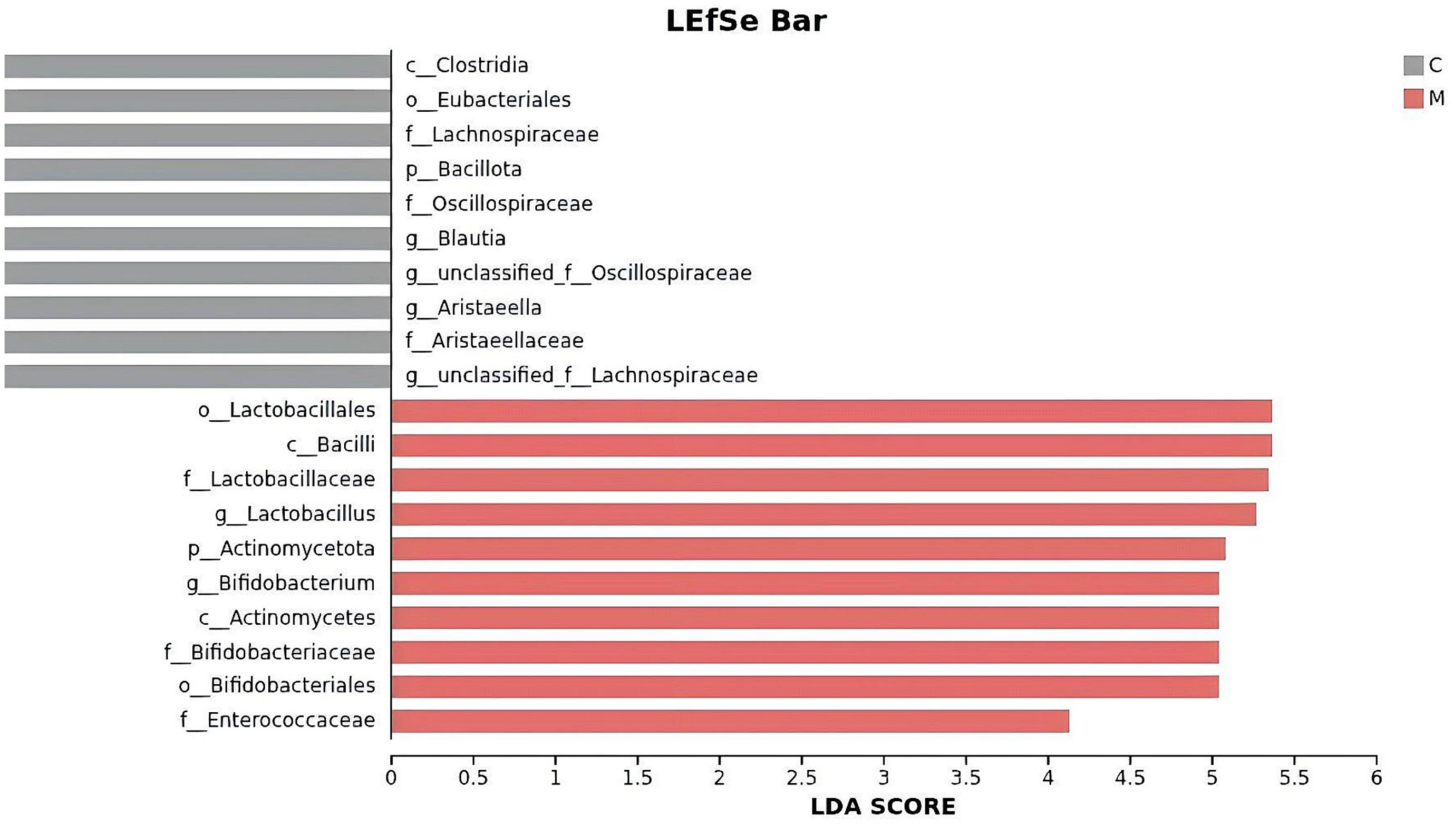
Linear discriminant analysis effect size (LEfSe) inter-group difference analysis linear discriminant analysis (LDA) plot.

Further research utilized LEfSe to identify differentially abundant genera to determine the characteristic changes in the gut microbiota of the experimental samples. The results showed that Bacillota, Clostridia, and Eubacteriales were significantly enriched in Group C, while Bifidobacterium, Bacilli, Lactobacillales, and Lactobacillaceae were significantly enriched in Group M (Figure 8).

**Figure 9:**
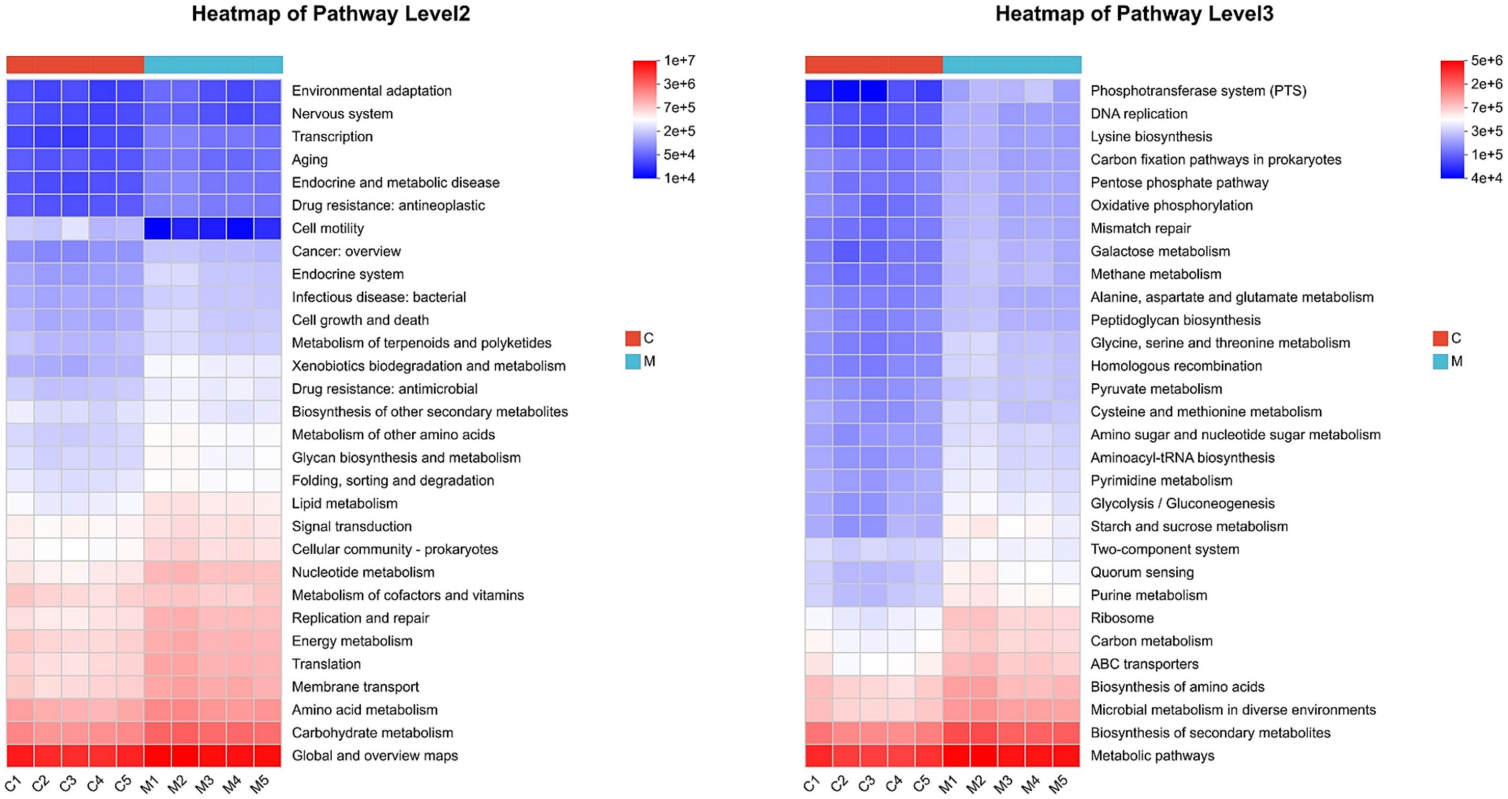
KEGG functional heatmap.

Additionally, functional prediction analysis of the gut microbiota’s 16S rRNA sequencing data was performed using PICRUSt2. At the Kyoto Encyclopedia of Genes and Genomes (KEGG) Level 2 level, amino acid metabolism, carbohydrate metabolism, and global and overview maps were the main metabolic pathways in which the microbes participated (Figure 9); while at the KEGG Level 3 level, biosynthesis of secondary metabolites, metabolic pathways, microbial metabolism in diverse environments, and galactose metabolism were also major metabolic pathways in which the microbes participated (Figure 9).

## 4 Discussion

This study investigates the impact of gut microbiota on LI and emphasizes the importance of establishing causal relationships. Our findings reveal that specific microbial taxa, such as Deltaproteobacteria and Bilophila are associated with an increased risk of LI, while others, like Paraprevotella and Blautia, exhibit protective effects. Additionally, the results from reverse MR analysis suggest that LI has influence on various metabolic pathways and gut microbiota, indicating a complex interplay between gut microbiota and disease susceptibility. Overall, this research underscores the necessity of understanding the causal mechanisms of gut microbiota and LI, paving the way for potential therapeutic interventions.

Our study’s findings align with previous research that has explored the relationship between gut microbiota and lactose metabolism, emphasizing specific microbial taxa associated with LI. For instance, an investigation involving 150 participants demonstrated that a higher abundance of *Faecalibacterium prausnitzii* is correlated with reduced symptoms of lactose malabsorption, reinforcing our findings that certain microbial populations may mitigate LI symptoms (9).

However, the impact of certain bacteria on LI remains controversial, as exemplified by Bifidobacterium, which is traditionally perceived as a probiotic. A study utilized metagenomic sequencing in a cohort of 200 individuals, revealing that Lactobacillus and Bifidobacterium species are enriched in lactose-tolerant individuals, suggesting a protective role against LI (10). But another study published in *Gut* revealed that individuals with LI exhibit a significantly increased abundance of the Bifidobacterium genus in their gut microbiota. Moreover, the abundance of Bifidobacterium is positively correlated with dairy intake and gastrointestinal symptoms (including abdominal pain, abdominal discomfort, and bloating). Further analysis demonstrated that Bifidobacterium partially mediates the association between dairy intake and gastrointestinal symptoms (11). Results from our MR analysis and microbiota sequencing support the latter interpretation.

Bifidobacterium is generally considered capable of utilizing lactose. Why might it be a risk factor? One possibility is that individuals with a specific genetic background (leading to high Bifidobacterium abundance) may also possess certain immune characteristics that make them more susceptible to LI. Alternatively, excessive fermentation of lactose by Bifidobacterium could produce gas, leading to symptoms such as bloating. Rats, which are natural models of LI, have not been reported to carry SNPs associated with persistent lactase production. Due to the intake of large amounts of undigested lactose, lactose-consuming Bifidobacterium naturally blooms. Supporting our findings, a study published in *Nature Genetics* revealed that the lactase locus (rs182549), which is associated with LI in European adults, is strongly linked to microbial abundance. Variations at this locus correlate with Bifidobacterium abundance, but these associations differ depending on dairy intake. In lactase-persistent adults, Bifidobacterium abundance is not influenced by dairy consumption. However, among lactose-intolerant individuals following a regular dairy diet, Bifidobacterium abundance increases significantly (12). The interaction between the human host and the gut microbiota deserves further investigation.

Moreover, a study on a Chinese cohort of 300 individuals, using machine learning to identify gut microbial signatures that can predict LI. Their results highlighted the protective effects of specific taxa such as Eubacterium and Ruminococcus against LI, which supports our discovery of the inverse relationship between specific gut microbes and LI risk (13). Another MR analysis identified that family Veillonellaceae, genus Oxalobacter and Senegalimassilia were protective against LI, whereas genus Anaerotruncus, *Eubacterium rectale* group and Ruminococcus 2 were found to be risk factors for LI (5). The discrepancy between these findings and our results may be attributed to differences in the cohorts used and notable batch effects, which highlights the necessity for *in vivo* validation. Additionally, a systematic review synthesized evidence from various studies, confirming that probiotics can alleviate symptoms of LI by enhancing the gut microbiota composition, thus corroborating our results on the beneficial effects of certain microbial species (14).

In contrast, some studies present findings that diverge from our results. For instance, a study involving 100 participants indicated that dietary lactose does not significantly alter the gut microbiota in those with LI, suggesting that the response may be more complex than previously understood (15). Furthermore, a research found no significant differences in the microbial composition between lactose-intolerant and tolerant individuals, raising questions about the consistency of microbial involvement in LI (16). The discrepancies may arise from variations in study design, sample populations, and methodologies employed to assess gut microbiota composition.

Additionally, another study highlighted that certain dietary fibers can modulate gut microbiota in lactose-intolerant individuals, emphasizing that dietary interventions might overshadow the microbial effects observed in our research (17). This suggests that factors such as diet and other environmental influences play a crucial role in shaping the gut microbiome and may need to be considered when evaluating the relationship between gut microbiota and LI.

In summary, while our study aligns with various findings that support the protective role of specific gut microbiota against LI, the contrasting results from certain studies indicate the need for further investigation to elucidate the complexities of the gut microbiome’s role in LI. These discrepancies highlight the importance of considering dietary factors, genetic variations, and methodological differences in future research endeavors.

The results of our study illustrate the significant impact of microbial taxa such as Deltaproteobacteria and Bilophila on the risk of LI, may through their involvement in metabolic pathways that modulate the gut environment. Deltaproteobacteria, specifically, has been associated with the breakdown of complex carbohydrates, including lactose, thereby influencing lactose metabolism and tolerability in susceptible individuals (18). The role of Bilophila, particularly *Bilophila wadsworthia*, further emphasizes this connection, as its abundance correlates positively with increased LI risk, suggesting a detrimental effect on gut health and lactose digestion (19). This aligns with findings that indicate certain microbial species may enhance the gut’s ability to ferment lactose, thus modulating the microbiota composition and potentially alleviating LI symptoms (20).

Moreover, the presence of specific metabolic pathways, such as the fermentation of SCFAs, may plays a crucial role in maintaining gut homeostasis and influencing the risk of LI. SCFAs are known to promote a healthy gut environment and mitigate inflammation, which is often exacerbated in LI (21). The observed protective effects of some microbial taxa, such as Paraprevotella and Blautia, underscore the complexity of these interactions, where certain species may confer resilience against LI while others may increase susceptibility (22). MR analysis indicates that the Blautia genus exerts a protective effect against LI. Concurrently, high dietary lactose intake has been observed to reduce the abundance of Blautia. Crucially, reverse MR analysis has ruled out the possibility that LI itself causes a decrease in Blautia abundance, suggesting this alteration is not induced by the disease state. A potential mechanistic explanation is that high lactose intake alters the intestinal microenvironment, thereby inhibiting the growth of Blautia. A reduction in Blautia abundance can decrease the production of SCFAs, which could affect intestinal barrier integrity and anti-inflammatory immune responses. The diminished protective capacity reduces the buffering effect against adverse intestinal reactions triggered by undigested lactose, ultimately leading to the manifestation or exacerbation of LI symptoms (23, 24). This duality highlights the importance of a balanced gut microbiome in managing digestive health.

Our sensitivity analysis further supports these findings, revealing that the associations between microbial taxa and LI are robust, with minimal evidence of horizontal pleiotropy. The absence of significant heterogeneity across our analyses reinforces the reliability of our results (25). This clarity in the data emphasizes the potential for targeted probiotic therapies that leverage beneficial microbes to improve lactose tolerance in affected populations, thereby enhancing dietary options and overall health outcomes (26).

In conclusion, our study elucidates the intricate relationship between gut microbiota composition, metabolic pathways, and the risk of LI. The significant roles played by specific microbial groups, particularly Deltaproteobacteria, Bilophila. Bifidobacterium and Blautia, highlight the importance of understanding gut ecology in the context of LI. Future research could aim to explore the therapeutic potential of manipulating gut microbiota to alleviate LI and improve dietary tolerability among individuals affected by this condition (27).

This study leverages a two-sample MR design, coupled with high-throughput 16S rRNA sequencing, to explore the causal relationship between gut microbiota and LI. Unlike previous studies that primarily relied on observational data, which are often confounded by unmeasured variables, our approach minimizes bias by using genetic variants as instrumental variables. This methodology allows for a more precise estimation of causal effects, enhancing the reliability of our findings. Our results reveal specific bacterial taxa and metabolic pathways associated with LI, providing novel insights that underscore the importance of gut microbiota in metabolic health. This comprehensive approach not only strengthens the validity of our findings but also contributes significantly to the understanding of the complex interplay between gut microbiota and lactose metabolism, which remains underexplored in existing literature (28–30).

However, our study still has limitations. The results of the animal experiment only represent a physiological response model; direct application to clinical practice will require validation through human intervention studies. The predictions based on 16S rRNA data and PICRUSt2 are indeed inferential and have limitations, and future research involving bacterial gavage with appropriate controls will be needed for validation.

## 5 Conclusion

This study provides insights into the causal relationship between gut microbiota and LI, identifying specific microbial taxa and metabolic pathways that may influence the risk of developing LI. The findings indicate that certain gut bacteria, such as members of the Deltaproteobacteria phylum and Bilophila are associated with increased risk, while other taxa, including Paraprevotella and Blautia, may have a protective effect. And our study suggests that LI also genetically influences host gut microbiota and metabolism pathways. Additionally, the study highlights the potential role of various metabolic pathways in modulating the risk of LI, suggesting that gut microbiota composition and function play significant roles in this condition. The significance of this research lies in its contribution to our understanding of the gut microbiota-lactose intolerance connection, which can inform future therapeutic strategies aimed at modulating gut microbiota to prevent or alleviate symptoms of LI.

## Data Availability

The data presented in the study are deposited in the CNCB-NGDC Genome Sequence Archive (GSA) repository, accession number CRA043512.

## Footnotes

1
https://storage.googleapis.com/finngen-public-data-r12/summary_stats/release/finngen_R12_E4_LACTONAS.gz

2
https://v.majorbio.com
