# Supplementary material for: Causal interplay between lactose intolerance and gut microbiota: a combined bidirectional Mendelian randomization and in vivo validation study
Source: Front Nutr. 2026 Jun 1;13:1803337. doi: 10.3389/fnut.2026.1803337 (PMC13265576; doi:10.3389/fnut.2026.1803337)
Supplement: Supplementary file 1 [file Data_Sheet_1.zip › supplementary materials/Forward/forest plot/ebi-a-GCST90027447.finngen_R12_E4_LACTONAS.pdf]

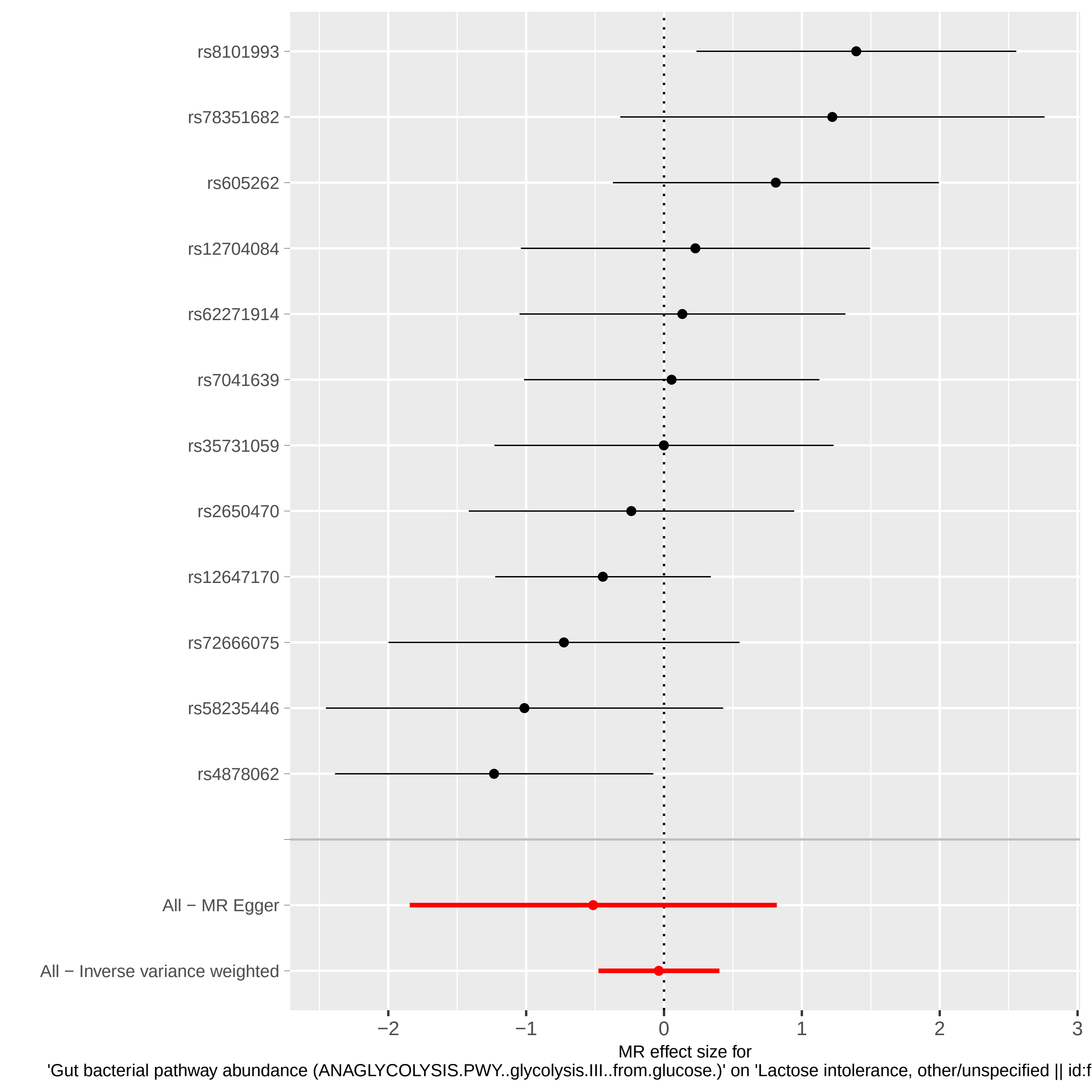

rs8101993
rs78351682
rs605262
rs12704084
rs62271914
rs7041639
rs35731059
rs2650470
rs12647170
rs72666075
rs58235446
rs4878062
All − MR Egger
All − Inverse variance weighted
−2
−1
0
1
2
3
MR effect size for
'Gut bacterial pathway abundance (ANAGLYCOLYSIS.PWY..glycolysis.III..from.glucose.)' on 'Lactose intolerance, other/unspecified || id:f
