# Supplementary material for: Causal interplay between lactose intolerance and gut microbiota: a combined bidirectional Mendelian randomization and in vivo validation study
Source: Front Nutr. 2026 Jun 1;13:1803337. doi: 10.3389/fnut.2026.1803337 (PMC13265576; doi:10.3389/fnut.2026.1803337)
Supplement: Supplementary file 1 [file Data_Sheet_1.zip › supplementary materials/Forward/forest plot/ebi-a-GCST90027454.finngen_R12_E4_LACTONAS.pdf]

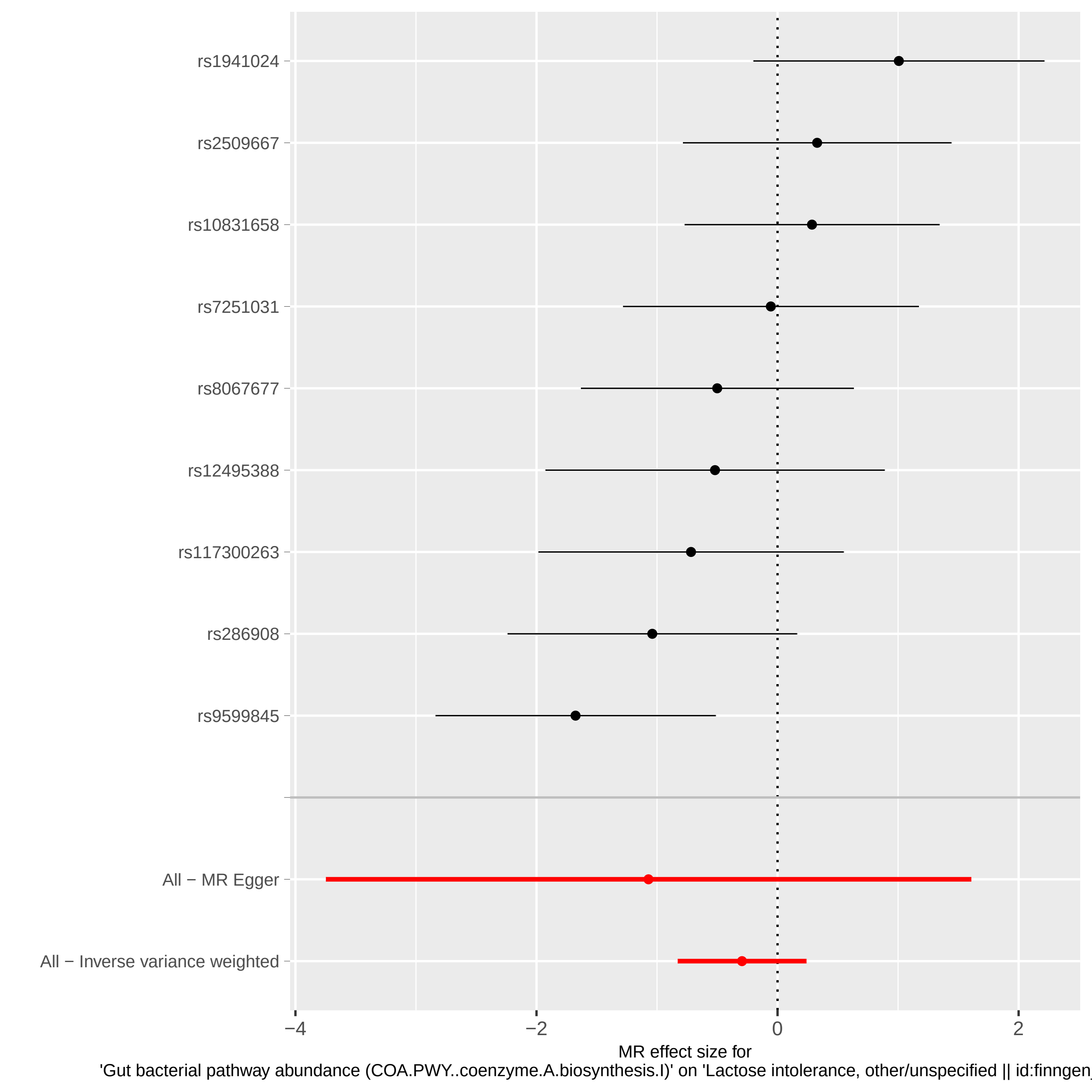

rs1941024
rs2509667
rs10831658
rs7251031
rs8067677
rs12495388
rs117300263
rs286908
rs9599845
All − MR Egger
All − Inverse variance weighted
−4
−2
0
2
MR effect size for
'Gut bacterial pathway abundance (COA.PWY..coenzyme.A.biosynthesis.I)' on 'Lactose intolerance, other/unspecified || id:finngen_
