# Supplementary material for: Causal interplay between lactose intolerance and gut microbiota: a combined bidirectional Mendelian randomization and in vivo validation study
Source: Front Nutr. 2026 Jun 1;13:1803337. doi: 10.3389/fnut.2026.1803337 (PMC13265576; doi:10.3389/fnut.2026.1803337)
Supplement: Supplementary file 1 [file Data_Sheet_1.zip › supplementary materials/Forward/forest plot/ebi-a-GCST90027457.finngen_R12_E4_LACTONAS.pdf]

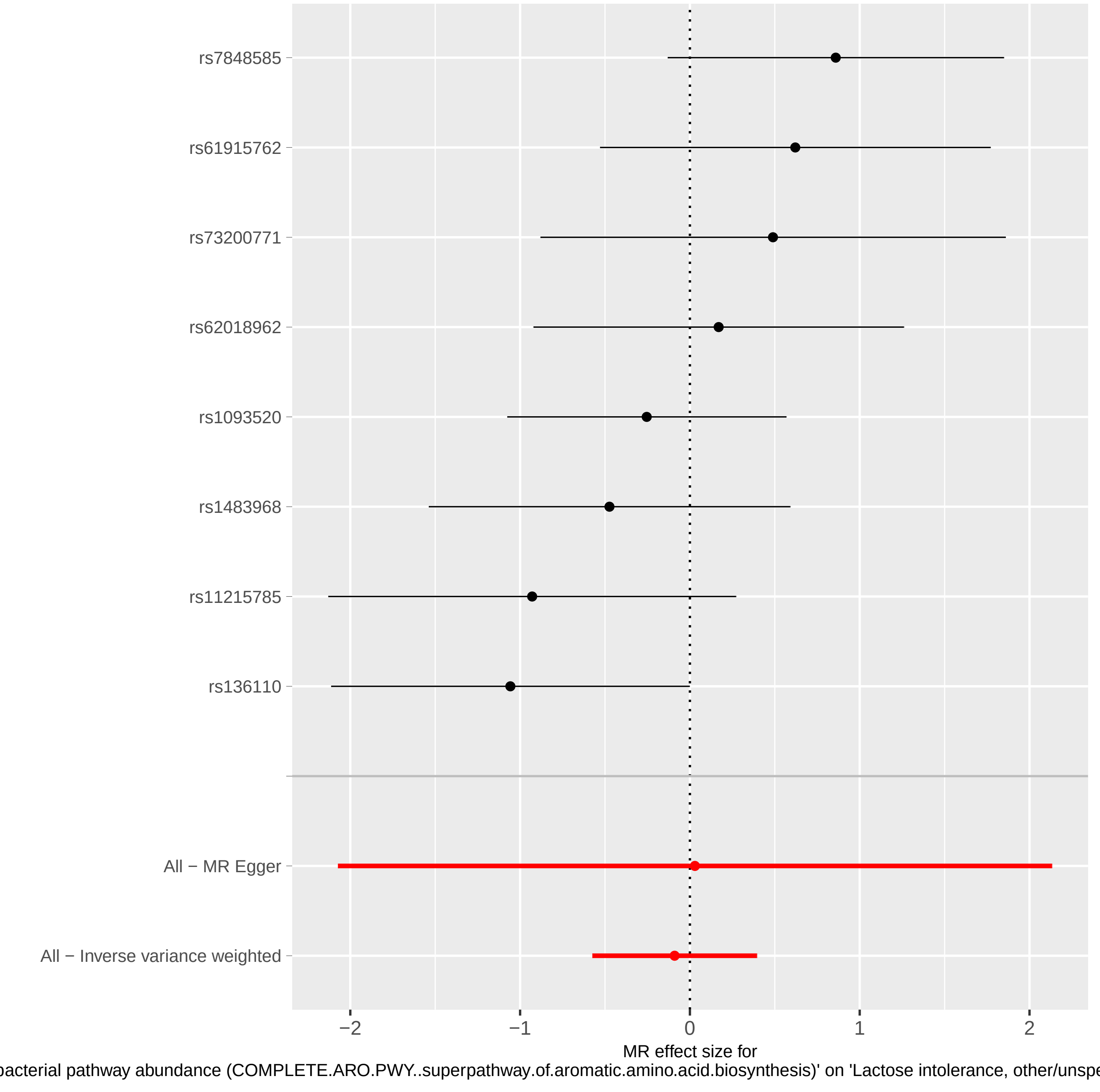

rs7848585
rs61915762
rs73200771
rs62018962
rs1093520
rs1483968
rs11215785
rs136110
All − MR Egger
All − Inverse variance weighted
−2
−1
0
1
2
MR effect size for
bacterial pathway abundance (COMPLETE.ARO.PWY..superpathway.of.aromatic.amino.acid.biosynthesis)' on 'Lactose intolerance, other/unspe
