# Supplementary material for: Causal interplay between lactose intolerance and gut microbiota: a combined bidirectional Mendelian randomization and in vivo validation study
Source: Front Nutr. 2026 Jun 1;13:1803337. doi: 10.3389/fnut.2026.1803337 (PMC13265576; doi:10.3389/fnut.2026.1803337)
Supplement: Supplementary file 1 [file Data_Sheet_1.zip › supplementary materials/Forward/forest plot/ebi-a-GCST90027480.finngen_R12_E4_LACTONAS.pdf]

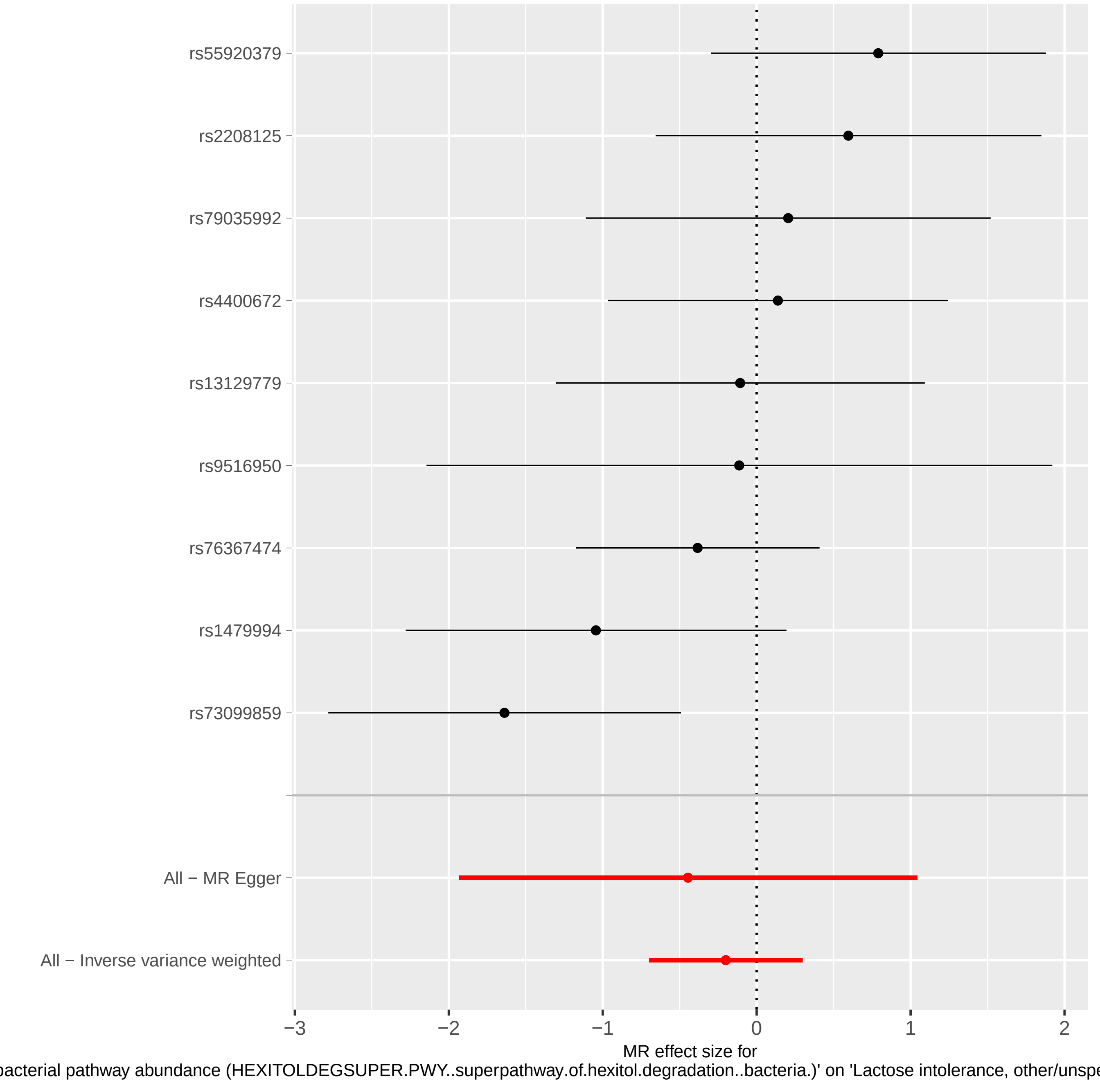
rs55920379
rs2208125
rs79035992
rs4400672
rs13129779
rs9516950
rs76367474
rs1479994
rs73099859
All − MR Egger
All − Inverse variance weighted
−3
−2
−1
0
1
2
MR effect size for
bacterial pathway abundance (HEXITOLDEGSUPER.PWY..superpathway.of.hexitol.degradation..bacteria.)' on 'Lactose intolerance, other/unspe
