# Supplementary material for: Causal interplay between lactose intolerance and gut microbiota: a combined bidirectional Mendelian randomization and in vivo validation study
Source: Front Nutr. 2026 Jun 1;13:1803337. doi: 10.3389/fnut.2026.1803337 (PMC13265576; doi:10.3389/fnut.2026.1803337)
Supplement: Supplementary file 1 [file Data_Sheet_1.zip › supplementary materials/Forward/forest plot/ebi-a-GCST90027484.finngen_R12_E4_LACTONAS.pdf]

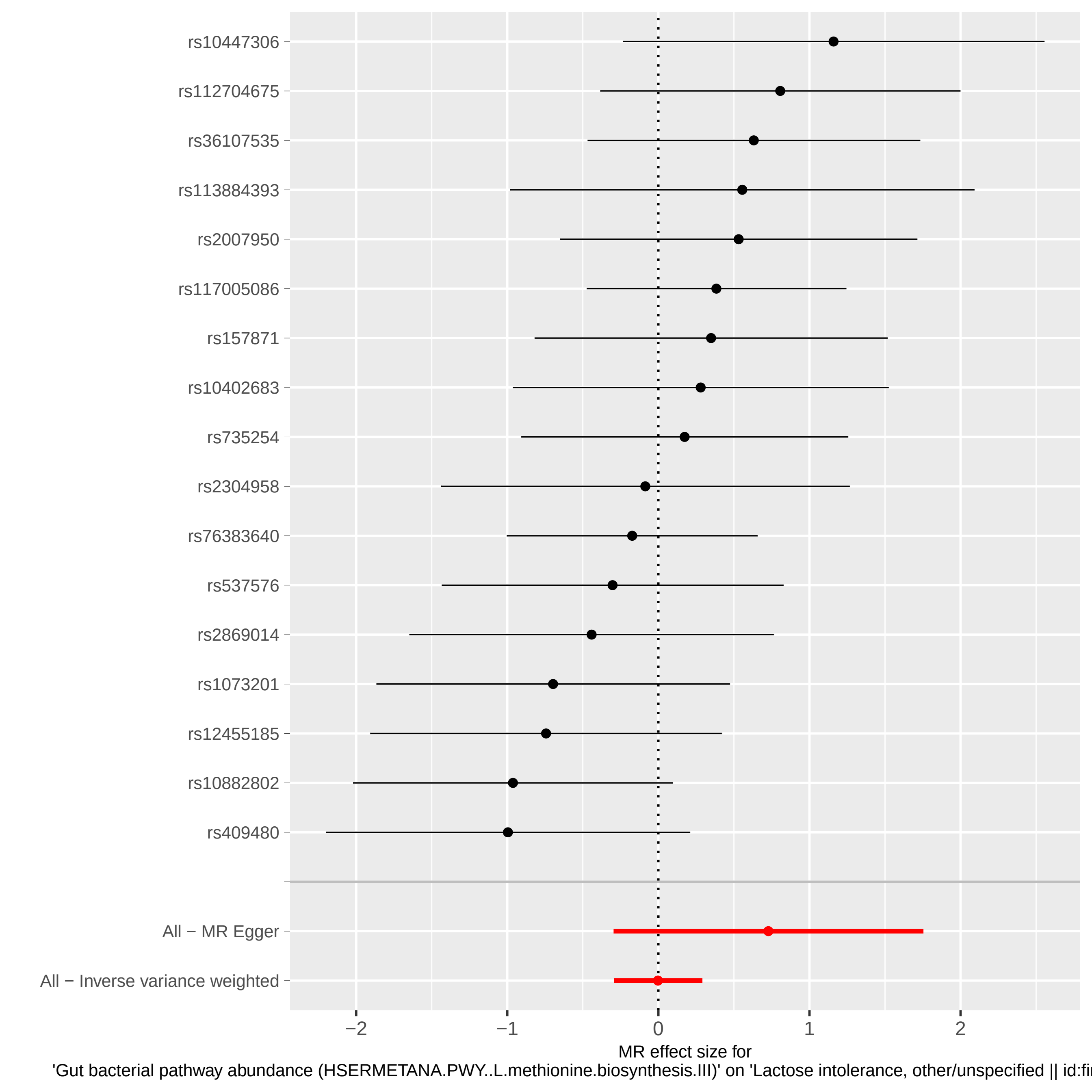
rs10447306
rs112704675
rs36107535
rs113884393
rs2007950
rs117005086
rs157871
rs10402683
rs735254
rs2304958
rs76383640
rs537576
rs2869014
rs1073201
rs12455185
rs10882802
rs409480
All − MR Egger
All − Inverse variance weighted
−2
−1
0
1
2
MR effect size for
'Gut bacterial pathway abundance (HSERMETANA.PWY..L.methionine.biosynthesis.III)' on 'Lactose intolerance, other/unspecified || id:fi
