# Supplementary material for: Causal interplay between lactose intolerance and gut microbiota: a combined bidirectional Mendelian randomization and in vivo validation study
Source: Front Nutr. 2026 Jun 1;13:1803337. doi: 10.3389/fnut.2026.1803337 (PMC13265576; doi:10.3389/fnut.2026.1803337)
Supplement: Supplementary file 1 [file Data_Sheet_1.zip › supplementary materials/Forward/forest plot/ebi-a-GCST90027488.finngen_R12_E4_LACTONAS.pdf]

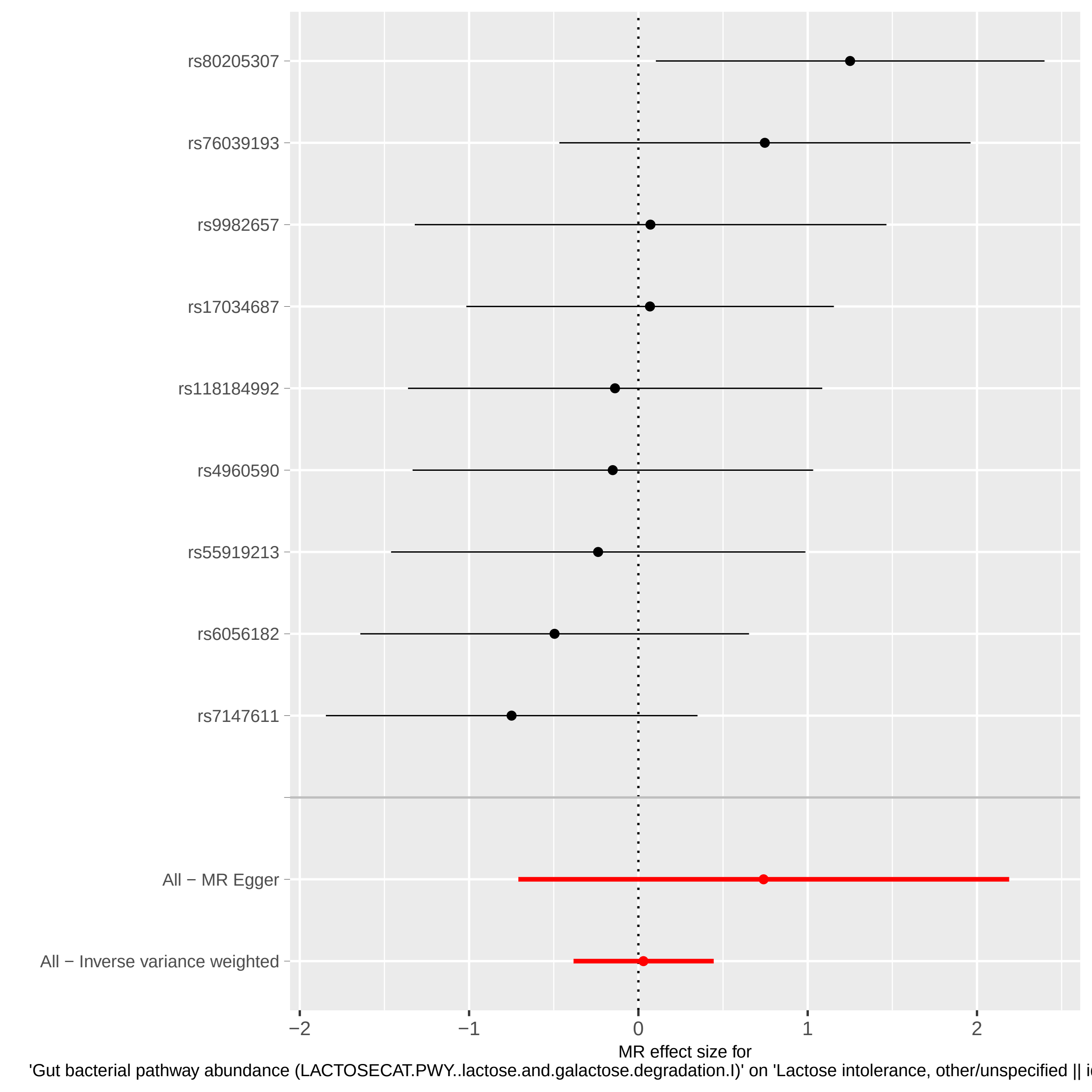

rs80205307
rs76039193
rs9982657
rs17034687
rs118184992
rs4960590
rs55919213
rs6056182
rs7147611
All − MR Egger
All − Inverse variance weighted
−2
−1
0
1
2
MR effect size for
'Gut bacterial pathway abundance (LACTOSECAT.PWY..lactose.and.galactose.degradation.I)' on 'Lactose intolerance, other/unspecified || i
