# Supplementary material for: Causal interplay between lactose intolerance and gut microbiota: a combined bidirectional Mendelian randomization and in vivo validation study
Source: Front Nutr. 2026 Jun 1;13:1803337. doi: 10.3389/fnut.2026.1803337 (PMC13265576; doi:10.3389/fnut.2026.1803337)
Supplement: Supplementary file 1 [file Data_Sheet_1.zip › supplementary materials/Forward/forest plot/ebi-a-GCST90027491.finngen_R12_E4_LACTONAS.pdf]

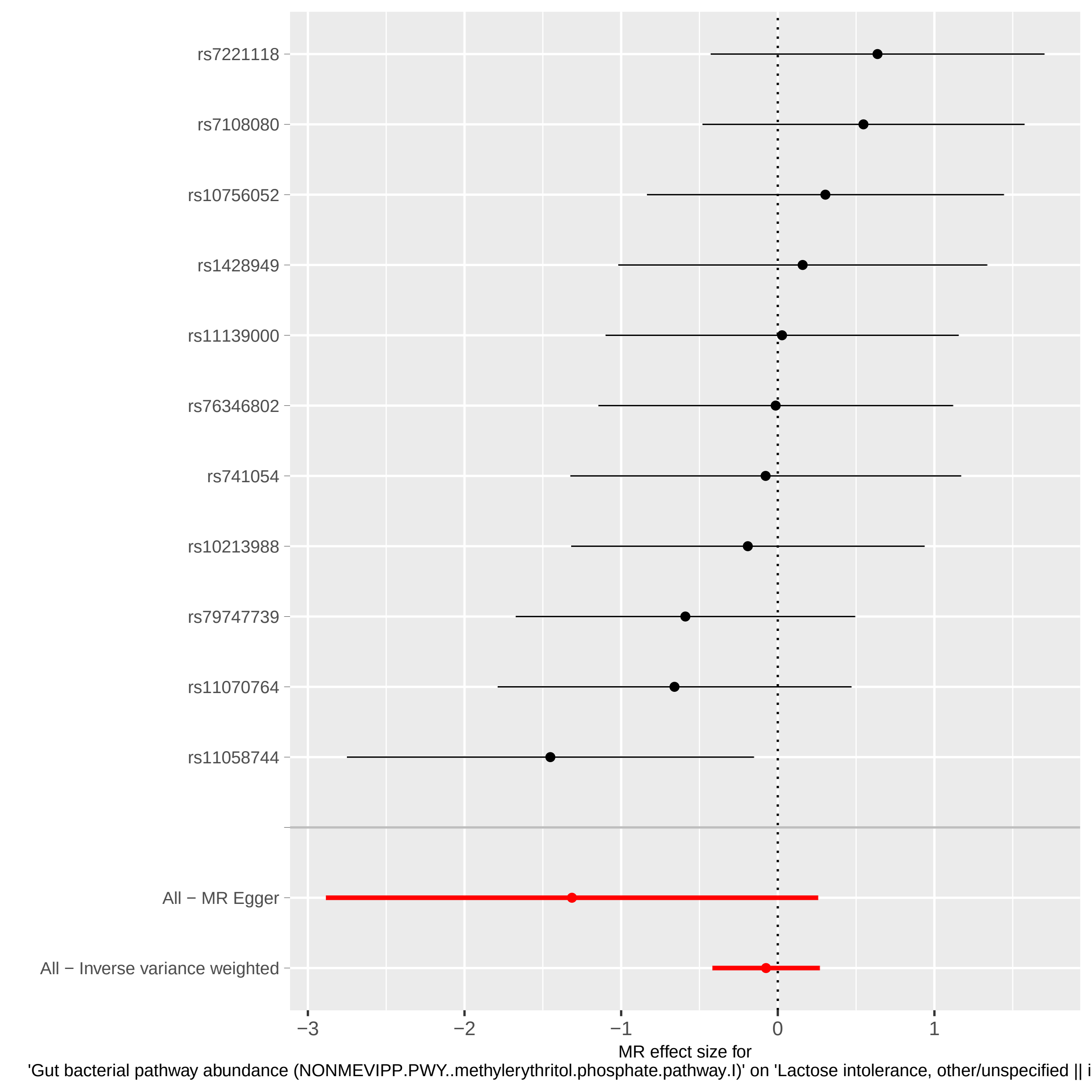
rs7221118
rs7108080
rs10756052
rs1428949
rs11139000
rs76346802
rs741054
rs10213988
rs79747739
rs11070764
rs11058744
All − MR Egger
All − Inverse variance weighted
−3
−2
−1
0
1
MR effect size for
'Gut bacterial pathway abundance (NONMEVIPP.PWY..methylerythritol.phosphate.pathway.I)' on 'Lactose intolerance, other/unspecified || i
