# Supplementary material for: Causal interplay between lactose intolerance and gut microbiota: a combined bidirectional Mendelian randomization and in vivo validation study
Source: Front Nutr. 2026 Jun 1;13:1803337. doi: 10.3389/fnut.2026.1803337 (PMC13265576; doi:10.3389/fnut.2026.1803337)
Supplement: Supplementary file 1 [file Data_Sheet_1.zip › supplementary materials/Forward/forest plot/ebi-a-GCST90027492.finngen_R12_E4_LACTONAS.pdf]

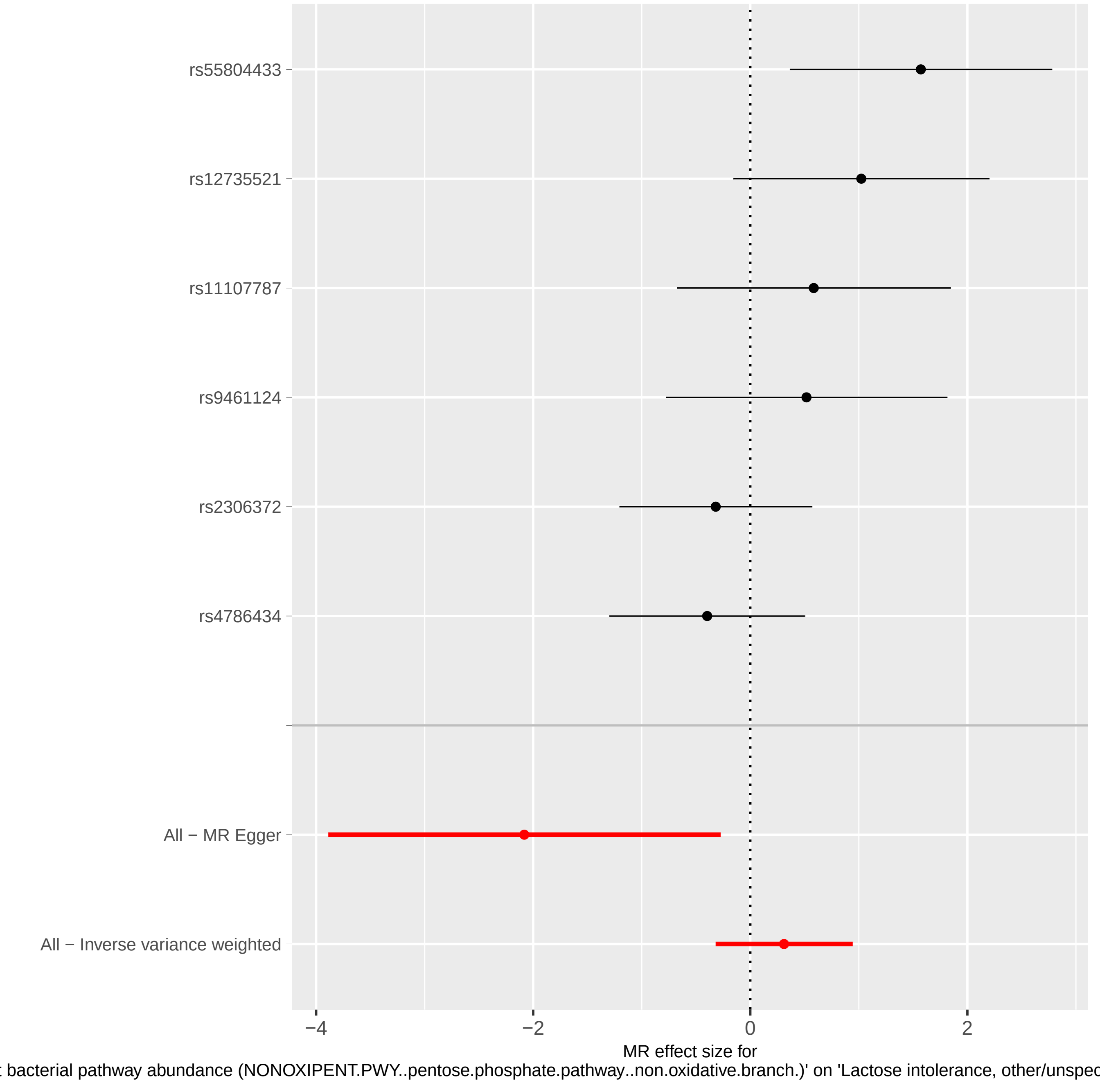
rs55804433
rs12735521
rs11107787
rs9461124
rs2306372
rs4786434
All − MR Egger
All − Inverse variance weighted
−4
−2
0
2
MR effect size for
bacterial pathway abundance (NONOXIPENT.PWY..pentose.phosphate.pathway..non.oxidative.branch.)' on 'Lactose intolerance, other/unspec
