# Supplementary material for: Causal interplay between lactose intolerance and gut microbiota: a combined bidirectional Mendelian randomization and in vivo validation study
Source: Front Nutr. 2026 Jun 1;13:1803337. doi: 10.3389/fnut.2026.1803337 (PMC13265576; doi:10.3389/fnut.2026.1803337)
Supplement: Supplementary file 1 [file Data_Sheet_1.zip › supplementary materials/Forward/forest plot/ebi-a-GCST90027505.finngen_R12_E4_LACTONAS.pdf]

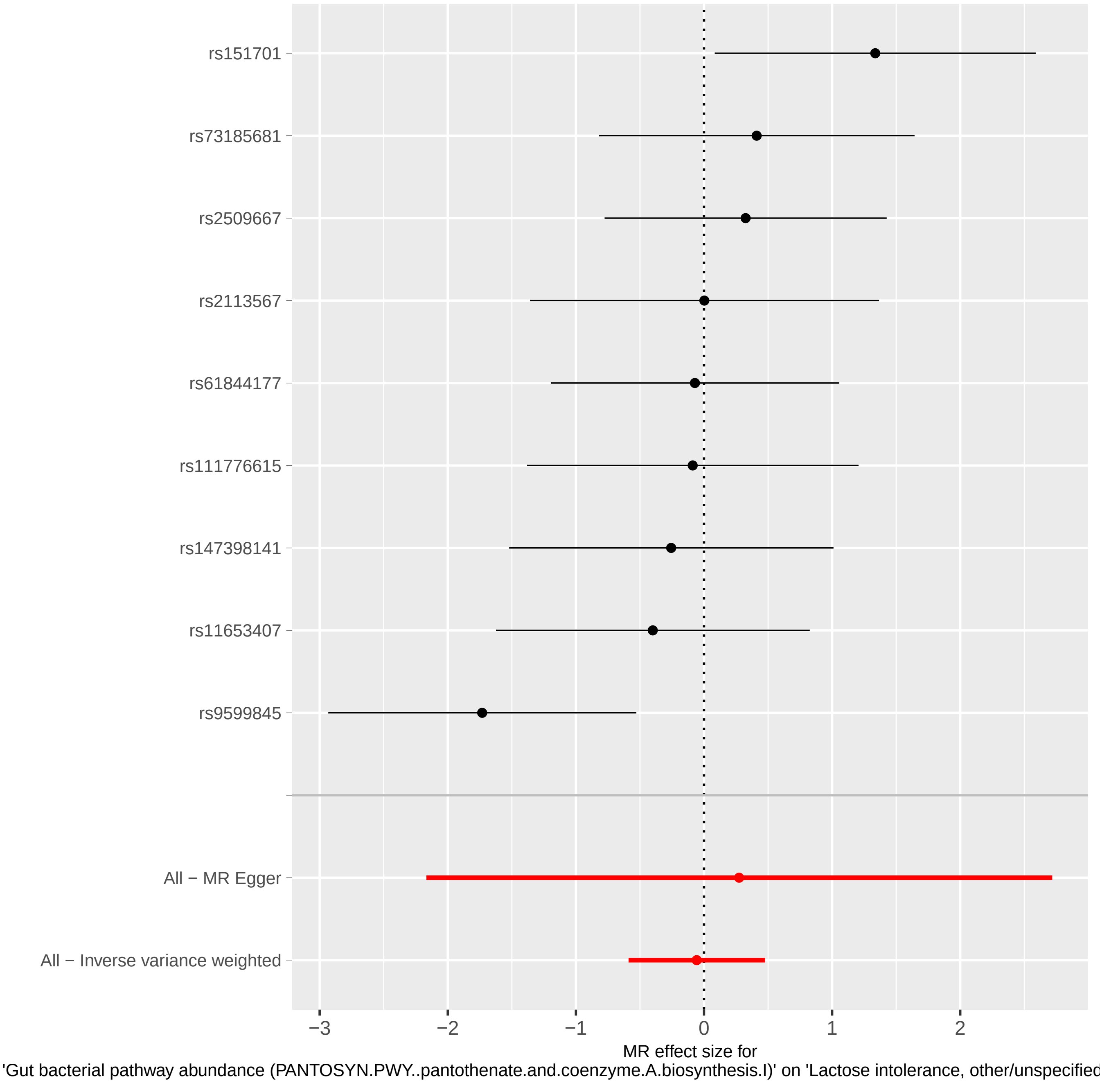

rs151701
rs73185681
rs2509667
rs2113567
rs61844177
rs111776615
rs147398141
rs11653407
rs9599845
All − MR Egger
All − Inverse variance weighted
−3
−2
−1
0
1
2
MR effect size for
'Gut bacterial pathway abundance (PANTOSYN.PWY..pantothenate.and.coenzyme.A.biosynthesis.I)' on 'Lactose intolerance, other/unspecified
