# Supplementary material for: Causal interplay between lactose intolerance and gut microbiota: a combined bidirectional Mendelian randomization and in vivo validation study
Source: Front Nutr. 2026 Jun 1;13:1803337. doi: 10.3389/fnut.2026.1803337 (PMC13265576; doi:10.3389/fnut.2026.1803337)
Supplement: Supplementary file 1 [file Data_Sheet_1.zip › supplementary materials/Forward/forest plot/ebi-a-GCST90027533.finngen_R12_E4_LACTONAS.pdf]

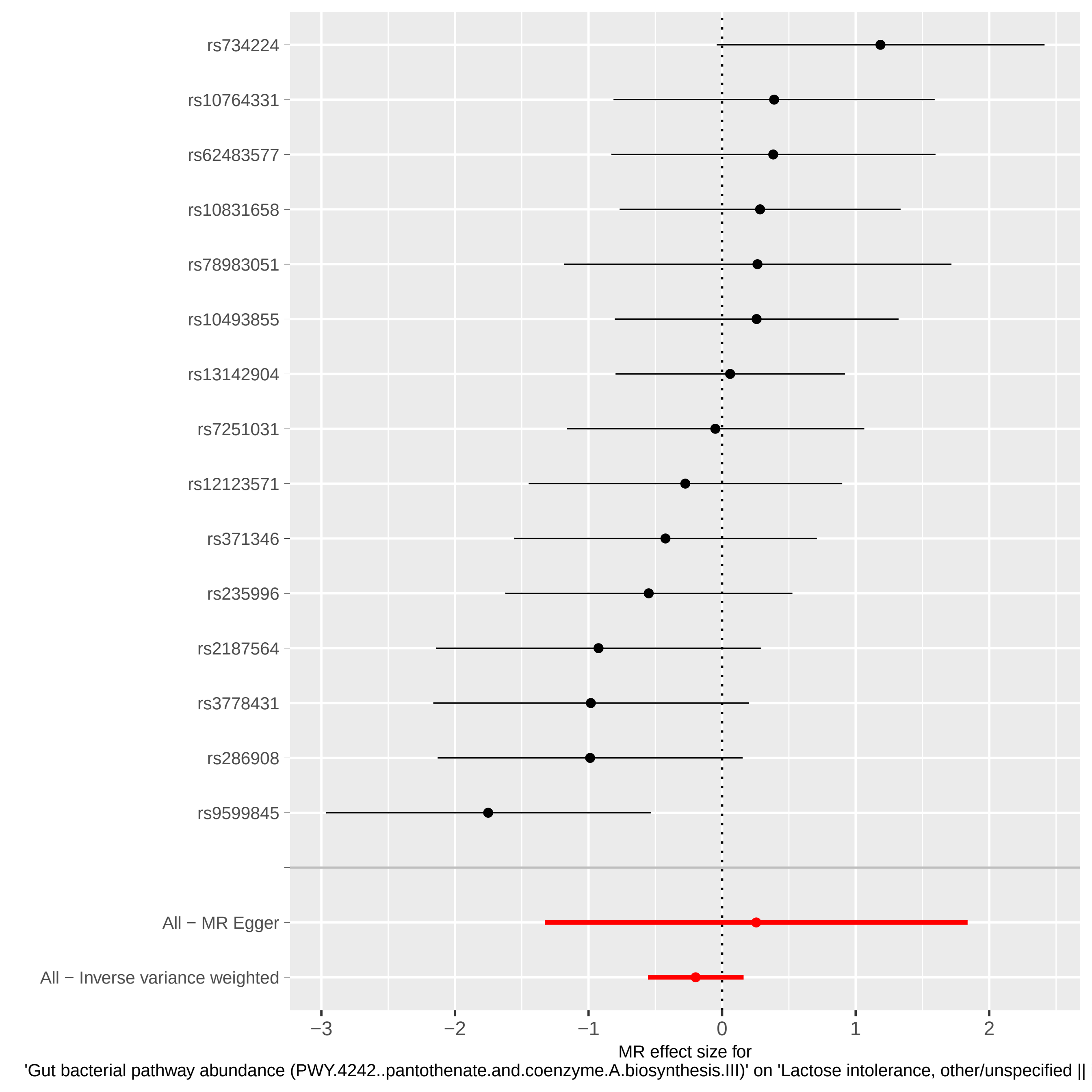
rs734224
rs10764331
rs62483577
rs10831658
rs78983051
rs10493855
rs13142904
rs7251031
rs12123571
rs371346
rs235996
rs2187564
rs3778431
rs286908
rs9599845
All − MR Egger
All − Inverse variance weighted
−3
−2
−1
0
1
2
MR effect size for
'Gut bacterial pathway abundance (PWY.4242..pantothenate.and.coenzyme.A.biosynthesis.III)' on 'Lactose intolerance, other/unspecified ||
