# Supplementary material for: Causal interplay between lactose intolerance and gut microbiota: a combined bidirectional Mendelian randomization and in vivo validation study
Source: Front Nutr. 2026 Jun 1;13:1803337. doi: 10.3389/fnut.2026.1803337 (PMC13265576; doi:10.3389/fnut.2026.1803337)
Supplement: Supplementary file 1 [file Data_Sheet_1.zip › supplementary materials/Forward/forest plot/ebi-a-GCST90027535.finngen_R12_E4_LACTONAS.pdf]

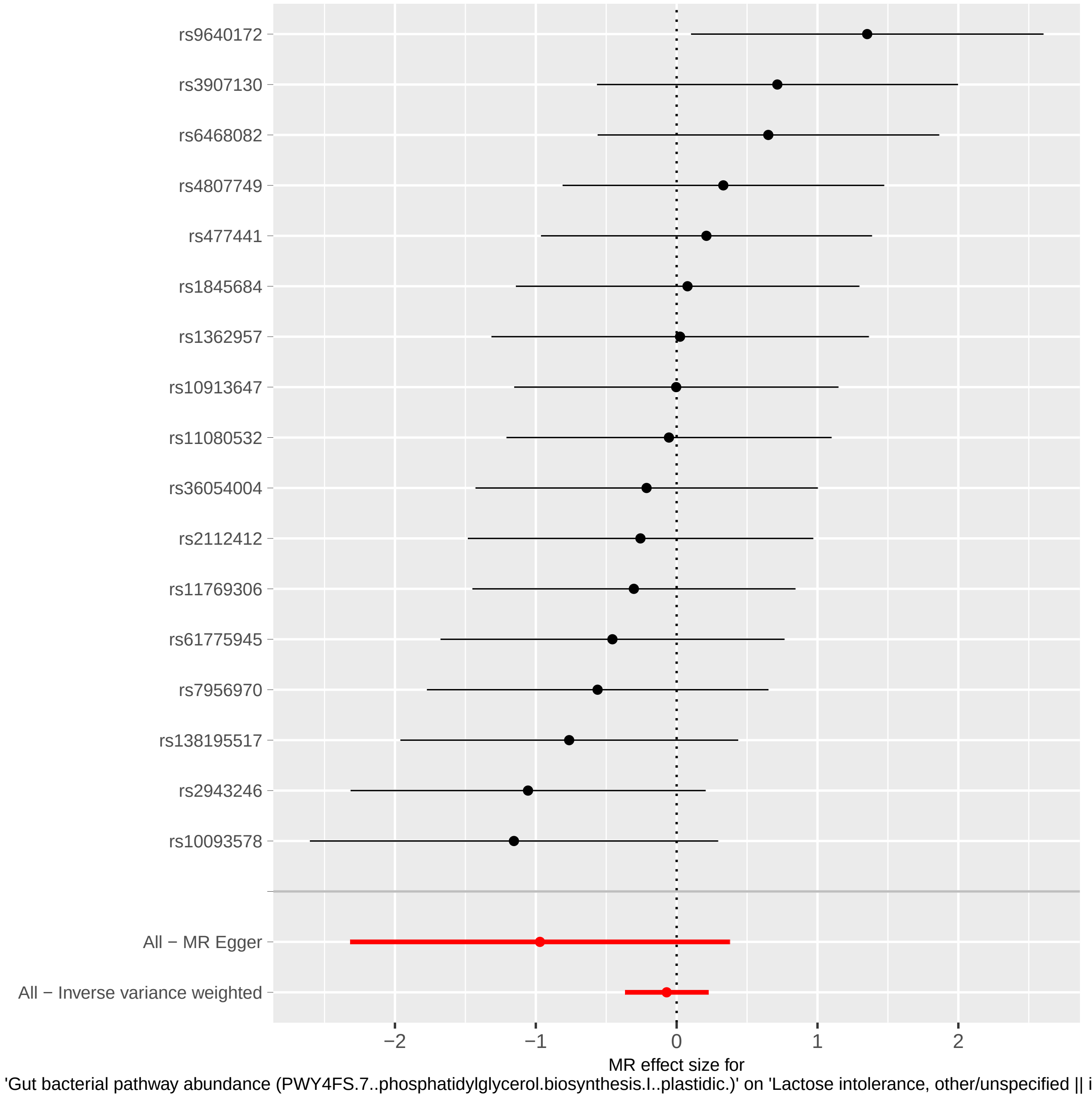
rs9640172
rs3907130
rs6468082
rs4807749
rs477441
rs1845684
rs1362957
rs10913647
rs11080532
rs36054004
rs2112412
rs11769306
rs61775945
rs7956970
rs138195517
rs2943246
rs10093578
All − MR Egger
All − Inverse variance weighted
−2
−1
0
1
2
MR effect size for
'Gut bacterial pathway abundance (PWY4FS.7..phosphatidylglycerol.biosynthesis.I..plastidic.)' on 'Lactose intolerance, other/unspecified || i
