# Supplementary material for: Causal interplay between lactose intolerance and gut microbiota: a combined bidirectional Mendelian randomization and in vivo validation study
Source: Front Nutr. 2026 Jun 1;13:1803337. doi: 10.3389/fnut.2026.1803337 (PMC13265576; doi:10.3389/fnut.2026.1803337)
Supplement: Supplementary file 1 [file Data_Sheet_1.zip › supplementary materials/Forward/forest plot/ebi-a-GCST90027538.finngen_R12_E4_LACTONAS.pdf]

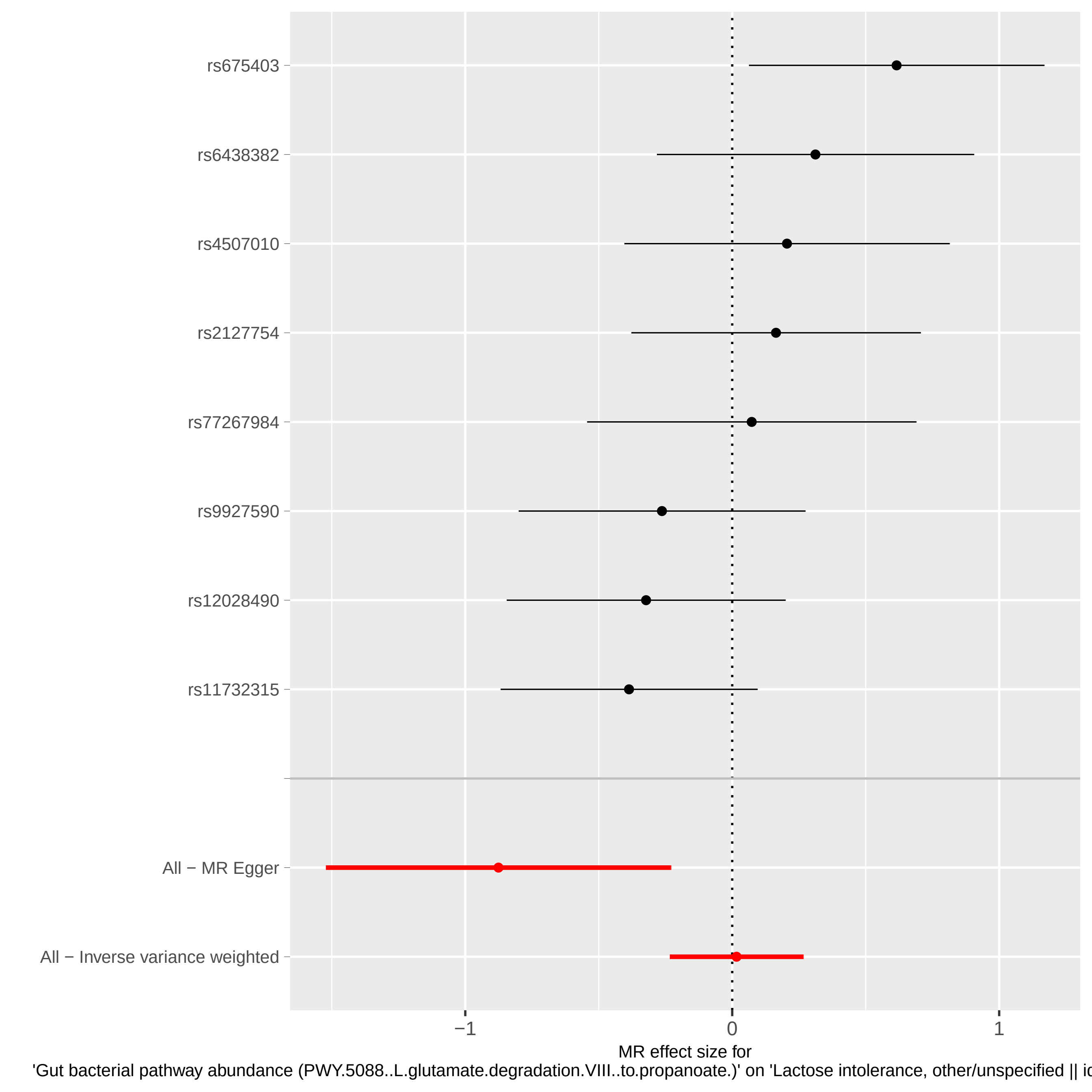
rs675403
rs6438382
rs4507010
rs2127754
rs77267984
rs9927590
rs12028490
rs11732315
All − MR Egger
All − Inverse variance weighted
−1
0
1
MR effect size for
'Gut bacterial pathway abundance (PWY.5088..L.glutamate.degradation.VIII..to.propanoate.)' on 'Lactose intolerance, other/unspecified || id
