# Supplementary material for: Causal interplay between lactose intolerance and gut microbiota: a combined bidirectional Mendelian randomization and in vivo validation study
Source: Front Nutr. 2026 Jun 1;13:1803337. doi: 10.3389/fnut.2026.1803337 (PMC13265576; doi:10.3389/fnut.2026.1803337)
Supplement: Supplementary file 1 [file Data_Sheet_1.zip › supplementary materials/Forward/forest plot/ebi-a-GCST90027558.finngen_R12_E4_LACTONAS.pdf]

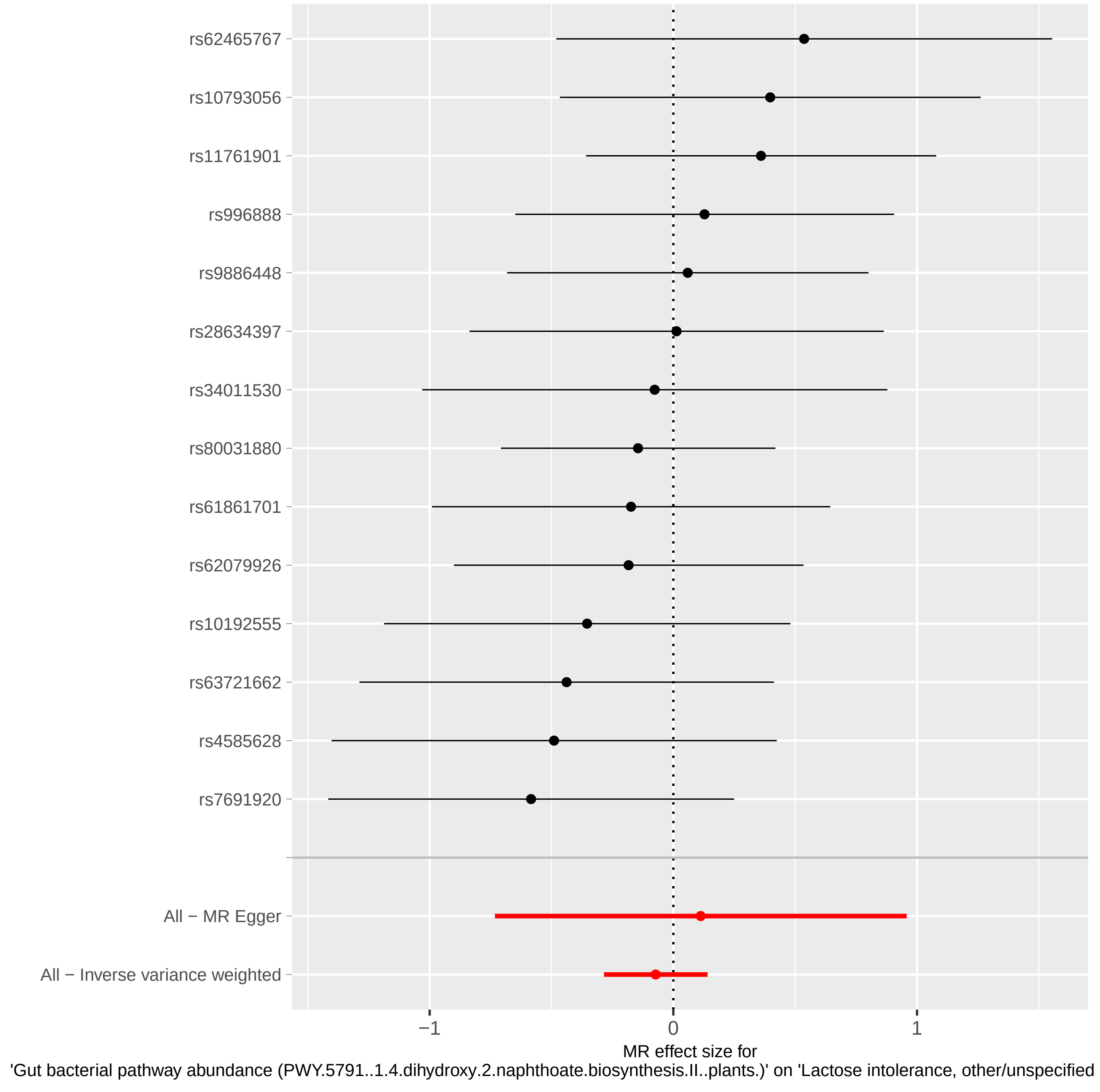

rs62465767
rs10793056
rs11761901
rs996888
rs9886448
rs28634397
rs34011530
rs80031880
rs61861701
rs62079926
rs10192555
rs63721662
rs4585628
rs7691920
All − MR Egger
All − Inverse variance weighted
−1
0
1
MR effect size for
'Gut bacterial pathway abundance (PWY.5791..1.4.dihydroxy.2.naphthoate.biosynthesis.II..plants.)' on 'Lactose intolerance, other/unspecified
