# Supplementary material for: Causal interplay between lactose intolerance and gut microbiota: a combined bidirectional Mendelian randomization and in vivo validation study
Source: Front Nutr. 2026 Jun 1;13:1803337. doi: 10.3389/fnut.2026.1803337 (PMC13265576; doi:10.3389/fnut.2026.1803337)
Supplement: Supplementary file 1 [file Data_Sheet_1.zip › supplementary materials/Forward/forest plot/ebi-a-GCST90027560.finngen_R12_E4_LACTONAS.pdf]

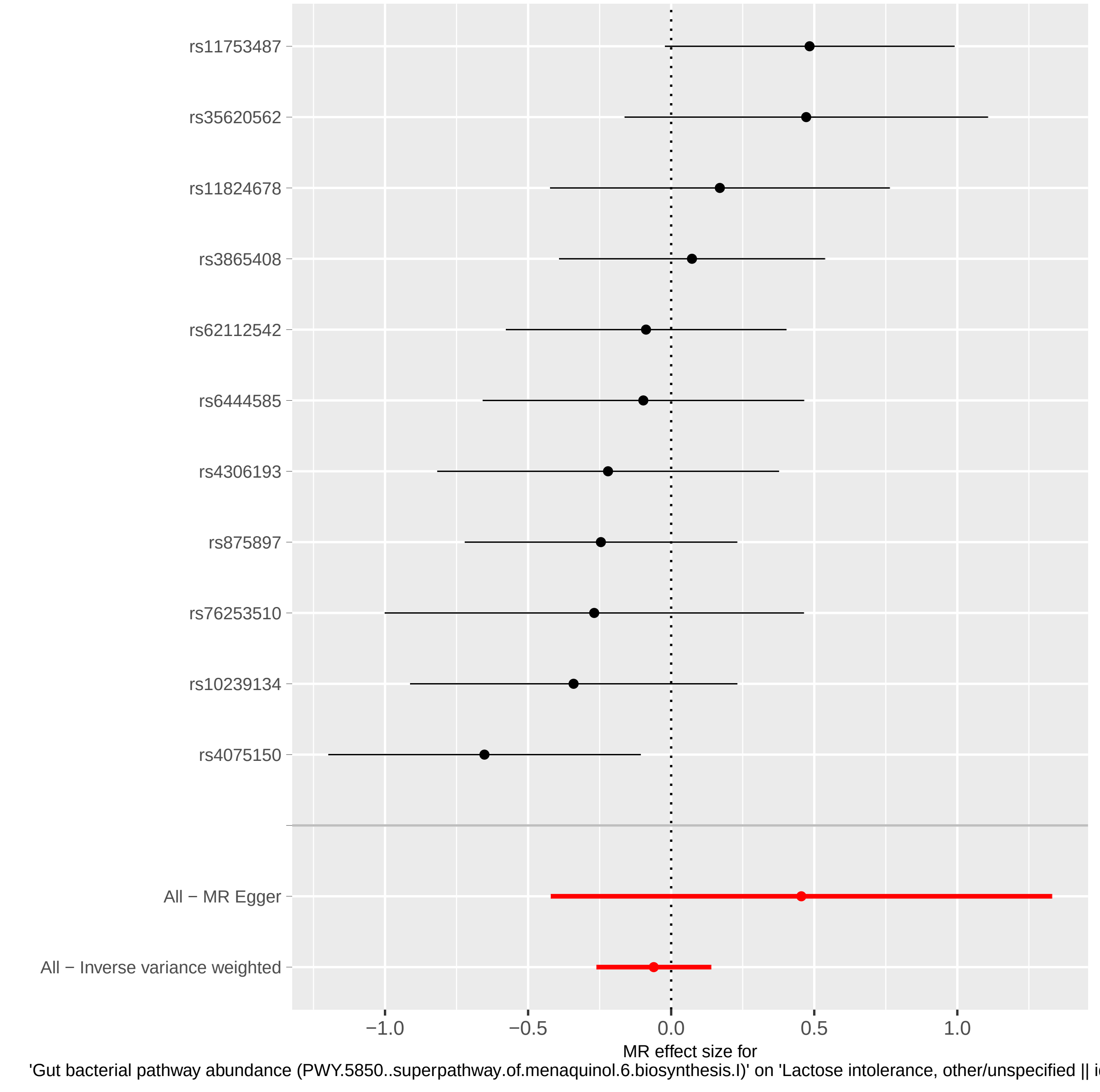
rs11753487
rs35620562
rs11824678
rs3865408
rs62112542
rs6444585
rs4306193
rs875897
rs76253510
rs10239134
rs4075150
All − MR Egger
All − Inverse variance weighted
−1.0
−0.5
0.0
0.5
1.0
MR effect size for
'Gut bacterial pathway abundance (PWY.5850..superpathway.of.menaquinol.6.biosynthesis.I)' on 'Lactose intolerance, other/unspecified || i
