# Supplementary material for: Causal interplay between lactose intolerance and gut microbiota: a combined bidirectional Mendelian randomization and in vivo validation study
Source: Front Nutr. 2026 Jun 1;13:1803337. doi: 10.3389/fnut.2026.1803337 (PMC13265576; doi:10.3389/fnut.2026.1803337)
Supplement: Supplementary file 1 [file Data_Sheet_1.zip › supplementary materials/Forward/forest plot/ebi-a-GCST90027562.finngen_R12_E4_LACTONAS.pdf]

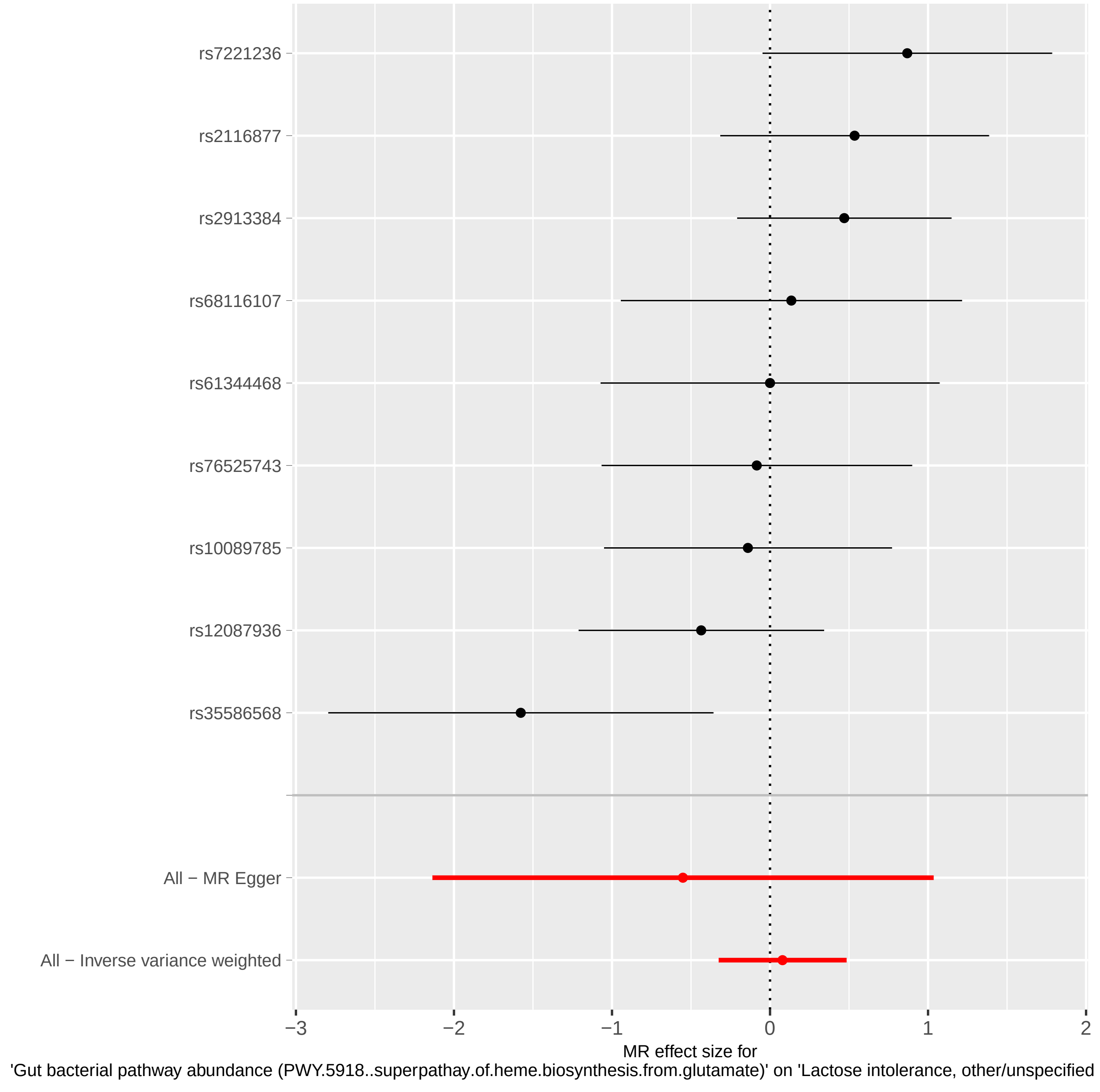

rs7221236
rs2116877
rs2913384
rs68116107
rs61344468
rs76525743
rs10089785
rs12087936
rs35586568
All − MR Egger
All − Inverse variance weighted
−3
−2
−1
0
1
2
MR effect size for
'Gut bacterial pathway abundance (PWY.5918..superpathay.of.heme.biosynthesis.from.glutamate)' on 'Lactose intolerance, other/unspecified
