# Supplementary material for: Causal interplay between lactose intolerance and gut microbiota: a combined bidirectional Mendelian randomization and in vivo validation study
Source: Front Nutr. 2026 Jun 1;13:1803337. doi: 10.3389/fnut.2026.1803337 (PMC13265576; doi:10.3389/fnut.2026.1803337)
Supplement: Supplementary file 1 [file Data_Sheet_1.zip › supplementary materials/Forward/forest plot/ebi-a-GCST90027571.finngen_R12_E4_LACTONAS.pdf]

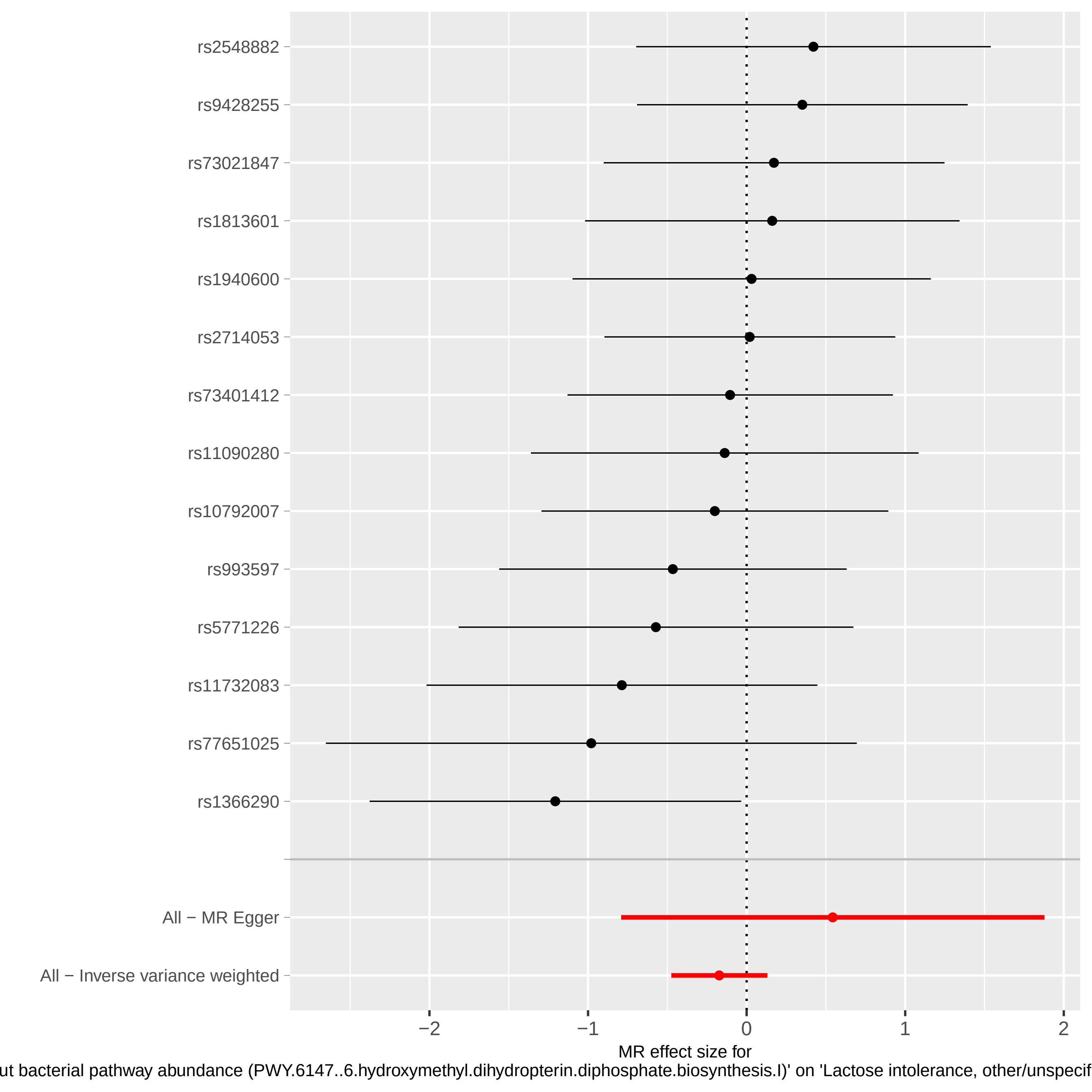

rs2548882
rs9428255
rs73021847
rs1813601
rs1940600
rs2714053
rs73401412
rs11090280
rs10792007
rs993597
rs5771226
rs11732083
rs77651025
rs1366290
All − MR Egger
All − Inverse variance weighted
−2
−1
0
1
2
MR effect size for
ut bacterial pathway abundance (PWY.6147..6.hydroxymethyl.dihydropterin.diphosphate.biosynthesis.I)' on 'Lactose intolerance, other/unspecif
