# Supplementary material for: Causal interplay between lactose intolerance and gut microbiota: a combined bidirectional Mendelian randomization and in vivo validation study
Source: Front Nutr. 2026 Jun 1;13:1803337. doi: 10.3389/fnut.2026.1803337 (PMC13265576; doi:10.3389/fnut.2026.1803337)
Supplement: Supplementary file 1 [file Data_Sheet_1.zip › supplementary materials/Forward/forest plot/ebi-a-GCST90027574.finngen_R12_E4_LACTONAS.pdf]

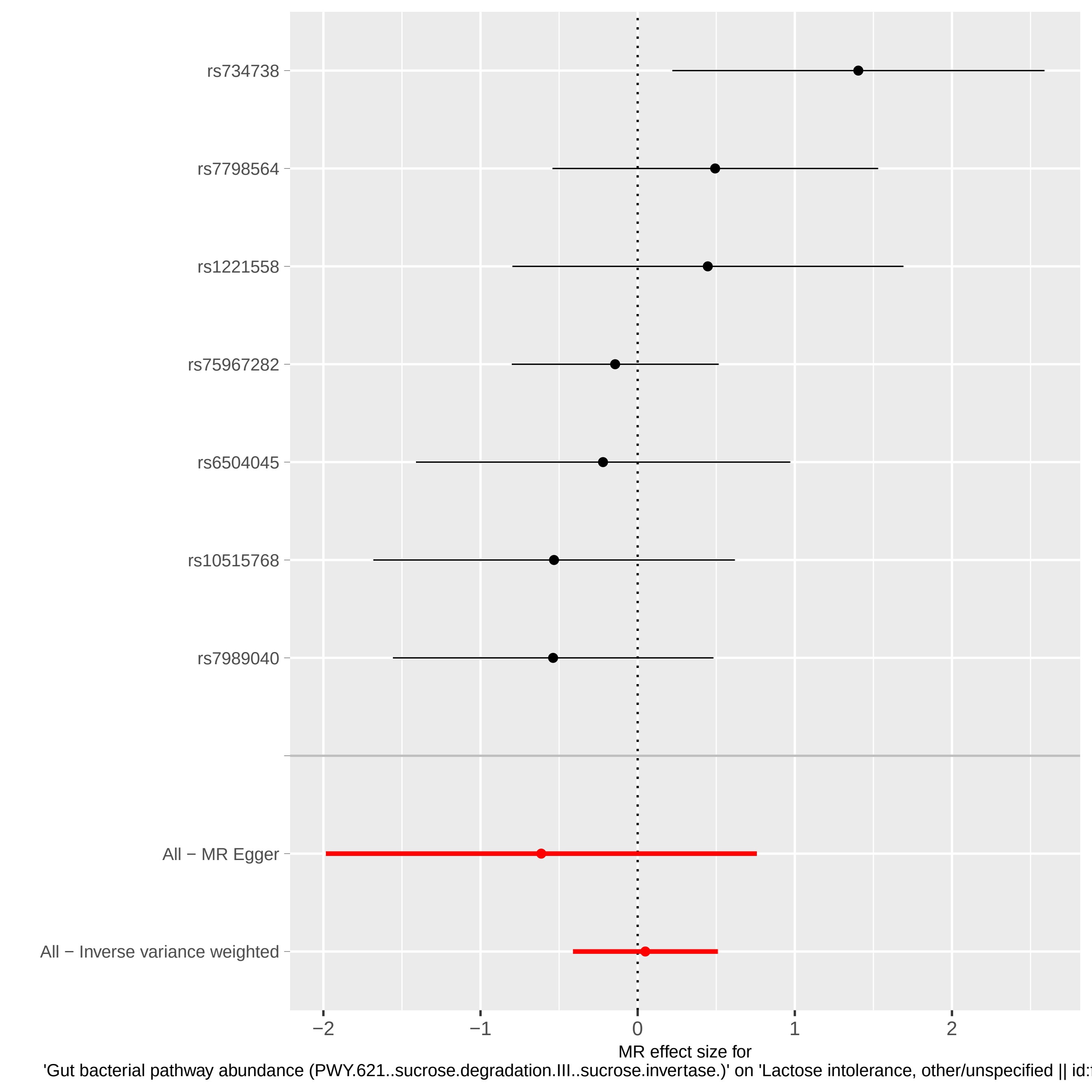

rs734738
rs7798564
rs1221558
rs75967282
rs6504045
rs10515768
rs7989040
All − MR Egger
All − Inverse variance weighted
−2
−1
0
1
2
MR effect size for
'Gut bacterial pathway abundance (PWY.621..sucrose.degradation.III..sucrose.invertase.)' on 'Lactose intolerance, other/unspecified || id:
