# Supplementary material for: Causal interplay between lactose intolerance and gut microbiota: a combined bidirectional Mendelian randomization and in vivo validation study
Source: Front Nutr. 2026 Jun 1;13:1803337. doi: 10.3389/fnut.2026.1803337 (PMC13265576; doi:10.3389/fnut.2026.1803337)
Supplement: Supplementary file 1 [file Data_Sheet_1.zip › supplementary materials/Forward/forest plot/ebi-a-GCST90027578.finngen_R12_E4_LACTONAS.pdf]

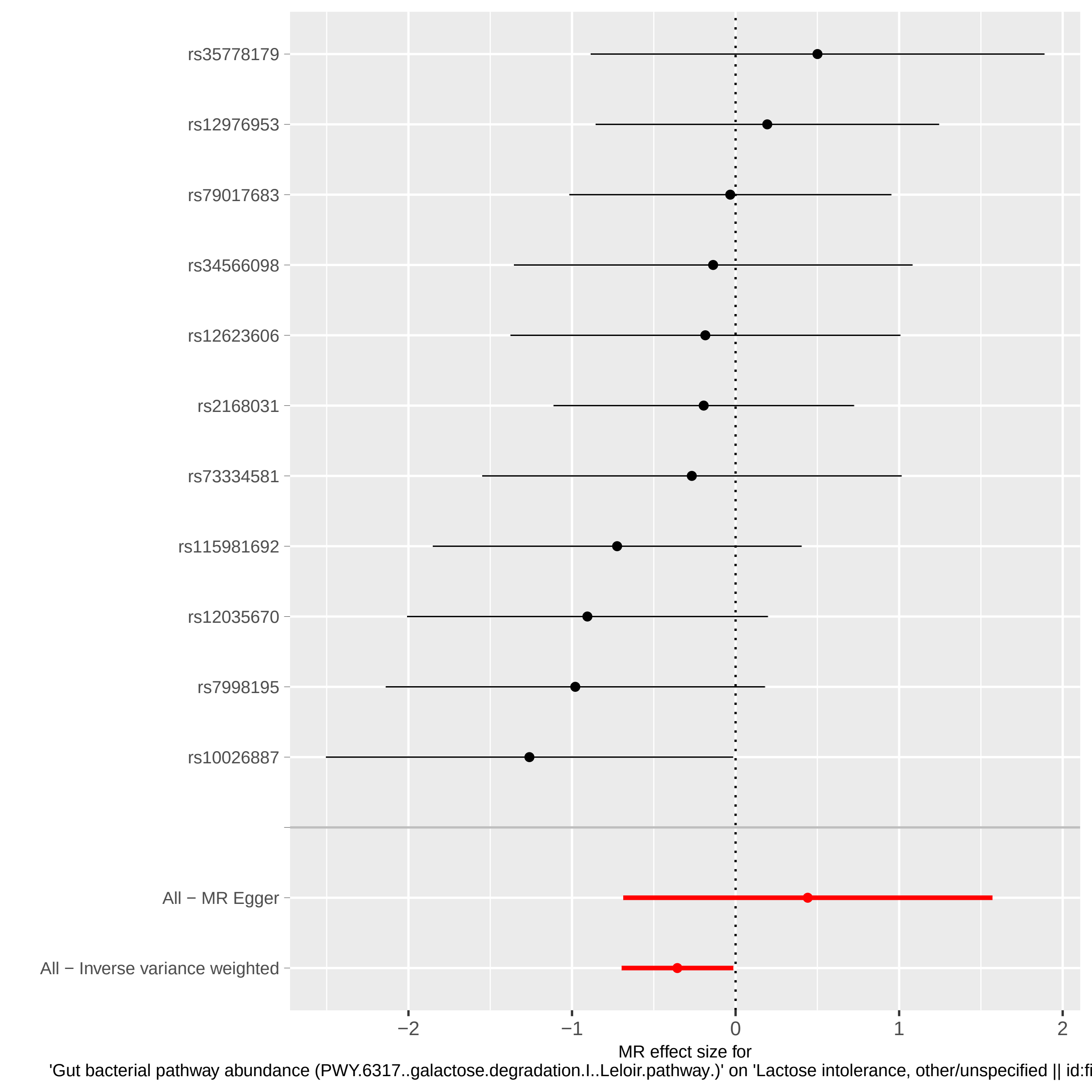
rs35778179
rs12976953
rs79017683
rs34566098
rs12623606
rs2168031
rs73334581
rs115981692
rs12035670
rs7998195
rs10026887
All − MR Egger
All − Inverse variance weighted
−2
−1
0
1
2
MR effect size for
'Gut bacterial pathway abundance (PWY.6317..galactose.degradation.I..Leloir.pathway.)' on 'Lactose intolerance, other/unspecified || id:fi
