# Supplementary material for: Causal interplay between lactose intolerance and gut microbiota: a combined bidirectional Mendelian randomization and in vivo validation study
Source: Front Nutr. 2026 Jun 1;13:1803337. doi: 10.3389/fnut.2026.1803337 (PMC13265576; doi:10.3389/fnut.2026.1803337)
Supplement: Supplementary file 1 [file Data_Sheet_1.zip › supplementary materials/Forward/forest plot/ebi-a-GCST90027583.finngen_R12_E4_LACTONAS.pdf]

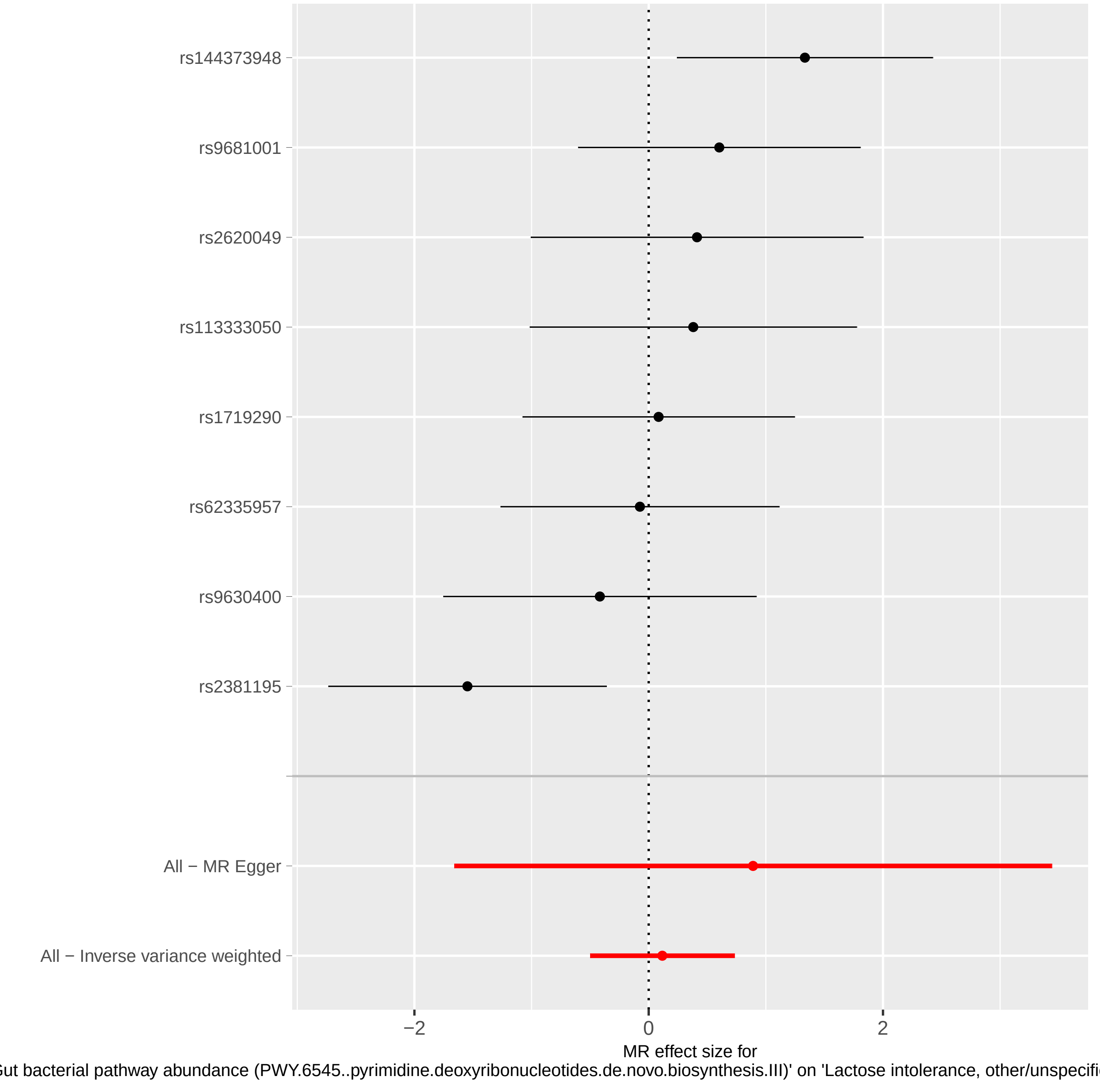
rs144373948
rs9681001
rs2620049
rs113333050
rs1719290
rs62335957
rs9630400
rs2381195
All − MR Egger
All − Inverse variance weighted
−2
0
2
MR effect size for
Gut bacterial pathway abundance (PWY.6545..pyrimidine.deoxyribonucleotides.de.novo.biosynthesis.III)' on 'Lactose intolerance, other/unspecifi
