# Supplementary material for: Causal interplay between lactose intolerance and gut microbiota: a combined bidirectional Mendelian randomization and in vivo validation study
Source: Front Nutr. 2026 Jun 1;13:1803337. doi: 10.3389/fnut.2026.1803337 (PMC13265576; doi:10.3389/fnut.2026.1803337)
Supplement: Supplementary file 1 [file Data_Sheet_1.zip › supplementary materials/Forward/forest plot/ebi-a-GCST90027589.finngen_R12_E4_LACTONAS.pdf]

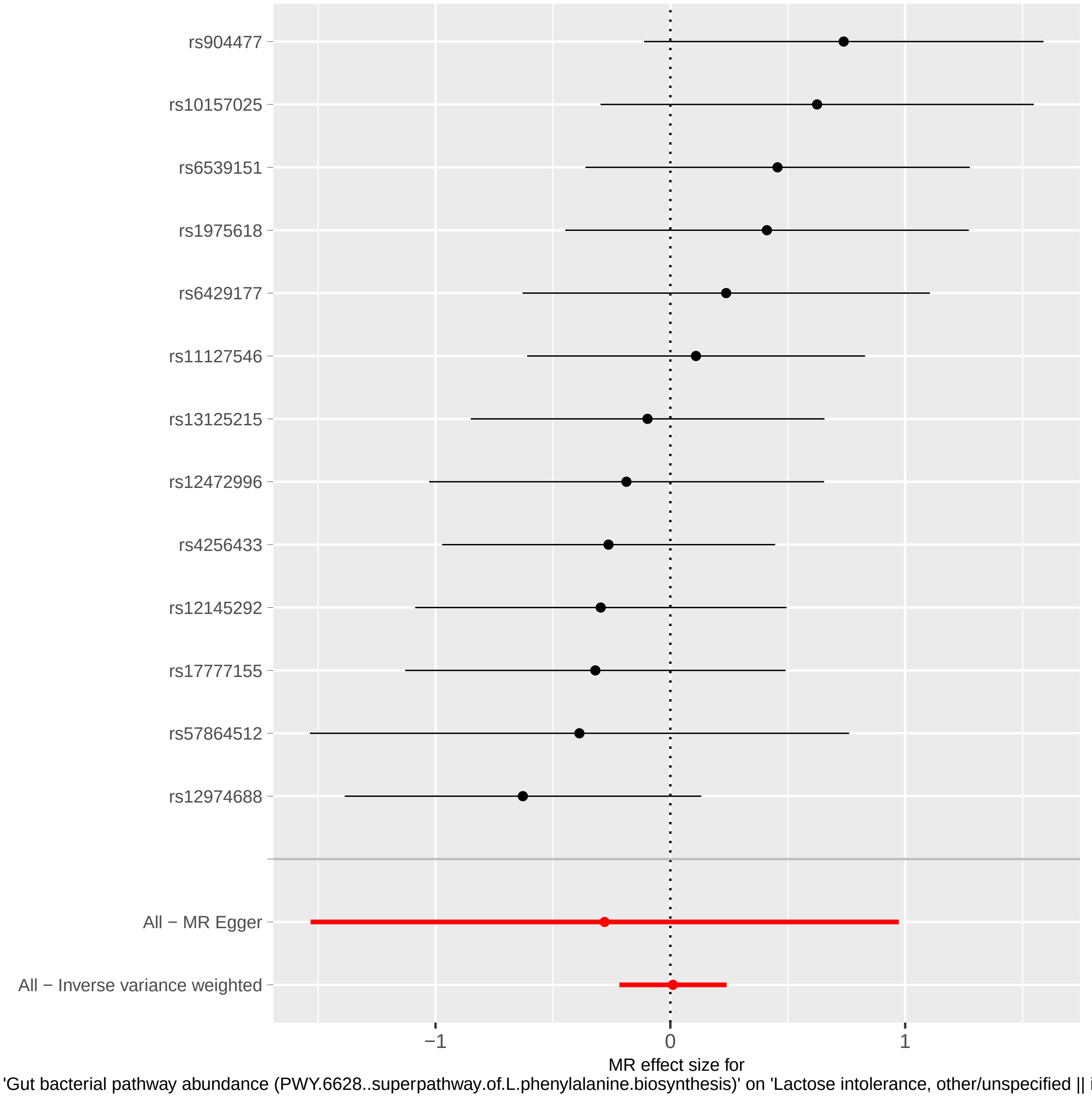
rs904477
rs10157025
rs6539151
rs1975618
rs6429177
rs11127546
rs13125215
rs12472996
rs4256433
rs12145292
rs17777155
rs57864512
rs12974688
All − MR Egger
All − Inverse variance weighted
−1
0
1
MR effect size for
'Gut bacterial pathway abundance (PWY.6628..superpathway.of.L.phenylalanine.biosynthesis)' on 'Lactose intolerance, other/unspecified || i
