# Supplementary material for: Causal interplay between lactose intolerance and gut microbiota: a combined bidirectional Mendelian randomization and in vivo validation study
Source: Front Nutr. 2026 Jun 1;13:1803337. doi: 10.3389/fnut.2026.1803337 (PMC13265576; doi:10.3389/fnut.2026.1803337)
Supplement: Supplementary file 1 [file Data_Sheet_1.zip › supplementary materials/Forward/forest plot/ebi-a-GCST90027590.finngen_R12_E4_LACTONAS.pdf]

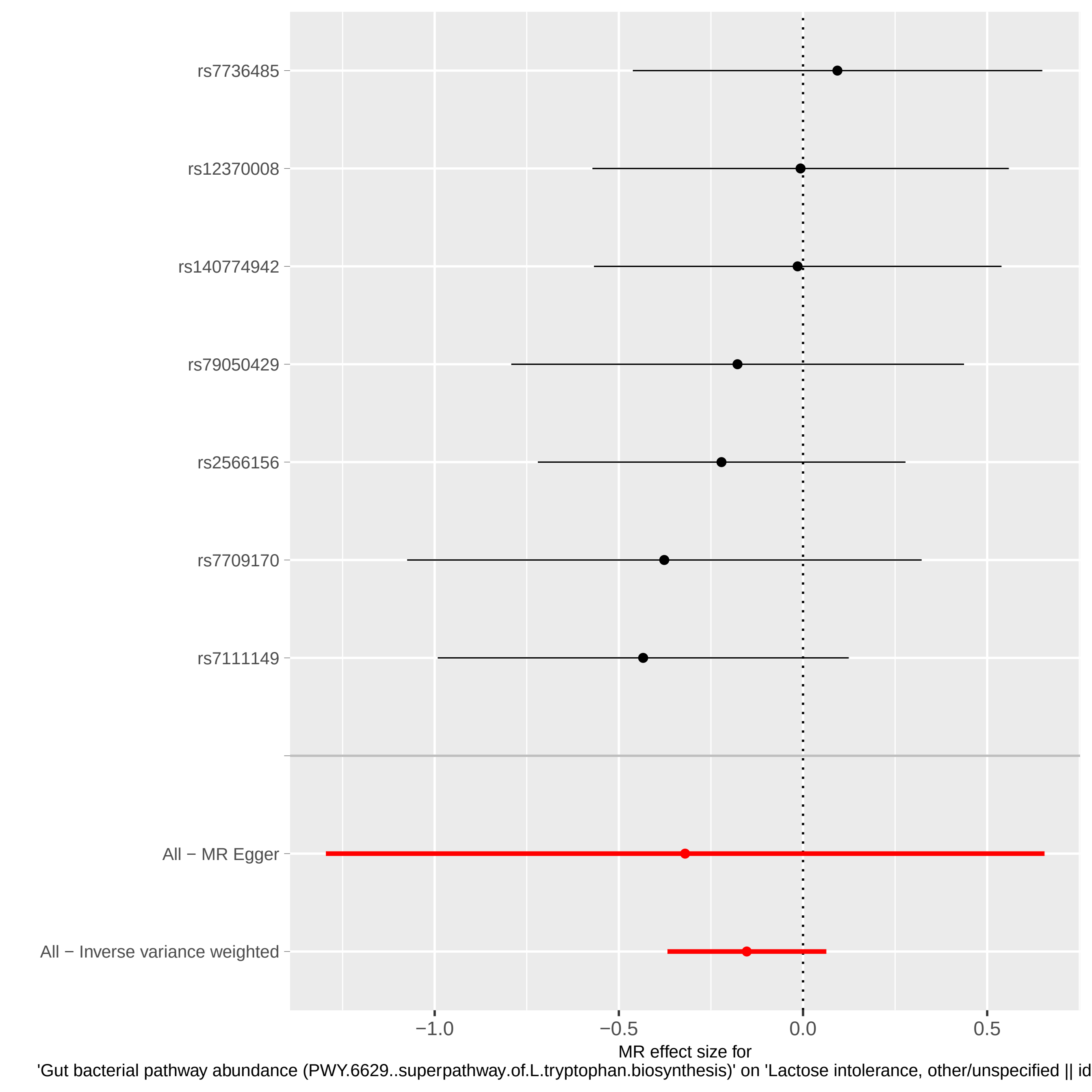

rs7736485
rs12370008
rs140774942
rs79050429
rs2566156
rs7709170
rs7111149
All − MR Egger
All − Inverse variance weighted
−1.0
−0.5
0.0
0.5
MR effect size for
'Gut bacterial pathway abundance (PWY.6629..superpathway.of.L.tryptophan.biosynthesis)' on 'Lactose intolerance, other/unspecified || id
