# Supplementary material for: Causal interplay between lactose intolerance and gut microbiota: a combined bidirectional Mendelian randomization and in vivo validation study
Source: Front Nutr. 2026 Jun 1;13:1803337. doi: 10.3389/fnut.2026.1803337 (PMC13265576; doi:10.3389/fnut.2026.1803337)
Supplement: Supplementary file 1 [file Data_Sheet_1.zip › supplementary materials/Forward/forest plot/ebi-a-GCST90027603.finngen_R12_E4_LACTONAS.pdf]

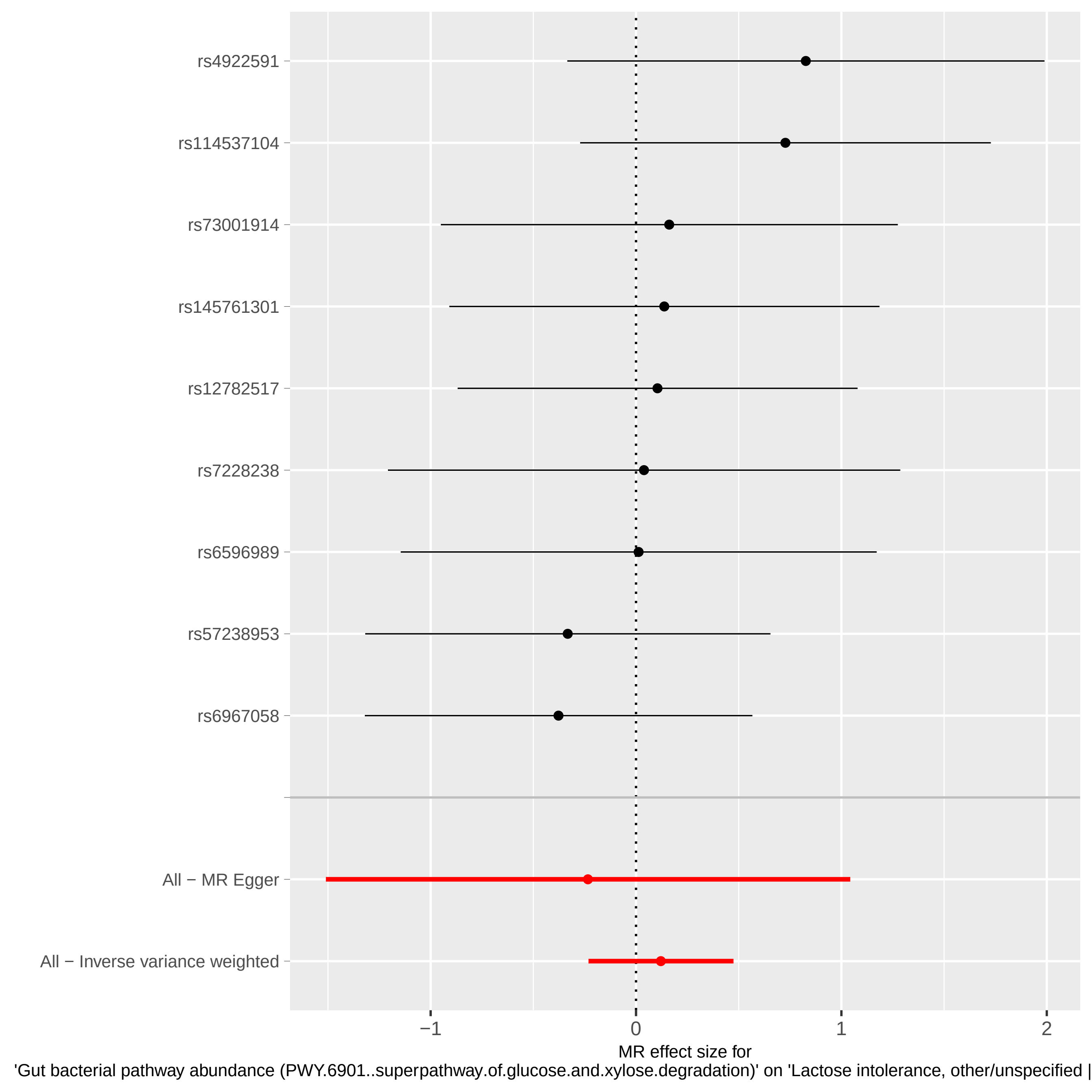
rs4922591
rs114537104
rs73001914
rs145761301
rs12782517
rs7228238
rs6596989
rs57238953
rs6967058
All − MR Egger
All − Inverse variance weighted
−1
0
1
2
MR effect size for
'Gut bacterial pathway abundance (PWY.6901..superpathway.of.glucose.and.xylose.degradation)' on 'Lactose intolerance, other/unspecified |
