# Supplementary material for: Causal interplay between lactose intolerance and gut microbiota: a combined bidirectional Mendelian randomization and in vivo validation study
Source: Front Nutr. 2026 Jun 1;13:1803337. doi: 10.3389/fnut.2026.1803337 (PMC13265576; doi:10.3389/fnut.2026.1803337)
Supplement: Supplementary file 1 [file Data_Sheet_1.zip › supplementary materials/Forward/forest plot/ebi-a-GCST90027609.finngen_R12_E4_LACTONAS.pdf]

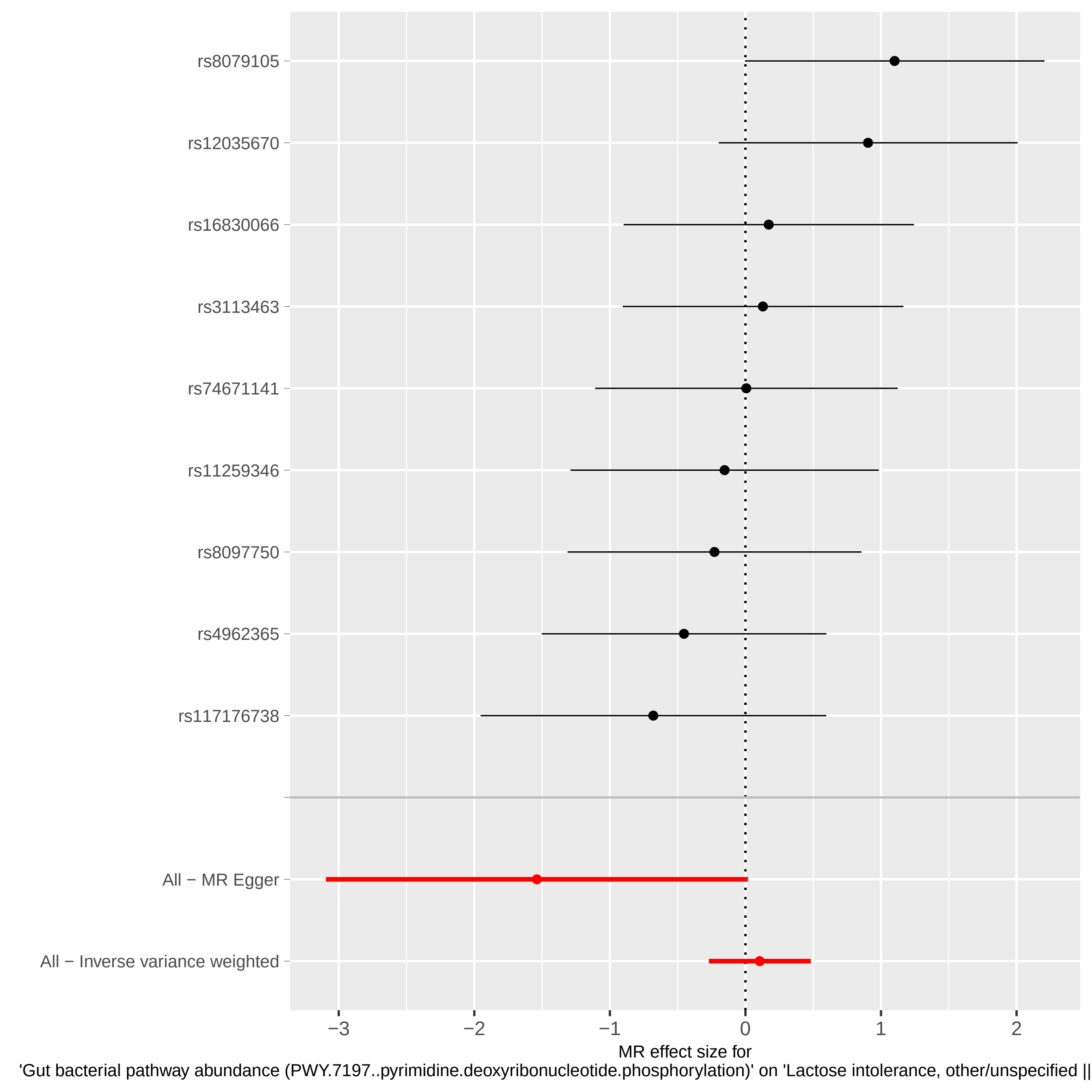
rs8079105
rs12035670
rs16830066
rs3113463
rs74671141
rs11259346
rs8097750
rs4962365
rs117176738
All − MR Egger
All − Inverse variance weighted
−3
−2
−1
0
1
2
MR effect size for
'Gut bacterial pathway abundance (PWY.7197..pyrimidine.deoxyribonucleotide.phosphorylation)' on 'Lactose intolerance, other/unspecified ||
