# Supplementary material for: Causal interplay between lactose intolerance and gut microbiota: a combined bidirectional Mendelian randomization and in vivo validation study
Source: Front Nutr. 2026 Jun 1;13:1803337. doi: 10.3389/fnut.2026.1803337 (PMC13265576; doi:10.3389/fnut.2026.1803337)
Supplement: Supplementary file 1 [file Data_Sheet_1.zip › supplementary materials/Forward/forest plot/ebi-a-GCST90027611.finngen_R12_E4_LACTONAS.pdf]

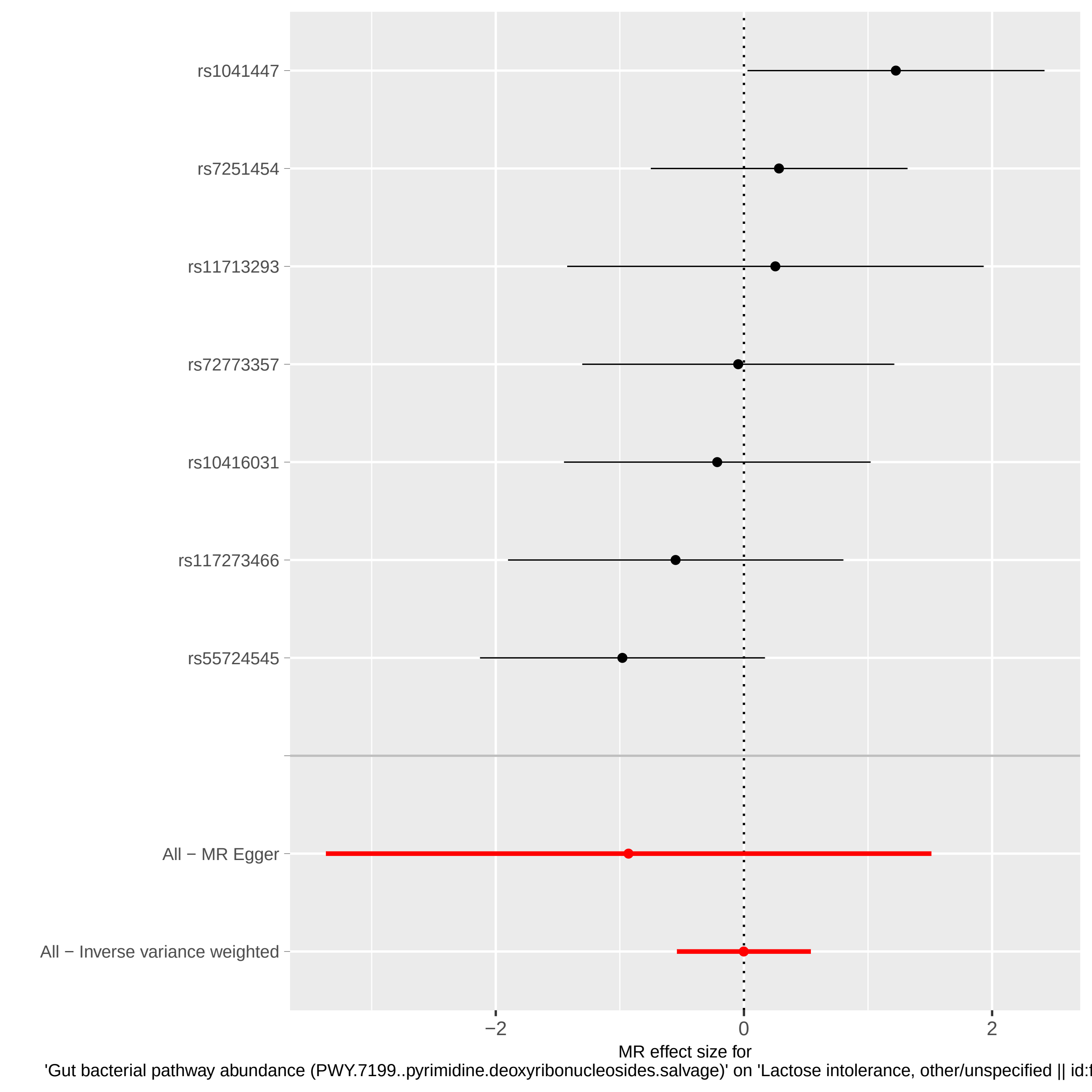
rs1041447
rs7251454
rs11713293
rs72773357
rs10416031
rs117273466
rs55724545
All − MR Egger
All − Inverse variance weighted
−2
0
2
MR effect size for
'Gut bacterial pathway abundance (PWY.7199..pyrimidine.deoxyribonucleosides.salvage)' on 'Lactose intolerance, other/unspecified || id:f
