# Supplementary material for: Causal interplay between lactose intolerance and gut microbiota: a combined bidirectional Mendelian randomization and in vivo validation study
Source: Front Nutr. 2026 Jun 1;13:1803337. doi: 10.3389/fnut.2026.1803337 (PMC13265576; doi:10.3389/fnut.2026.1803337)
Supplement: Supplementary file 1 [file Data_Sheet_1.zip › supplementary materials/Forward/forest plot/ebi-a-GCST90027612.finngen_R12_E4_LACTONAS.pdf]

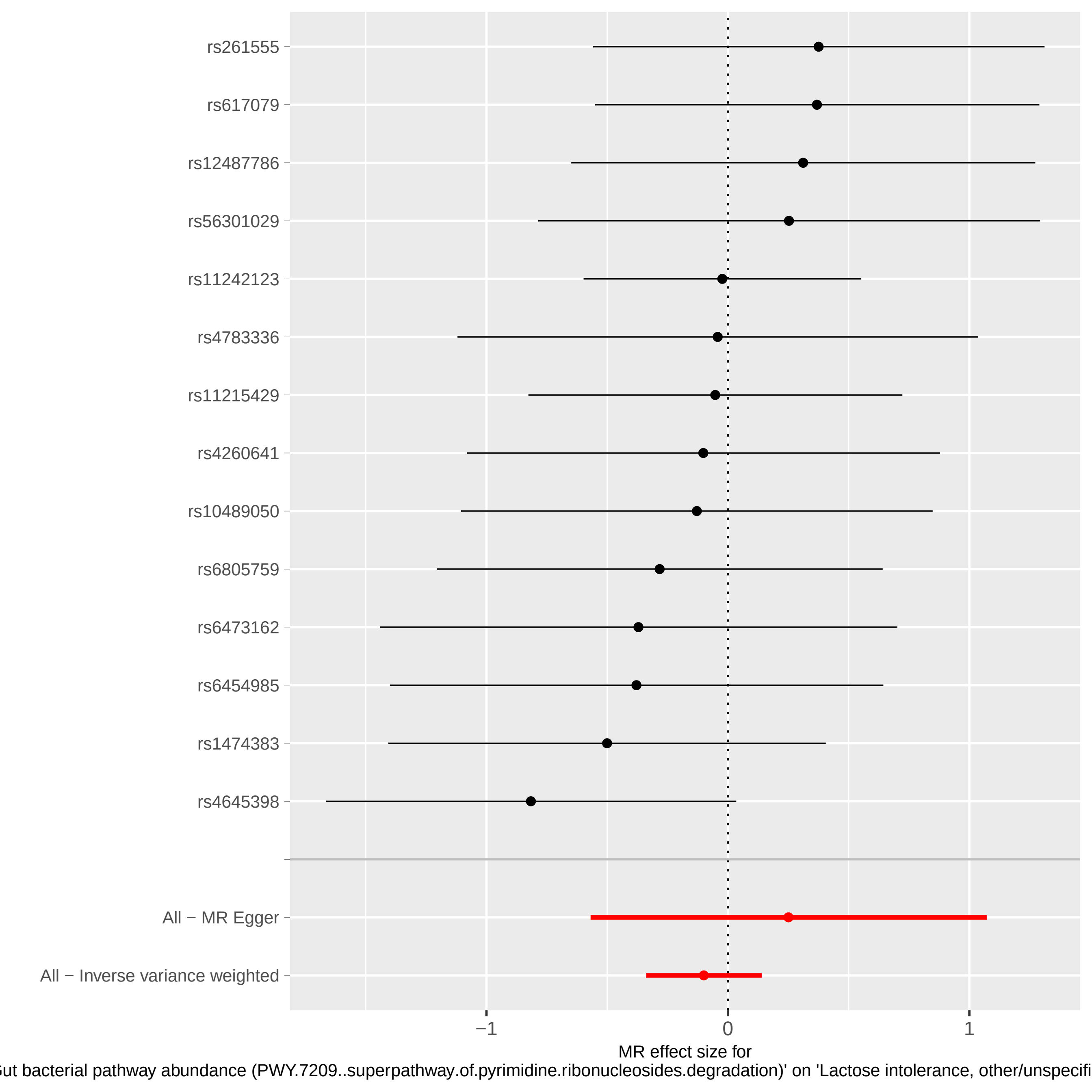

rs261555
rs617079
rs12487786
rs56301029
rs11242123
rs4783336
rs11215429
rs4260641
rs10489050
rs6805759
rs6473162
rs6454985
rs1474383
rs4645398
All − MR Egger
All − Inverse variance weighted
−1
0
1
MR effect size for
Gut bacterial pathway abundance (PWY.7209..superpathway.of.pyrimidine.ribonucleosides.degradation)' on 'Lactose intolerance, other/unspecifi
