# Supplementary material for: Causal interplay between lactose intolerance and gut microbiota: a combined bidirectional Mendelian randomization and in vivo validation study
Source: Front Nutr. 2026 Jun 1;13:1803337. doi: 10.3389/fnut.2026.1803337 (PMC13265576; doi:10.3389/fnut.2026.1803337)
Supplement: Supplementary file 1 [file Data_Sheet_1.zip › supplementary materials/Forward/forest plot/ebi-a-GCST90027614.finngen_R12_E4_LACTONAS.pdf]

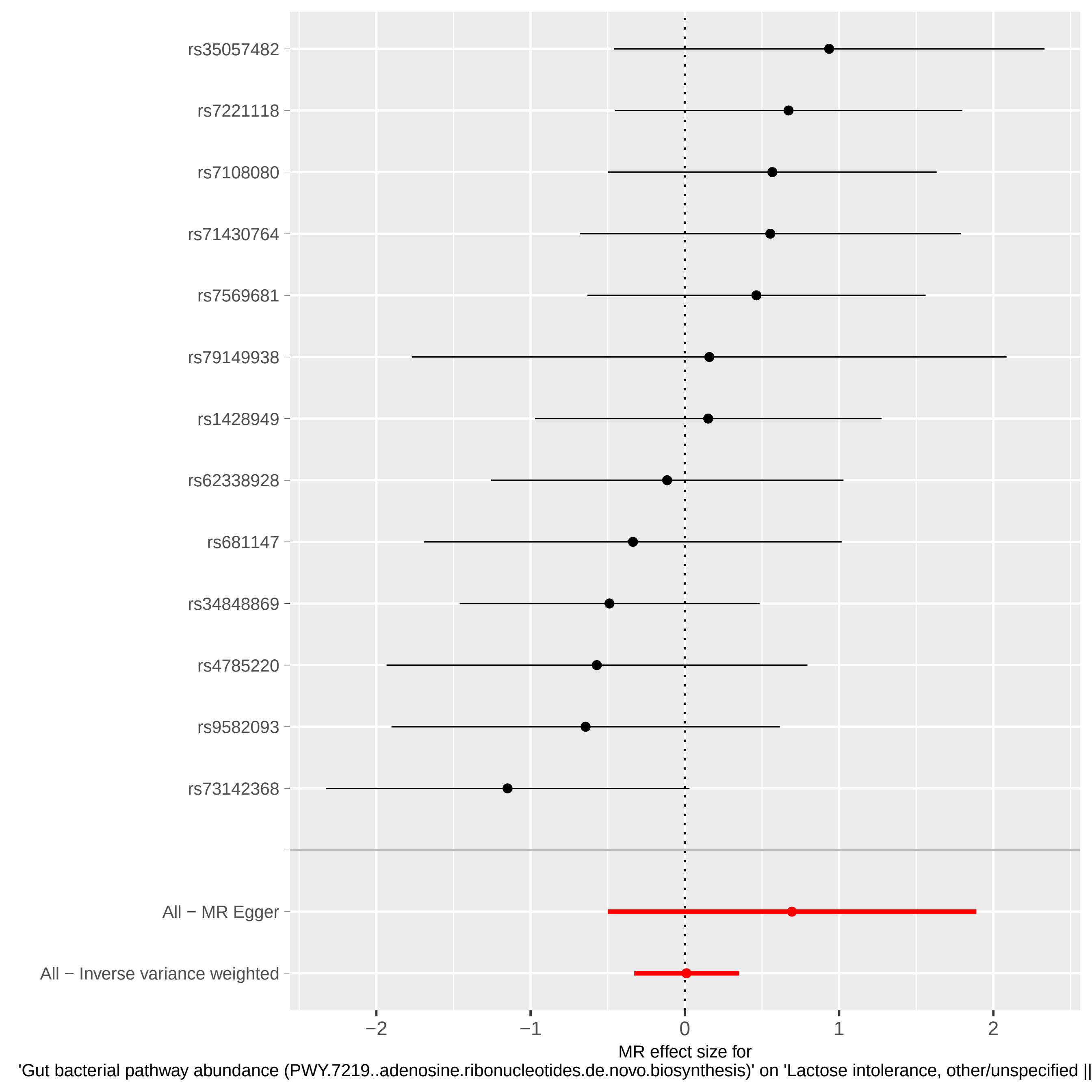
rs35057482
rs7221118
rs7108080
rs71430764
rs7569681
rs79149938
rs1428949
rs62338928
rs681147
rs34848869
rs4785220
rs9582093
rs73142368
All − MR Egger
All − Inverse variance weighted
−2
−1
0
1
2
MR effect size for
'Gut bacterial pathway abundance (PWY.7219..adenosine.ribonucleotides.de.novo.biosynthesis)' on 'Lactose intolerance, other/unspecified ||
