# Supplementary material for: Causal interplay between lactose intolerance and gut microbiota: a combined bidirectional Mendelian randomization and in vivo validation study
Source: Front Nutr. 2026 Jun 1;13:1803337. doi: 10.3389/fnut.2026.1803337 (PMC13265576; doi:10.3389/fnut.2026.1803337)
Supplement: Supplementary file 1 [file Data_Sheet_1.zip › supplementary materials/Forward/forest plot/ebi-a-GCST90027625.finngen_R12_E4_LACTONAS.pdf]

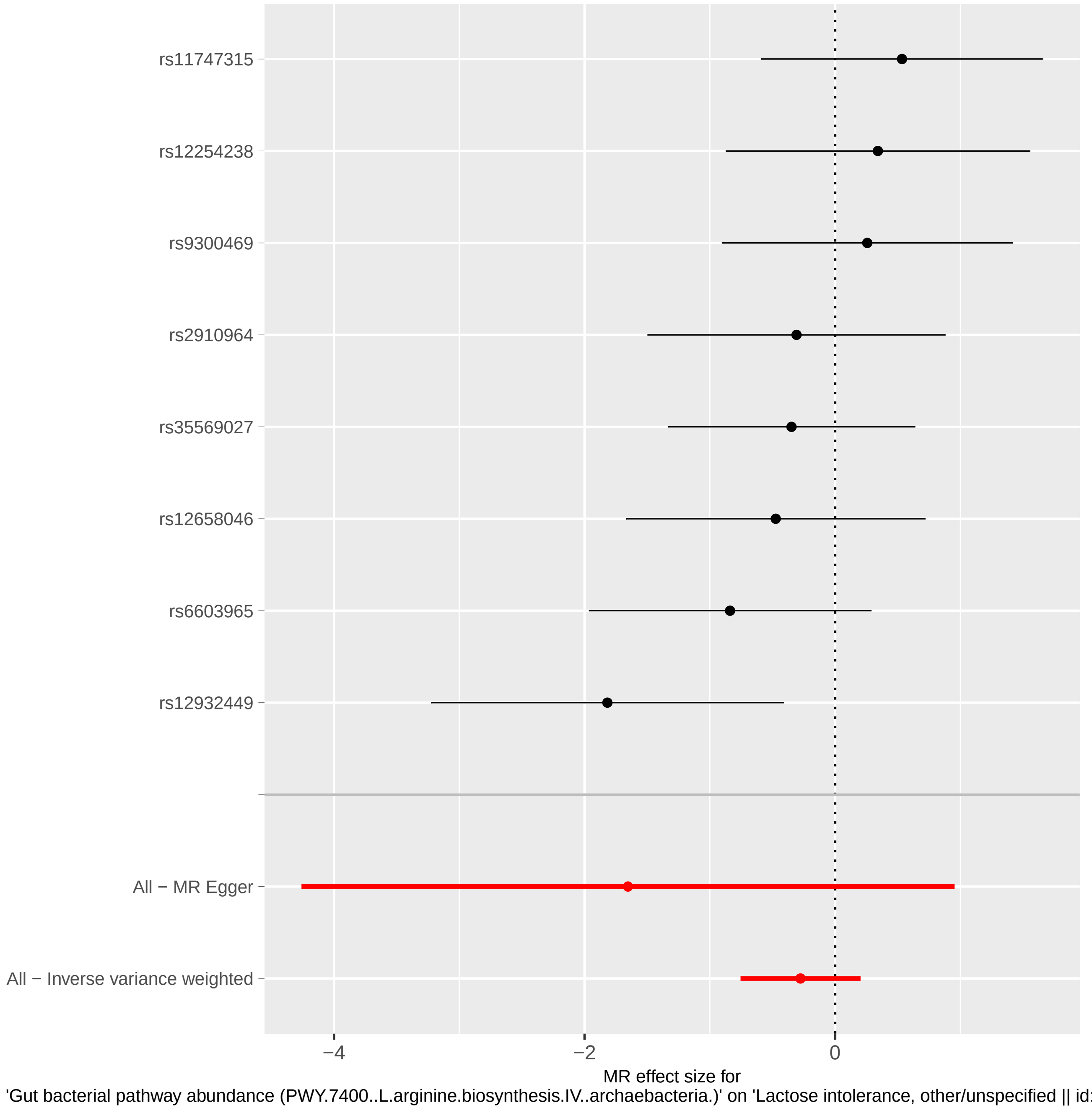

rs11747315
rs12254238
rs9300469
rs2910964
rs35569027
rs12658046
rs6603965
rs12932449
All − MR Egger
All − Inverse variance weighted
−4
−2
0
MR effect size for
'Gut bacterial pathway abundance (PWY.7400..L.arginine.biosynthesis.IV..archaebacteria.)' on 'Lactose intolerance, other/unspecified || id:
