# Supplementary figures and images for: Causal interplay between lactose intolerance and gut microbiota: a combined bidirectional Mendelian randomization and in vivo validation study (part 2 of 2)
Source: Front Nutr. 2026 Jun 1;13:1803337. doi: 10.3389/fnut.2026.1803337 (PMC13265576; doi:10.3389/fnut.2026.1803337)

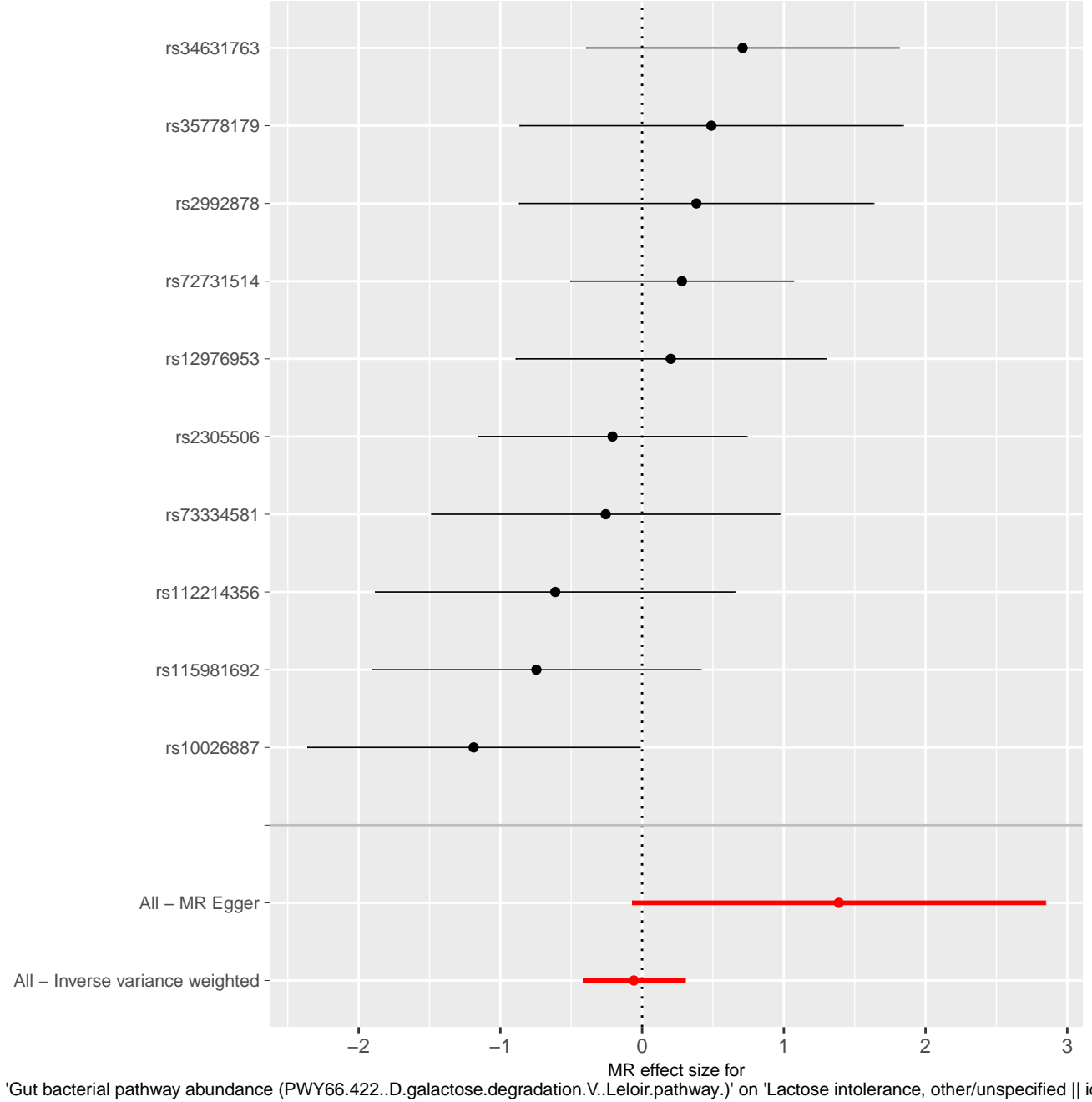

Supplement: Supplementary file 1 [file Data_Sheet_1.zip › supplementary materials/Forward/forest plot/ebi-a-GCST90027592.finngen_R12_E4_LACTONAS.pdf]

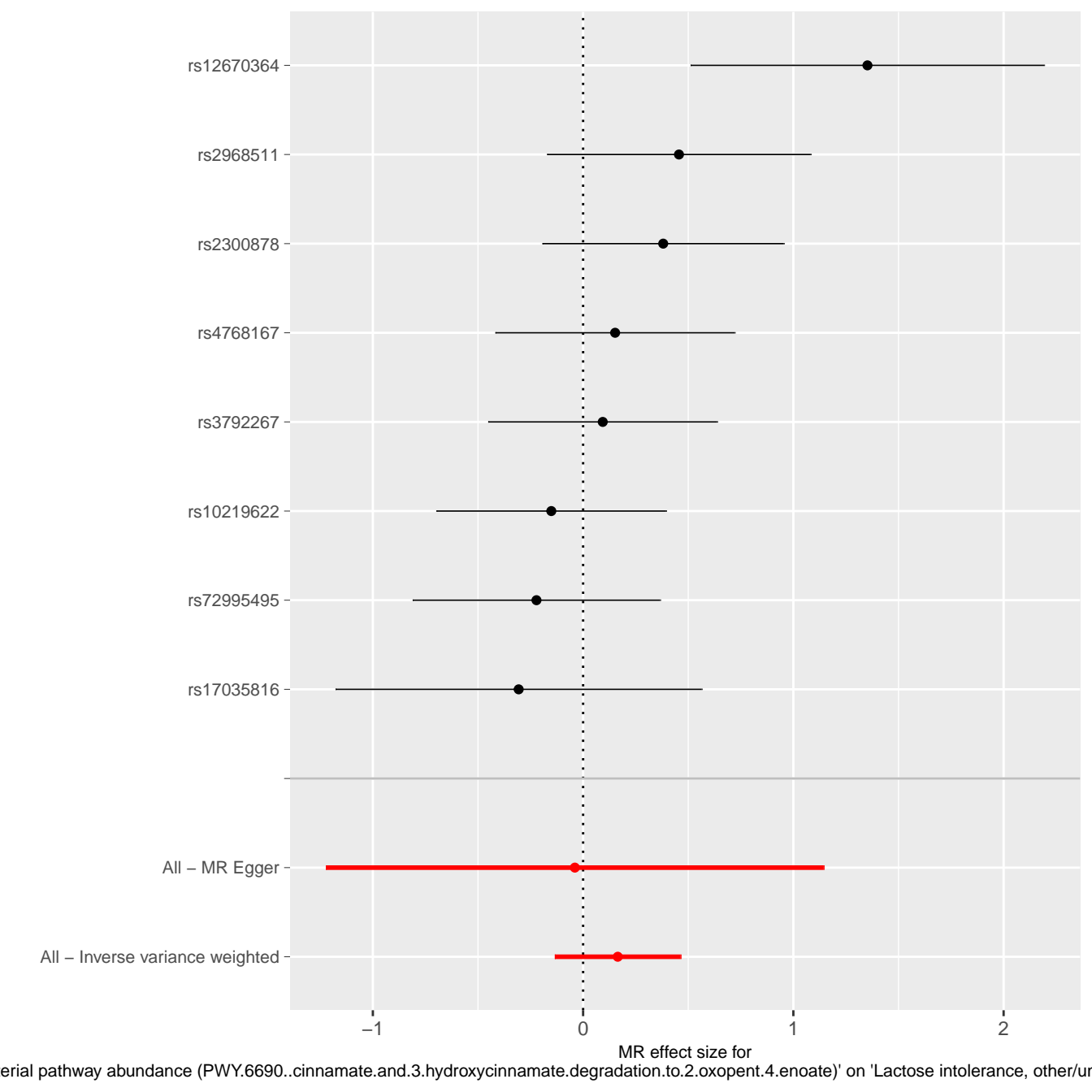

Supplement: Supplementary file 1 [file Data_Sheet_1.zip › supplementary materials/Forward/forest plot/ebi-a-GCST90027593.finngen_R12_E4_LACTONAS.pdf]

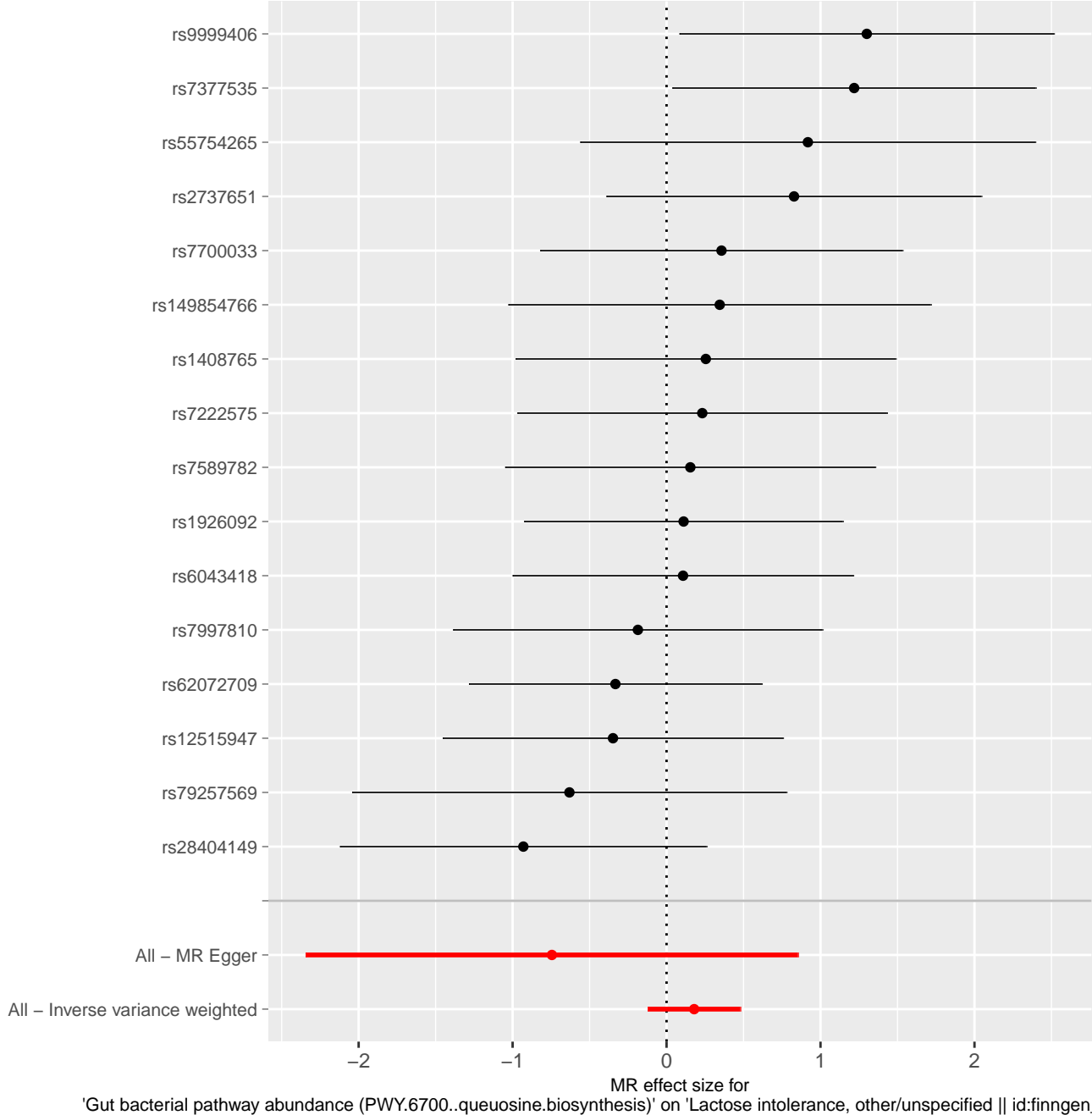

Supplement: Supplementary file 1 [file Data_Sheet_1.zip › supplementary materials/Forward/forest plot/ebi-a-GCST90027594.finngen_R12_E4_LACTONAS.pdf]

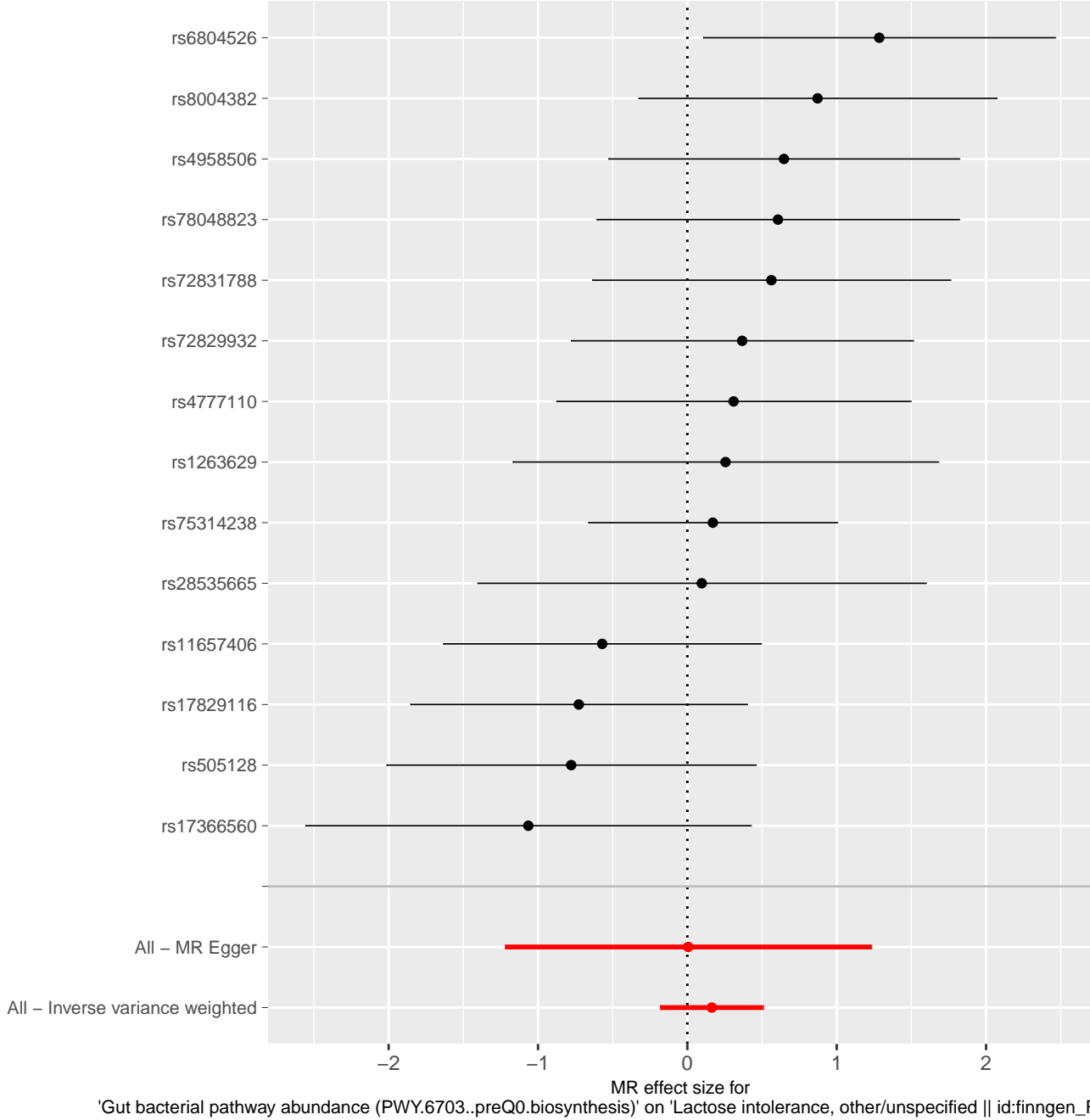

Supplement: Supplementary file 1 [file Data_Sheet_1.zip › supplementary materials/Forward/forest plot/ebi-a-GCST90027595.finngen_R12_E4_LACTONAS.pdf]

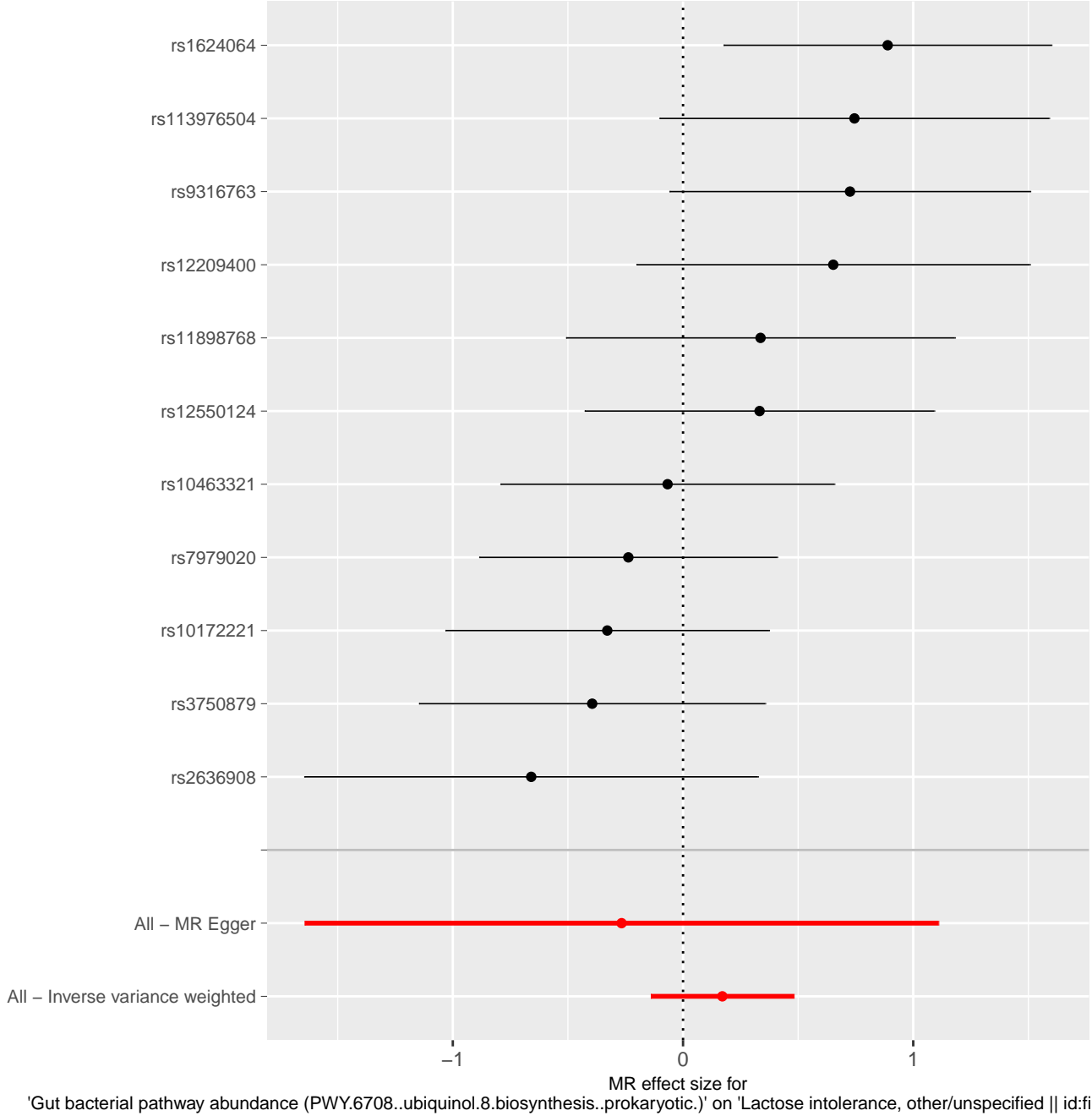

Supplement: Supplementary file 1 [file Data_Sheet_1.zip › supplementary materials/Forward/forest plot/ebi-a-GCST90027596.finngen_R12_E4_LACTONAS.pdf]

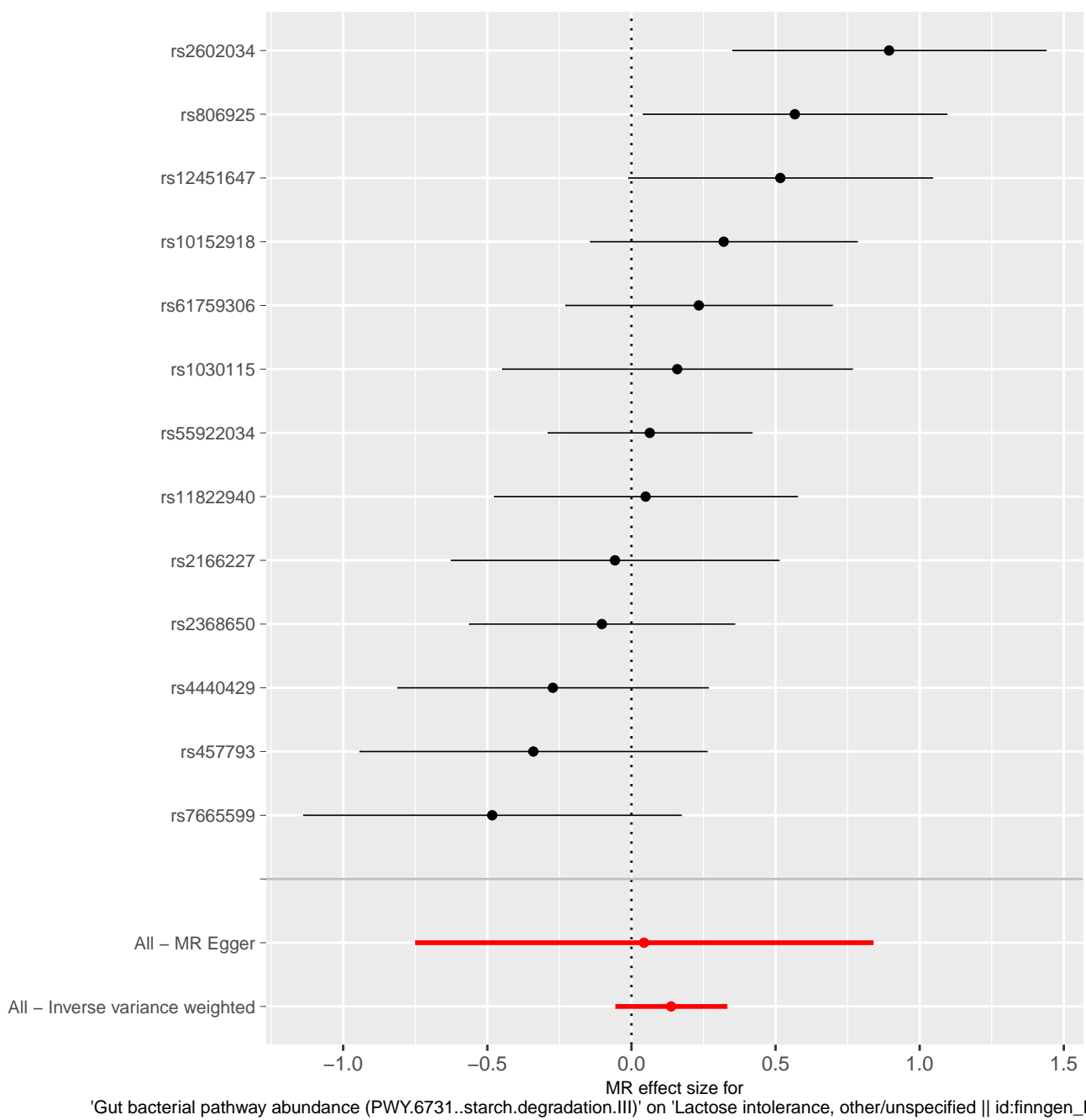

Supplement: Supplementary file 1 [file Data_Sheet_1.zip › supplementary materials/Forward/forest plot/ebi-a-GCST90027597.finngen_R12_E4_LACTONAS.pdf]

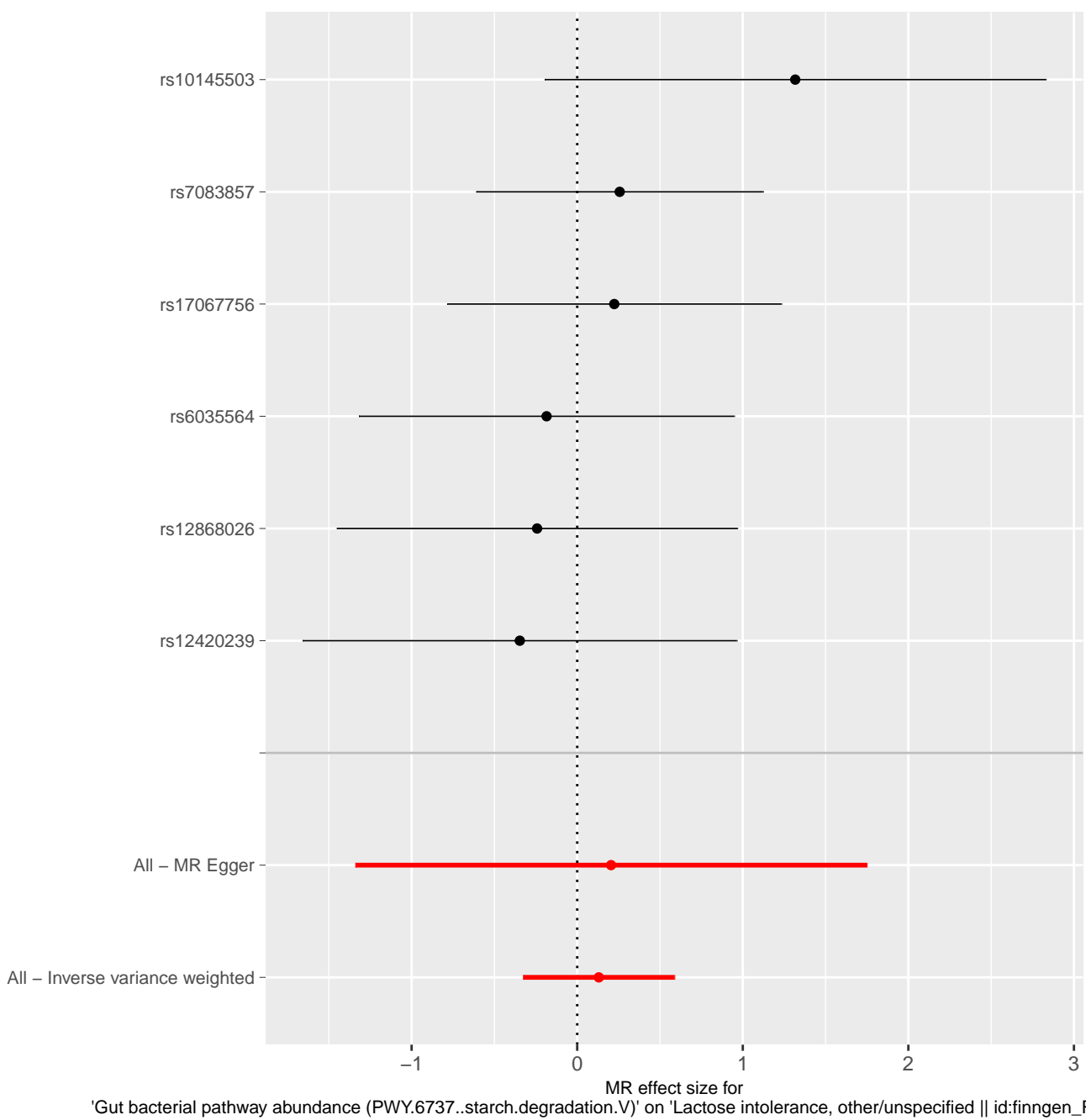

Supplement: Supplementary file 1 [file Data_Sheet_1.zip › supplementary materials/Forward/forest plot/ebi-a-GCST90027598.finngen_R12_E4_LACTONAS.pdf]

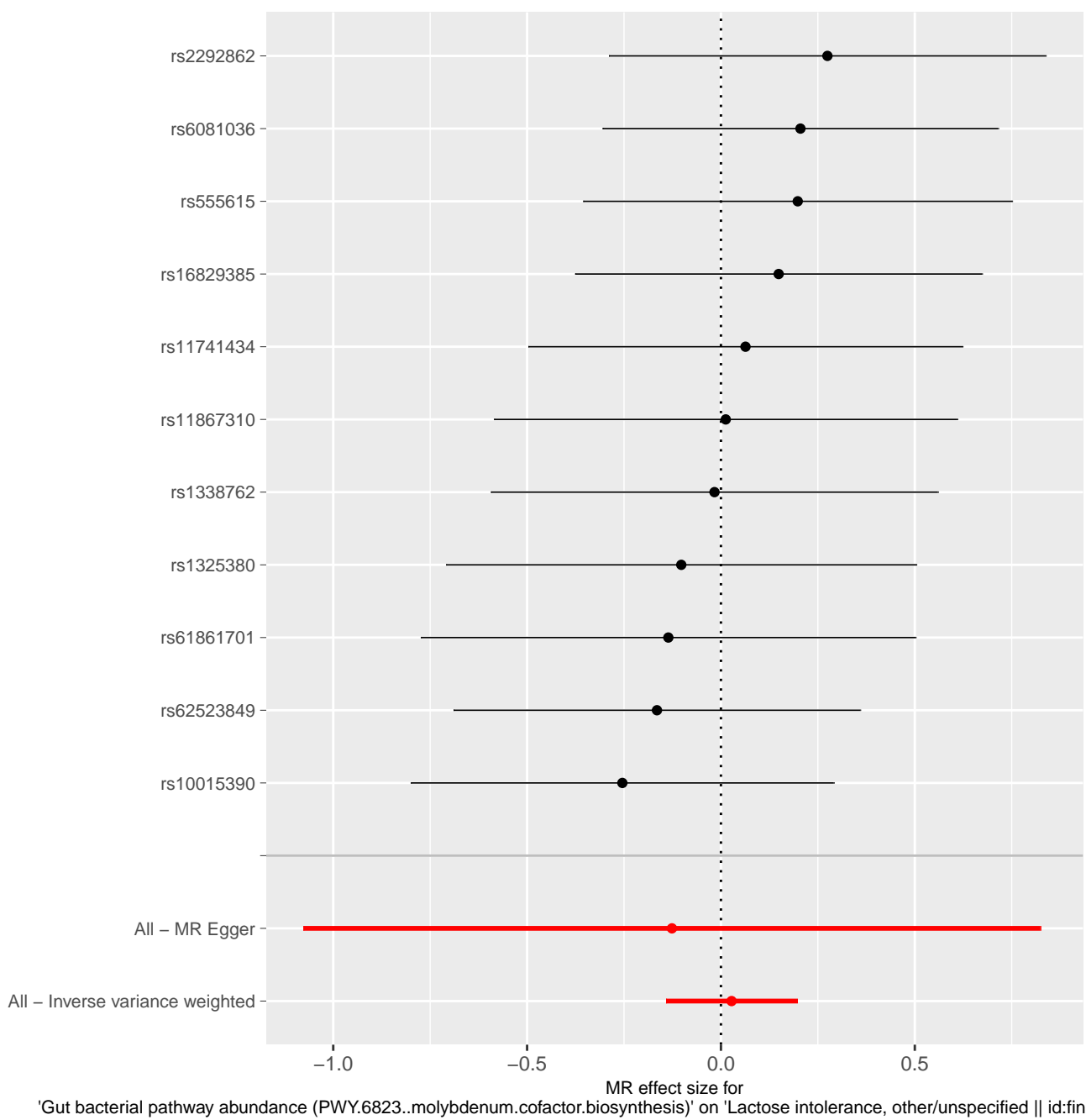

Supplement: Supplementary file 1 [file Data_Sheet_1.zip › supplementary materials/Forward/forest plot/ebi-a-GCST90027599.finngen_R12_E4_LACTONAS.pdf]

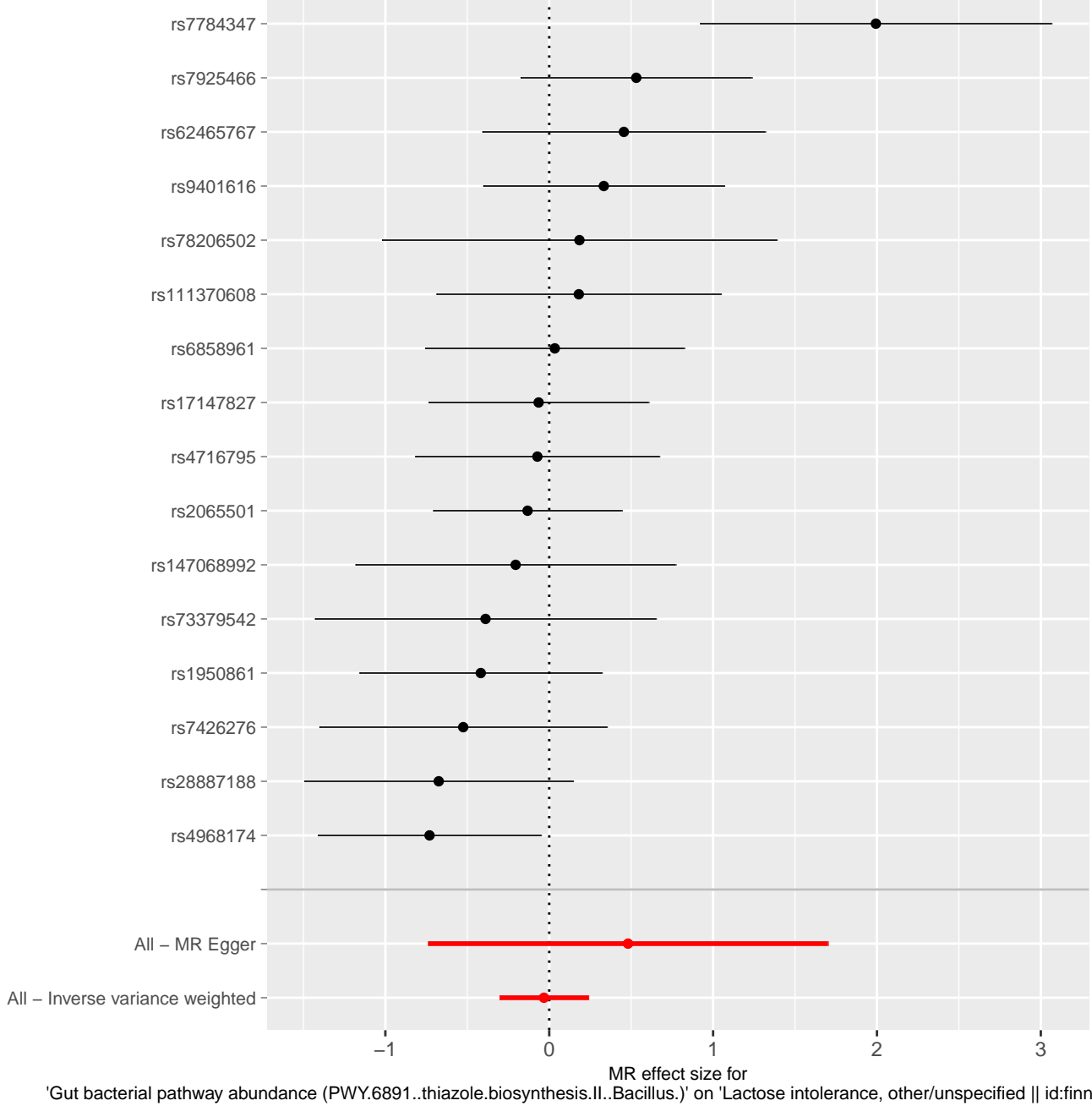

Supplement: Supplementary file 1 [file Data_Sheet_1.zip › supplementary materials/Forward/forest plot/ebi-a-GCST90027600.finngen_R12_E4_LACTONAS.pdf]

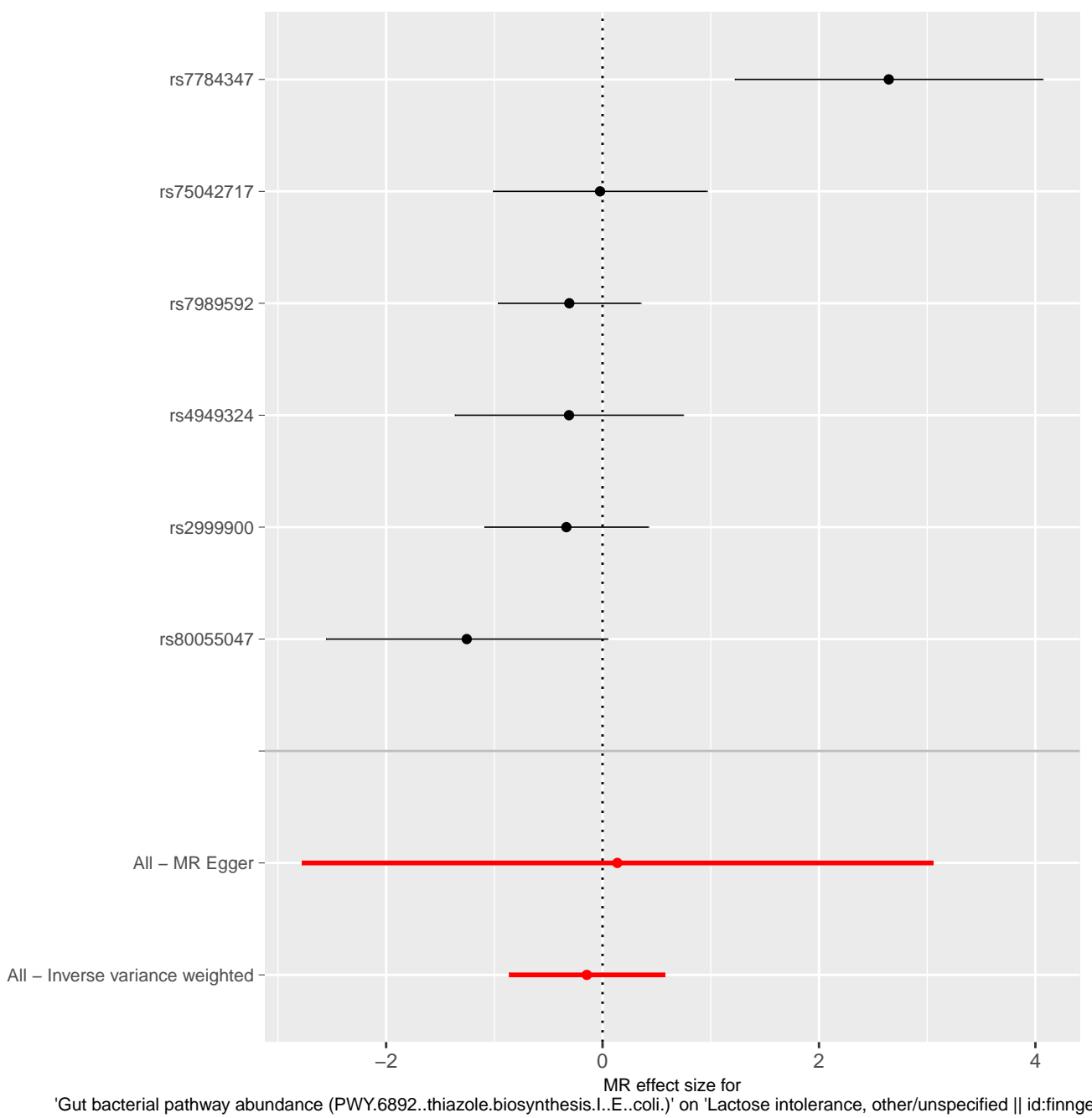

Supplement: Supplementary file 1 [file Data_Sheet_1.zip › supplementary materials/Forward/forest plot/ebi-a-GCST90027601.finngen_R12_E4_LACTONAS.pdf]

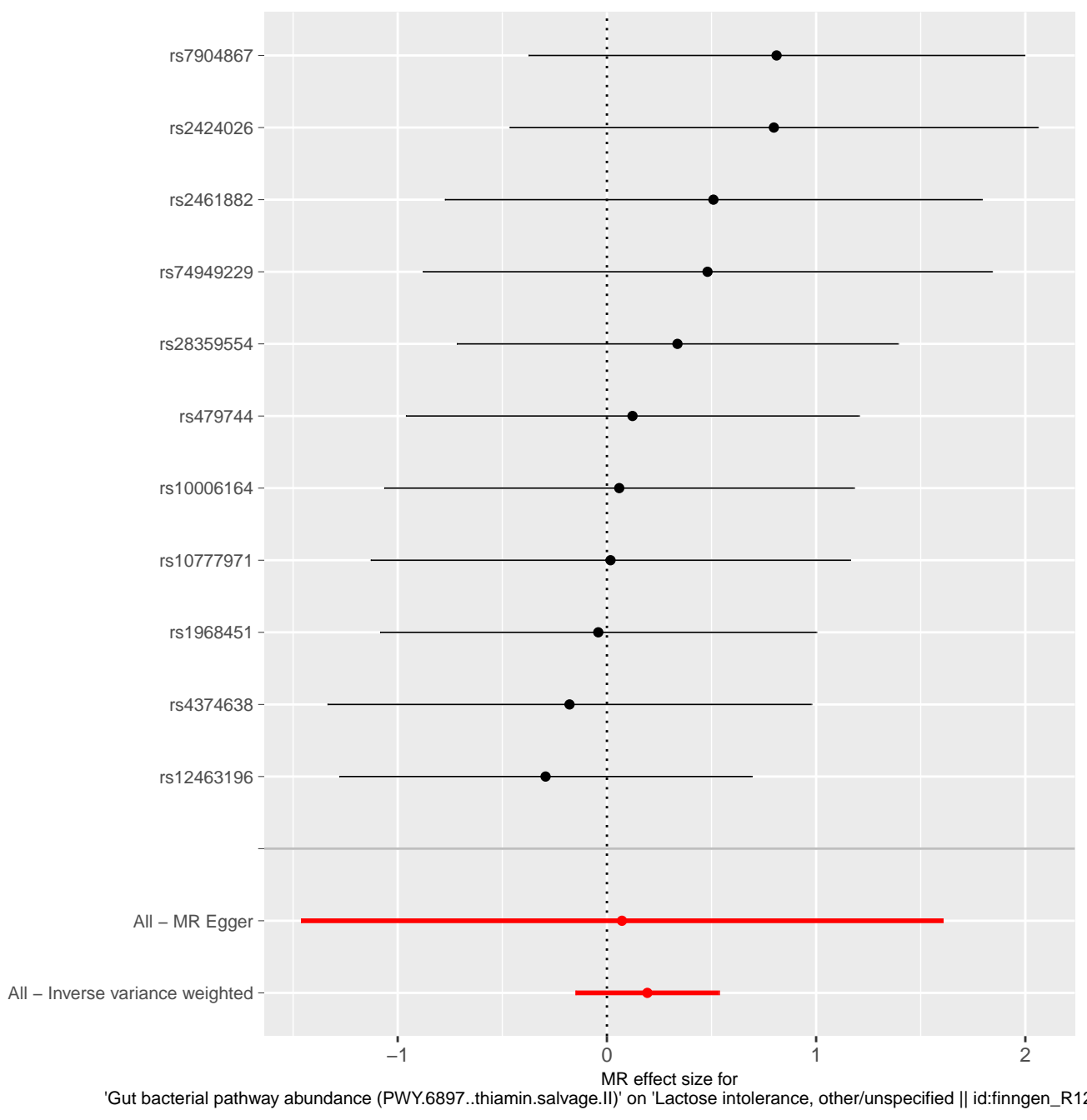

Supplement: Supplementary file 1 [file Data_Sheet_1.zip › supplementary materials/Forward/forest plot/ebi-a-GCST90027602.finngen_R12_E4_LACTONAS.pdf]

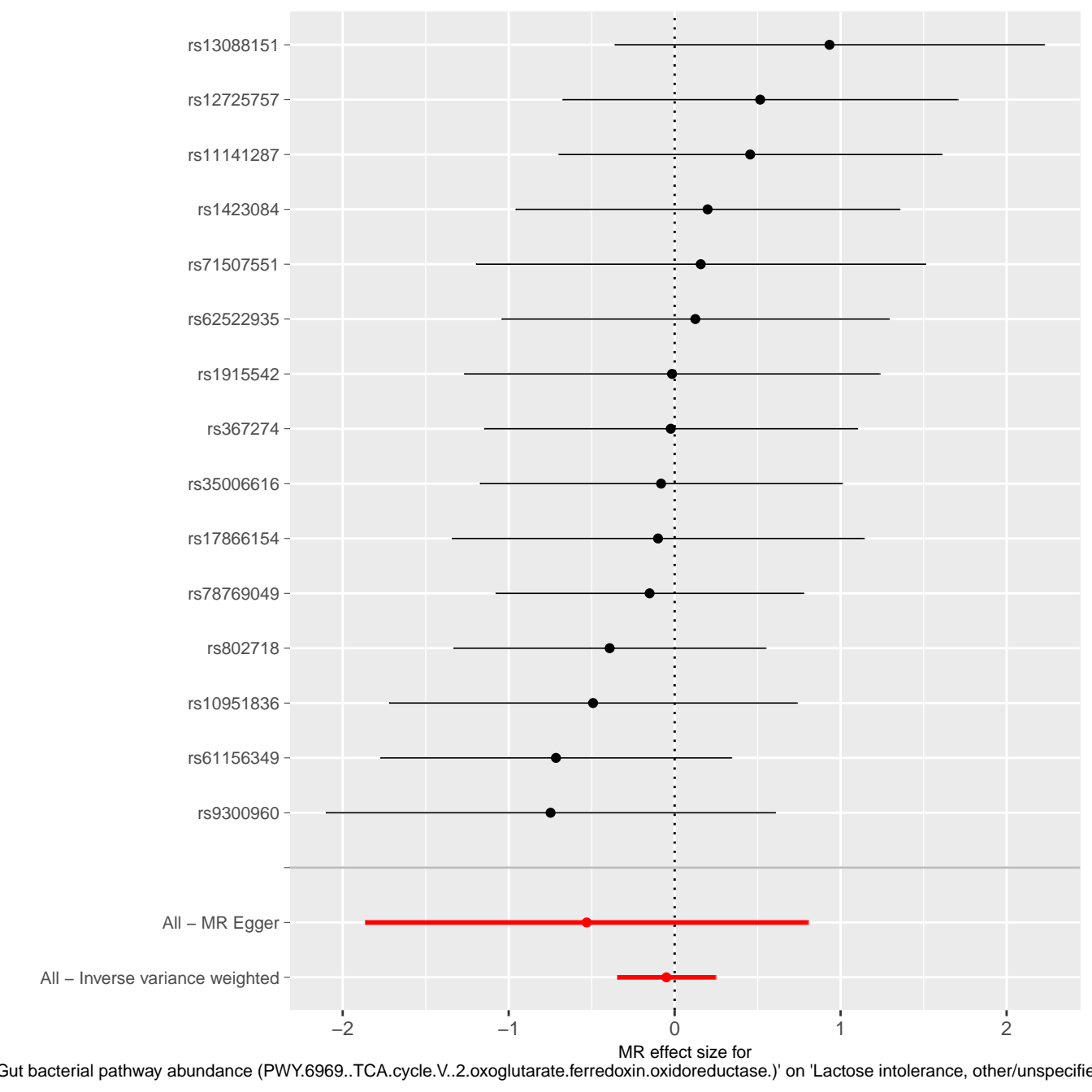

Supplement: Supplementary file 1 [file Data_Sheet_1.zip › supplementary materials/Forward/forest plot/ebi-a-GCST90027604.finngen_R12_E4_LACTONAS.pdf]

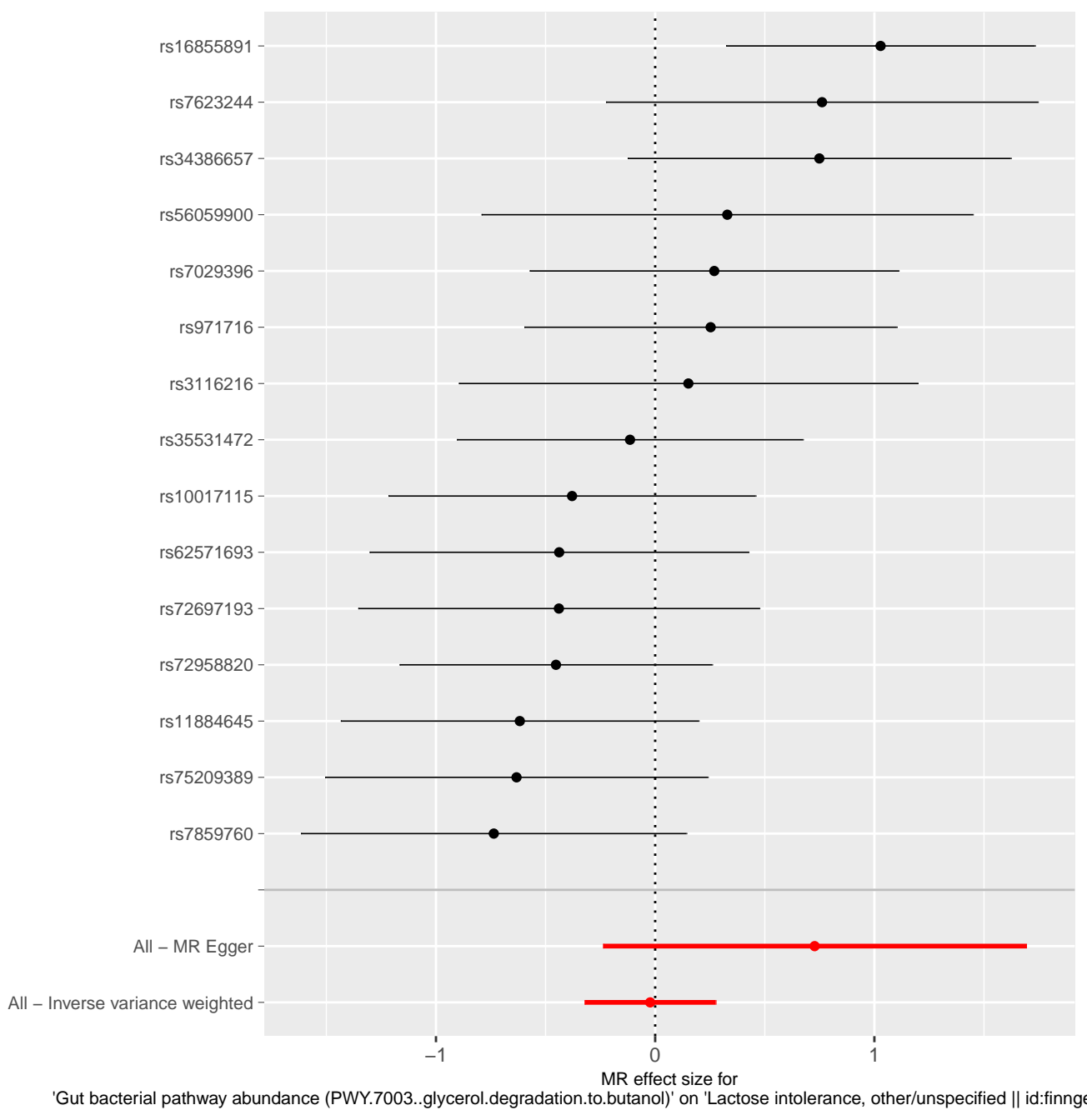

Supplement: Supplementary file 1 [file Data_Sheet_1.zip › supplementary materials/Forward/forest plot/ebi-a-GCST90027605.finngen_R12_E4_LACTONAS.pdf]

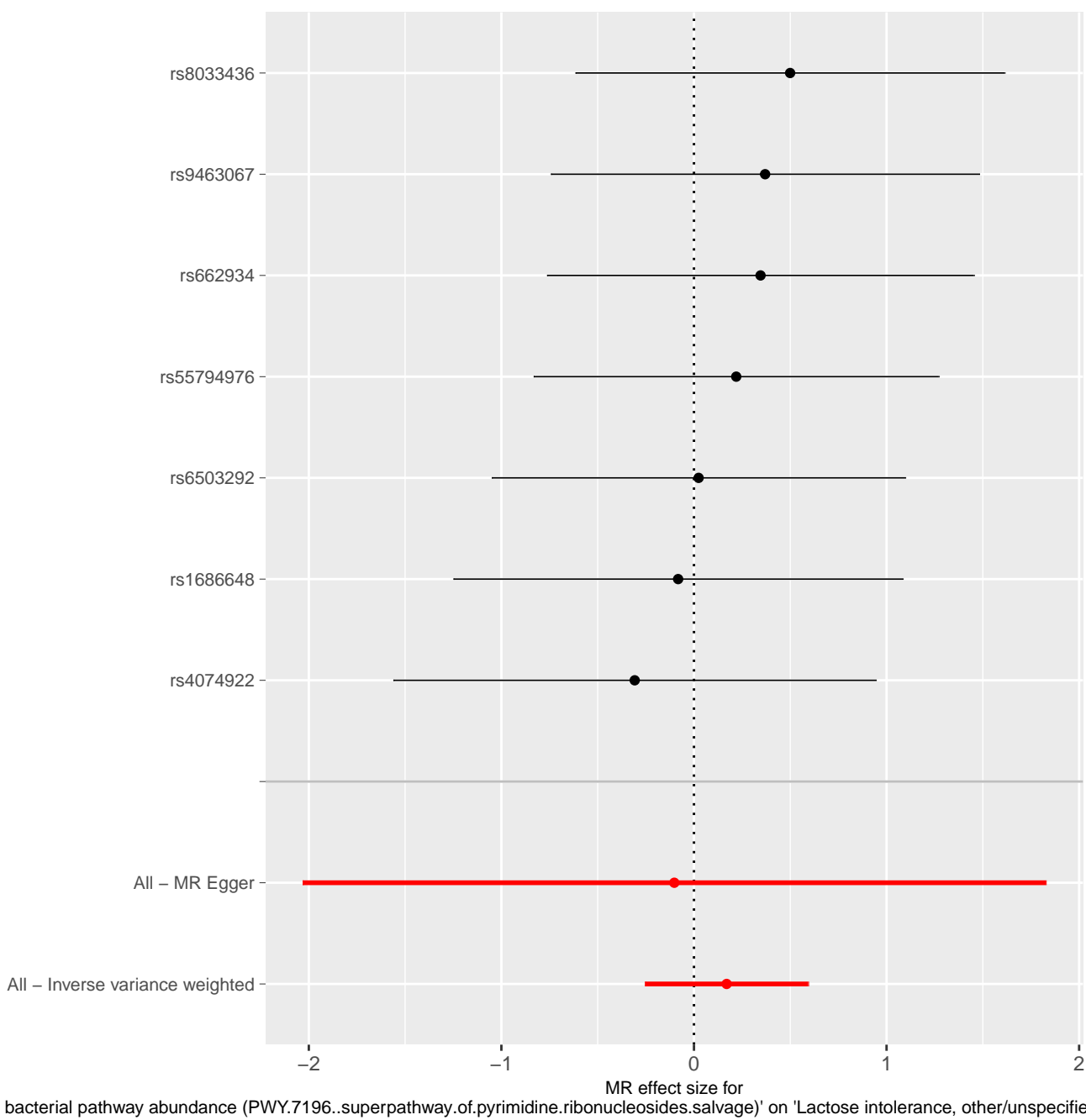

Supplement: Supplementary file 1 [file Data_Sheet_1.zip › supplementary materials/Forward/forest plot/ebi-a-GCST90027608.finngen_R12_E4_LACTONAS.pdf]

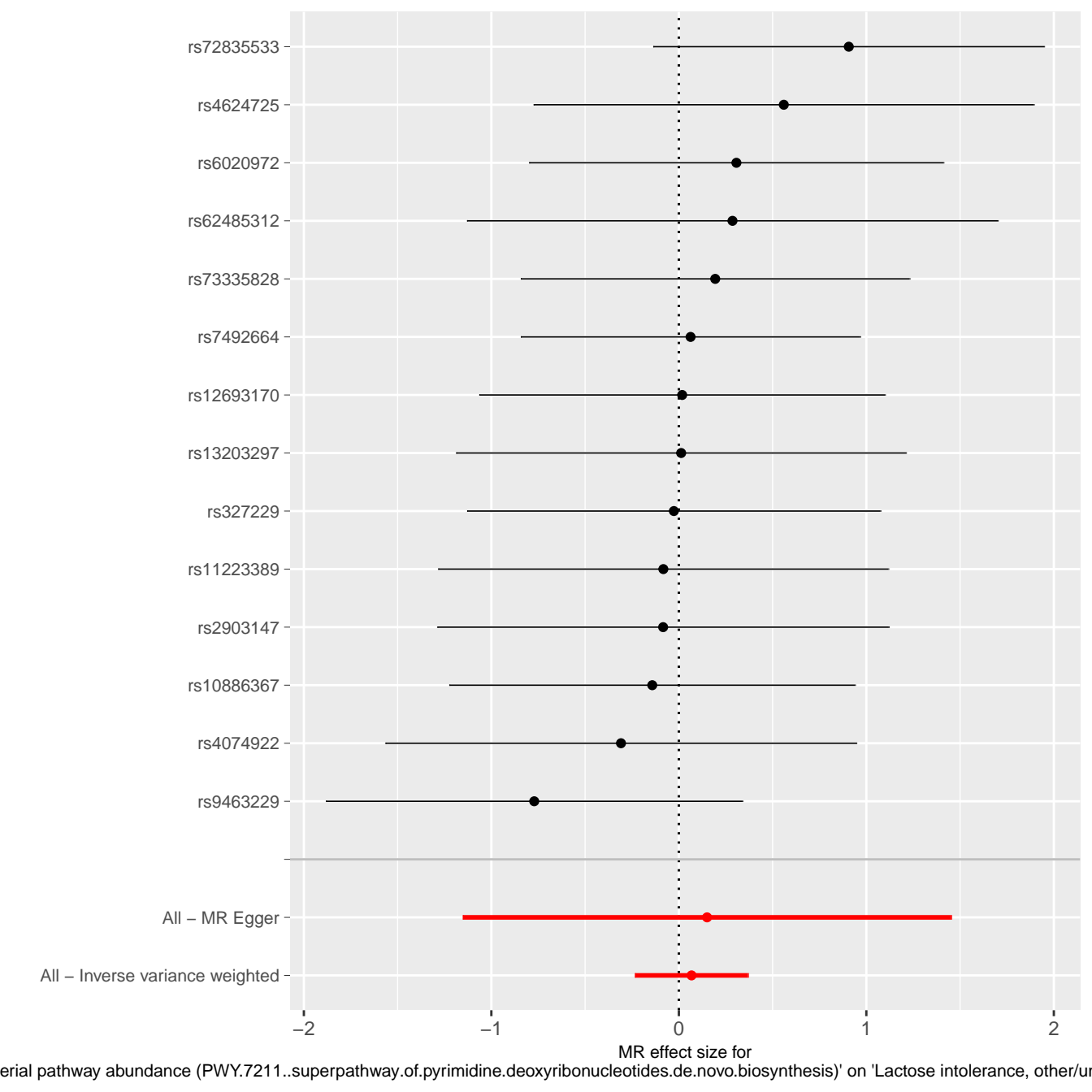

Supplement: Supplementary file 1 [file Data_Sheet_1.zip › supplementary materials/Forward/forest plot/ebi-a-GCST90027613.finngen_R12_E4_LACTONAS.pdf]

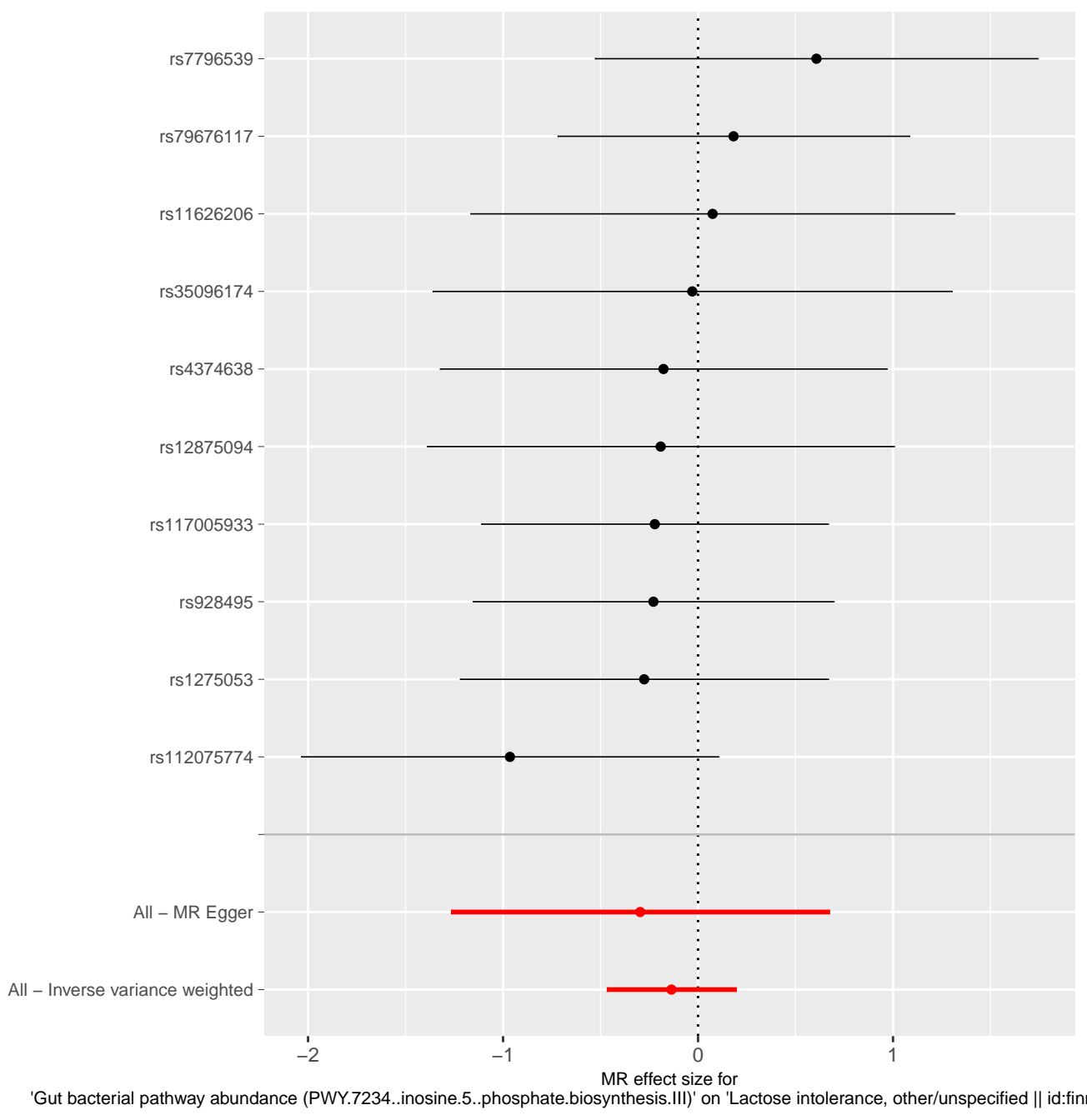

Supplement: Supplementary file 1 [file Data_Sheet_1.zip › supplementary materials/Forward/forest plot/ebi-a-GCST90027616.finngen_R12_E4_LACTONAS.pdf]

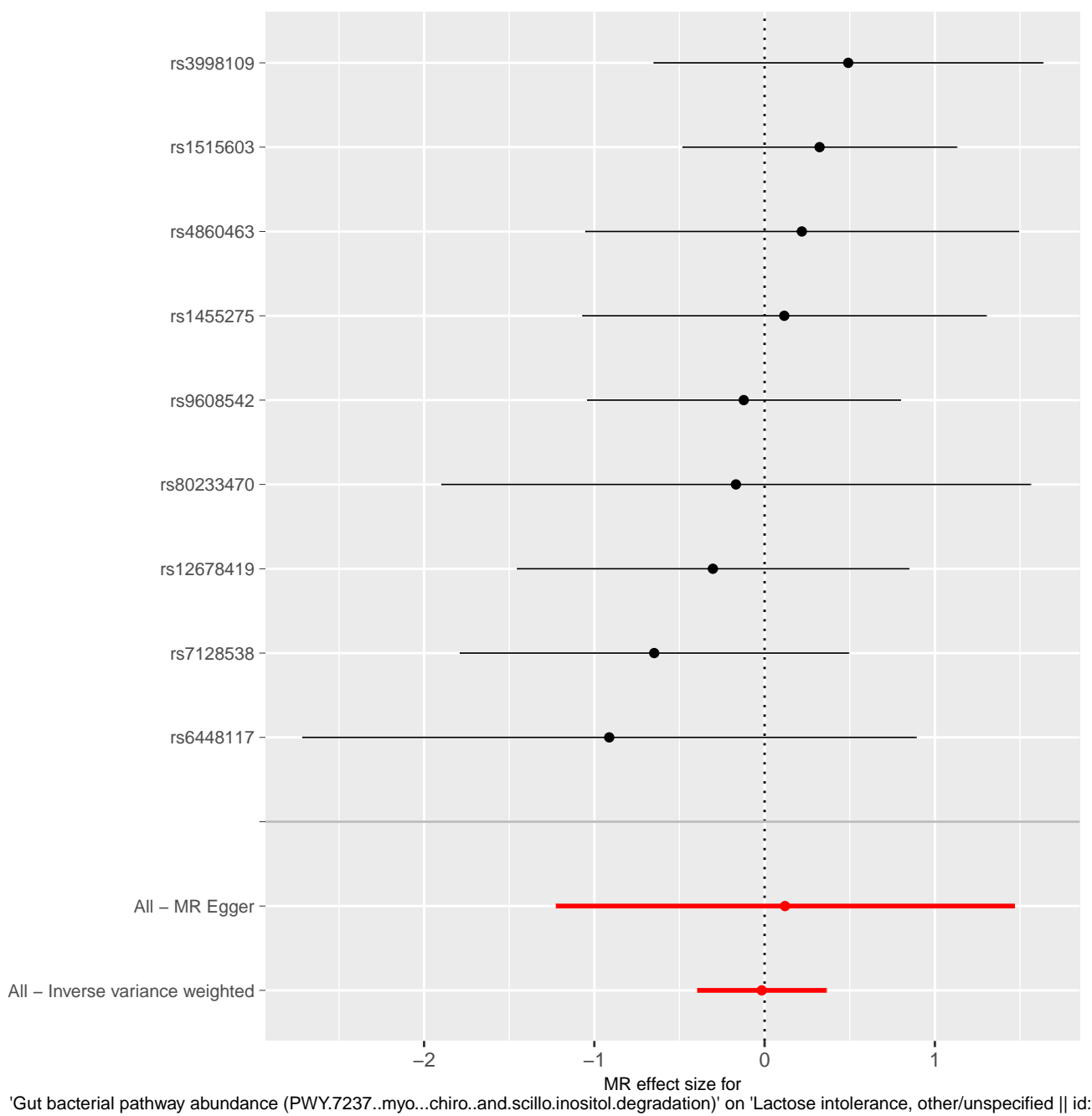

Supplement: Supplementary file 1 [file Data_Sheet_1.zip › supplementary materials/Forward/forest plot/ebi-a-GCST90027617.finngen_R12_E4_LACTONAS.pdf]

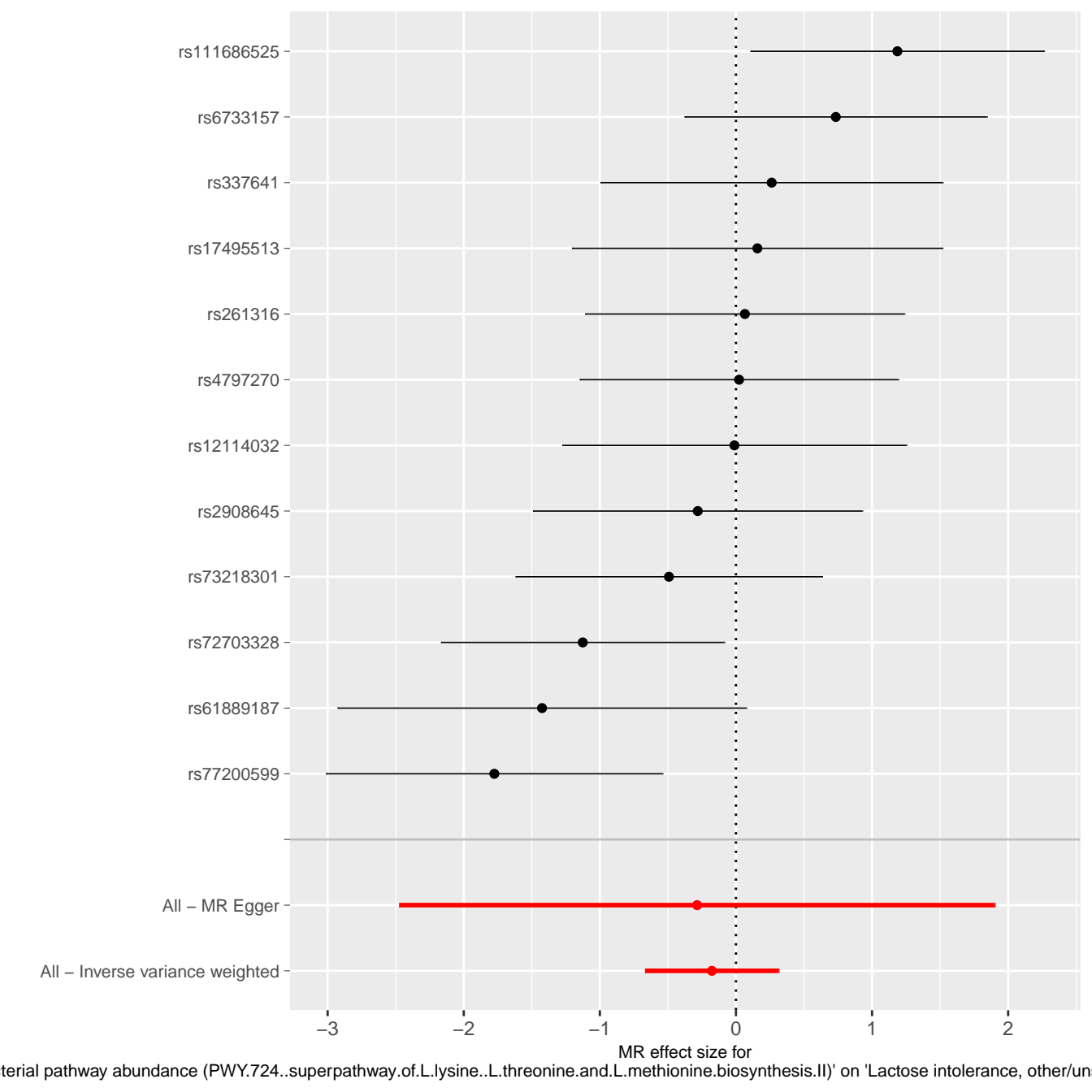

Supplement: Supplementary file 1 [file Data_Sheet_1.zip › supplementary materials/Forward/forest plot/ebi-a-GCST90027618.finngen_R12_E4_LACTONAS.pdf]

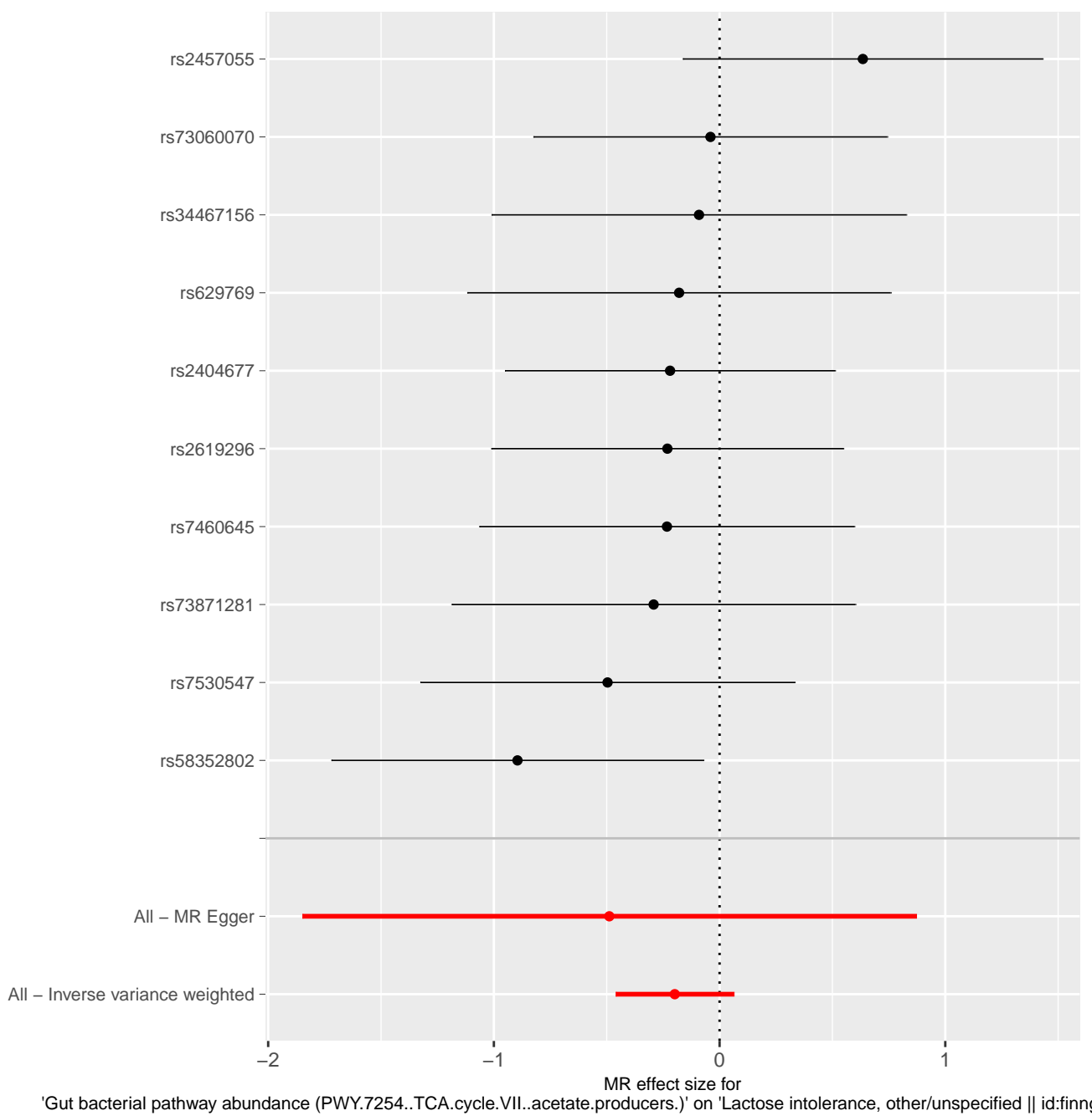

Supplement: Supplementary file 1 [file Data_Sheet_1.zip › supplementary materials/Forward/forest plot/ebi-a-GCST90027619.finngen_R12_E4_LACTONAS.pdf]

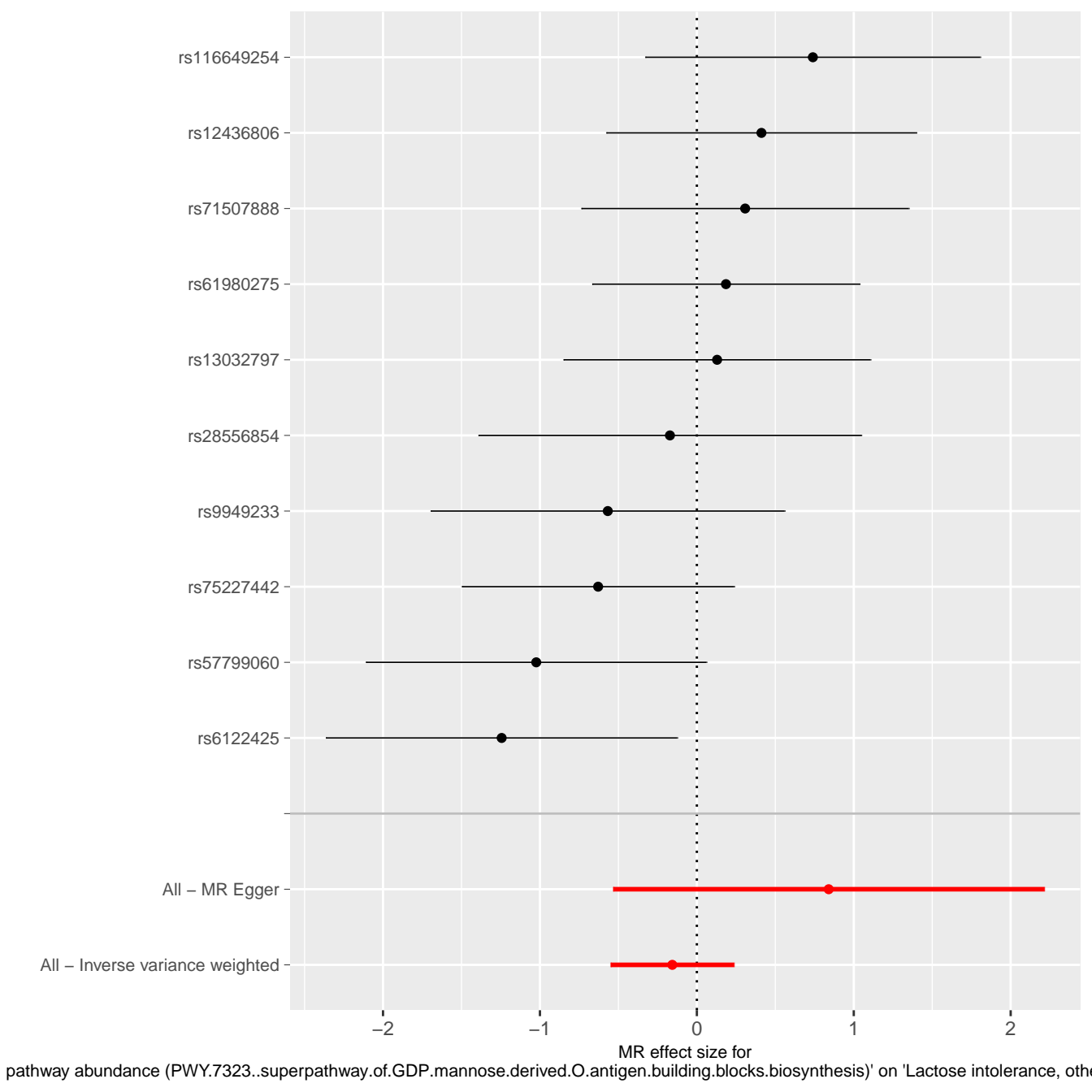

Supplement: Supplementary file 1 [file Data_Sheet_1.zip › supplementary materials/Forward/forest plot/ebi-a-GCST90027621.finngen_R12_E4_LACTONAS.pdf]

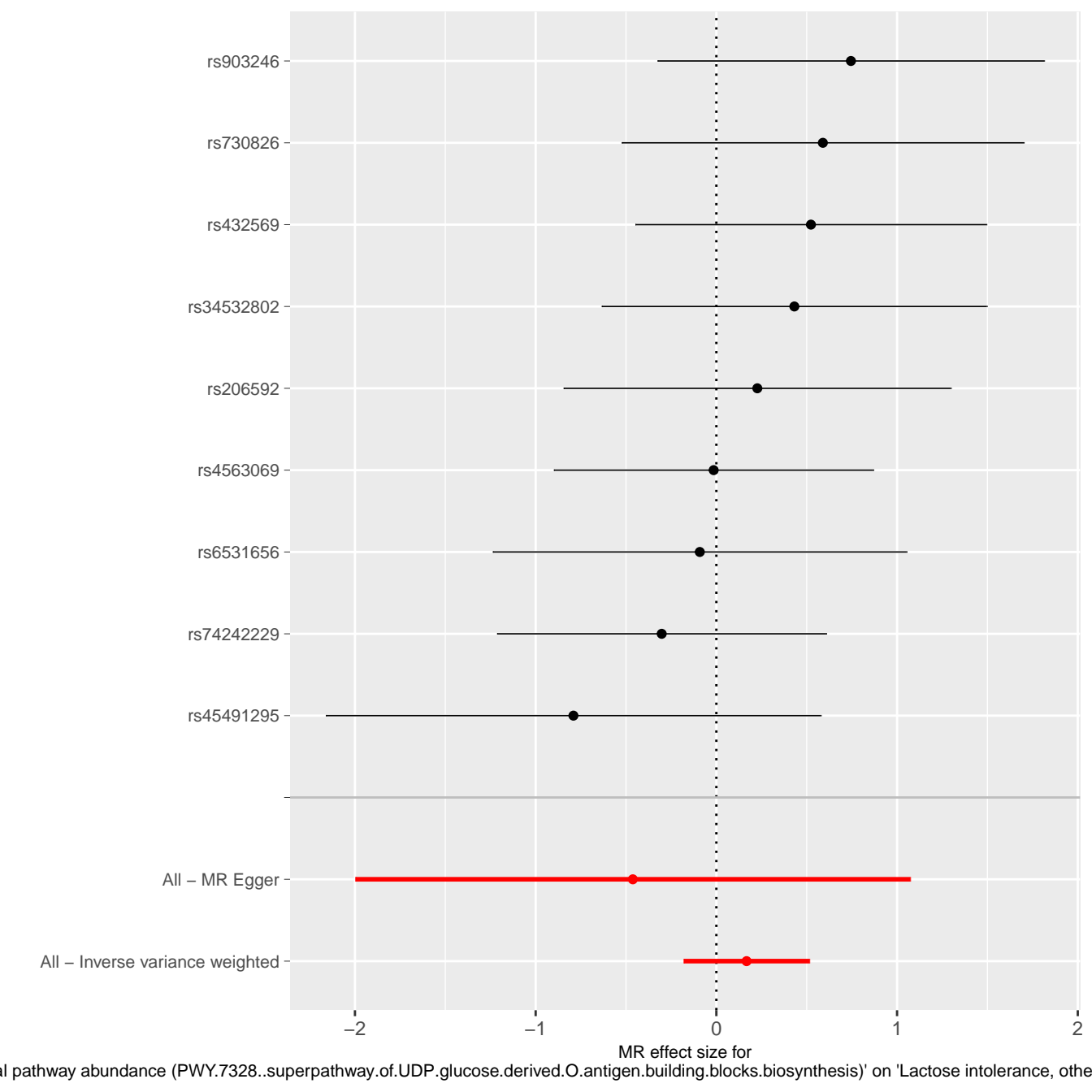

Supplement: Supplementary file 1 [file Data_Sheet_1.zip › supplementary materials/Forward/forest plot/ebi-a-GCST90027622.finngen_R12_E4_LACTONAS.pdf]

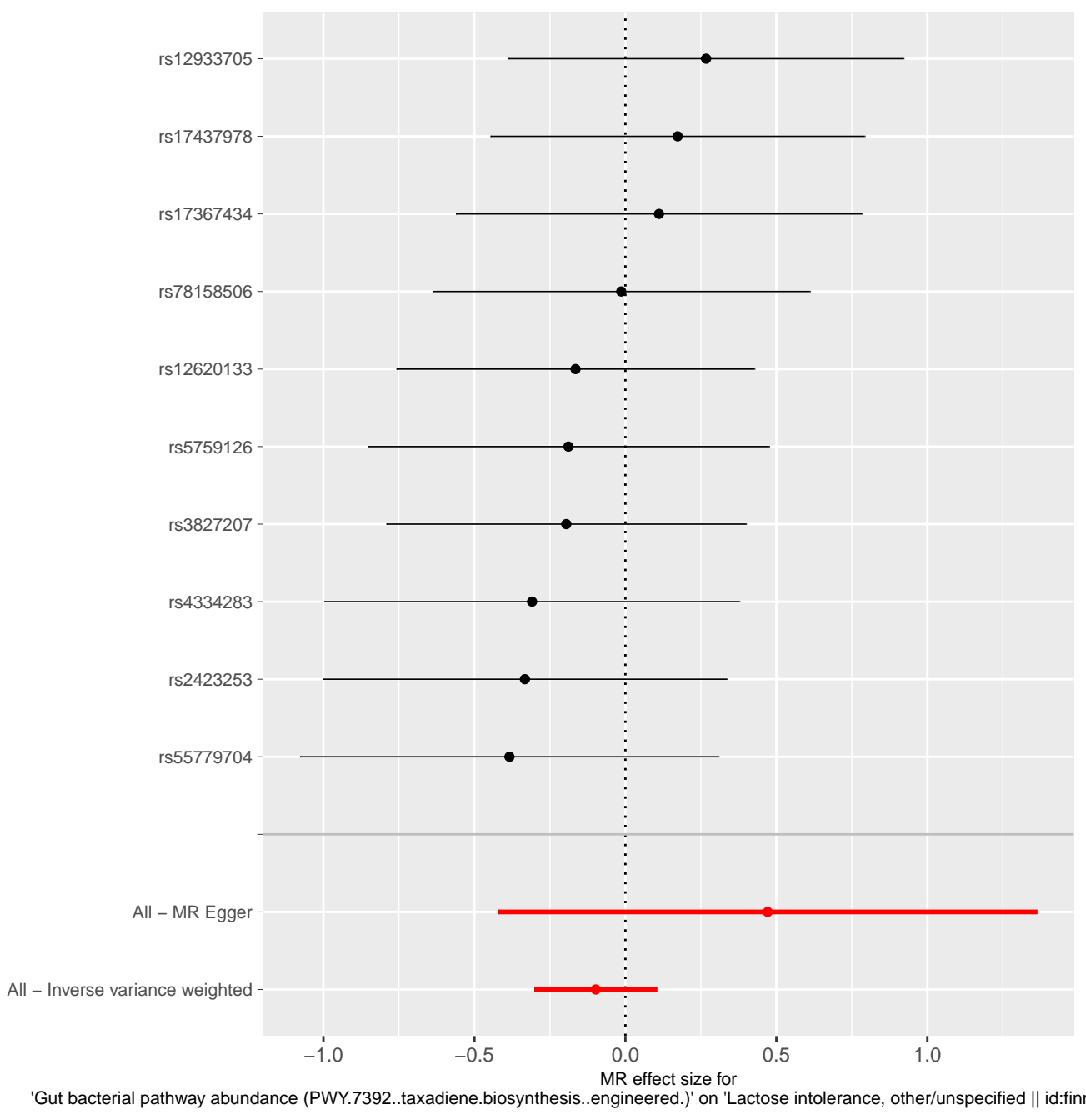

Supplement: Supplementary file 1 [file Data_Sheet_1.zip › supplementary materials/Forward/forest plot/ebi-a-GCST90027624.finngen_R12_E4_LACTONAS.pdf]

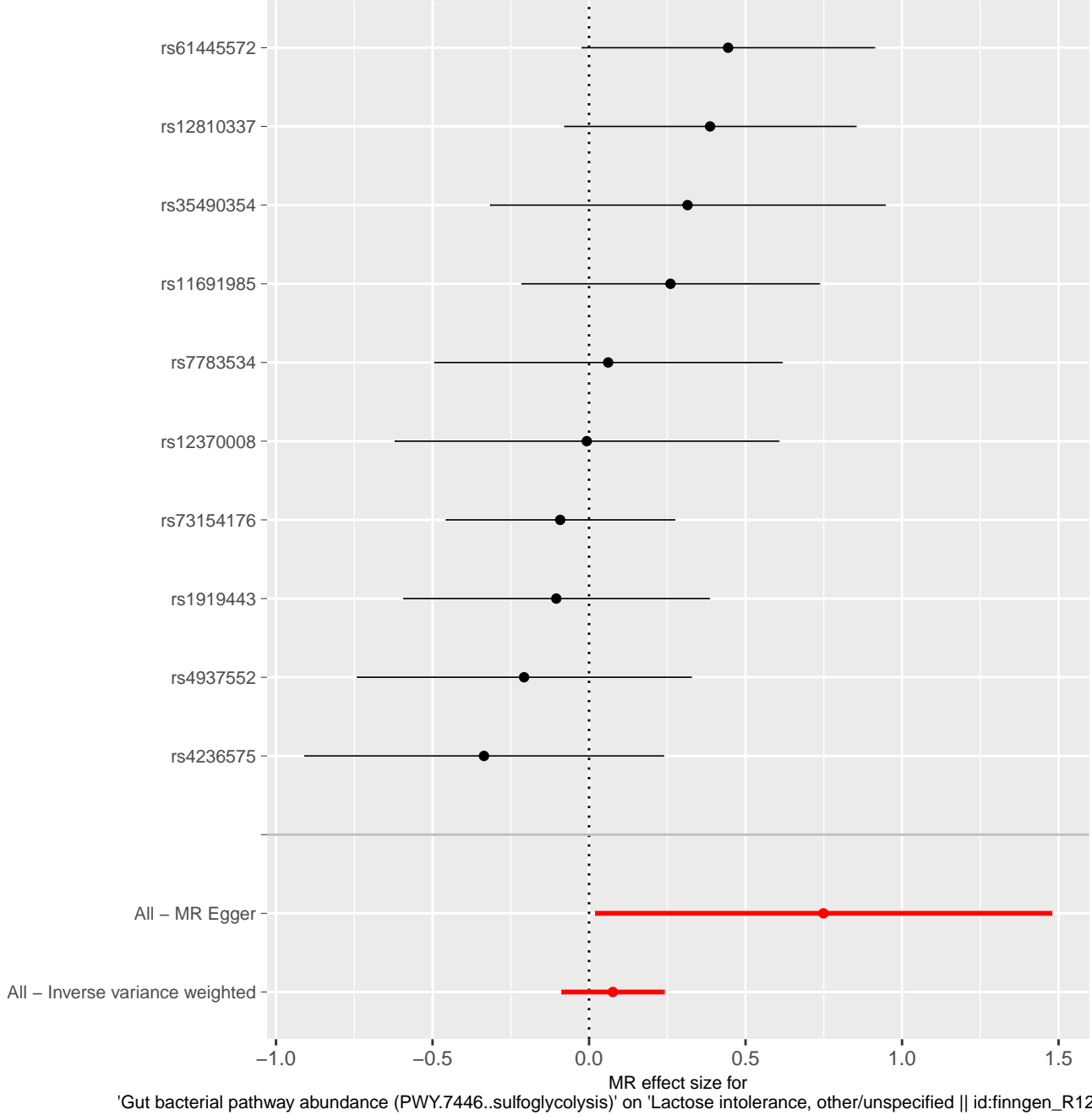

Supplement: Supplementary file 1 [file Data_Sheet_1.zip › supplementary materials/Forward/forest plot/ebi-a-GCST90027626.finngen_R12_E4_LACTONAS.pdf]

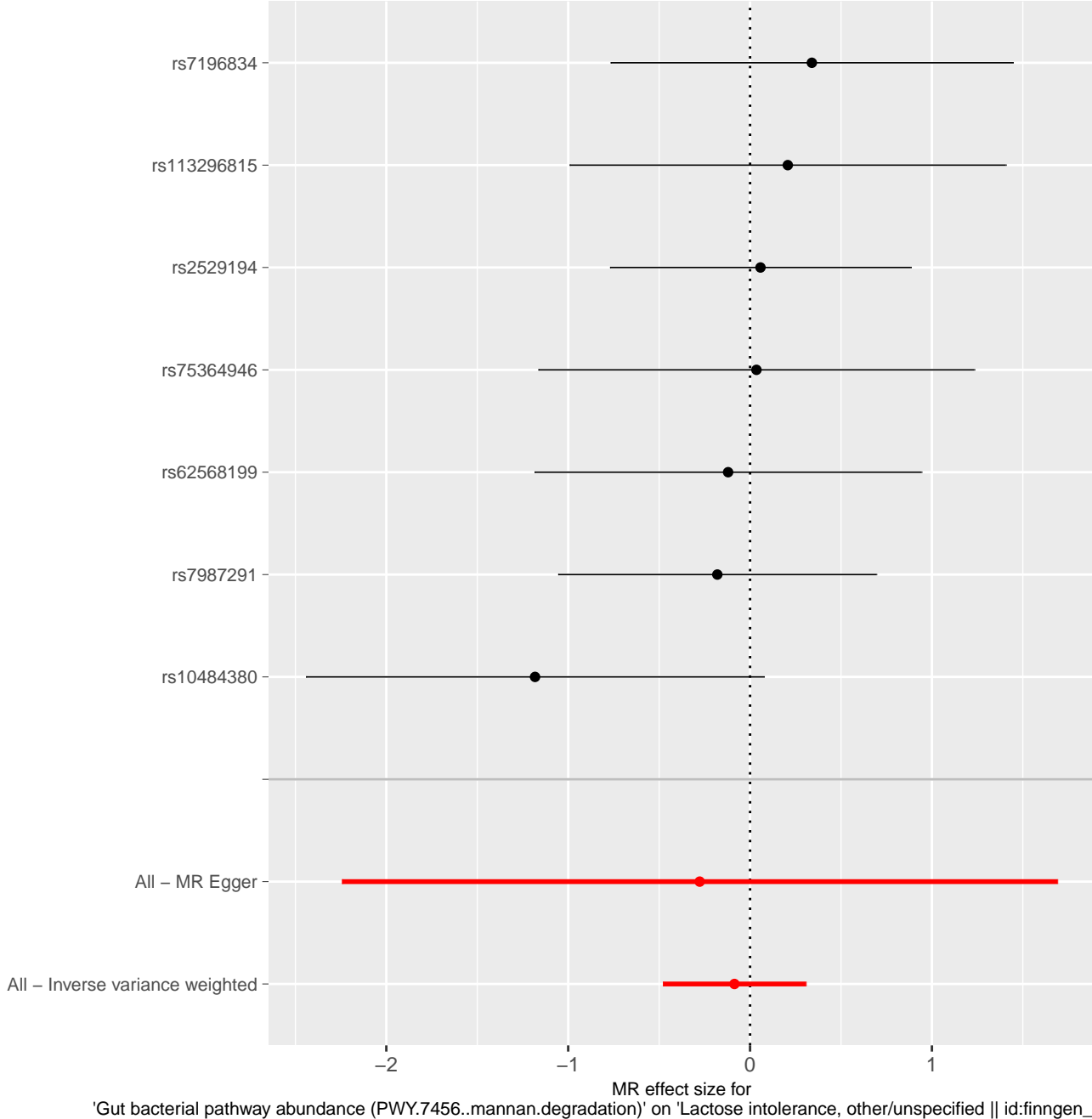

Supplement: Supplementary file 1 [file Data_Sheet_1.zip › supplementary materials/Forward/forest plot/ebi-a-GCST90027627.finngen_R12_E4_LACTONAS.pdf]

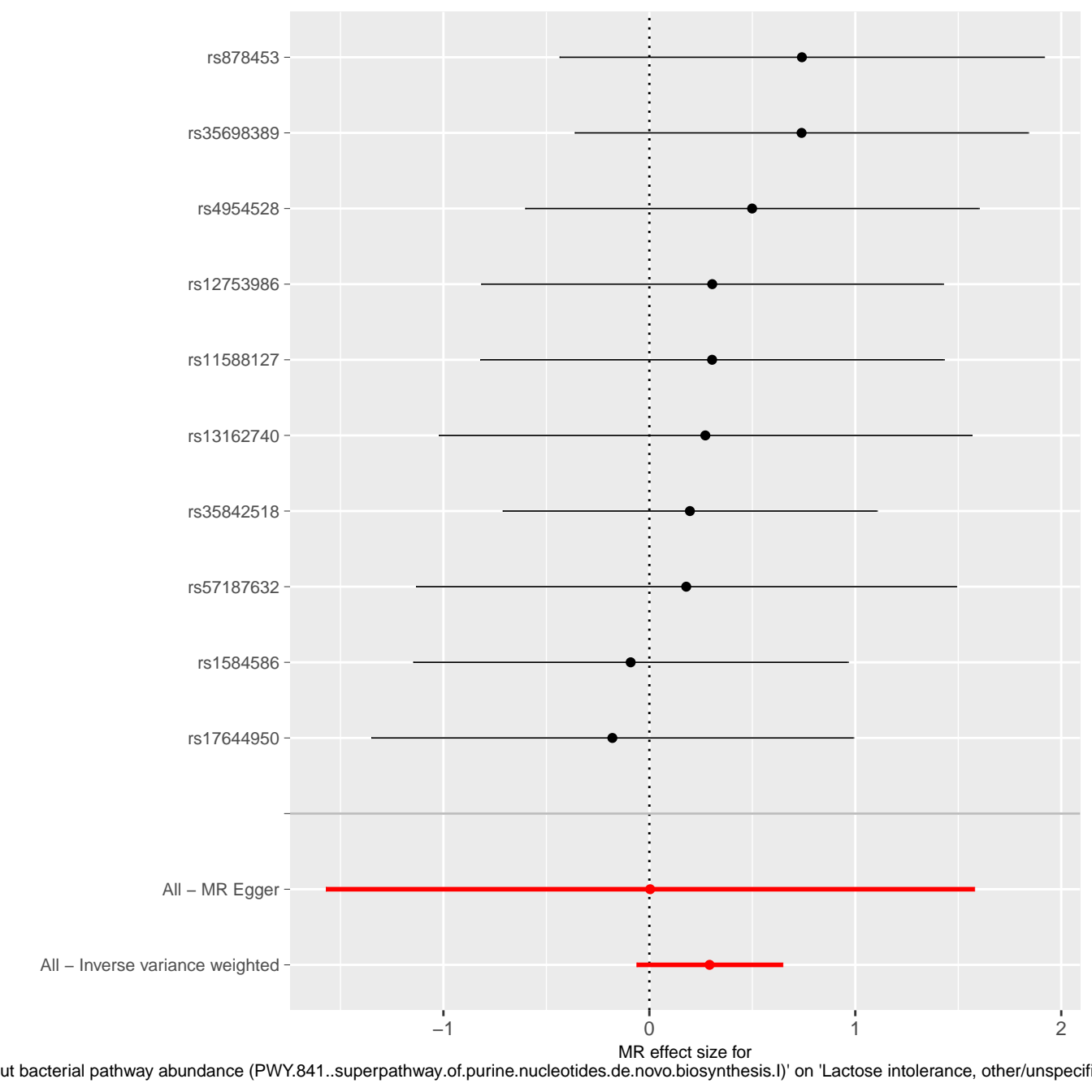

Supplement: Supplementary file 1 [file Data_Sheet_1.zip › supplementary materials/Forward/forest plot/ebi-a-GCST90027628.finngen_R12_E4_LACTONAS.pdf]

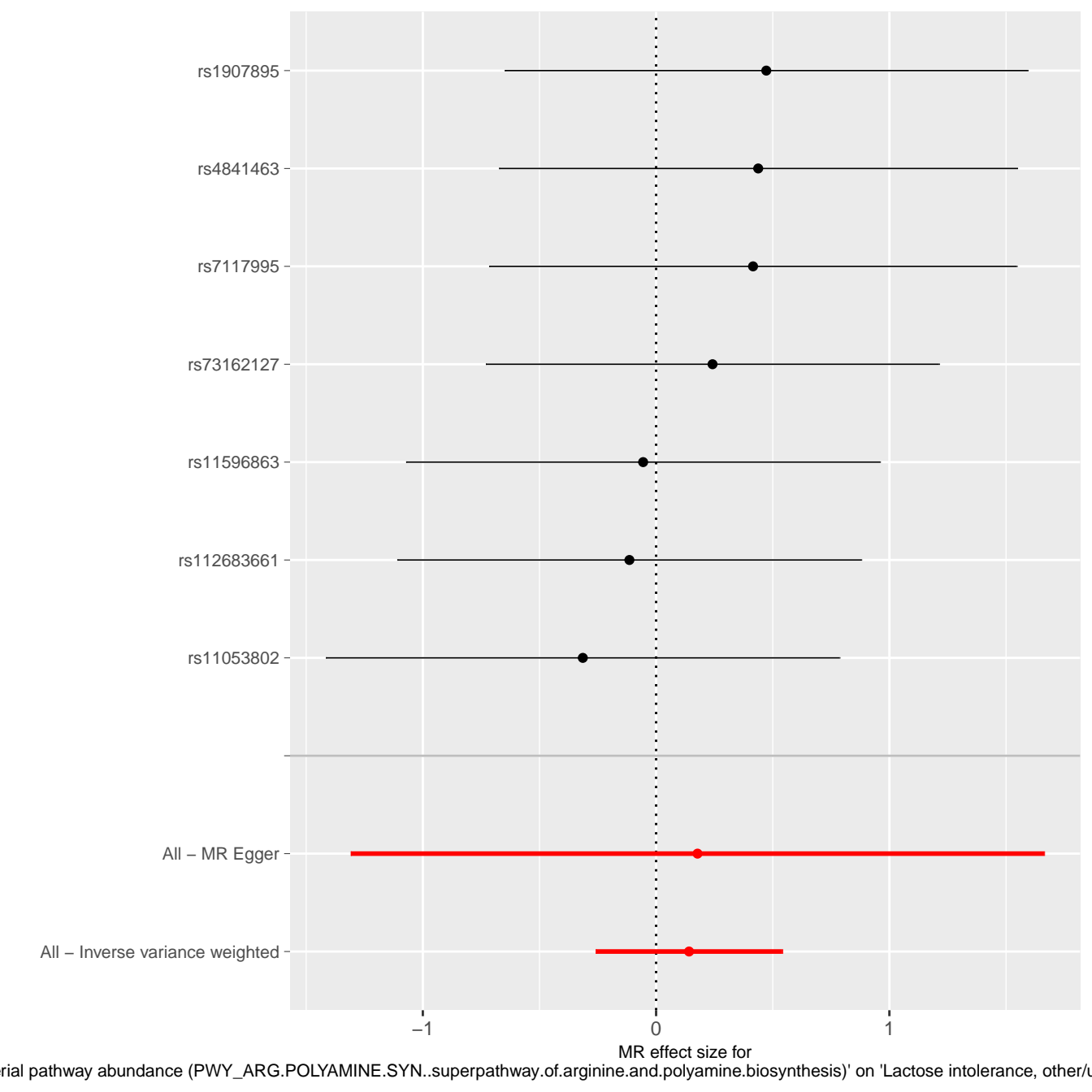

Supplement: Supplementary file 1 [file Data_Sheet_1.zip › supplementary materials/Forward/forest plot/ebi-a-GCST90027629.finngen_R12_E4_LACTONAS.pdf]

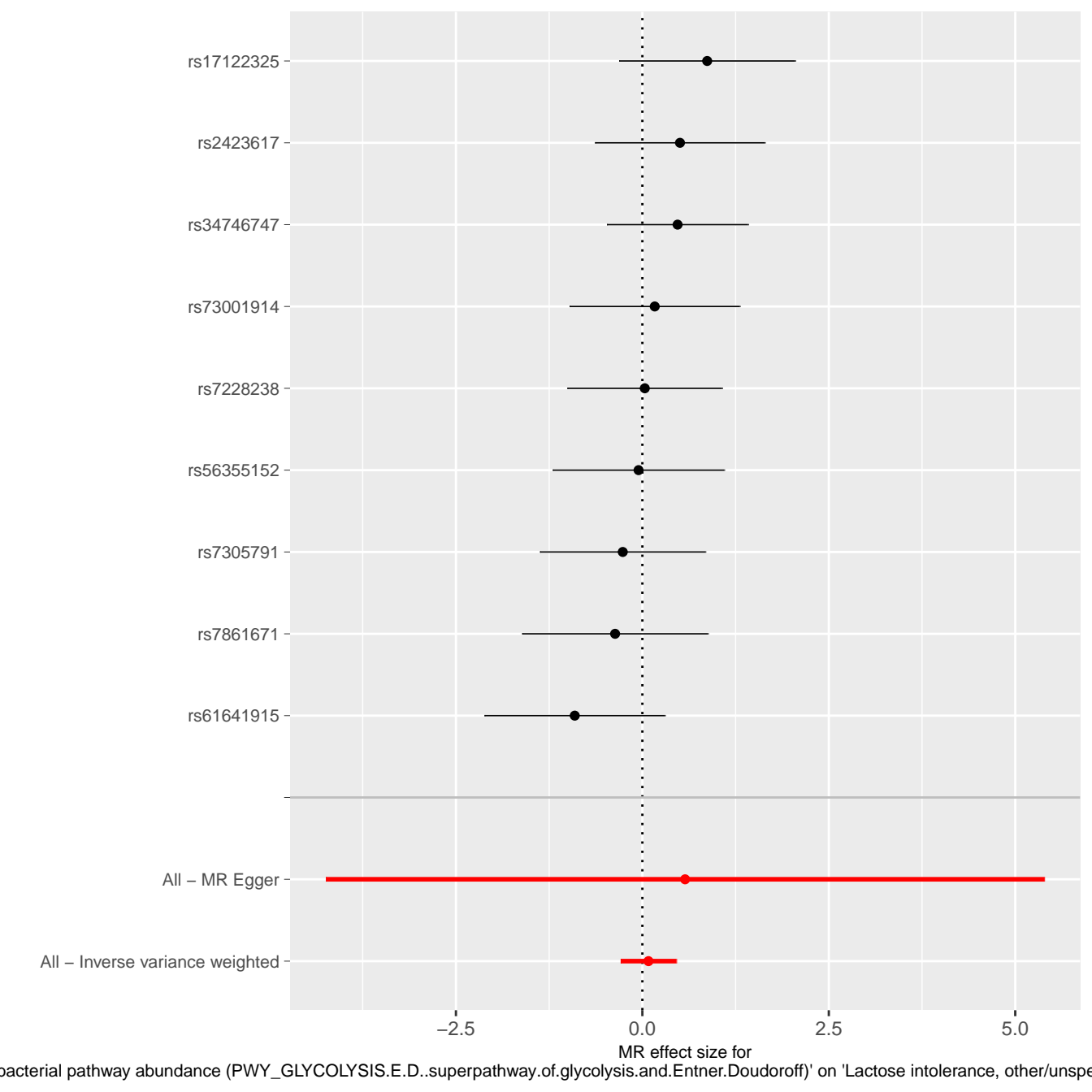

Supplement: Supplementary file 1 [file Data_Sheet_1.zip › supplementary materials/Forward/forest plot/ebi-a-GCST90027630.finngen_R12_E4_LACTONAS.pdf]

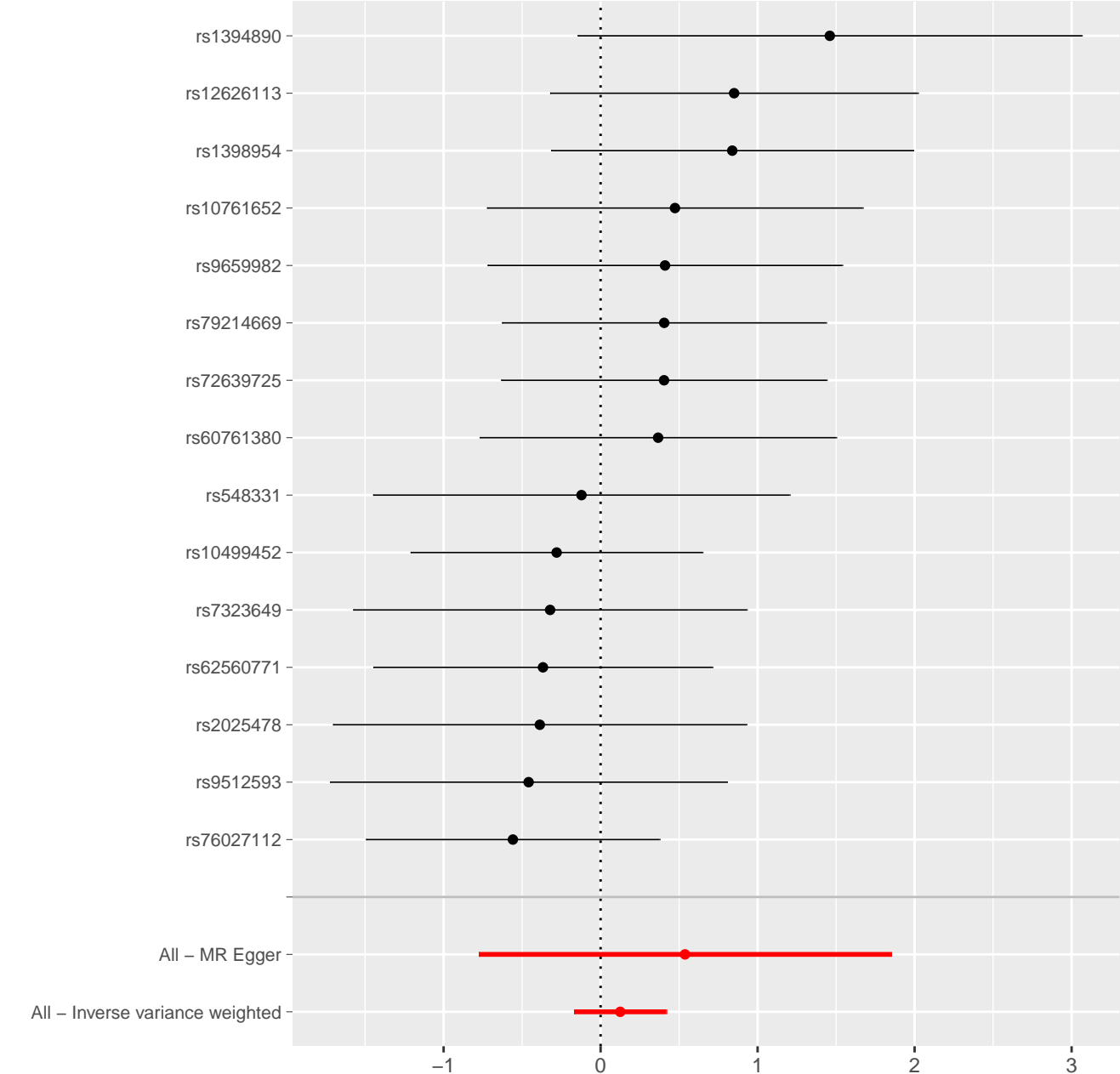

Supplement: Supplementary file 1 [file Data_Sheet_1.zip › supplementary materials/Forward/forest plot/ebi-a-GCST90027631.finngen_R12_E4_LACTONAS.pdf]

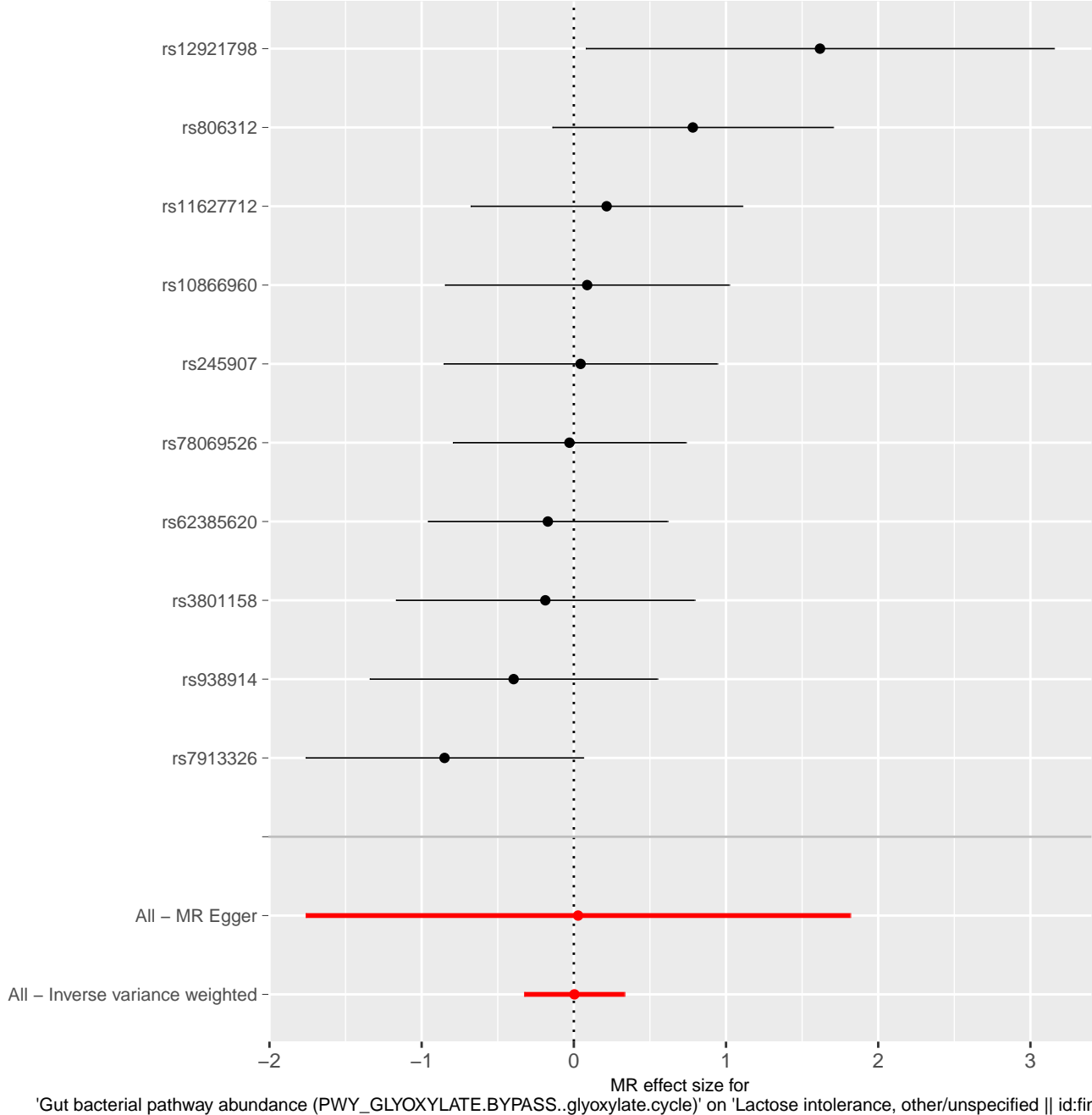

Supplement: Supplementary file 1 [file Data_Sheet_1.zip › supplementary materials/Forward/forest plot/ebi-a-GCST90027632.finngen_R12_E4_LACTONAS.pdf]

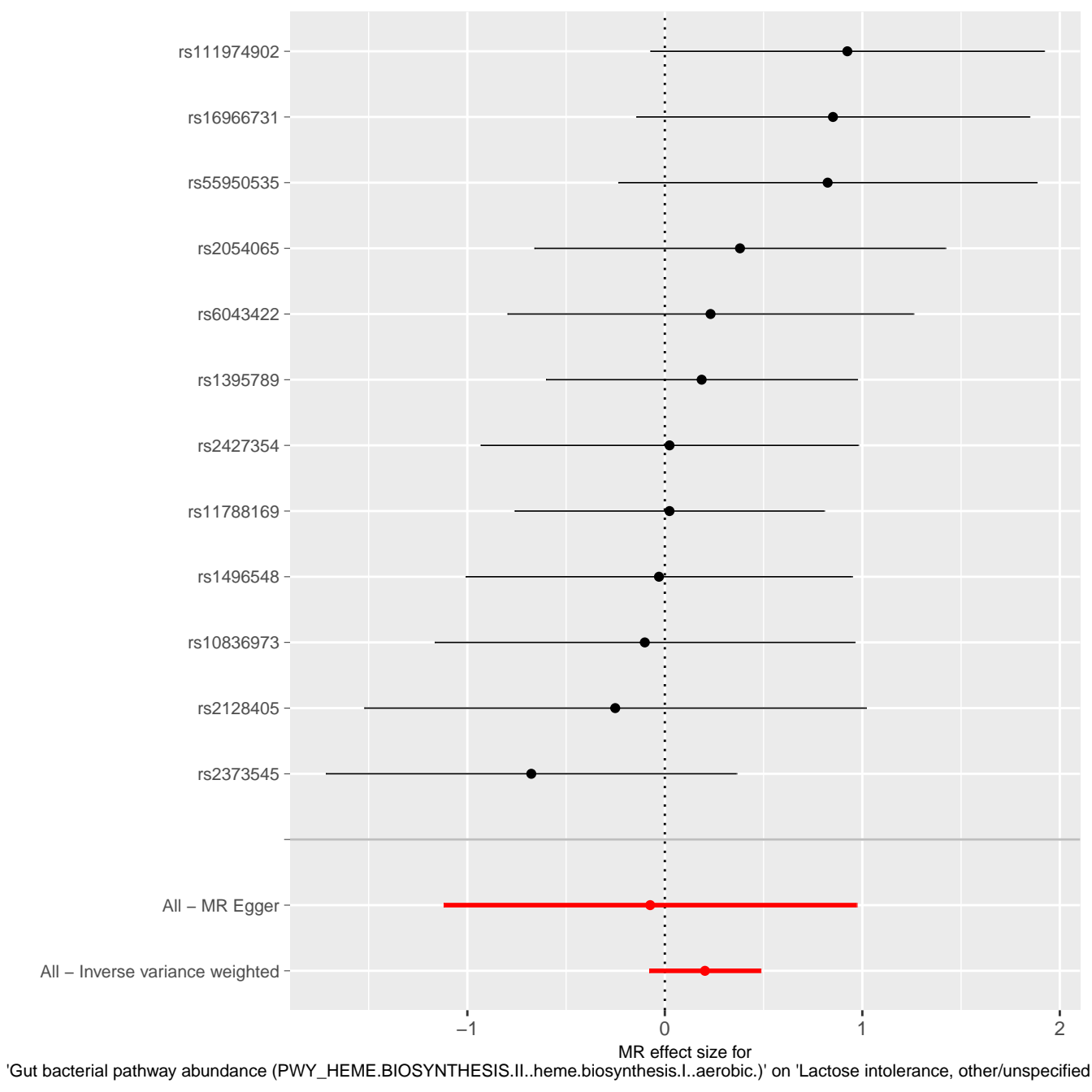

Supplement: Supplementary file 1 [file Data_Sheet_1.zip › supplementary materials/Forward/forest plot/ebi-a-GCST90027633.finngen_R12_E4_LACTONAS.pdf]
