# Supplementary figures and images for: Causal interplay between lactose intolerance and gut microbiota: a combined bidirectional Mendelian randomization and in vivo validation study (part 1 of 2)
Source: Front Nutr. 2026 Jun 1;13:1803337. doi: 10.3389/fnut.2026.1803337 (PMC13265576; doi:10.3389/fnut.2026.1803337)

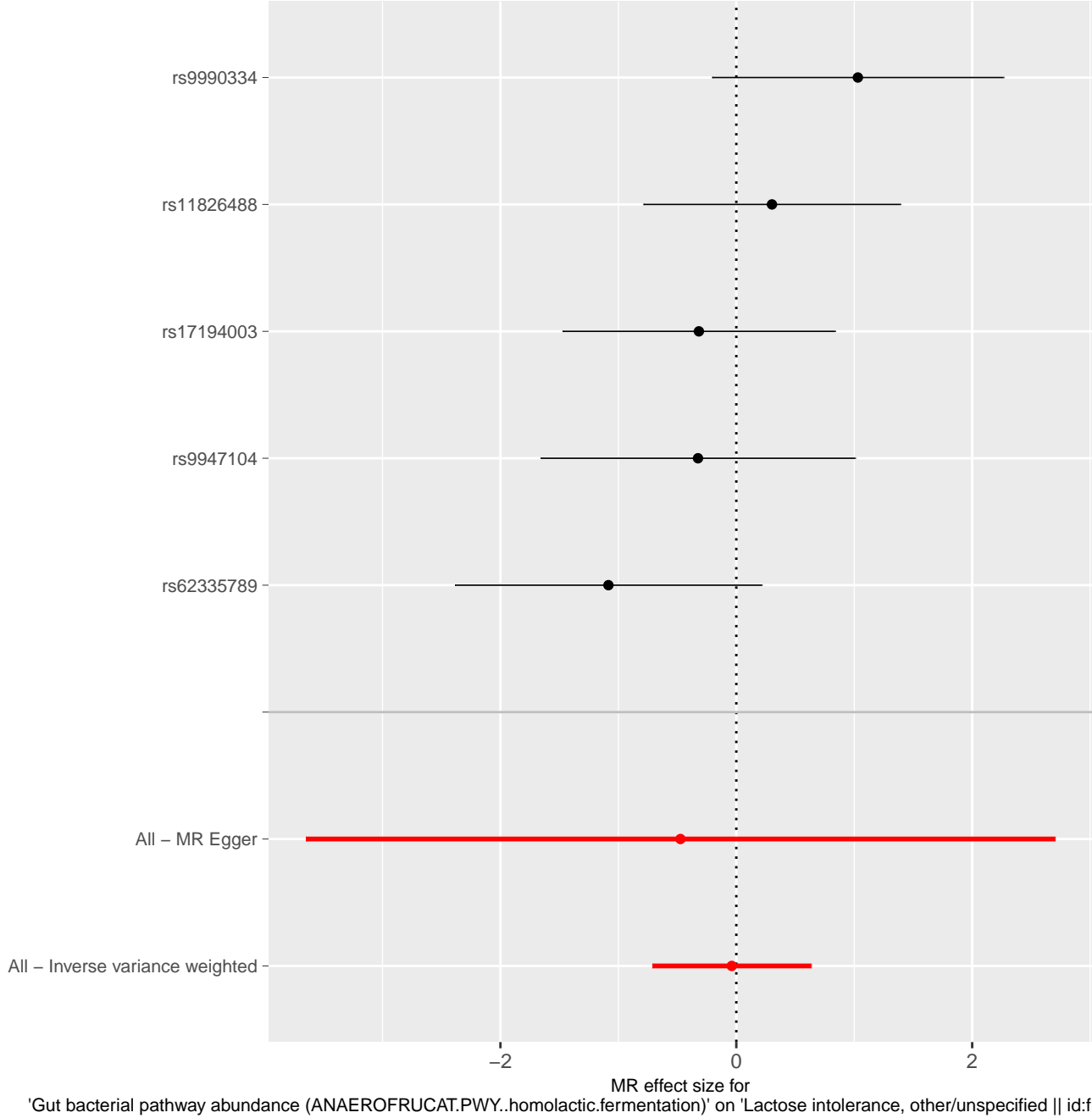

Supplement: Supplementary file 1 [file Data_Sheet_1.zip › supplementary materials/Forward/forest plot/ebi-a-GCST90027446.finngen_R12_E4_LACTONAS.pdf]

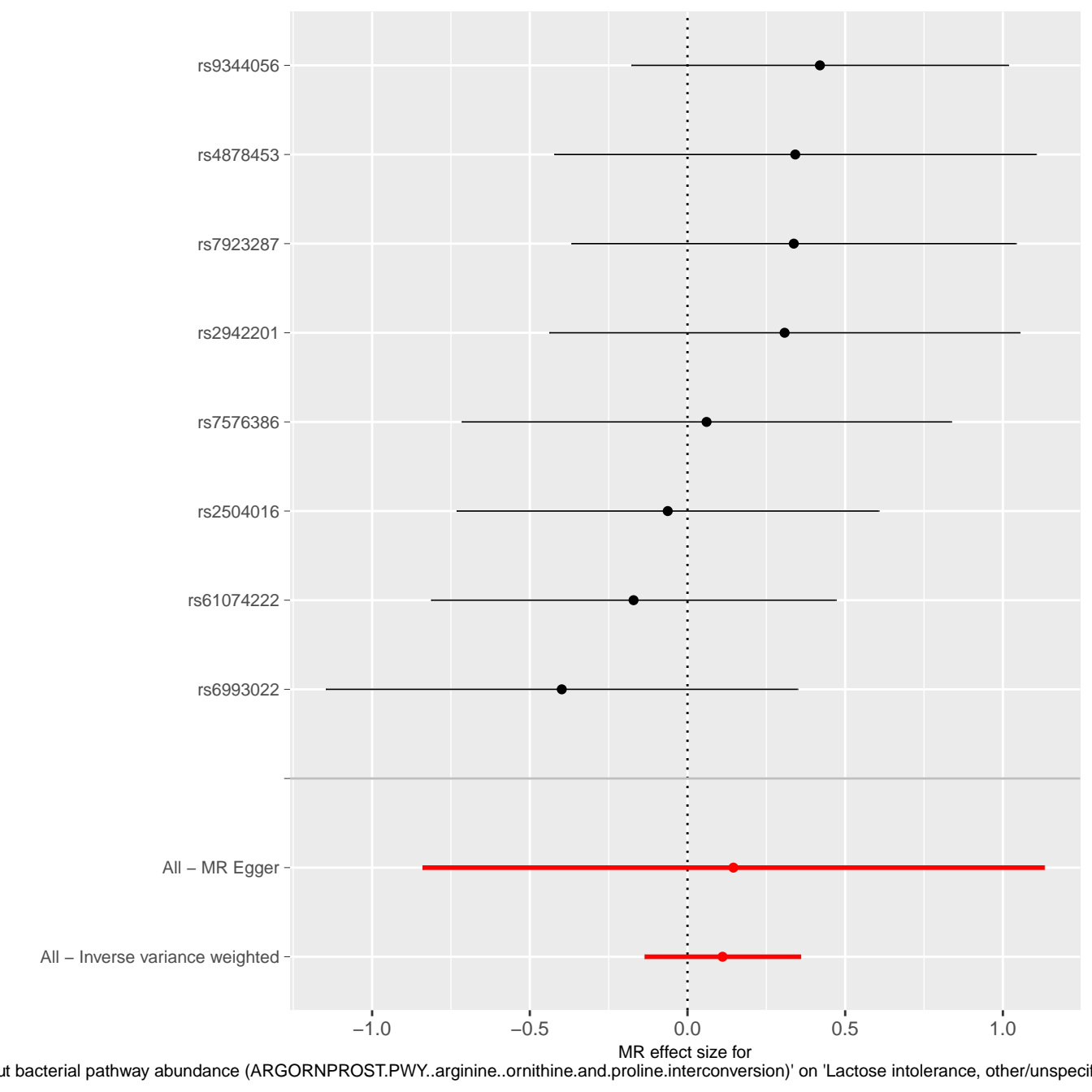

Supplement: Supplementary file 1 [file Data_Sheet_1.zip › supplementary materials/Forward/forest plot/ebi-a-GCST90027449.finngen_R12_E4_LACTONAS.pdf]

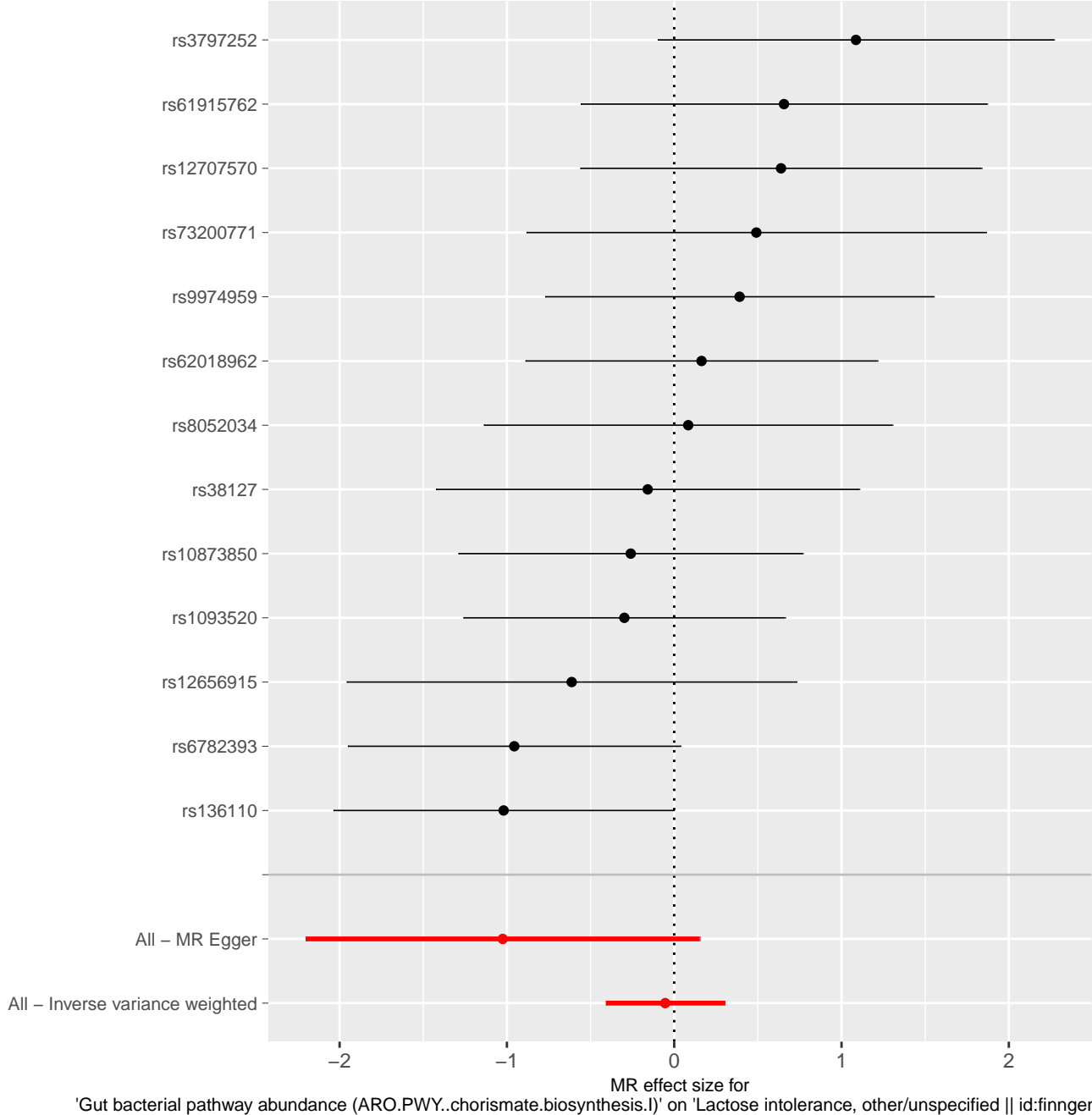

Supplement: Supplementary file 1 [file Data_Sheet_1.zip › supplementary materials/Forward/forest plot/ebi-a-GCST90027450.finngen_R12_E4_LACTONAS.pdf]

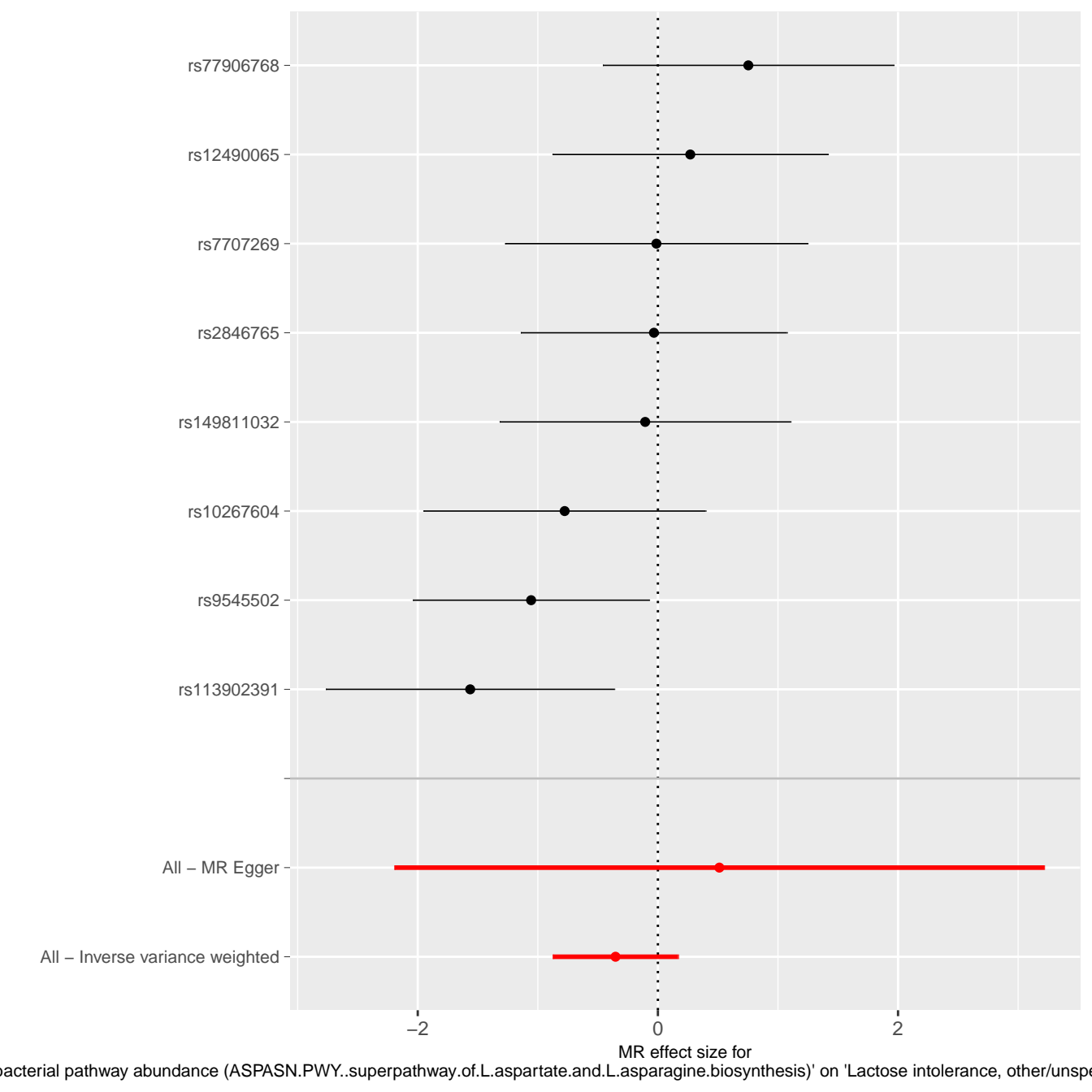

Supplement: Supplementary file 1 [file Data_Sheet_1.zip › supplementary materials/Forward/forest plot/ebi-a-GCST90027451.finngen_R12_E4_LACTONAS.pdf]

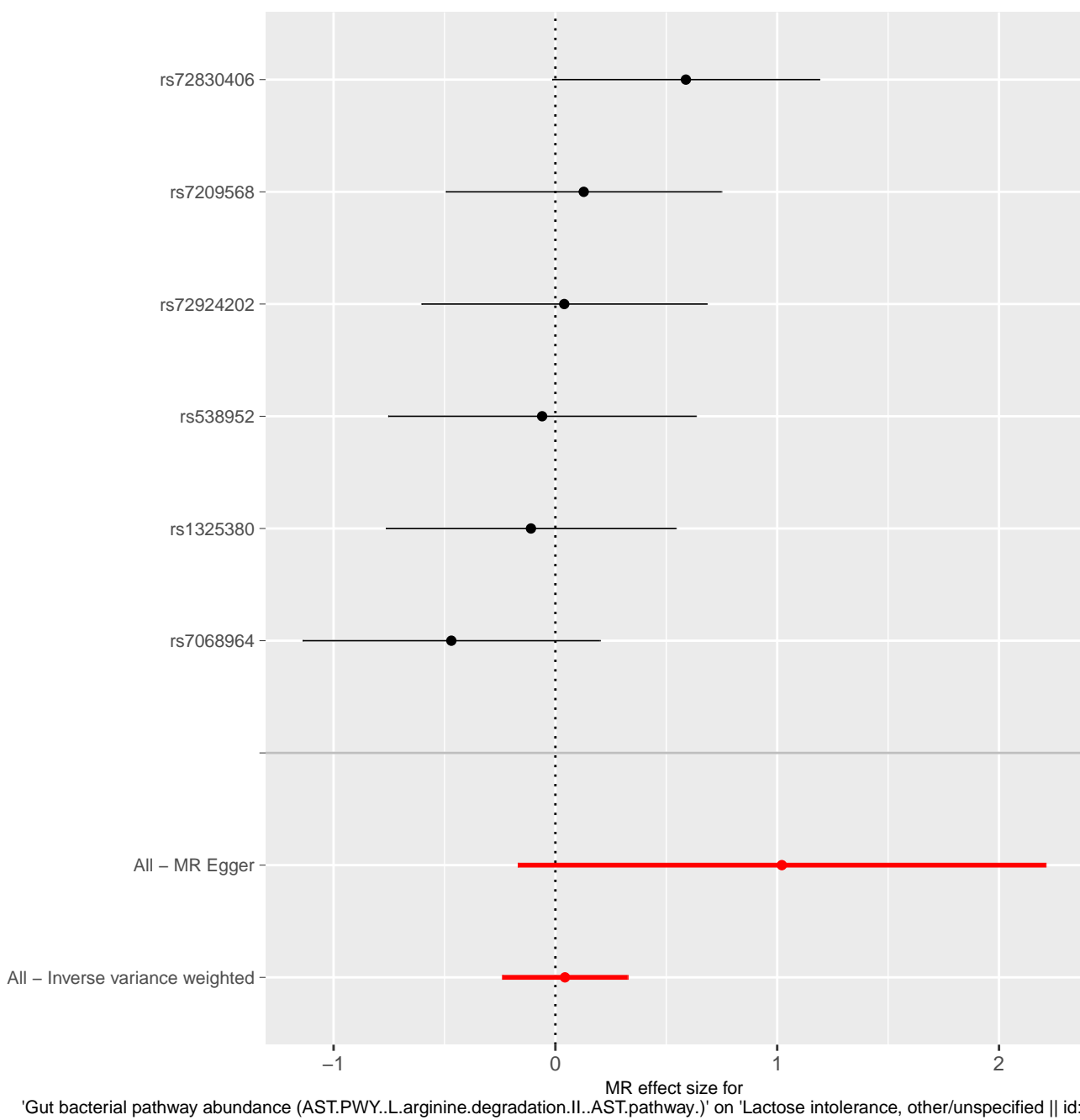

Supplement: Supplementary file 1 [file Data_Sheet_1.zip › supplementary materials/Forward/forest plot/ebi-a-GCST90027452.finngen_R12_E4_LACTONAS.pdf]

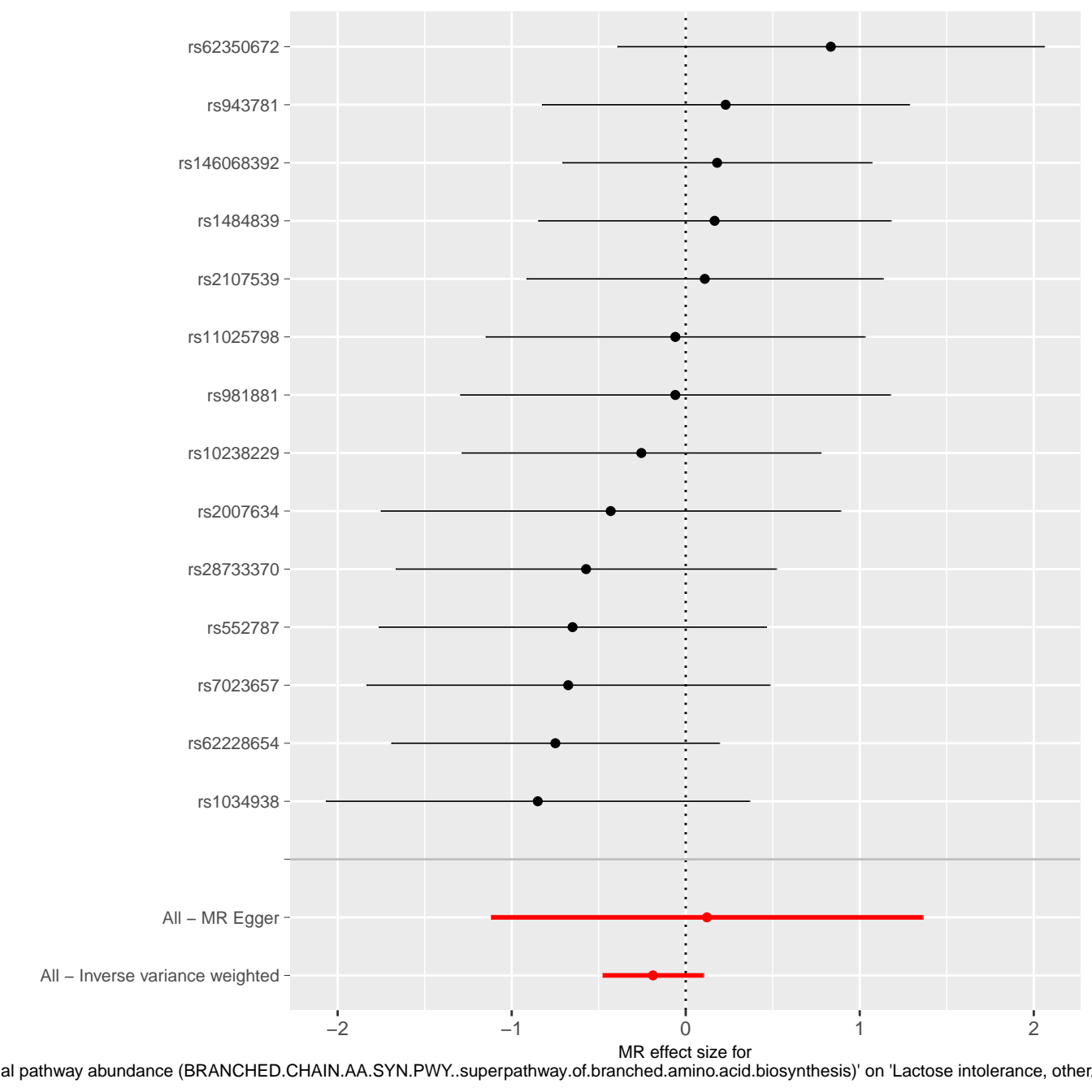

Supplement: Supplementary file 1 [file Data_Sheet_1.zip › supplementary materials/Forward/forest plot/ebi-a-GCST90027453.finngen_R12_E4_LACTONAS.pdf]

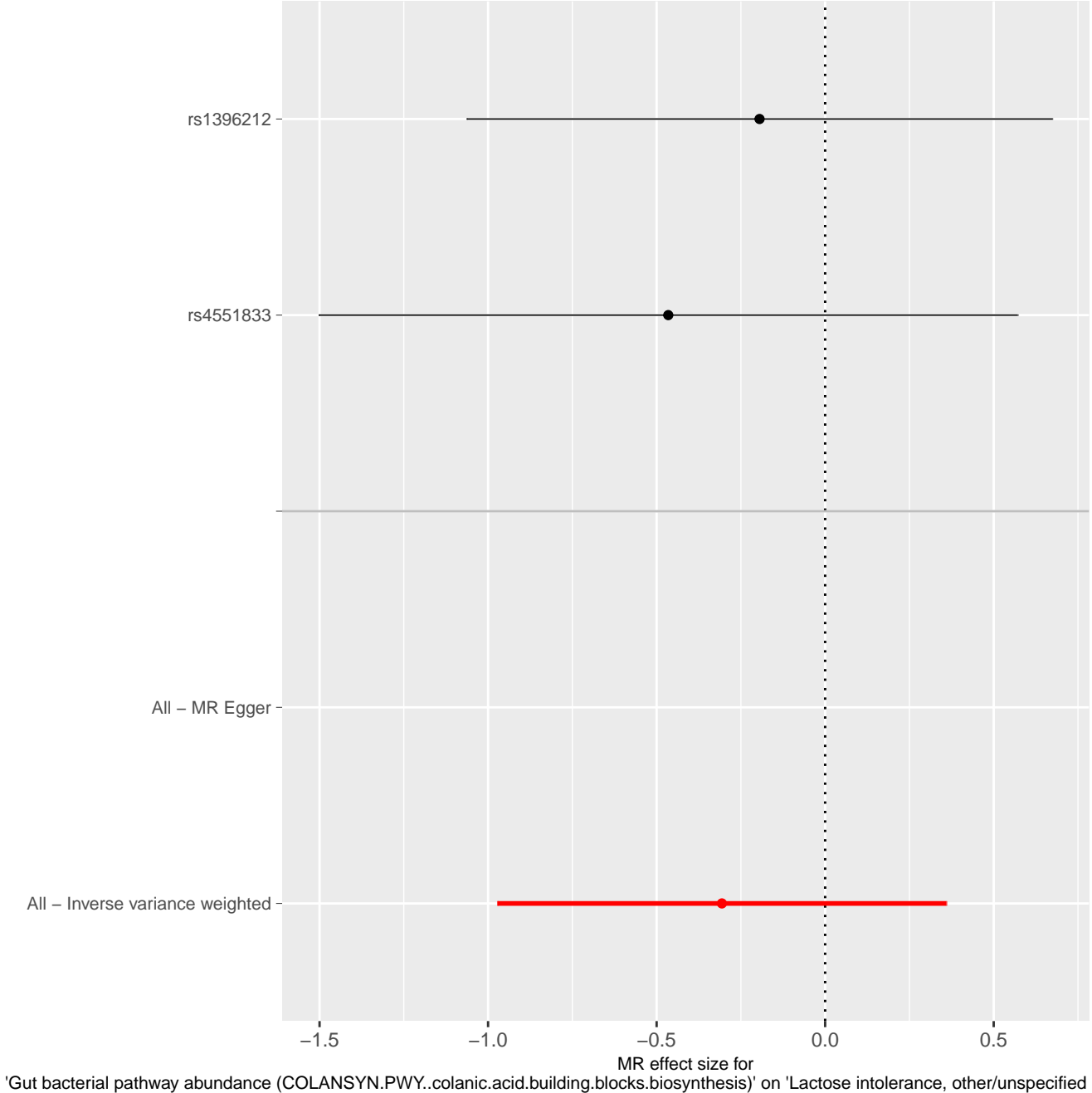

Supplement: Supplementary file 1 [file Data_Sheet_1.zip › supplementary materials/Forward/forest plot/ebi-a-GCST90027456.finngen_R12_E4_LACTONAS.pdf]

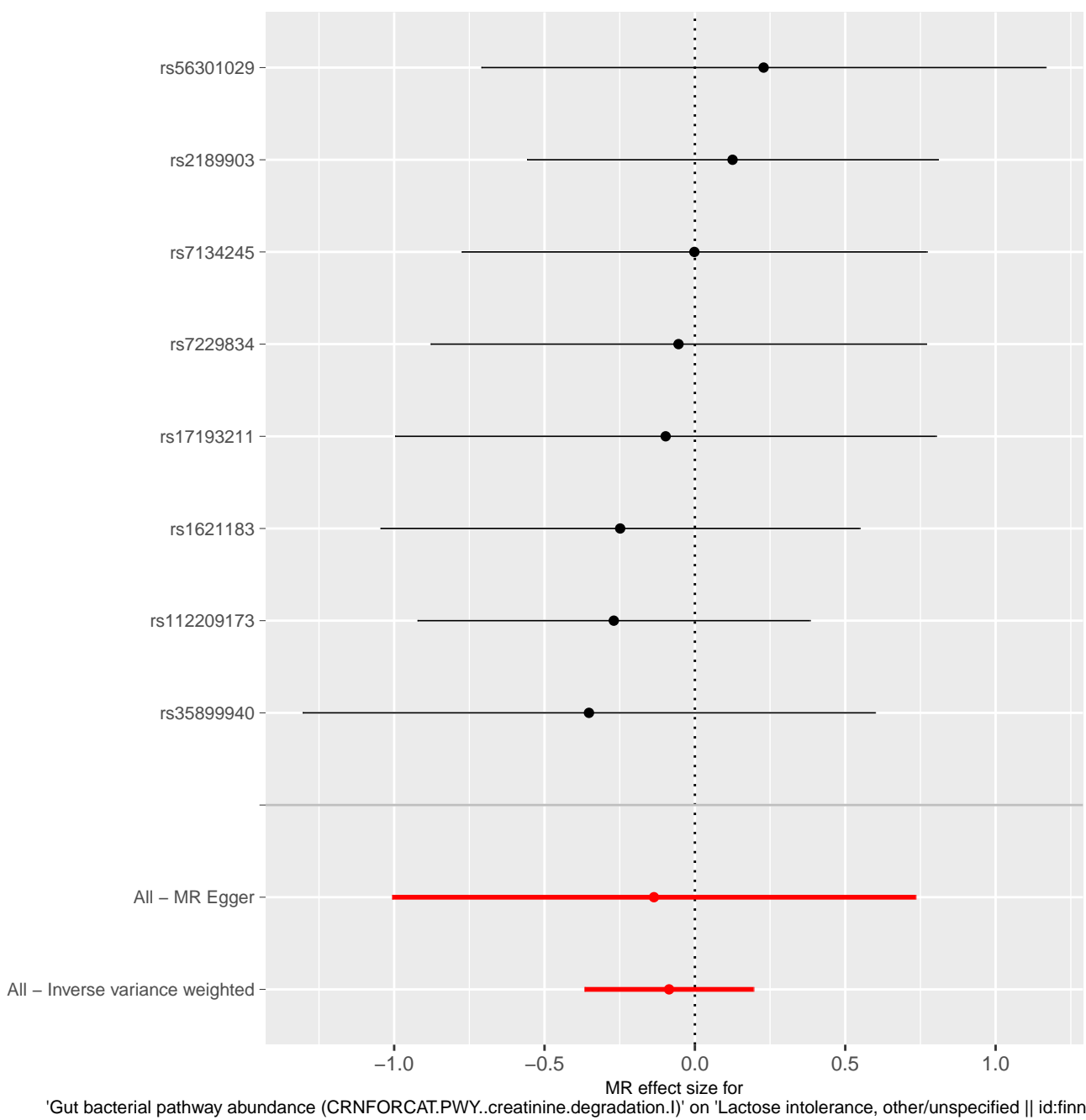

Supplement: Supplementary file 1 [file Data_Sheet_1.zip › supplementary materials/Forward/forest plot/ebi-a-GCST90027458.finngen_R12_E4_LACTONAS.pdf]

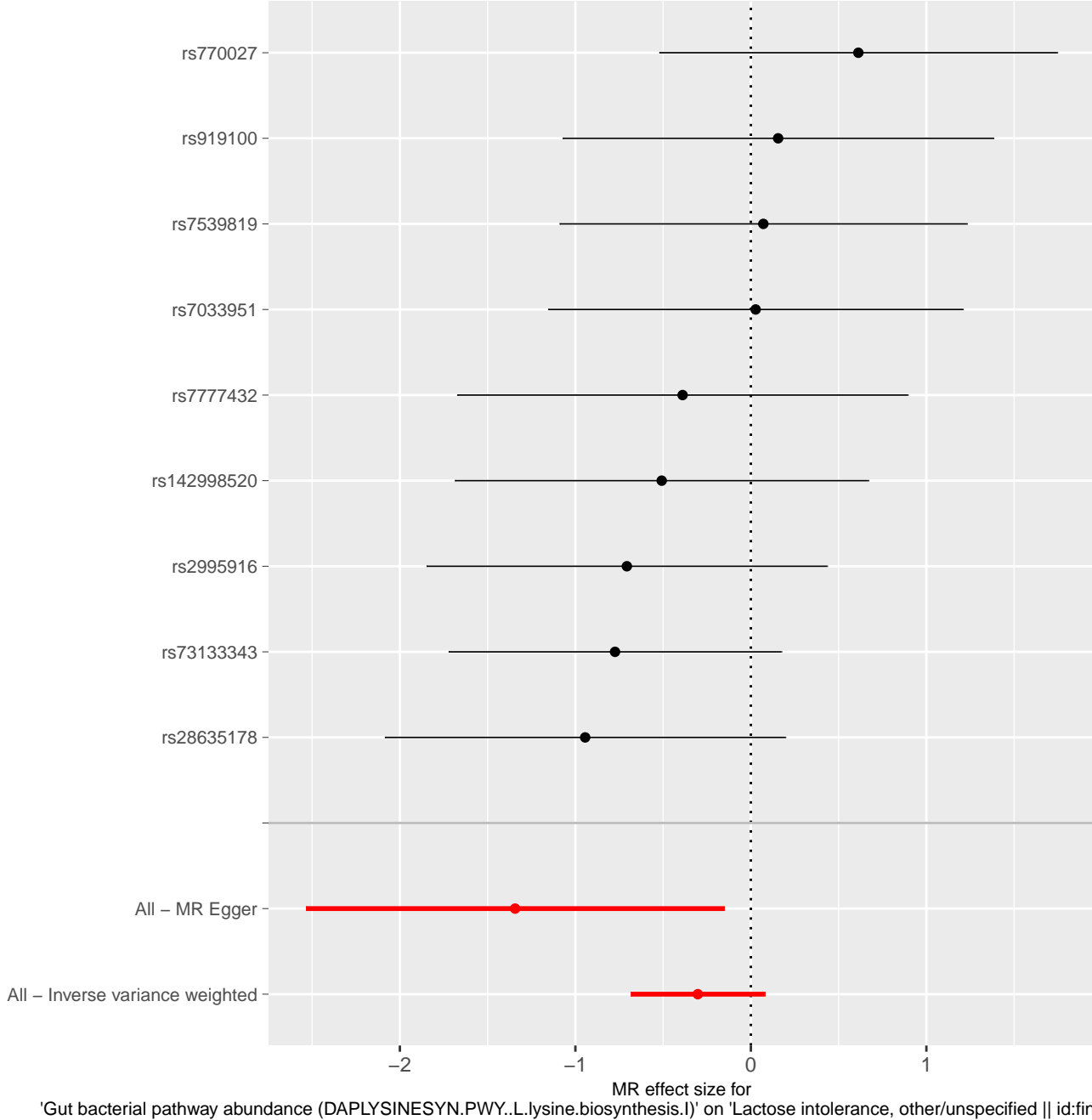

Supplement: Supplementary file 1 [file Data_Sheet_1.zip › supplementary materials/Forward/forest plot/ebi-a-GCST90027459.finngen_R12_E4_LACTONAS.pdf]

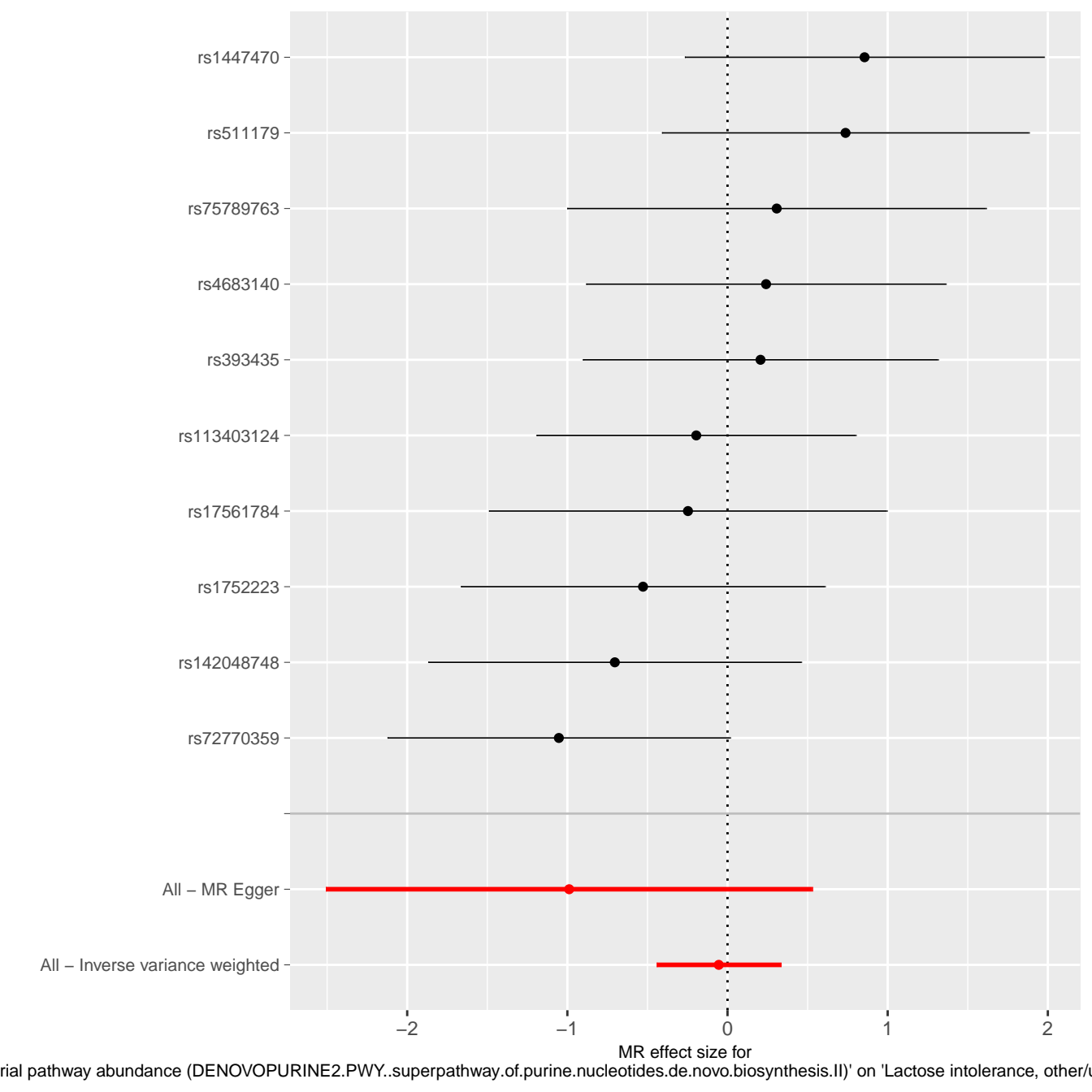

Supplement: Supplementary file 1 [file Data_Sheet_1.zip › supplementary materials/Forward/forest plot/ebi-a-GCST90027460.finngen_R12_E4_LACTONAS.pdf]

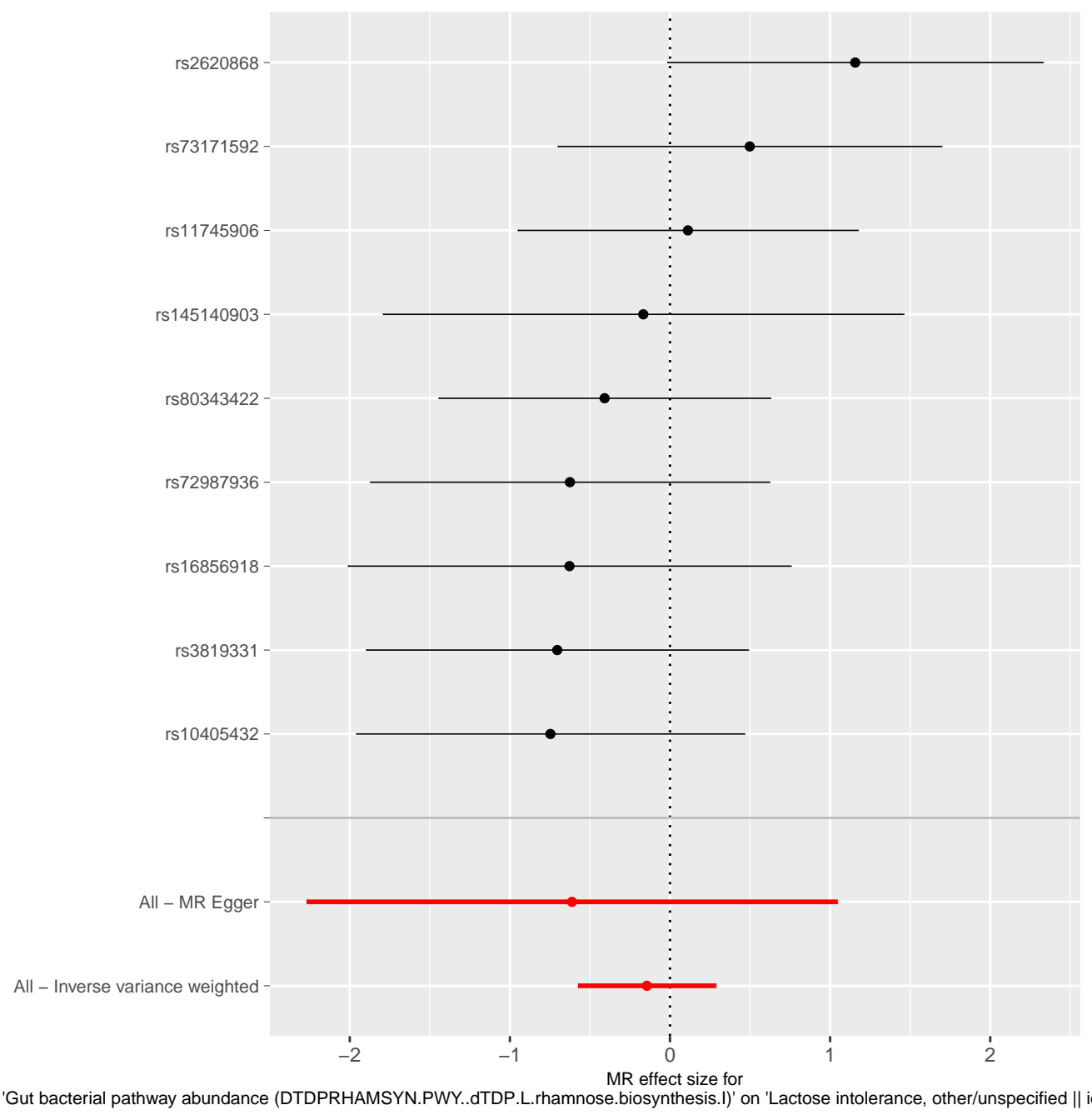

Supplement: Supplementary file 1 [file Data_Sheet_1.zip › supplementary materials/Forward/forest plot/ebi-a-GCST90027461.finngen_R12_E4_LACTONAS.pdf]

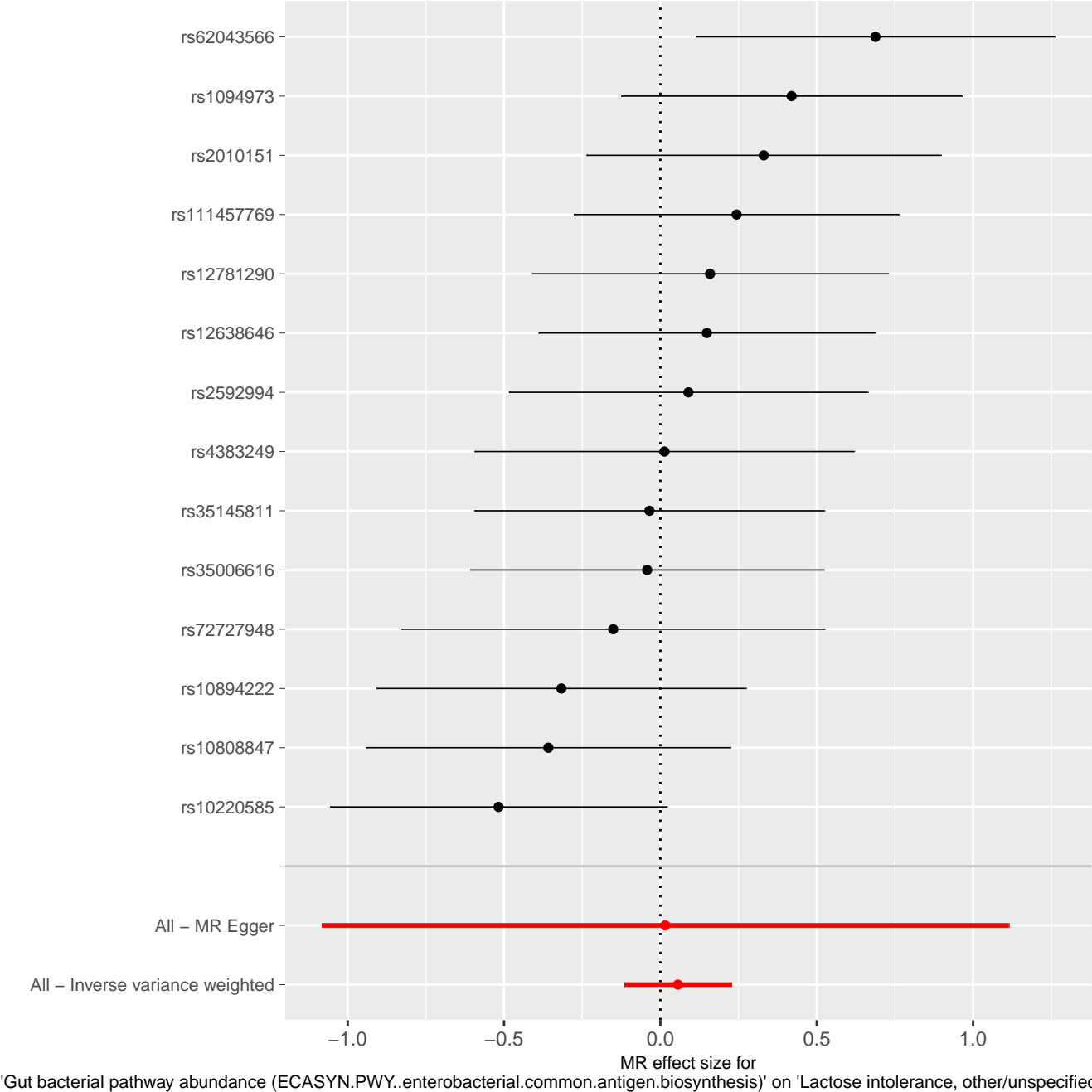

Supplement: Supplementary file 1 [file Data_Sheet_1.zip › supplementary materials/Forward/forest plot/ebi-a-GCST90027462.finngen_R12_E4_LACTONAS.pdf]

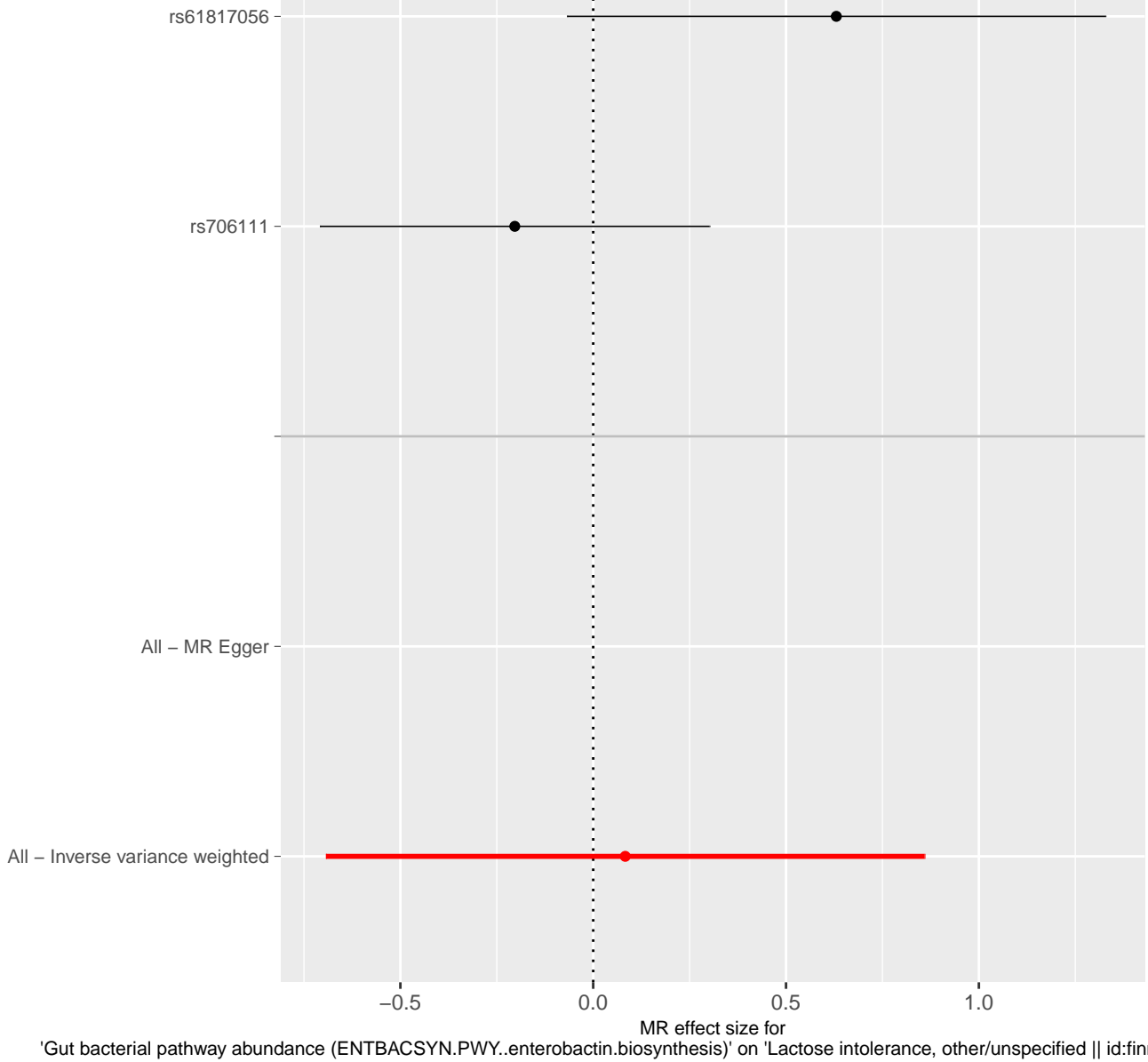

Supplement: Supplementary file 1 [file Data_Sheet_1.zip › supplementary materials/Forward/forest plot/ebi-a-GCST90027463.finngen_R12_E4_LACTONAS.pdf]

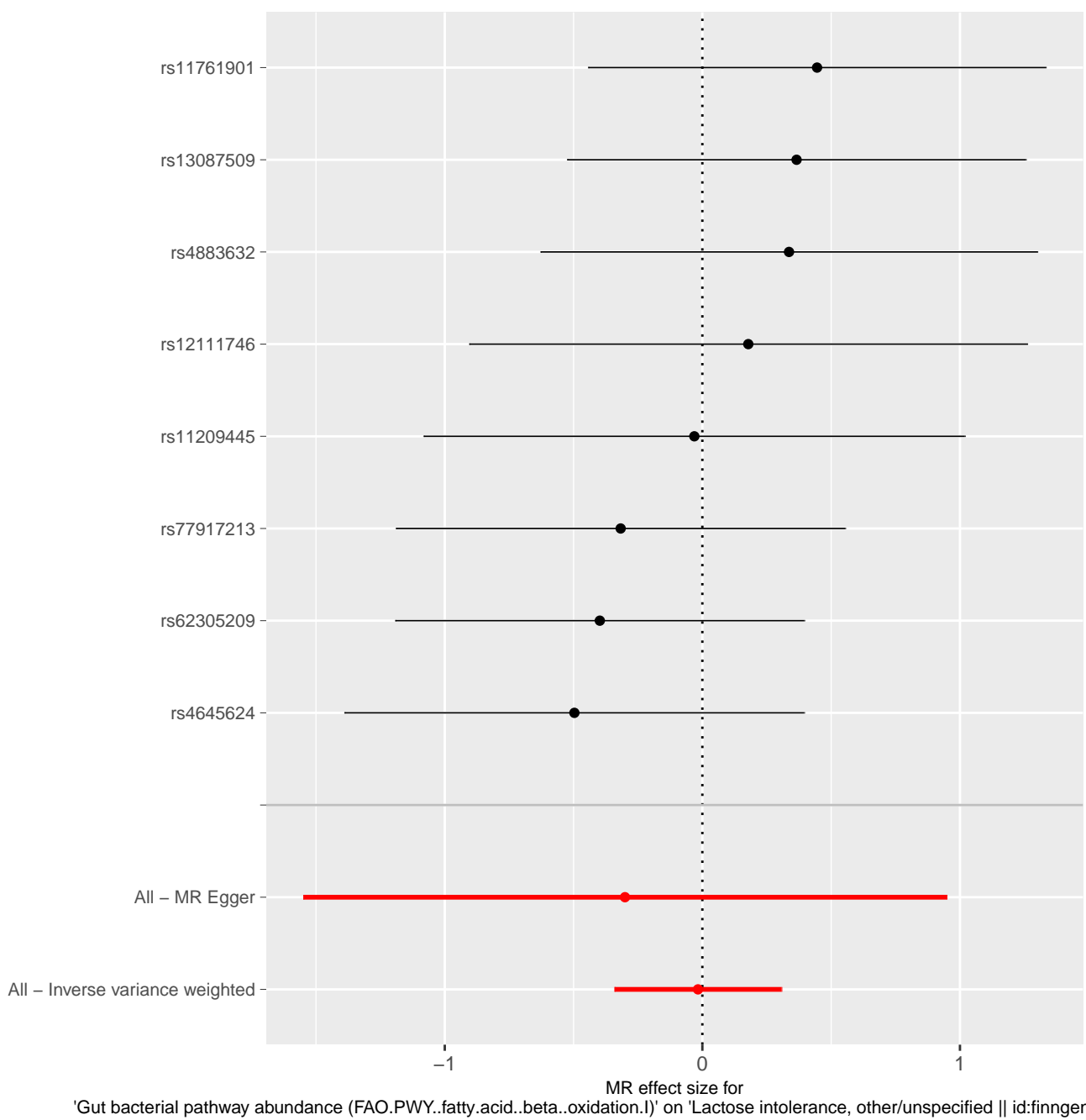

Supplement: Supplementary file 1 [file Data_Sheet_1.zip › supplementary materials/Forward/forest plot/ebi-a-GCST90027464.finngen_R12_E4_LACTONAS.pdf]

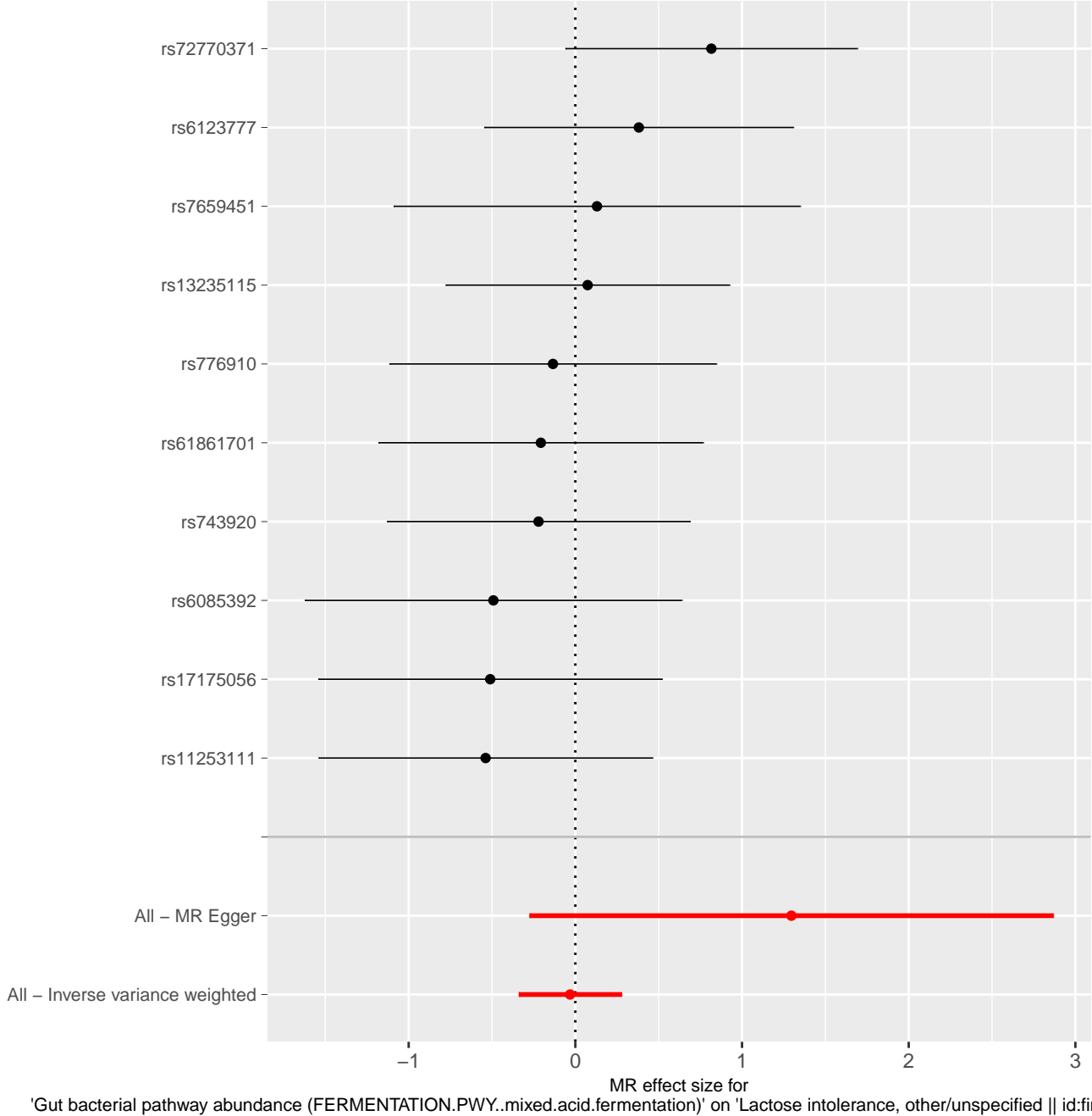

Supplement: Supplementary file 1 [file Data_Sheet_1.zip › supplementary materials/Forward/forest plot/ebi-a-GCST90027465.finngen_R12_E4_LACTONAS.pdf]

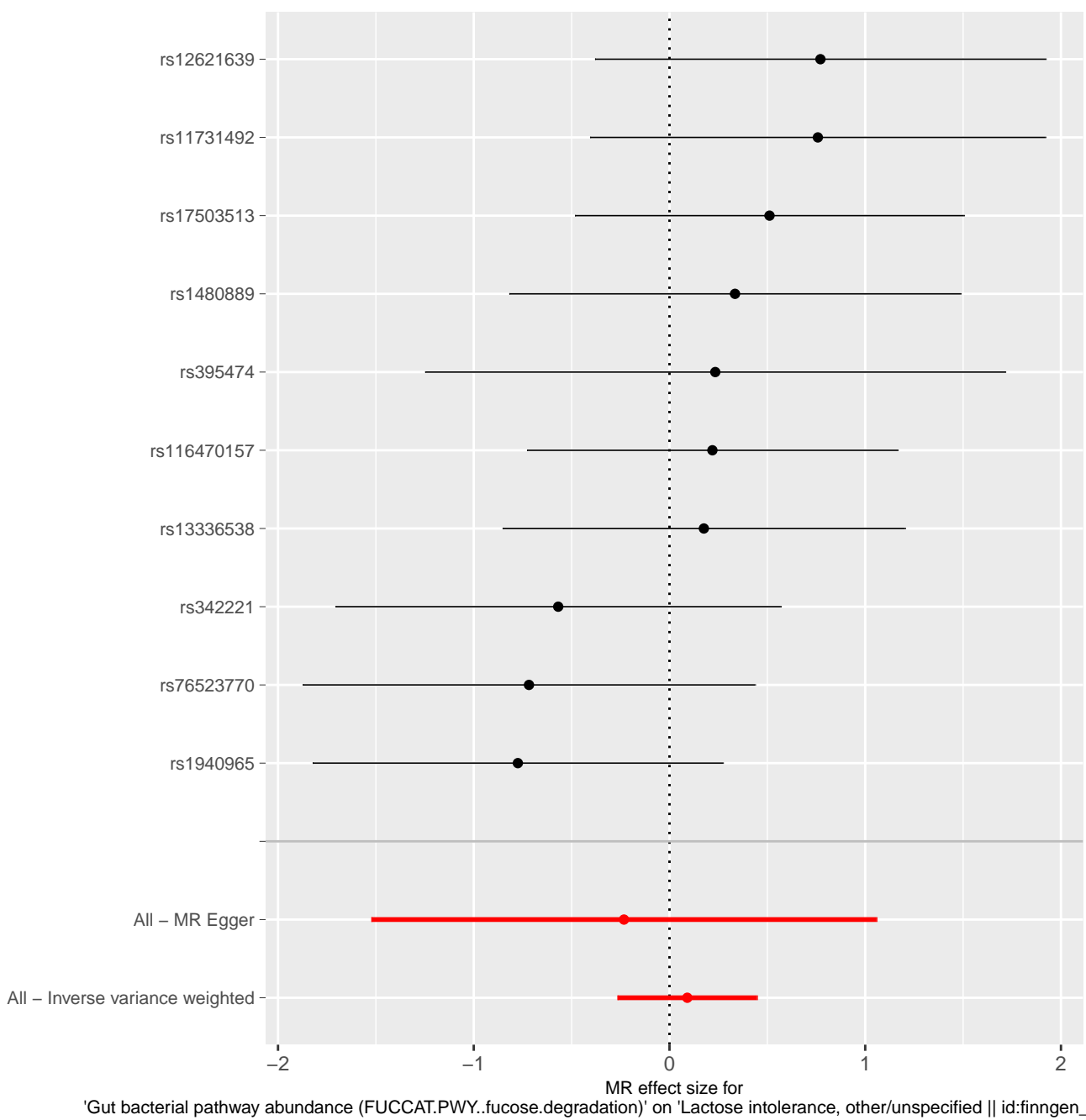

Supplement: Supplementary file 1 [file Data_Sheet_1.zip › supplementary materials/Forward/forest plot/ebi-a-GCST90027466.finngen_R12_E4_LACTONAS.pdf]

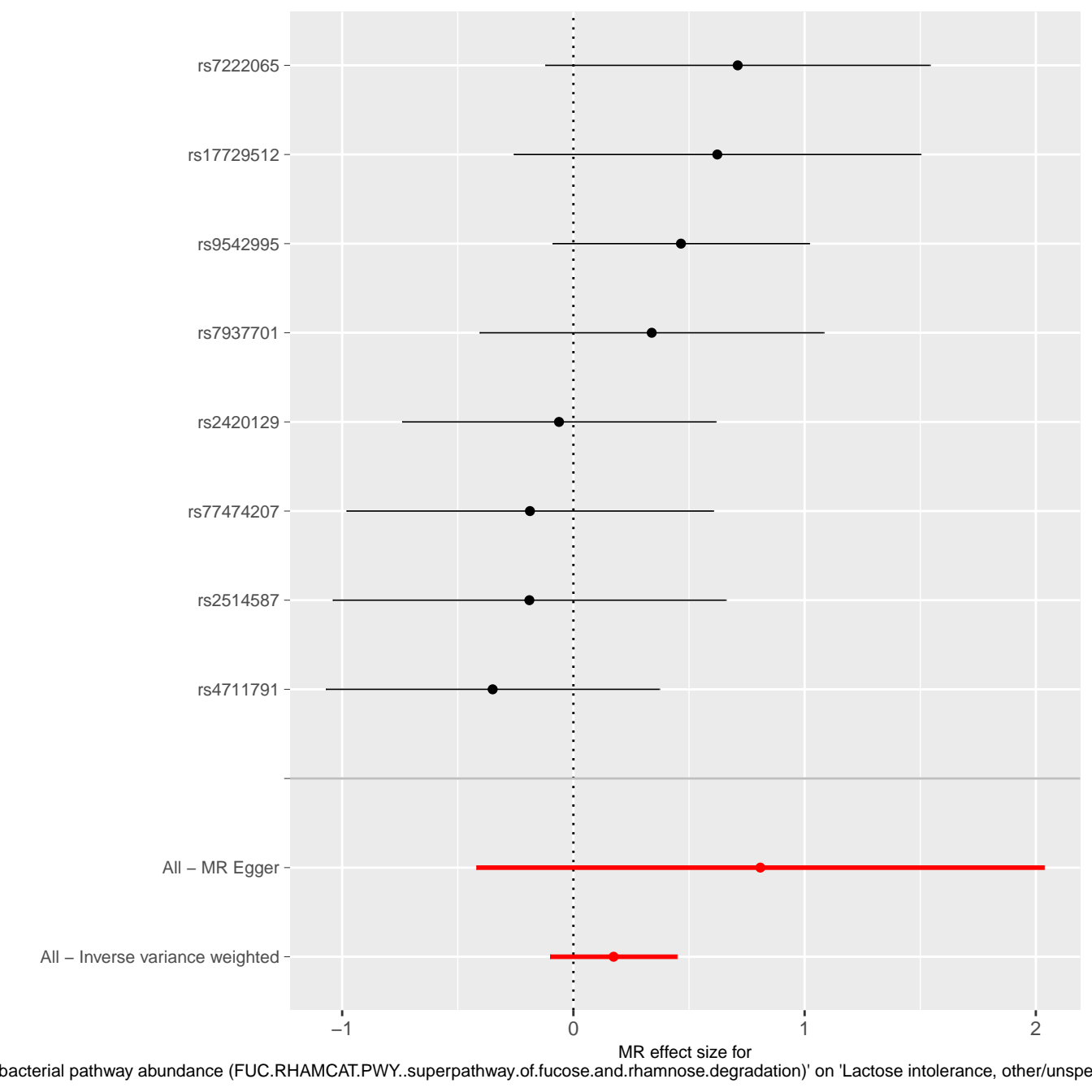

Supplement: Supplementary file 1 [file Data_Sheet_1.zip › supplementary materials/Forward/forest plot/ebi-a-GCST90027467.finngen_R12_E4_LACTONAS.pdf]

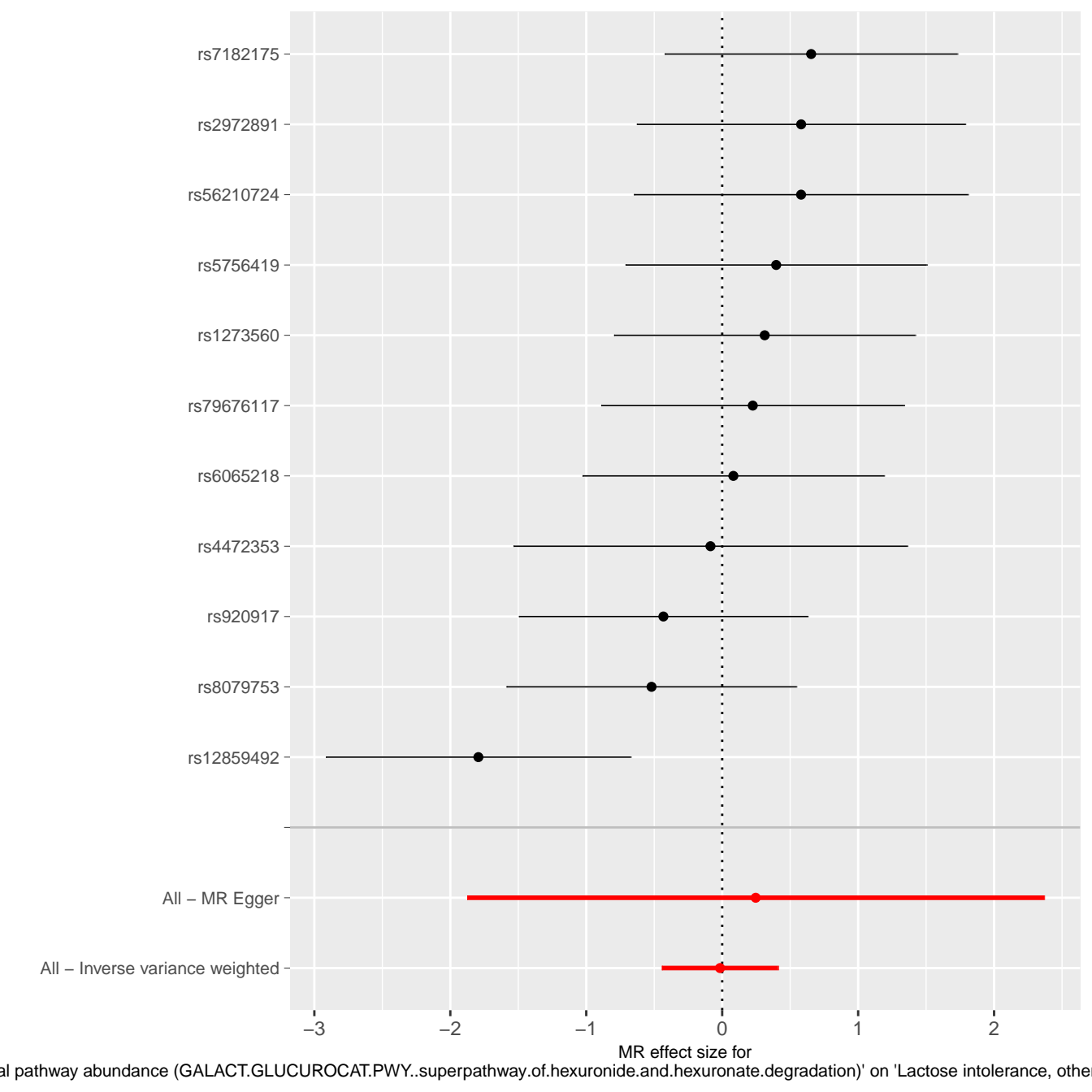

Supplement: Supplementary file 1 [file Data_Sheet_1.zip › supplementary materials/Forward/forest plot/ebi-a-GCST90027469.finngen_R12_E4_LACTONAS.pdf]

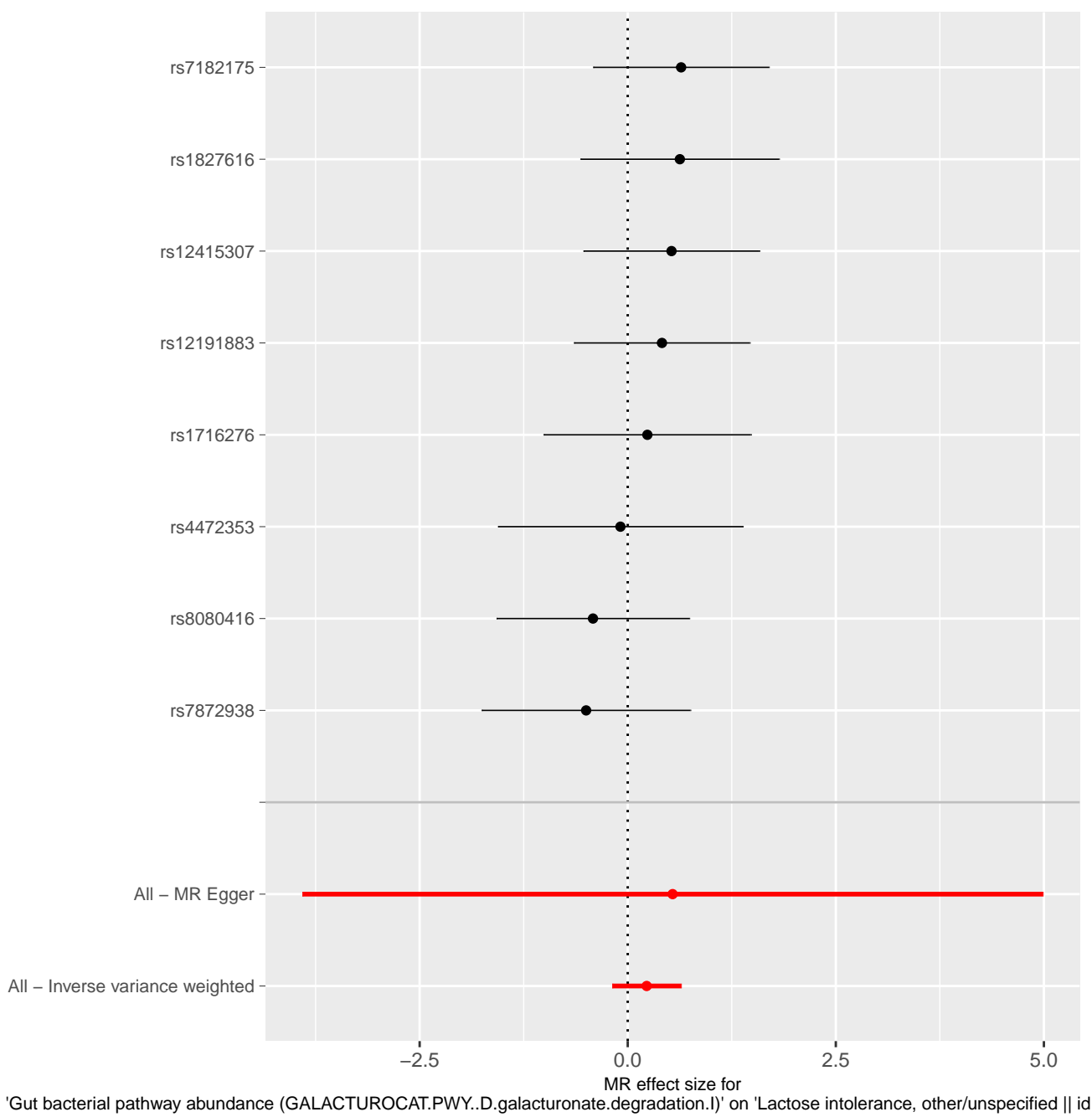

Supplement: Supplementary file 1 [file Data_Sheet_1.zip › supplementary materials/Forward/forest plot/ebi-a-GCST90027470.finngen_R12_E4_LACTONAS.pdf]

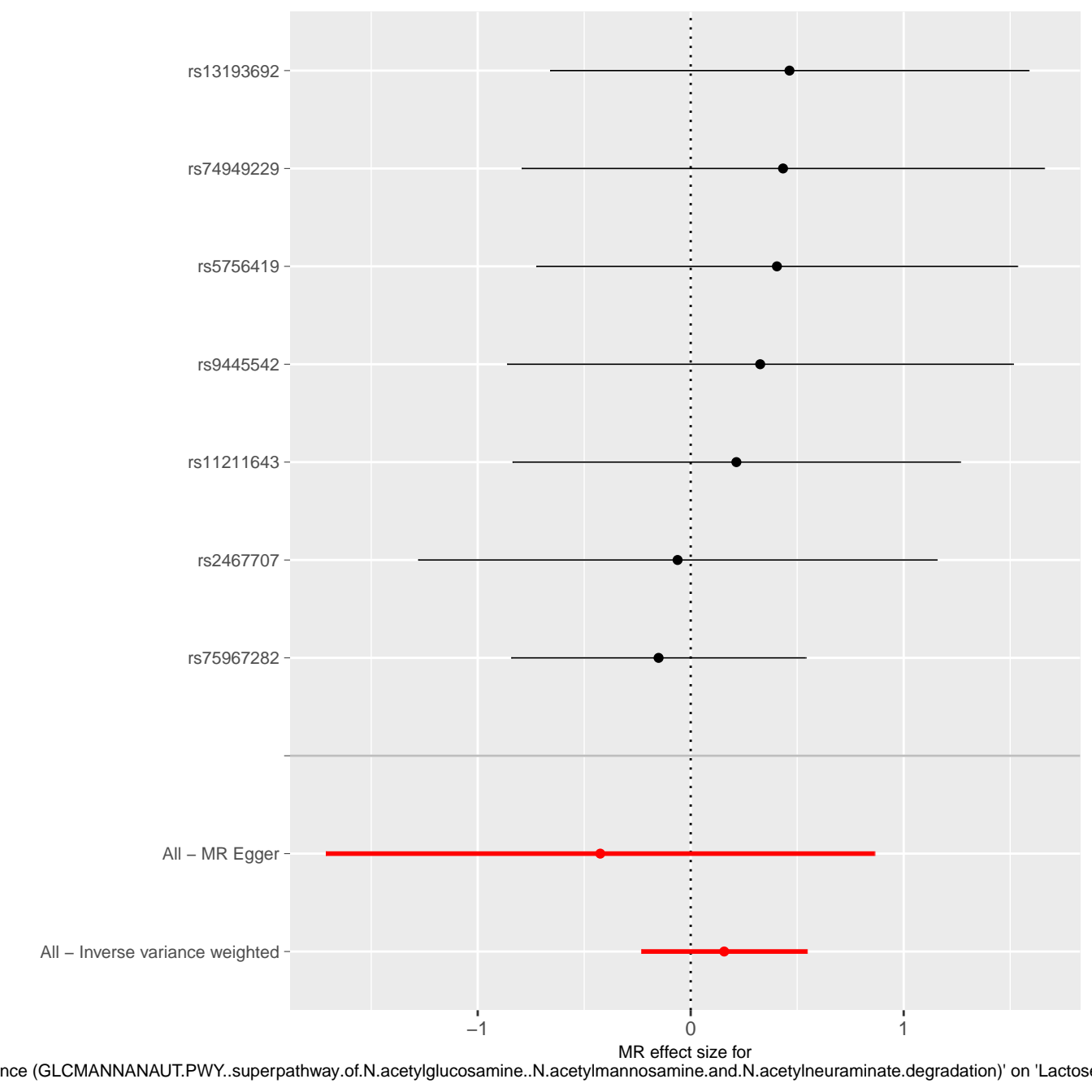

Supplement: Supplementary file 1 [file Data_Sheet_1.zip › supplementary materials/Forward/forest plot/ebi-a-GCST90027471.finngen_R12_E4_LACTONAS.pdf]

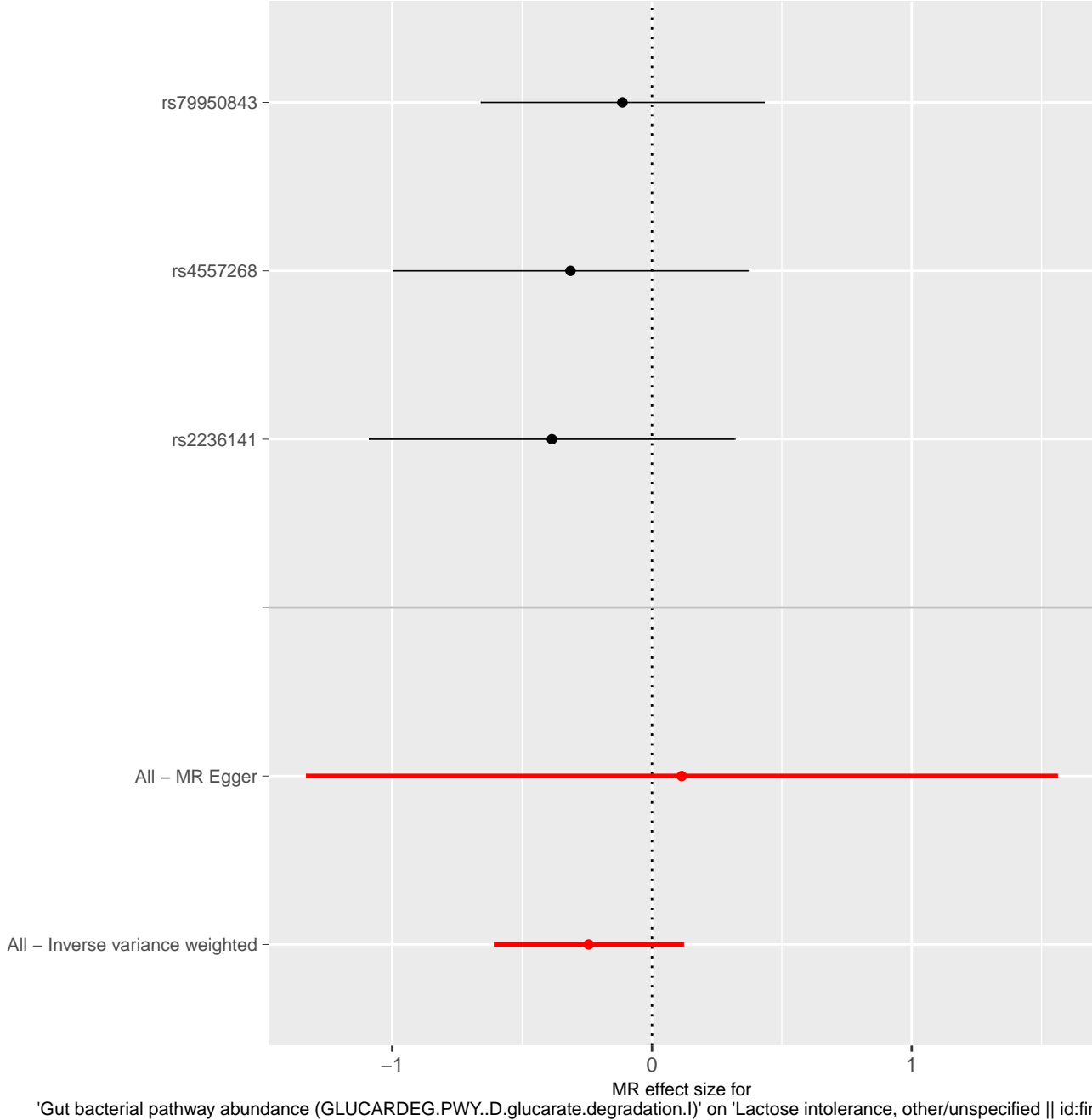

Supplement: Supplementary file 1 [file Data_Sheet_1.zip › supplementary materials/Forward/forest plot/ebi-a-GCST90027472.finngen_R12_E4_LACTONAS.pdf]

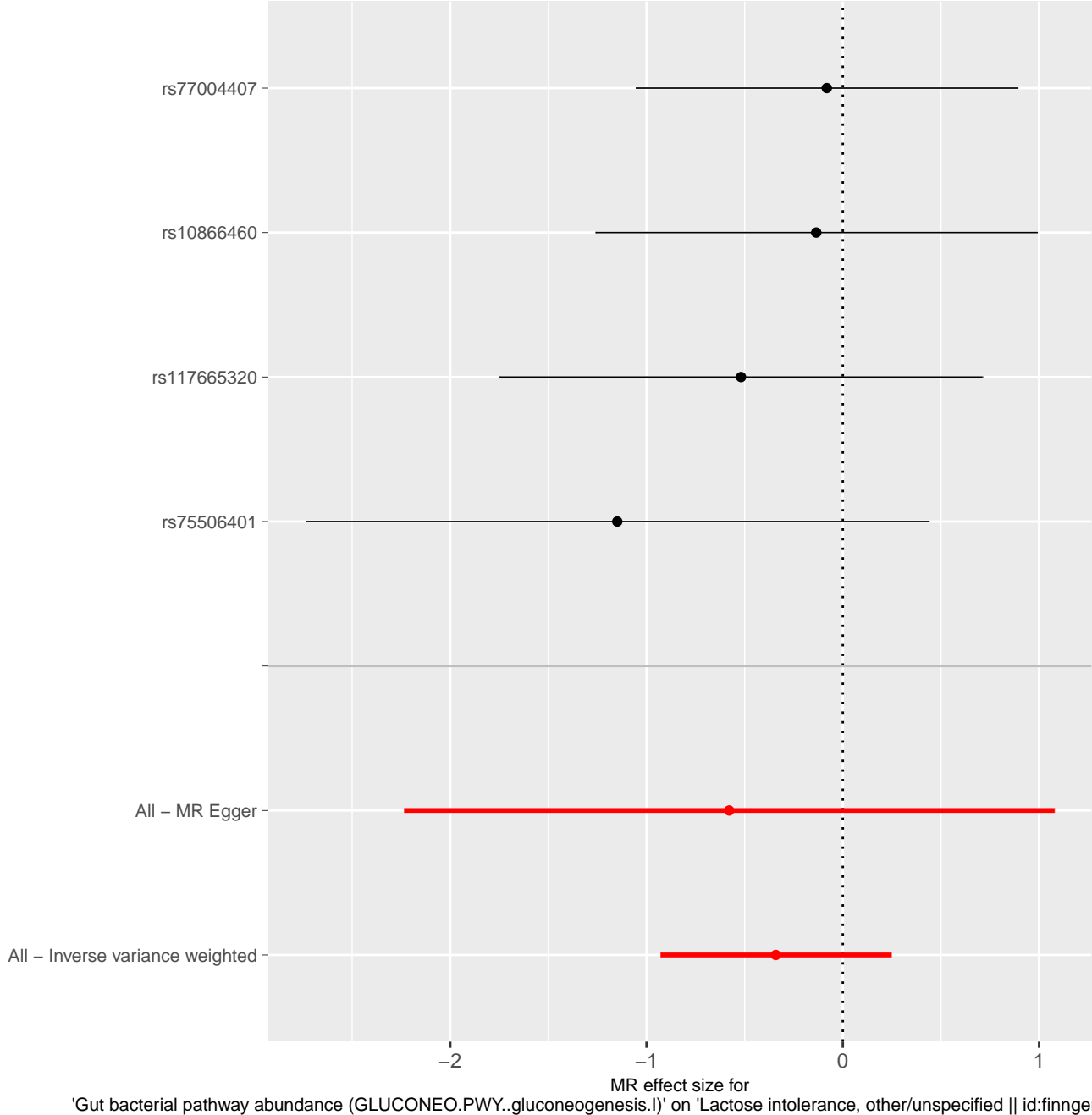

Supplement: Supplementary file 1 [file Data_Sheet_1.zip › supplementary materials/Forward/forest plot/ebi-a-GCST90027473.finngen_R12_E4_LACTONAS.pdf]

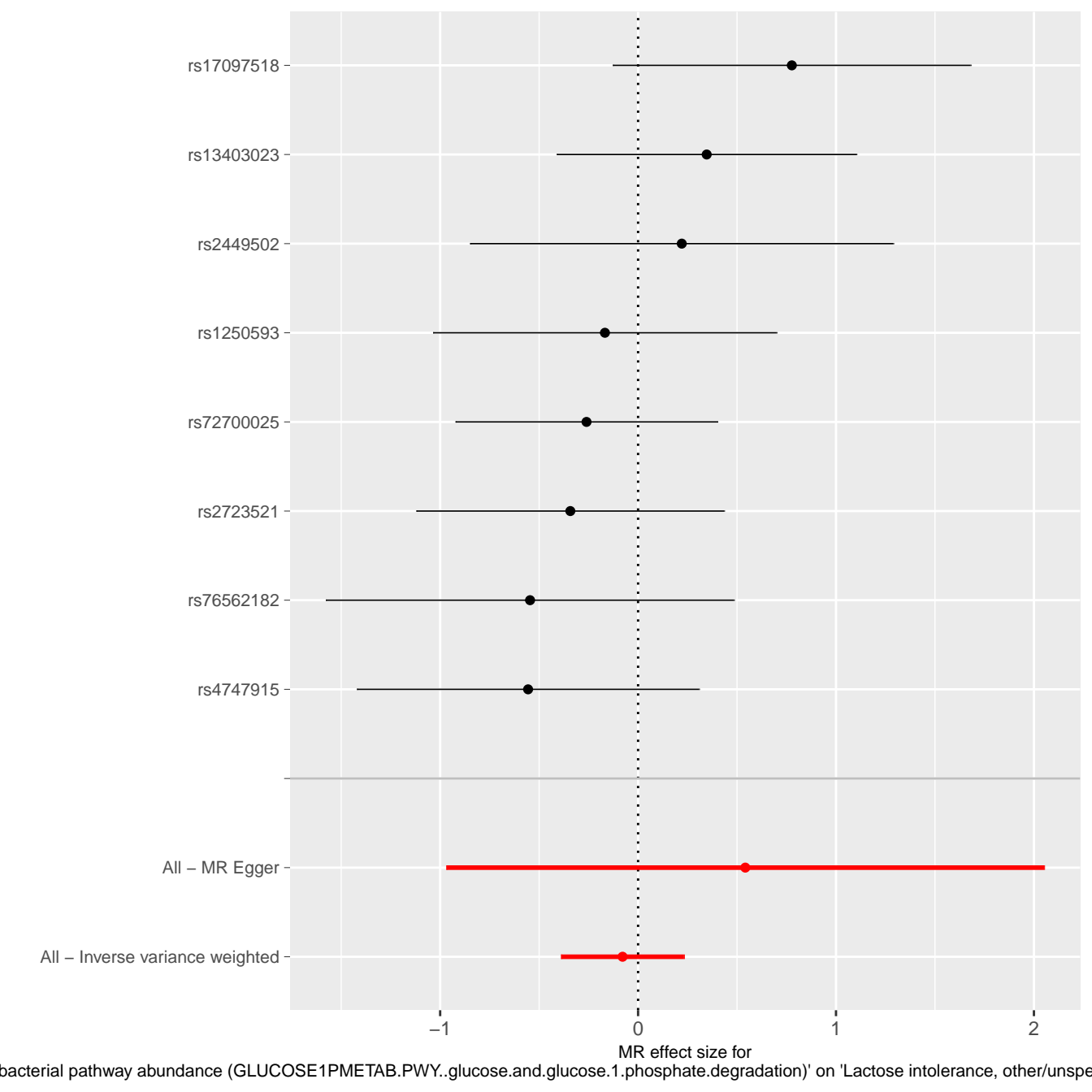

Supplement: Supplementary file 1 [file Data_Sheet_1.zip › supplementary materials/Forward/forest plot/ebi-a-GCST90027474.finngen_R12_E4_LACTONAS.pdf]

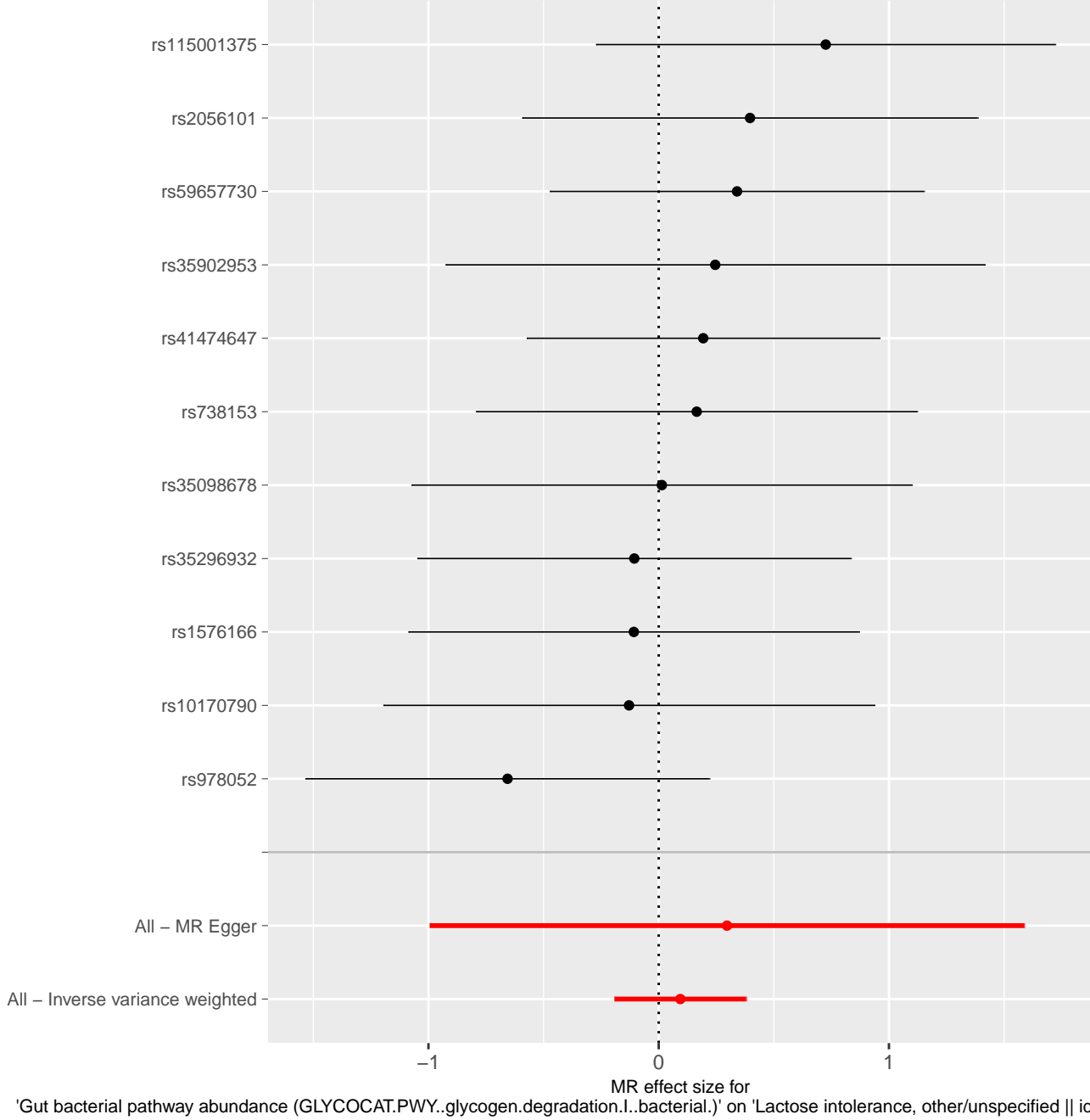

Supplement: Supplementary file 1 [file Data_Sheet_1.zip › supplementary materials/Forward/forest plot/ebi-a-GCST90027475.finngen_R12_E4_LACTONAS.pdf]

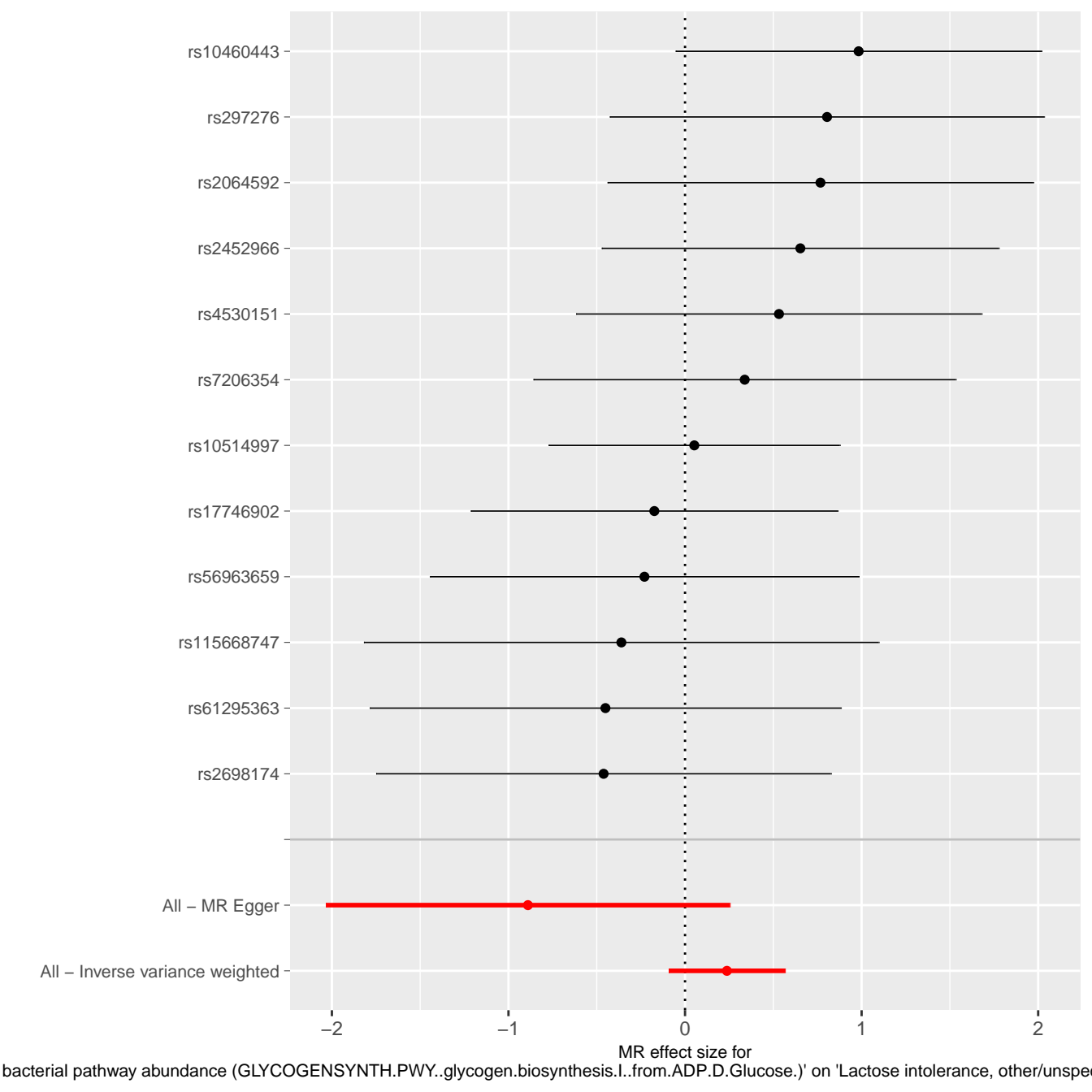

Supplement: Supplementary file 1 [file Data_Sheet_1.zip › supplementary materials/Forward/forest plot/ebi-a-GCST90027476.finngen_R12_E4_LACTONAS.pdf]

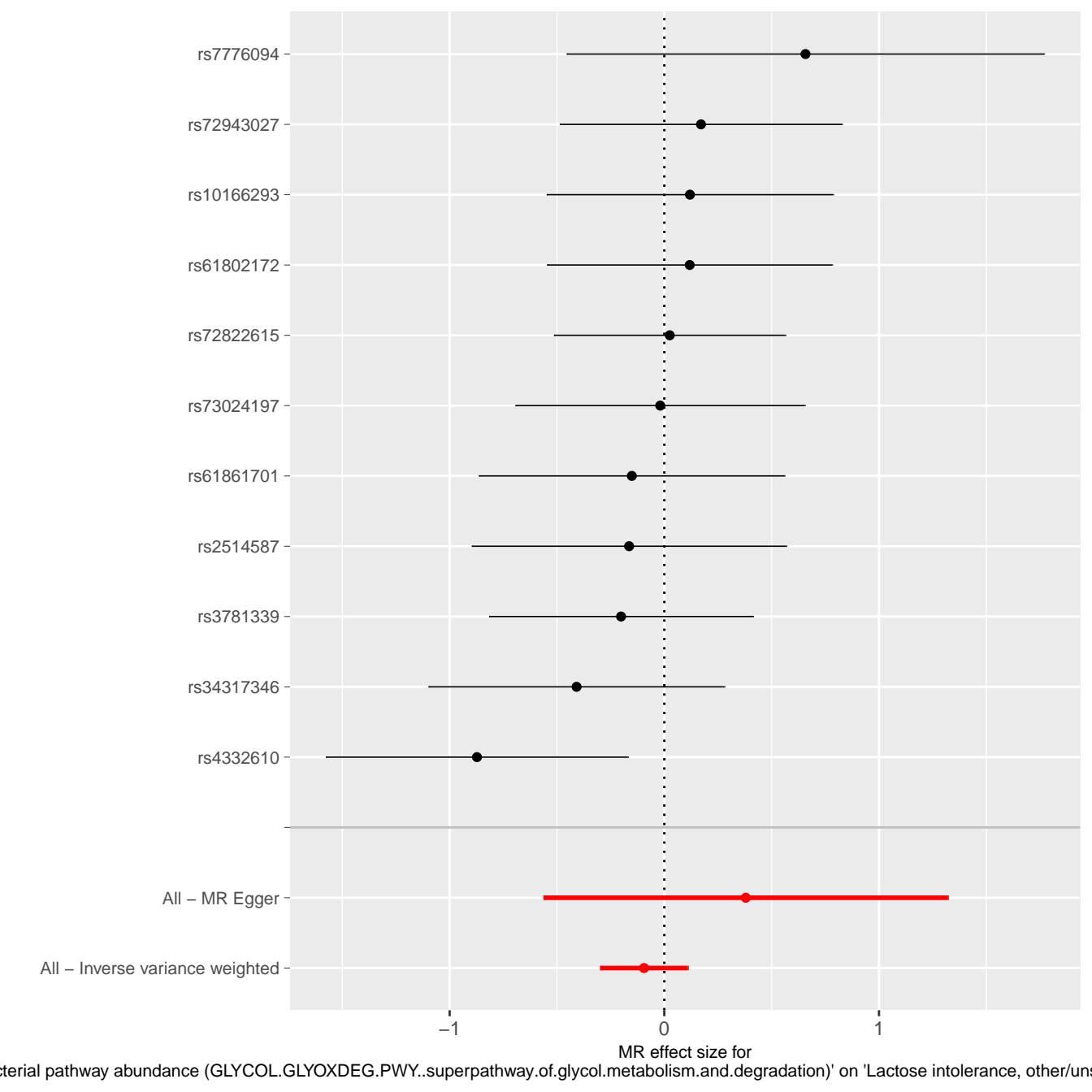

Supplement: Supplementary file 1 [file Data_Sheet_1.zip › supplementary materials/Forward/forest plot/ebi-a-GCST90027477.finngen_R12_E4_LACTONAS.pdf]

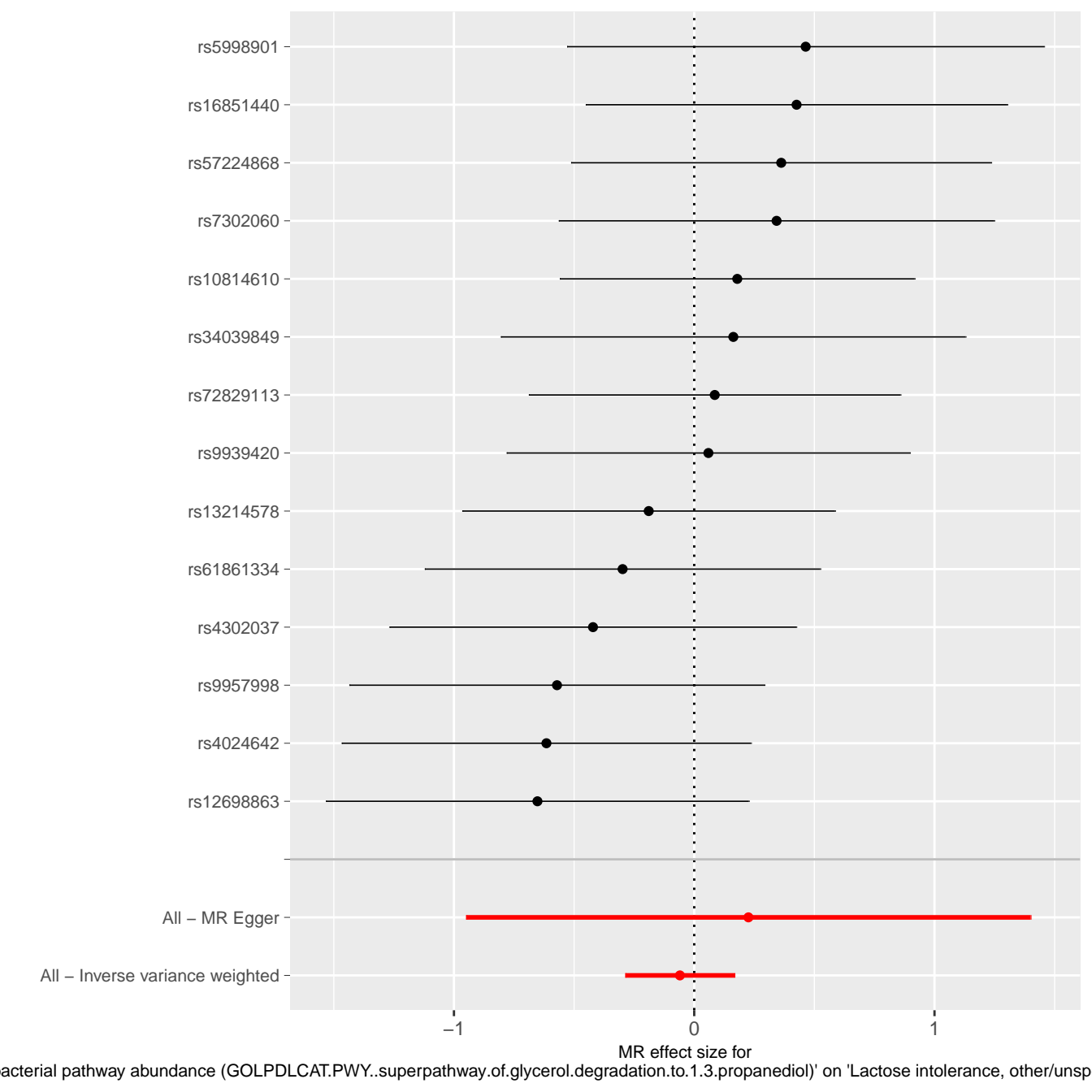

Supplement: Supplementary file 1 [file Data_Sheet_1.zip › supplementary materials/Forward/forest plot/ebi-a-GCST90027478.finngen_R12_E4_LACTONAS.pdf]

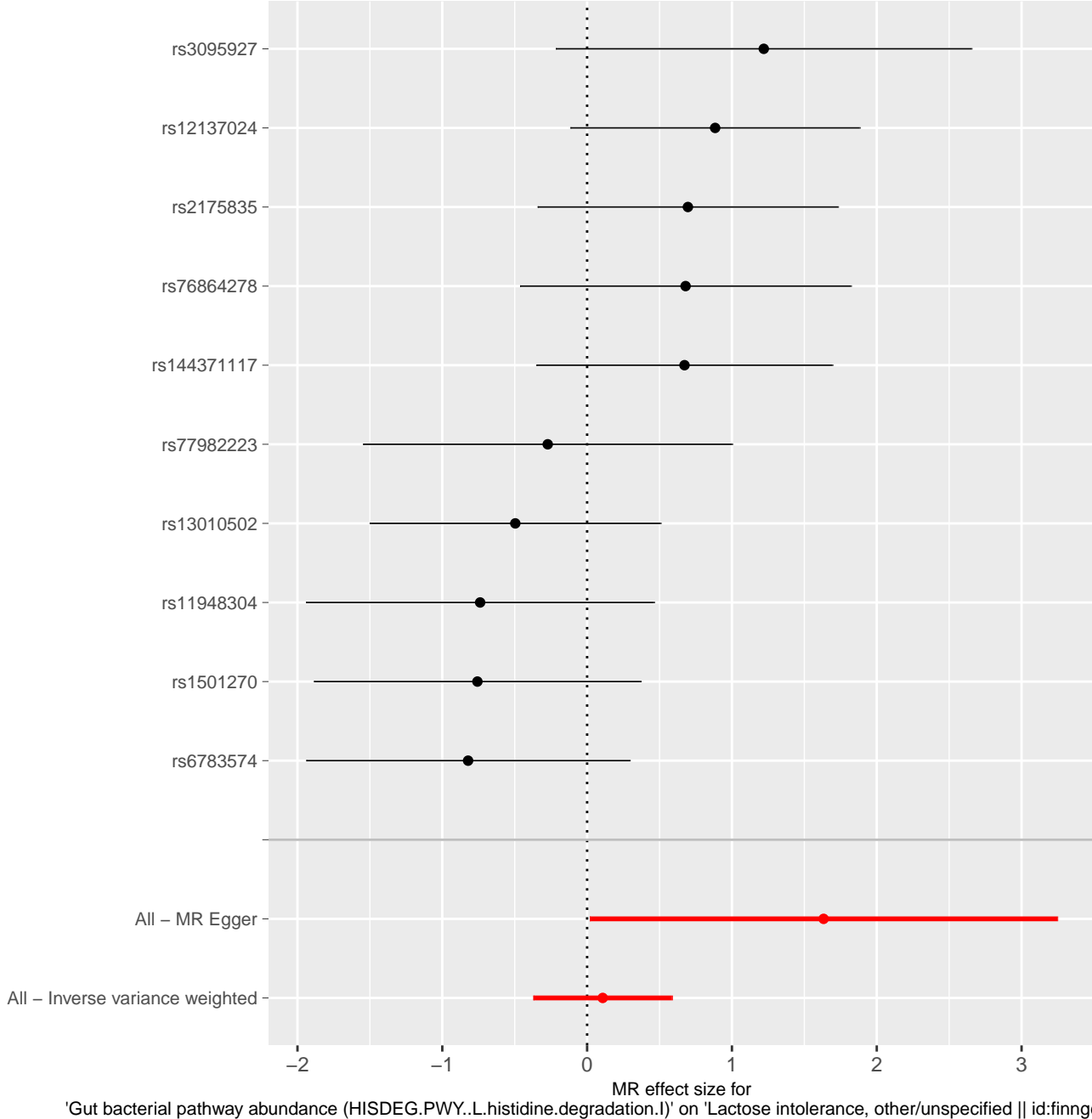

Supplement: Supplementary file 1 [file Data_Sheet_1.zip › supplementary materials/Forward/forest plot/ebi-a-GCST90027481.finngen_R12_E4_LACTONAS.pdf]

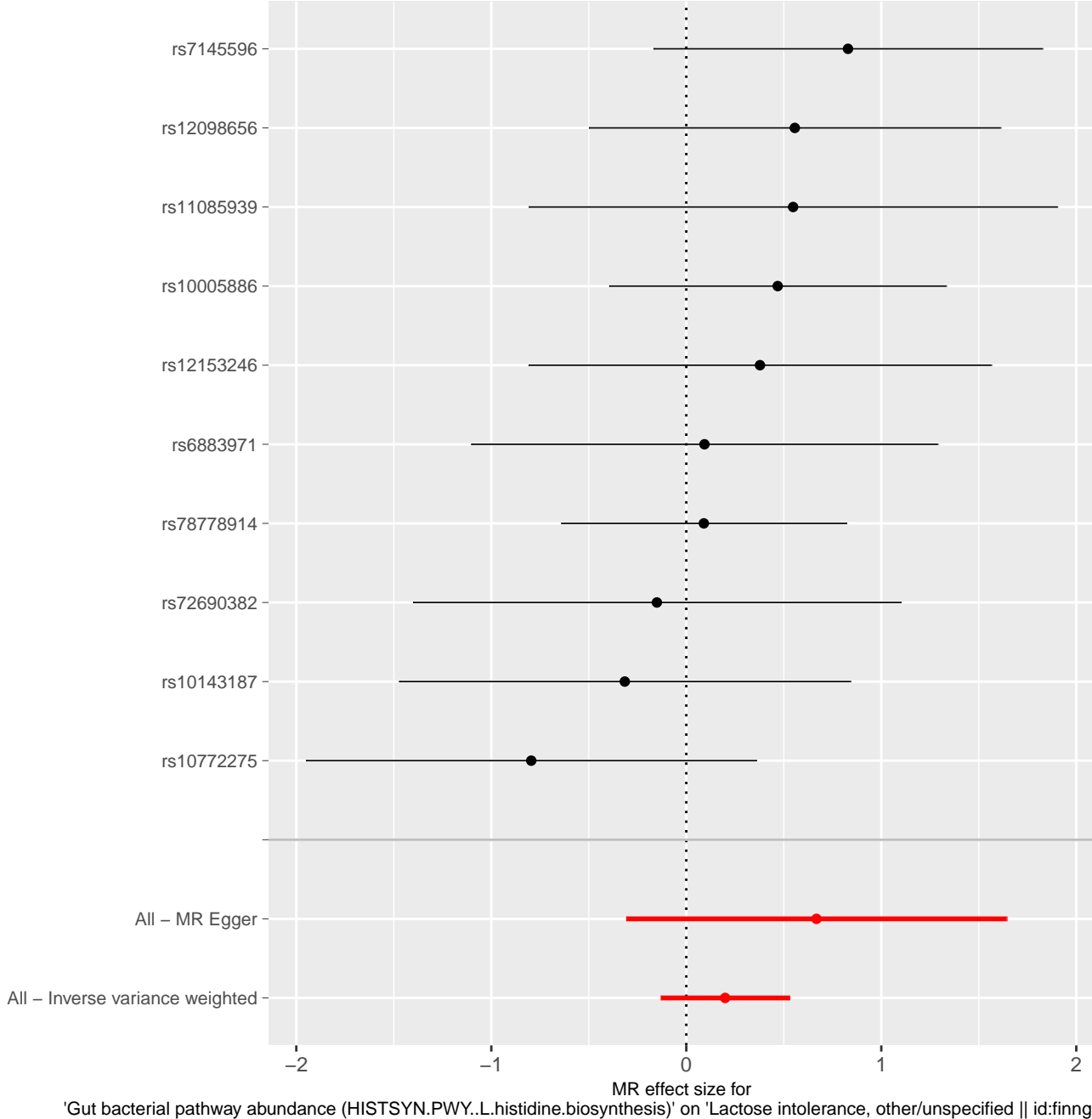

Supplement: Supplementary file 1 [file Data_Sheet_1.zip › supplementary materials/Forward/forest plot/ebi-a-GCST90027482.finngen_R12_E4_LACTONAS.pdf]

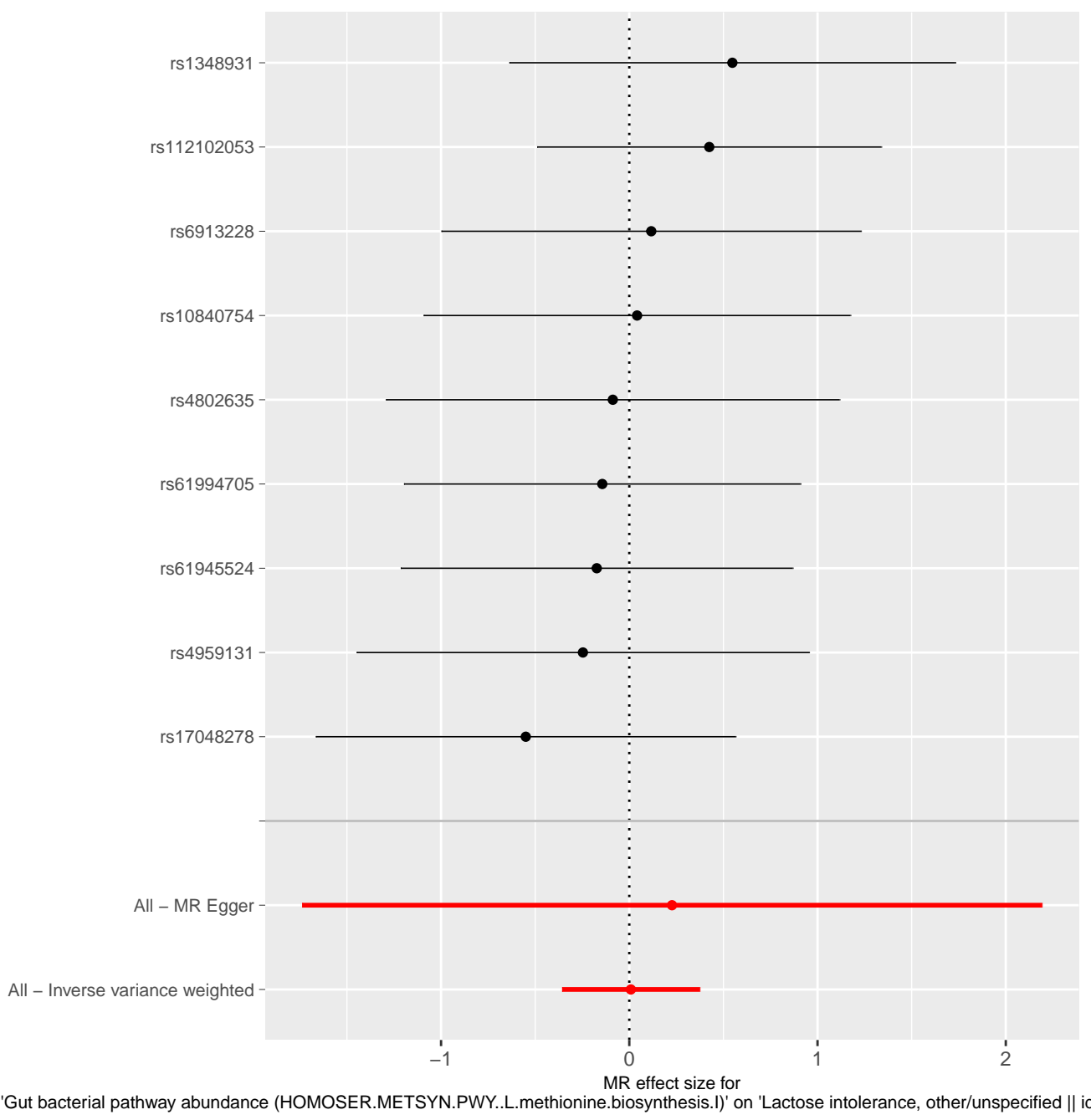

Supplement: Supplementary file 1 [file Data_Sheet_1.zip › supplementary materials/Forward/forest plot/ebi-a-GCST90027483.finngen_R12_E4_LACTONAS.pdf]

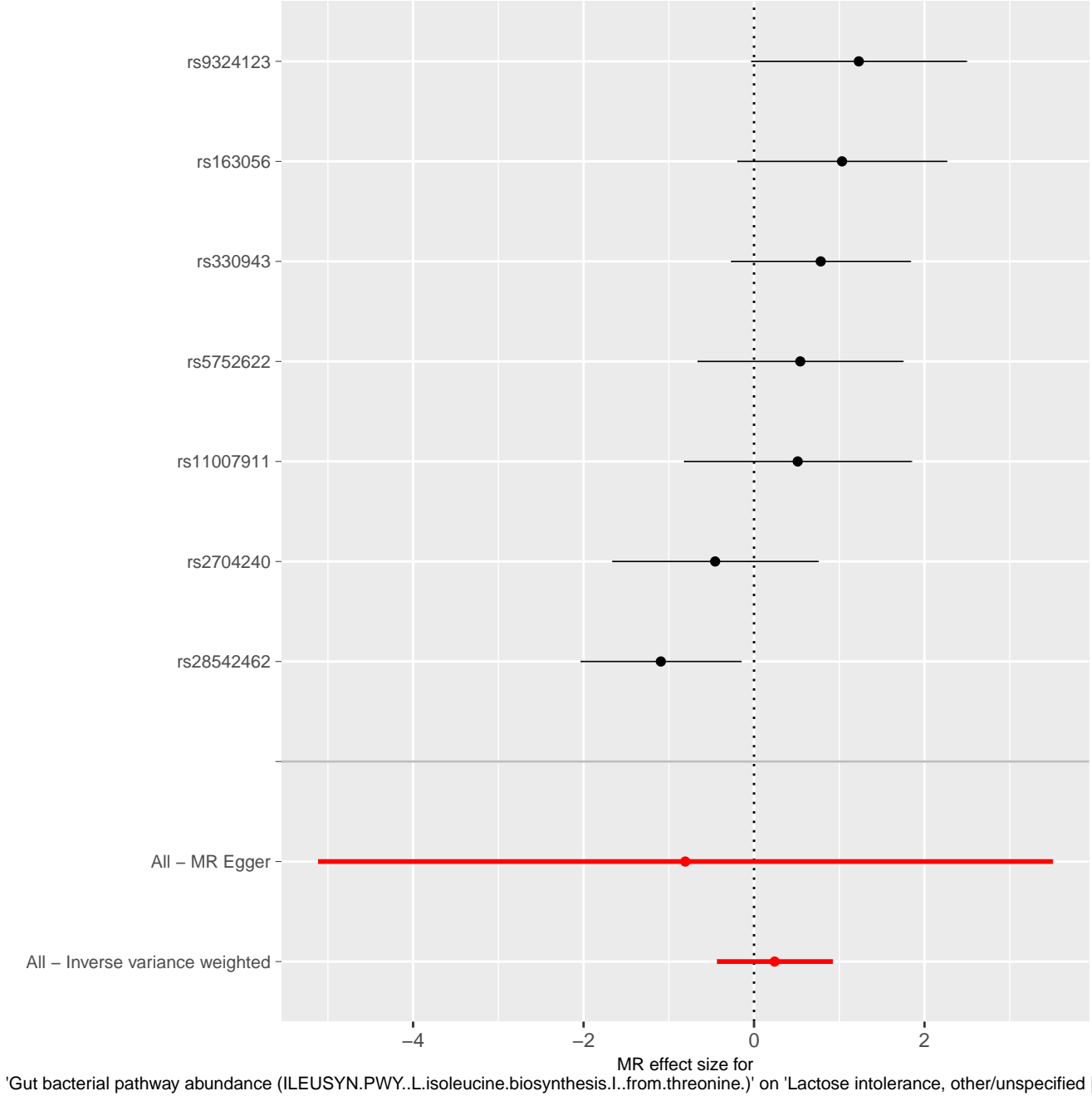

Supplement: Supplementary file 1 [file Data_Sheet_1.zip › supplementary materials/Forward/forest plot/ebi-a-GCST90027485.finngen_R12_E4_LACTONAS.pdf]

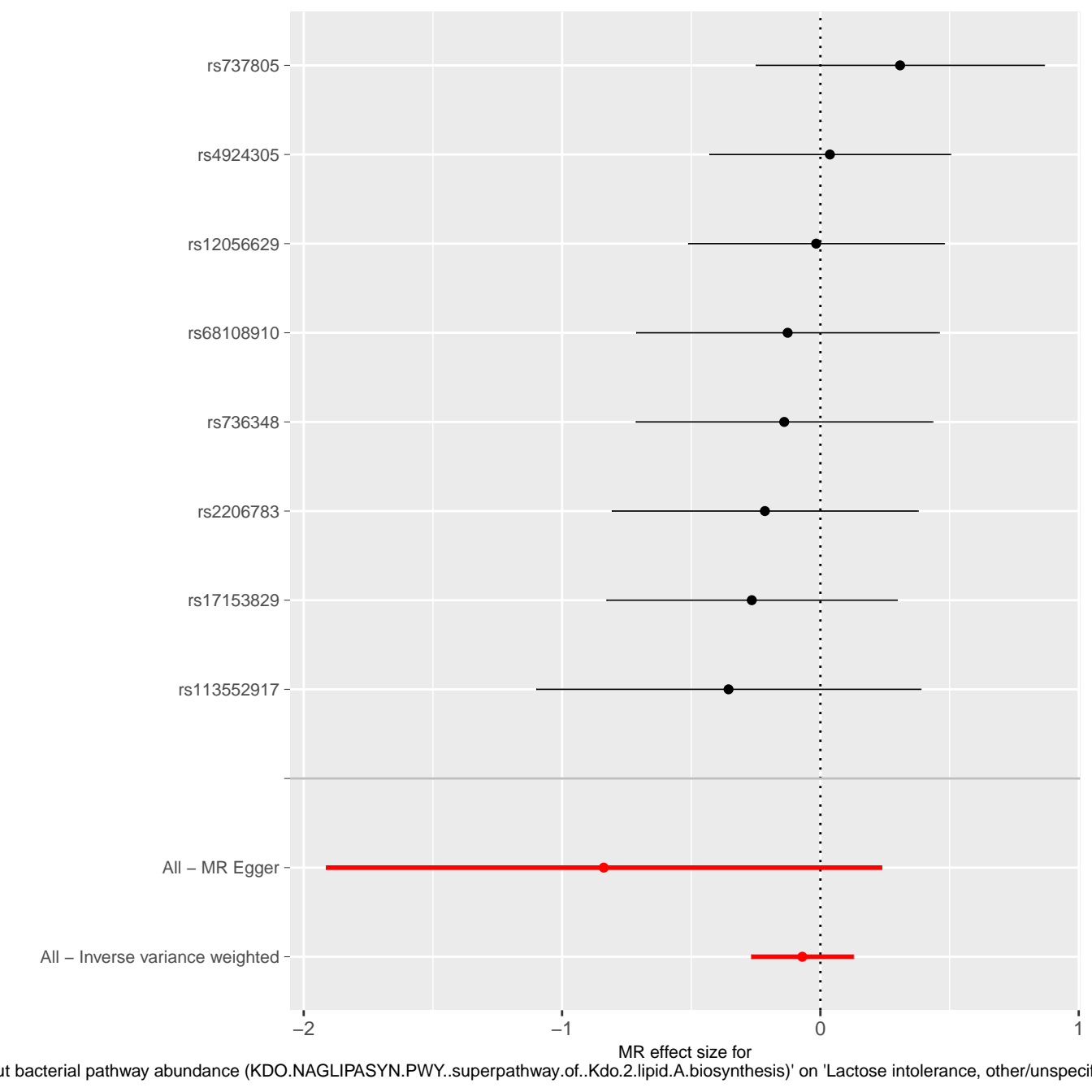

Supplement: Supplementary file 1 [file Data_Sheet_1.zip › supplementary materials/Forward/forest plot/ebi-a-GCST90027486.finngen_R12_E4_LACTONAS.pdf]

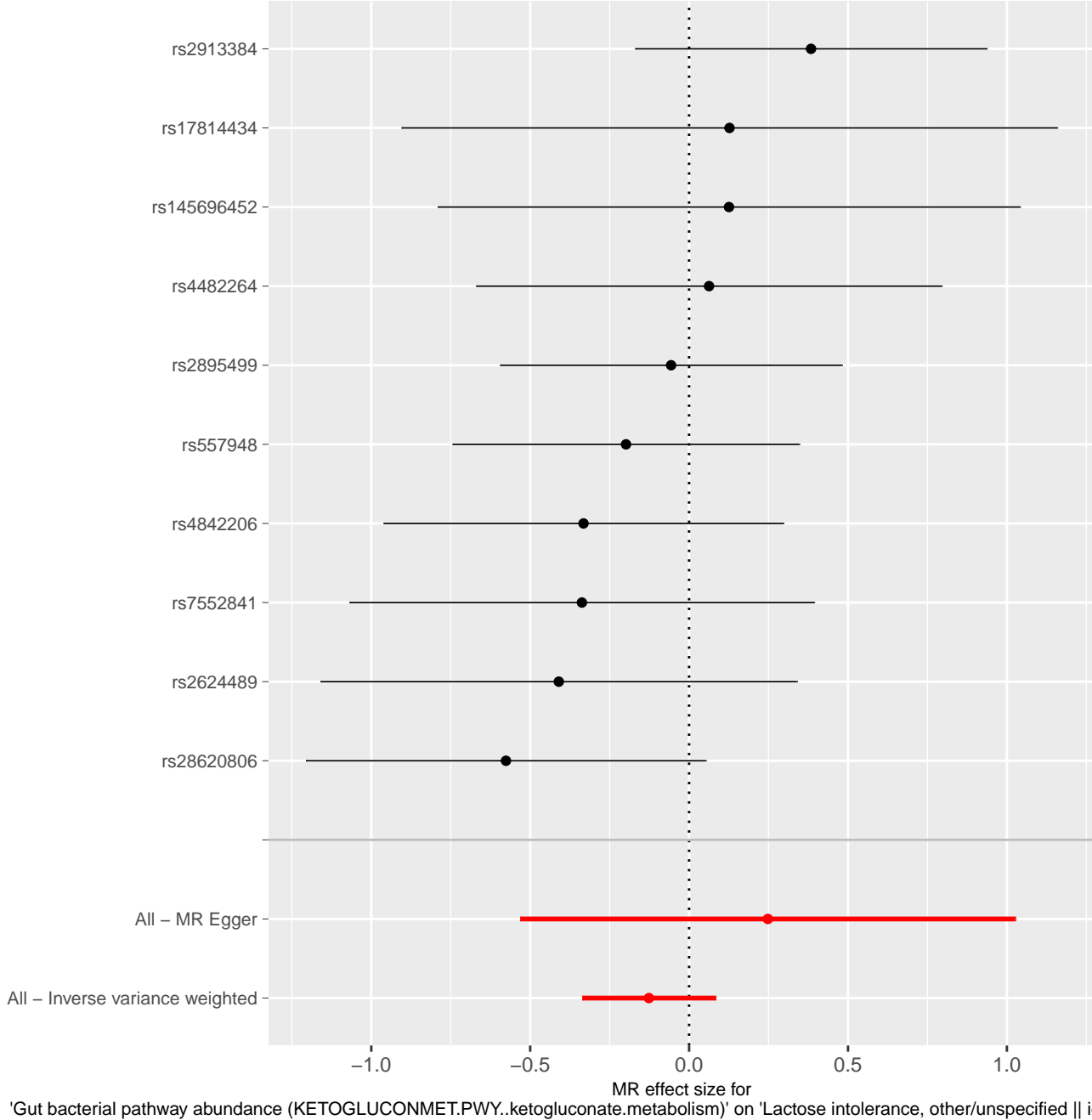

Supplement: Supplementary file 1 [file Data_Sheet_1.zip › supplementary materials/Forward/forest plot/ebi-a-GCST90027487.finngen_R12_E4_LACTONAS.pdf]

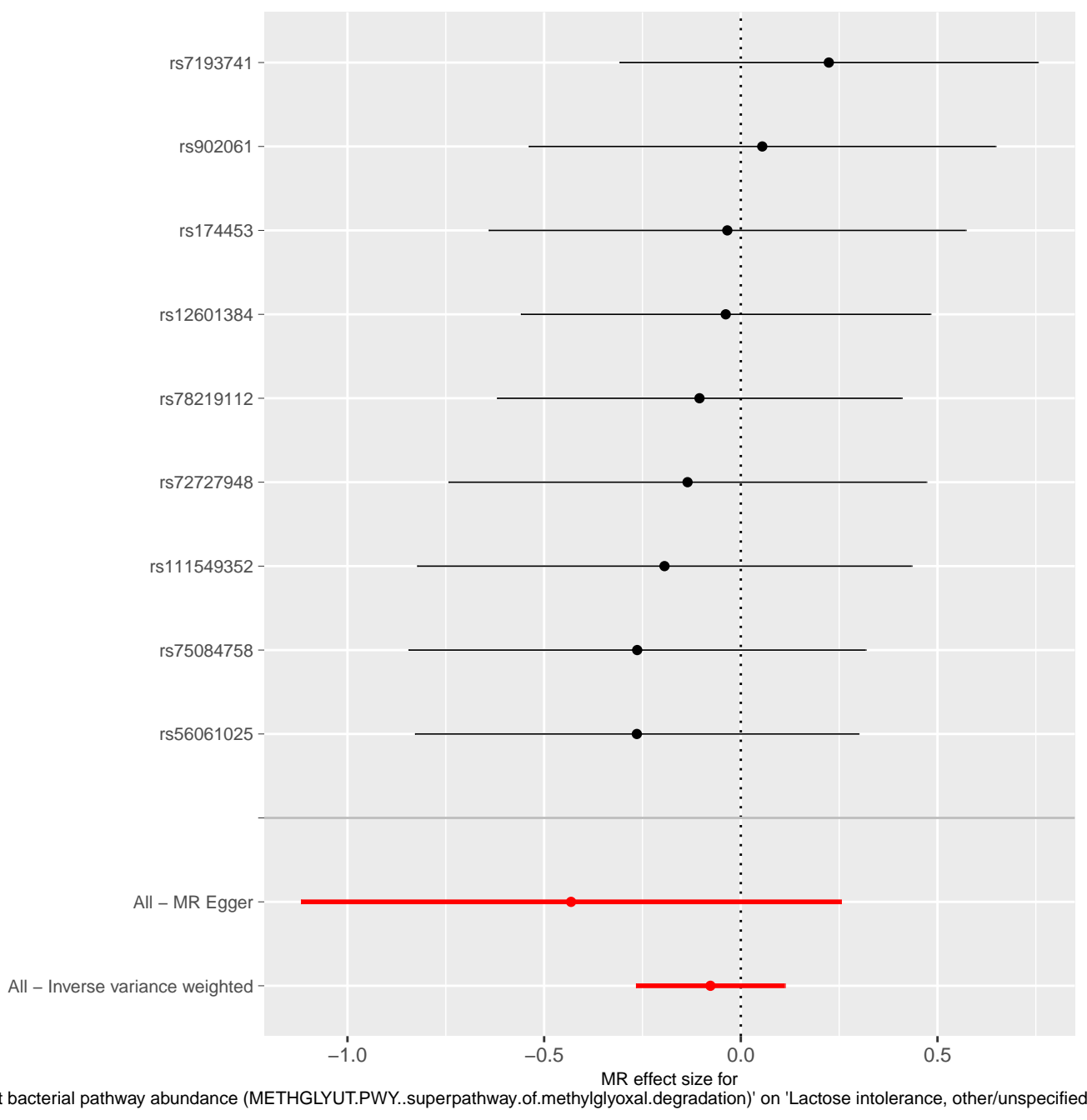

Supplement: Supplementary file 1 [file Data_Sheet_1.zip › supplementary materials/Forward/forest plot/ebi-a-GCST90027489.finngen_R12_E4_LACTONAS.pdf]

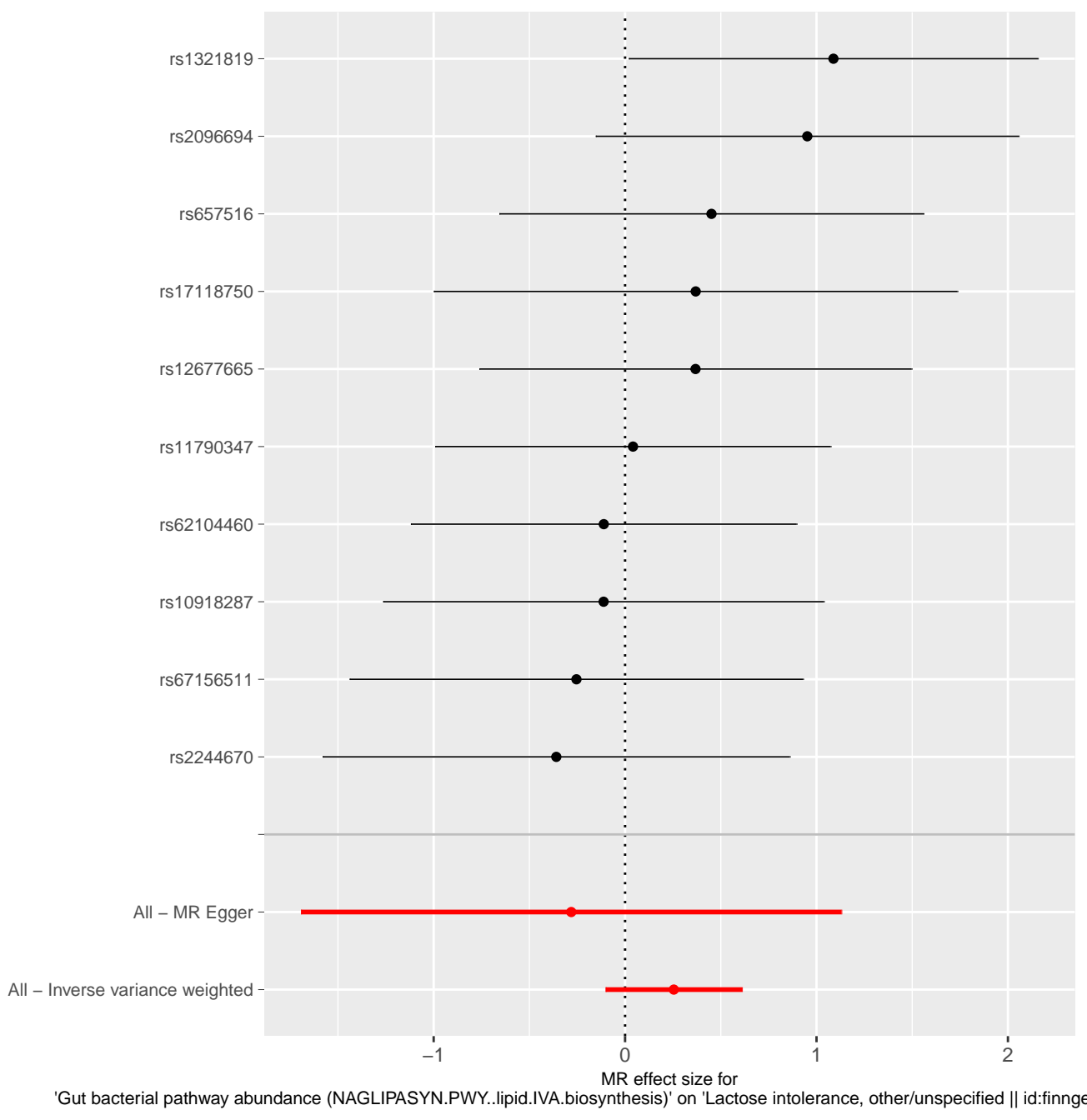

Supplement: Supplementary file 1 [file Data_Sheet_1.zip › supplementary materials/Forward/forest plot/ebi-a-GCST90027490.finngen_R12_E4_LACTONAS.pdf]

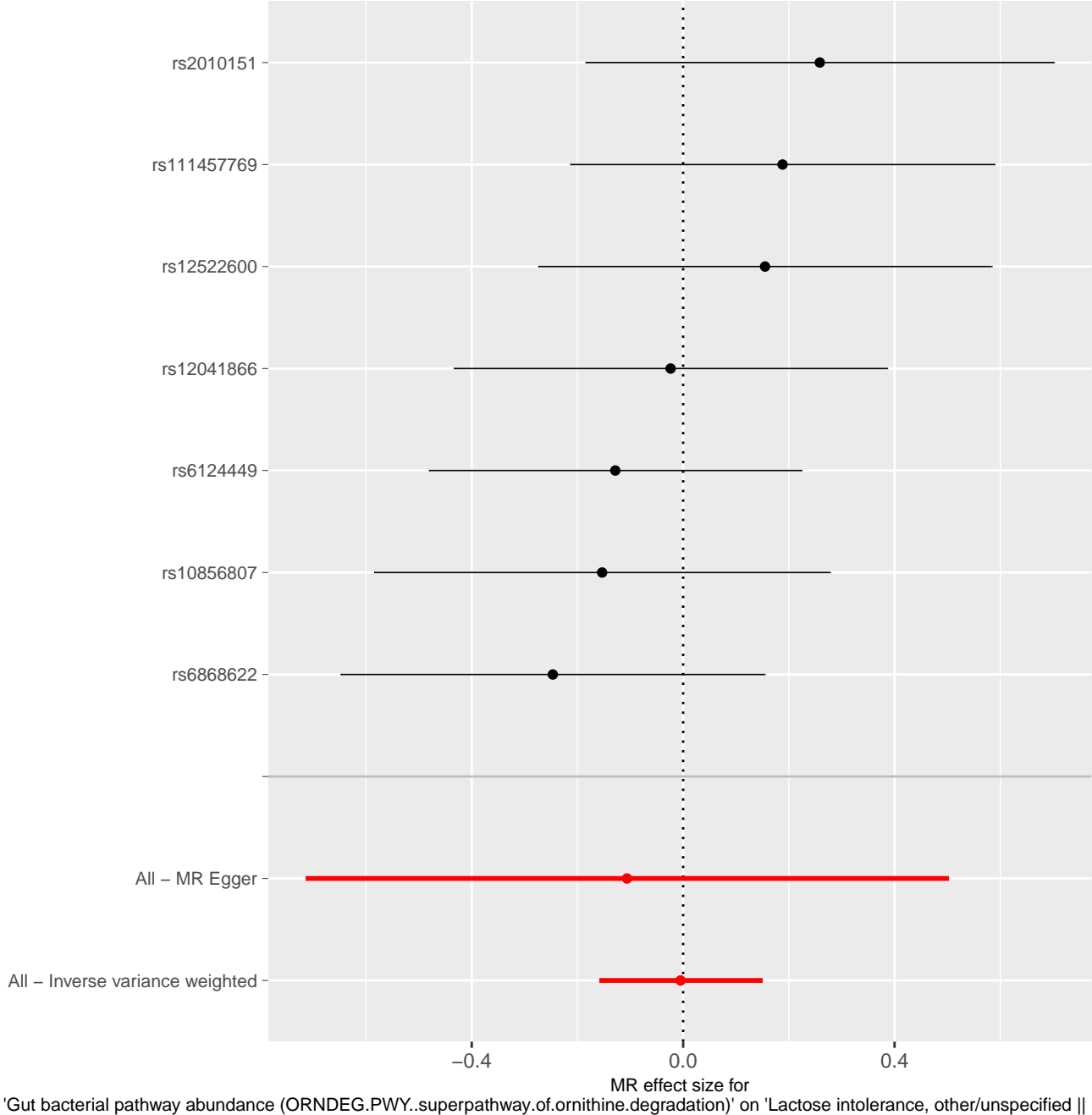

Supplement: Supplementary file 1 [file Data_Sheet_1.zip › supplementary materials/Forward/forest plot/ebi-a-GCST90027493.finngen_R12_E4_LACTONAS.pdf]

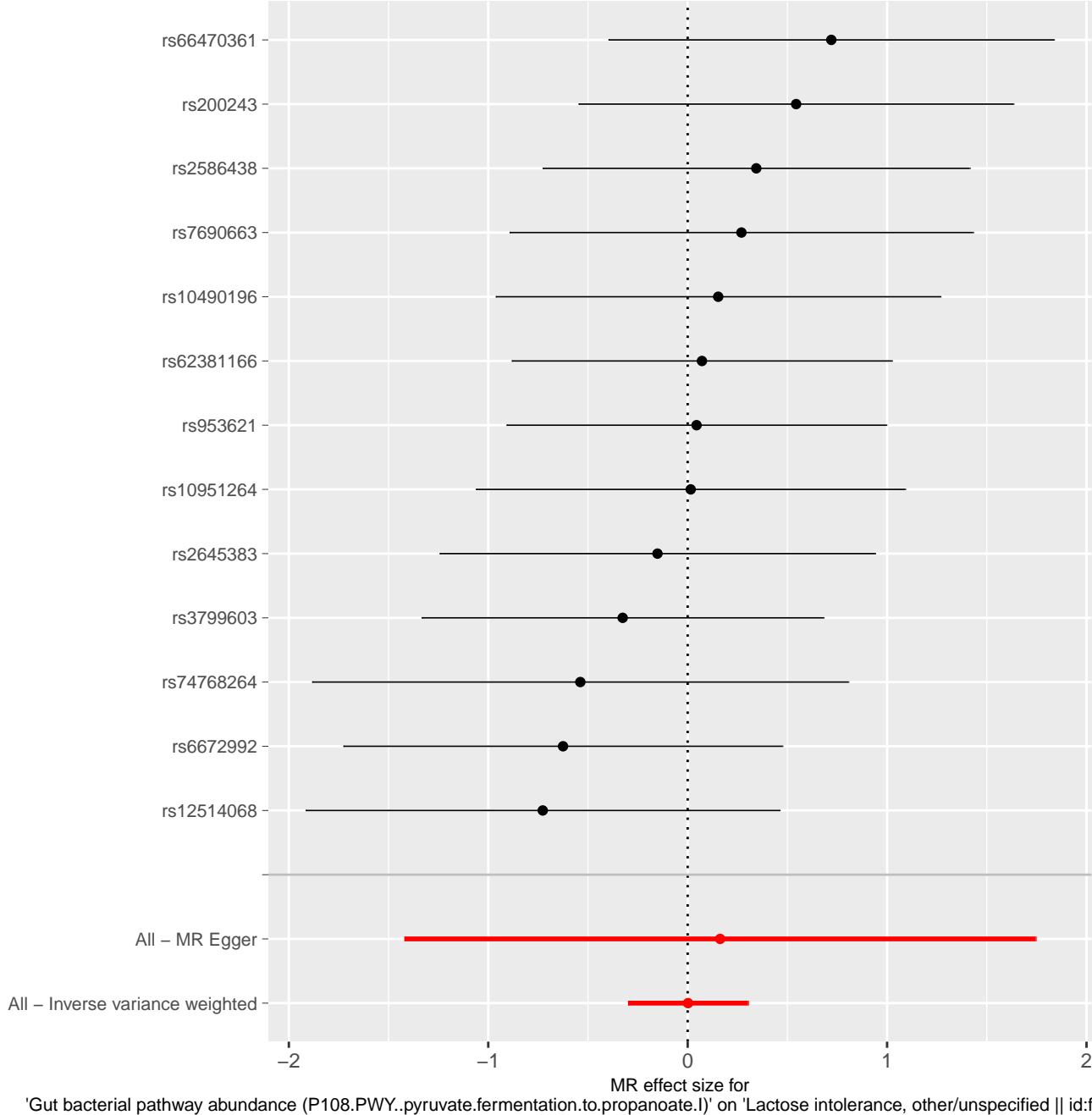

Supplement: Supplementary file 1 [file Data_Sheet_1.zip › supplementary materials/Forward/forest plot/ebi-a-GCST90027495.finngen_R12_E4_LACTONAS.pdf]

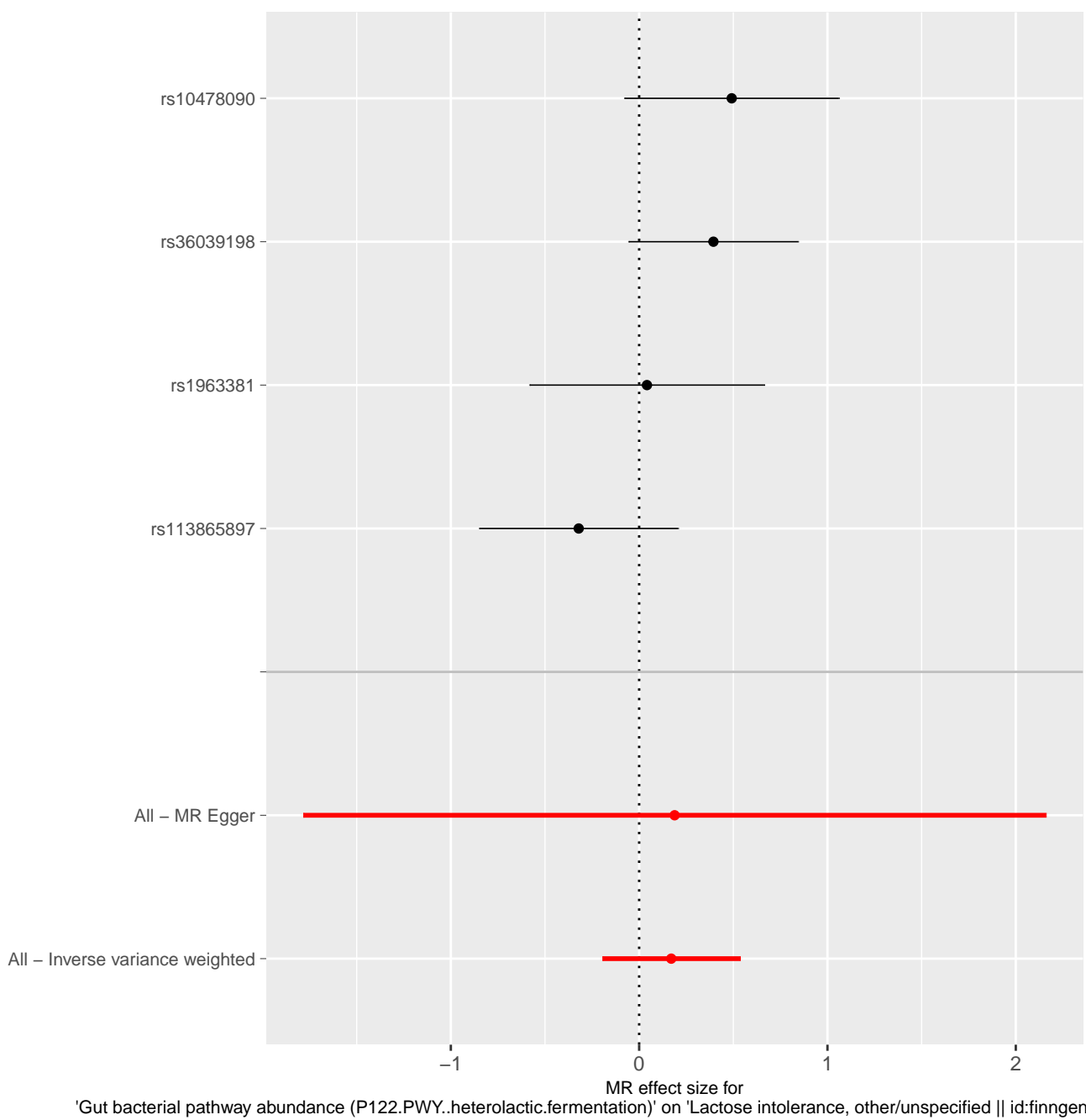

Supplement: Supplementary file 1 [file Data_Sheet_1.zip › supplementary materials/Forward/forest plot/ebi-a-GCST90027496.finngen_R12_E4_LACTONAS.pdf]

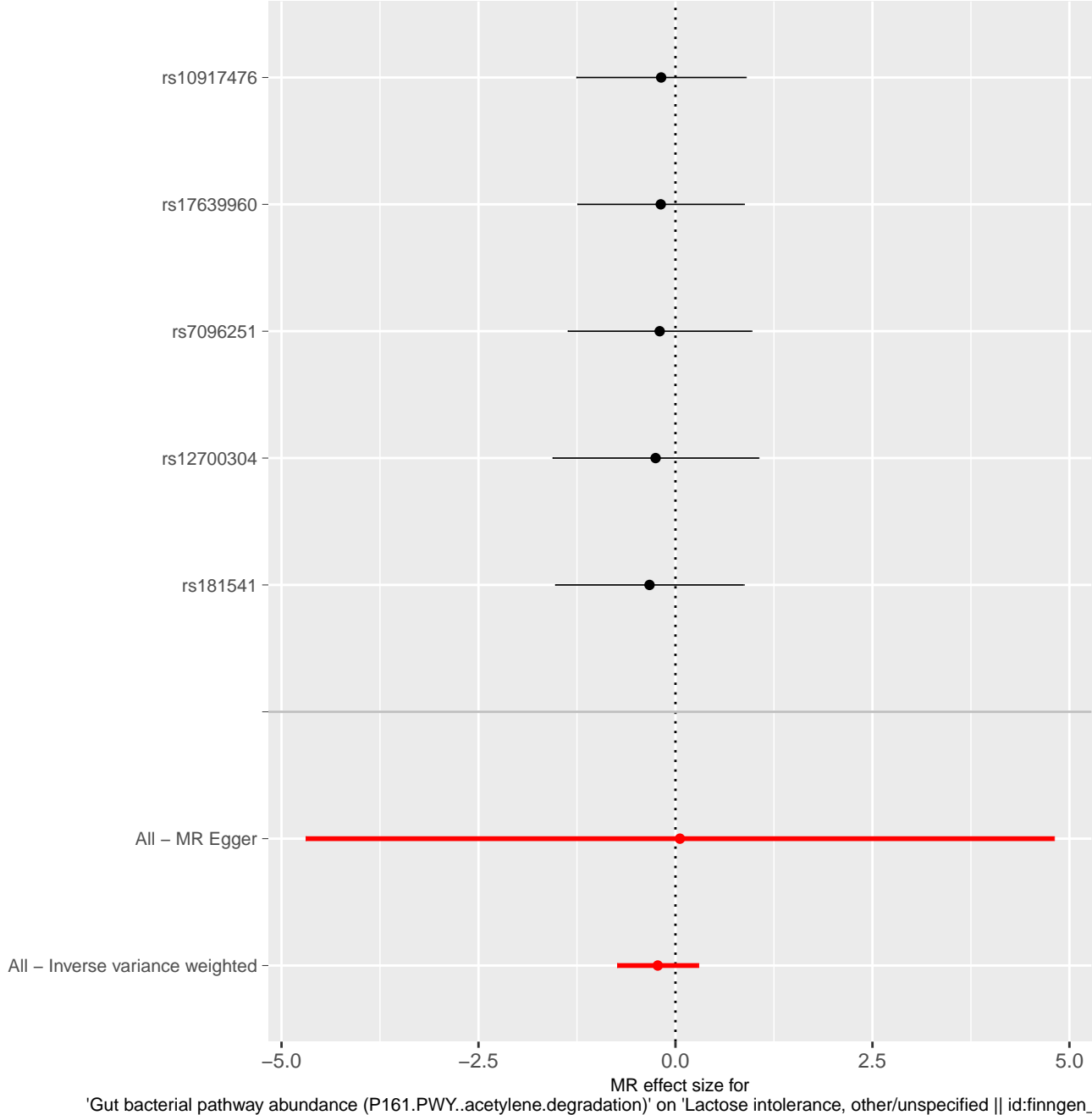

Supplement: Supplementary file 1 [file Data_Sheet_1.zip › supplementary materials/Forward/forest plot/ebi-a-GCST90027497.finngen_R12_E4_LACTONAS.pdf]

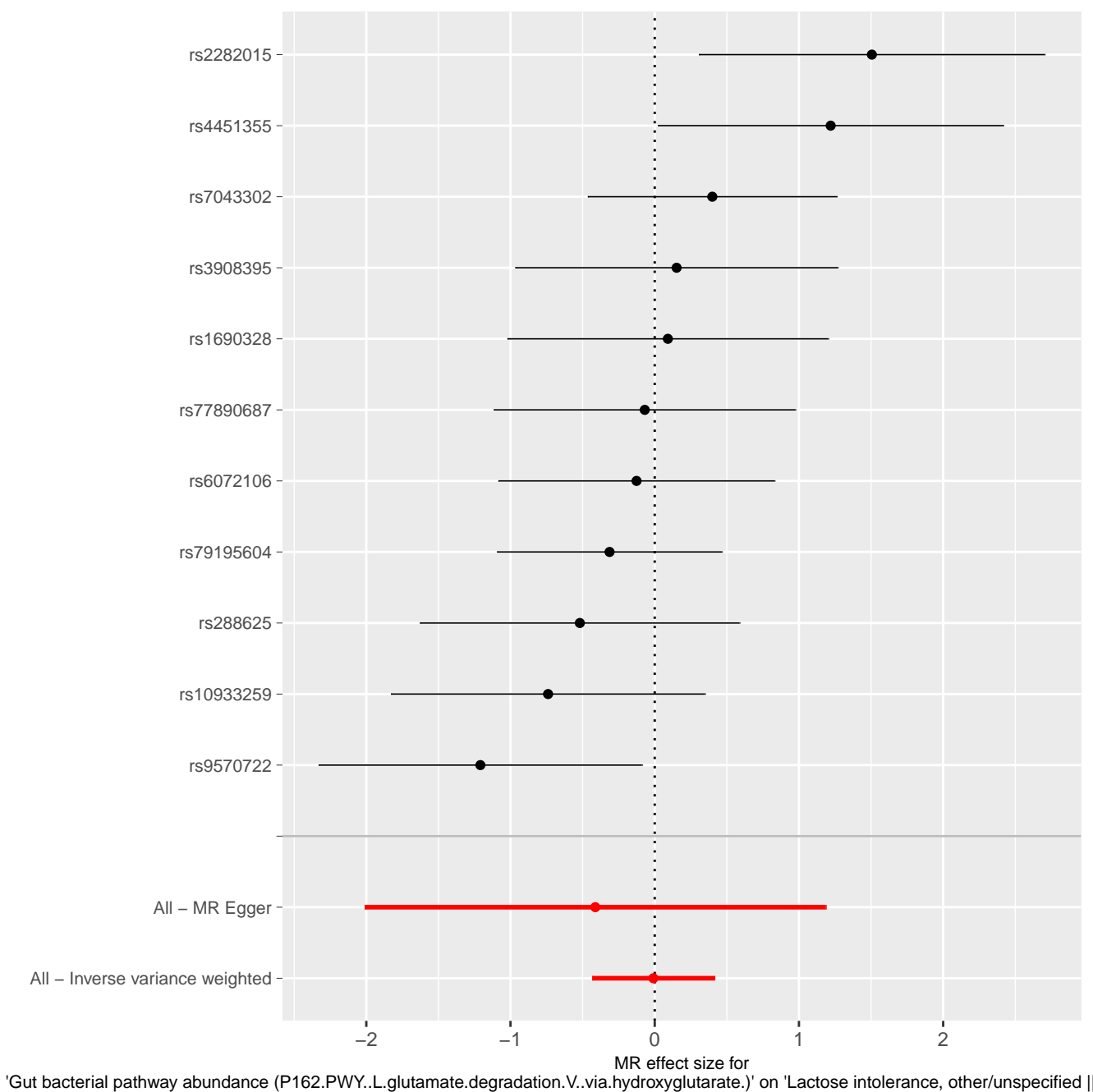

Supplement: Supplementary file 1 [file Data_Sheet_1.zip › supplementary materials/Forward/forest plot/ebi-a-GCST90027498.finngen_R12_E4_LACTONAS.pdf]

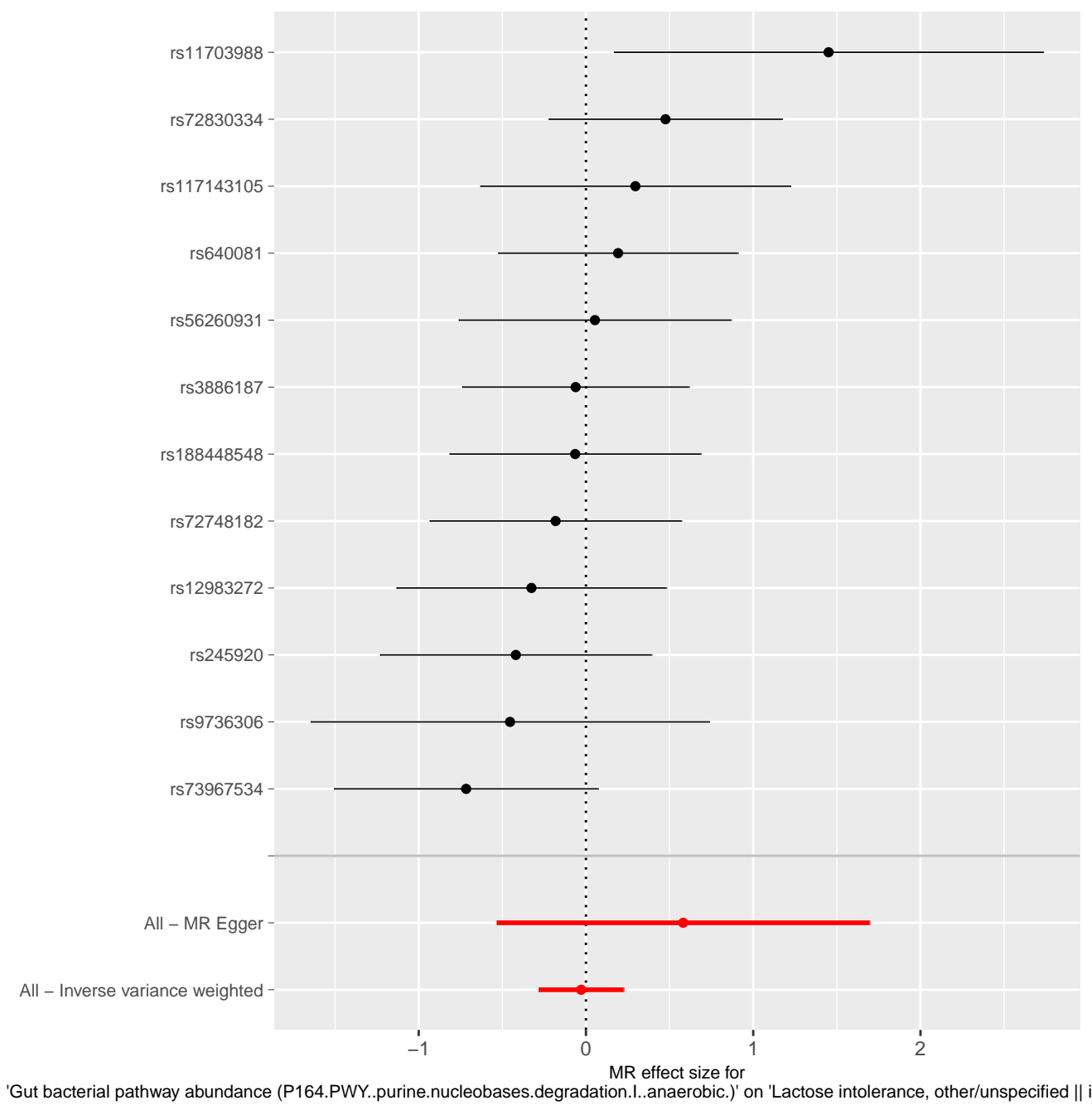

Supplement: Supplementary file 1 [file Data_Sheet_1.zip › supplementary materials/Forward/forest plot/ebi-a-GCST90027499.finngen_R12_E4_LACTONAS.pdf]

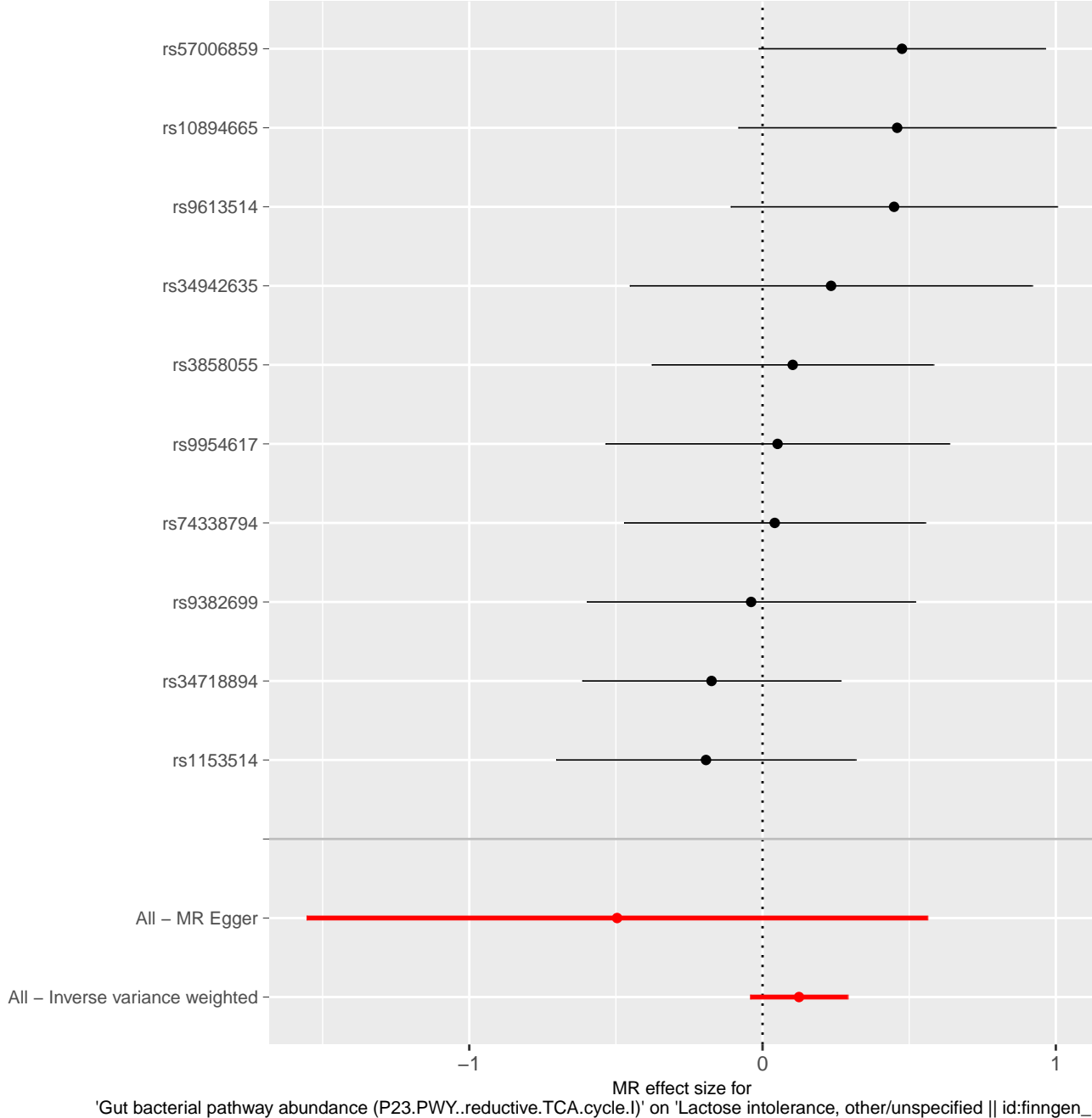

Supplement: Supplementary file 1 [file Data_Sheet_1.zip › supplementary materials/Forward/forest plot/ebi-a-GCST90027500.finngen_R12_E4_LACTONAS.pdf]

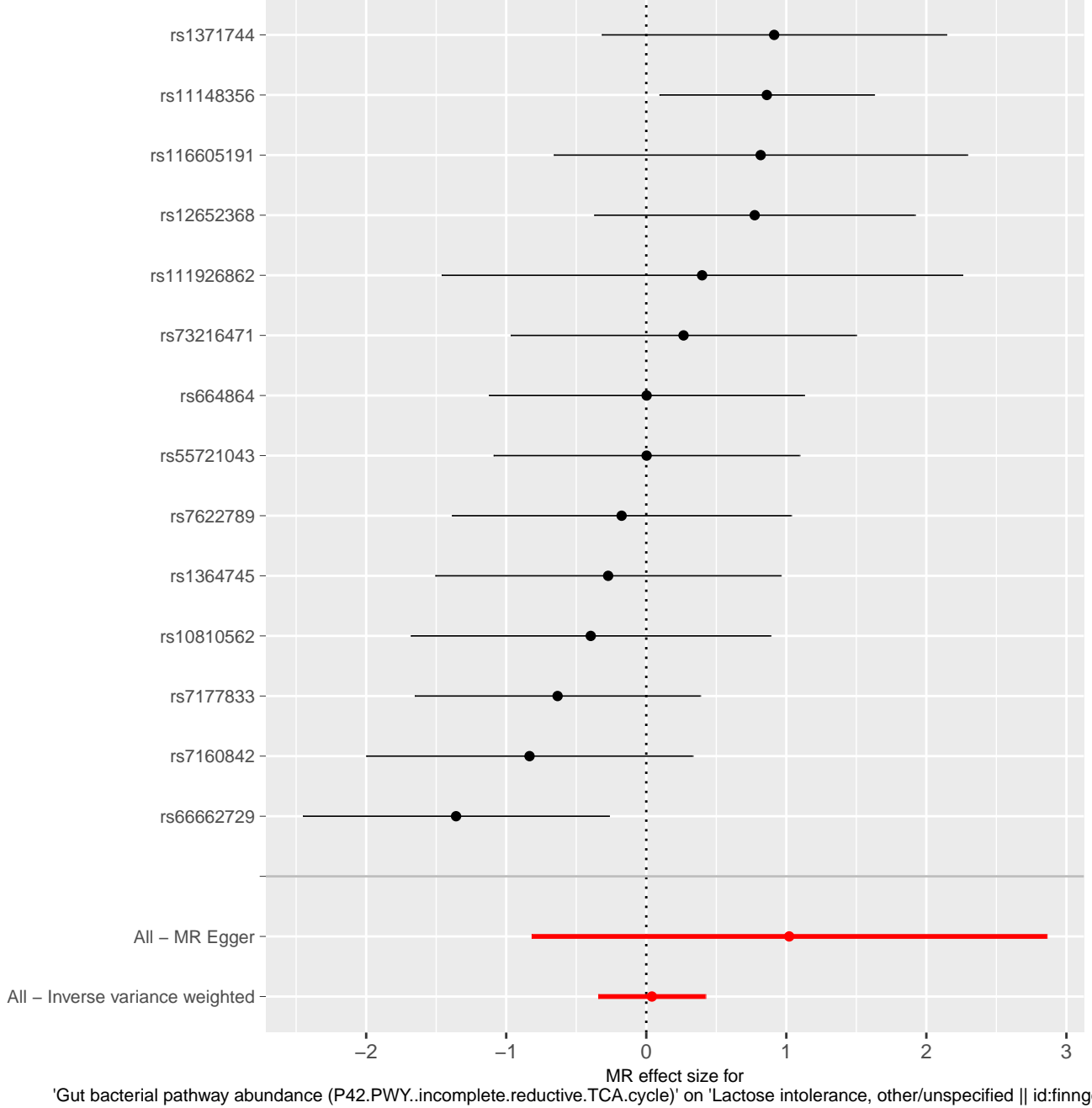

Supplement: Supplementary file 1 [file Data_Sheet_1.zip › supplementary materials/Forward/forest plot/ebi-a-GCST90027501.finngen_R12_E4_LACTONAS.pdf]

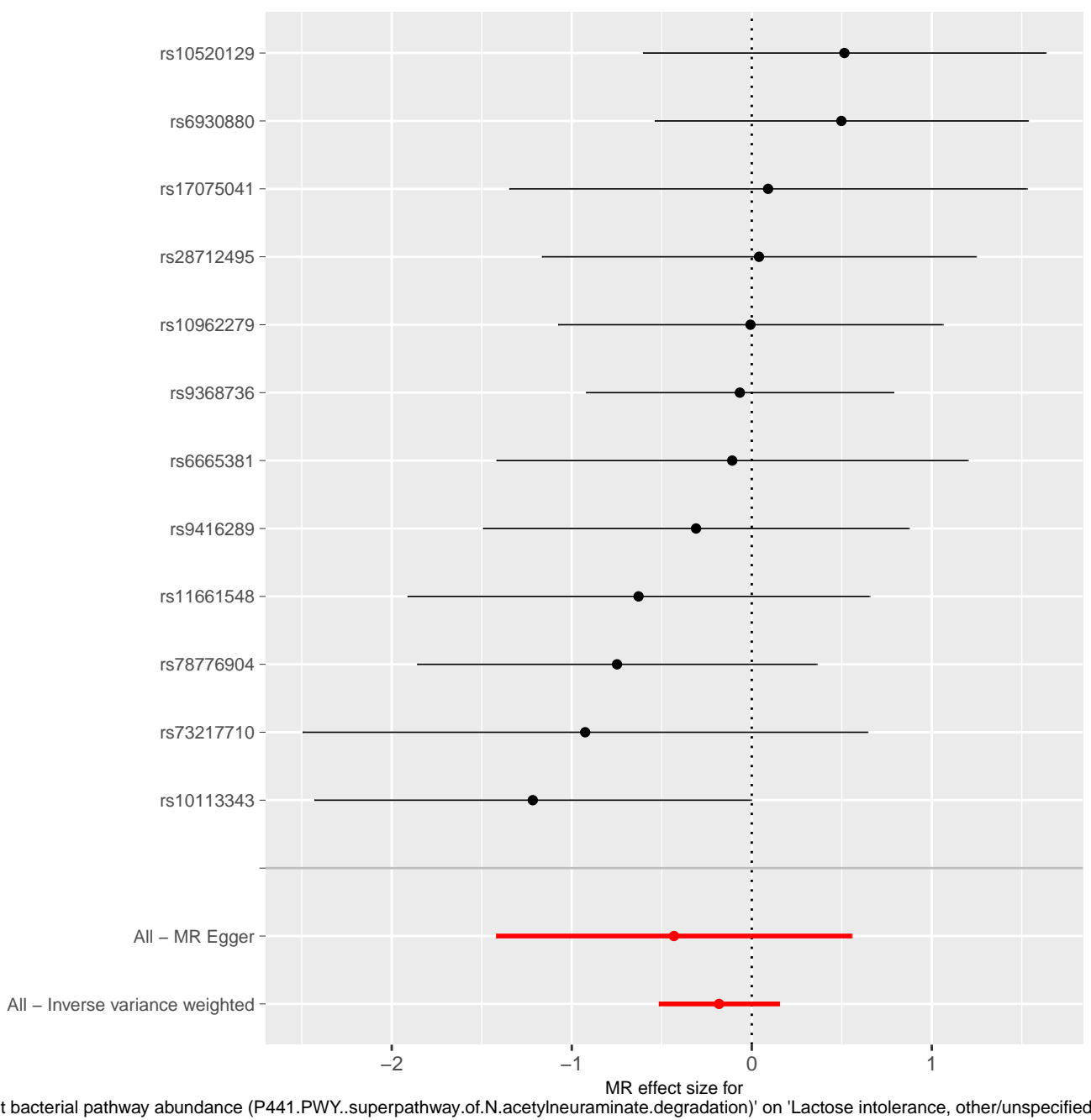

Supplement: Supplementary file 1 [file Data_Sheet_1.zip › supplementary materials/Forward/forest plot/ebi-a-GCST90027502.finngen_R12_E4_LACTONAS.pdf]

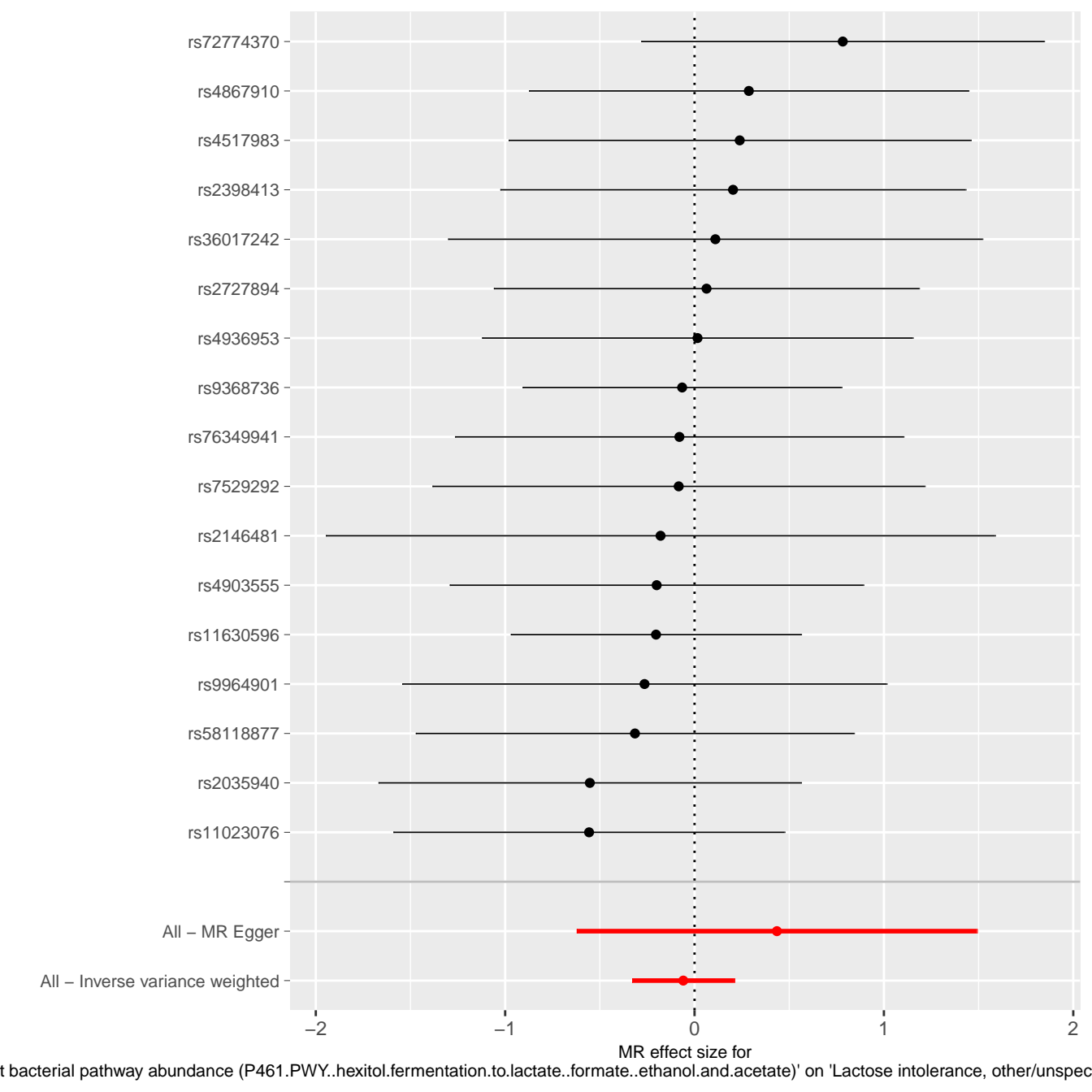

Supplement: Supplementary file 1 [file Data_Sheet_1.zip › supplementary materials/Forward/forest plot/ebi-a-GCST90027503.finngen_R12_E4_LACTONAS.pdf]

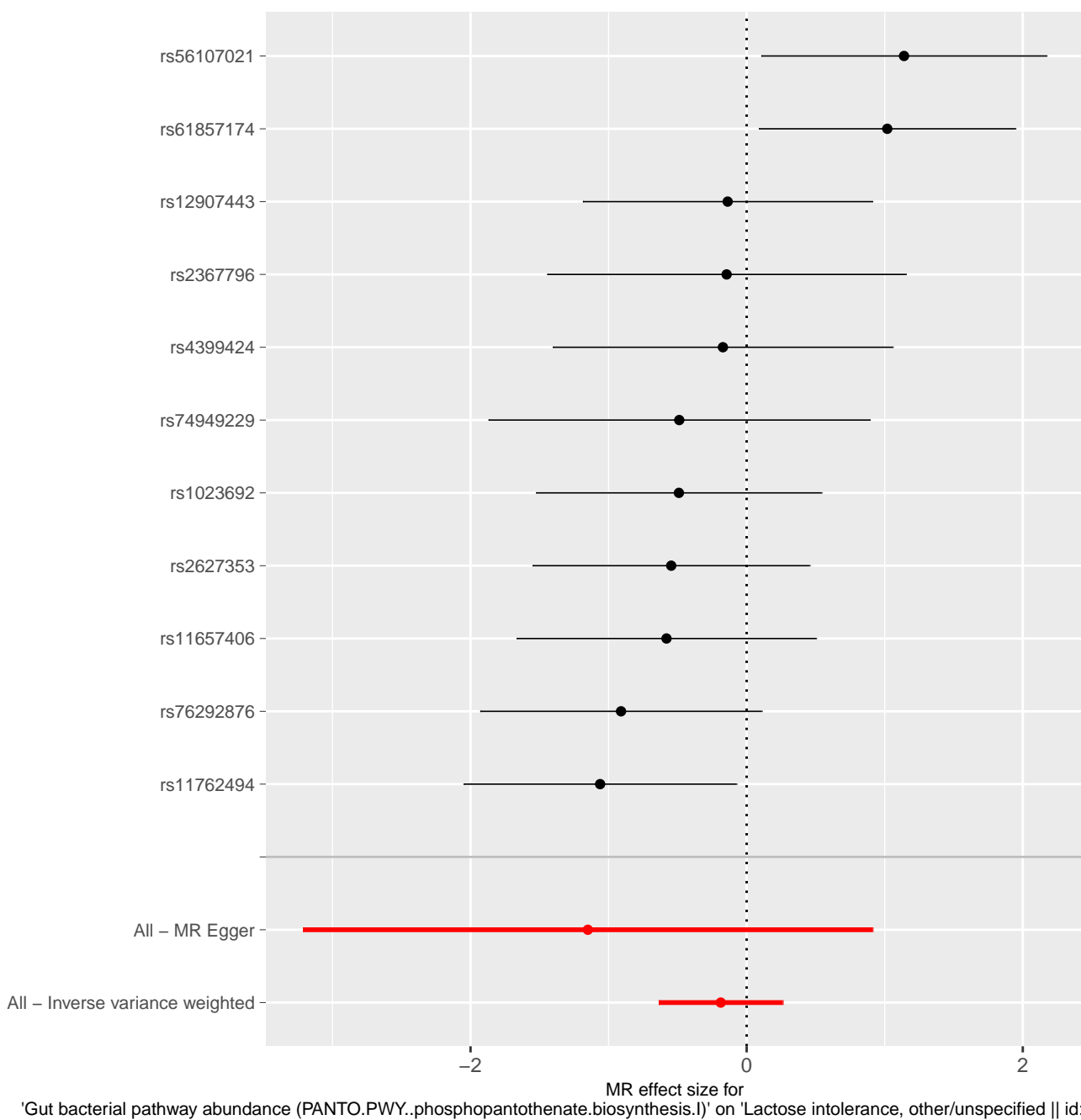

Supplement: Supplementary file 1 [file Data_Sheet_1.zip › supplementary materials/Forward/forest plot/ebi-a-GCST90027504.finngen_R12_E4_LACTONAS.pdf]

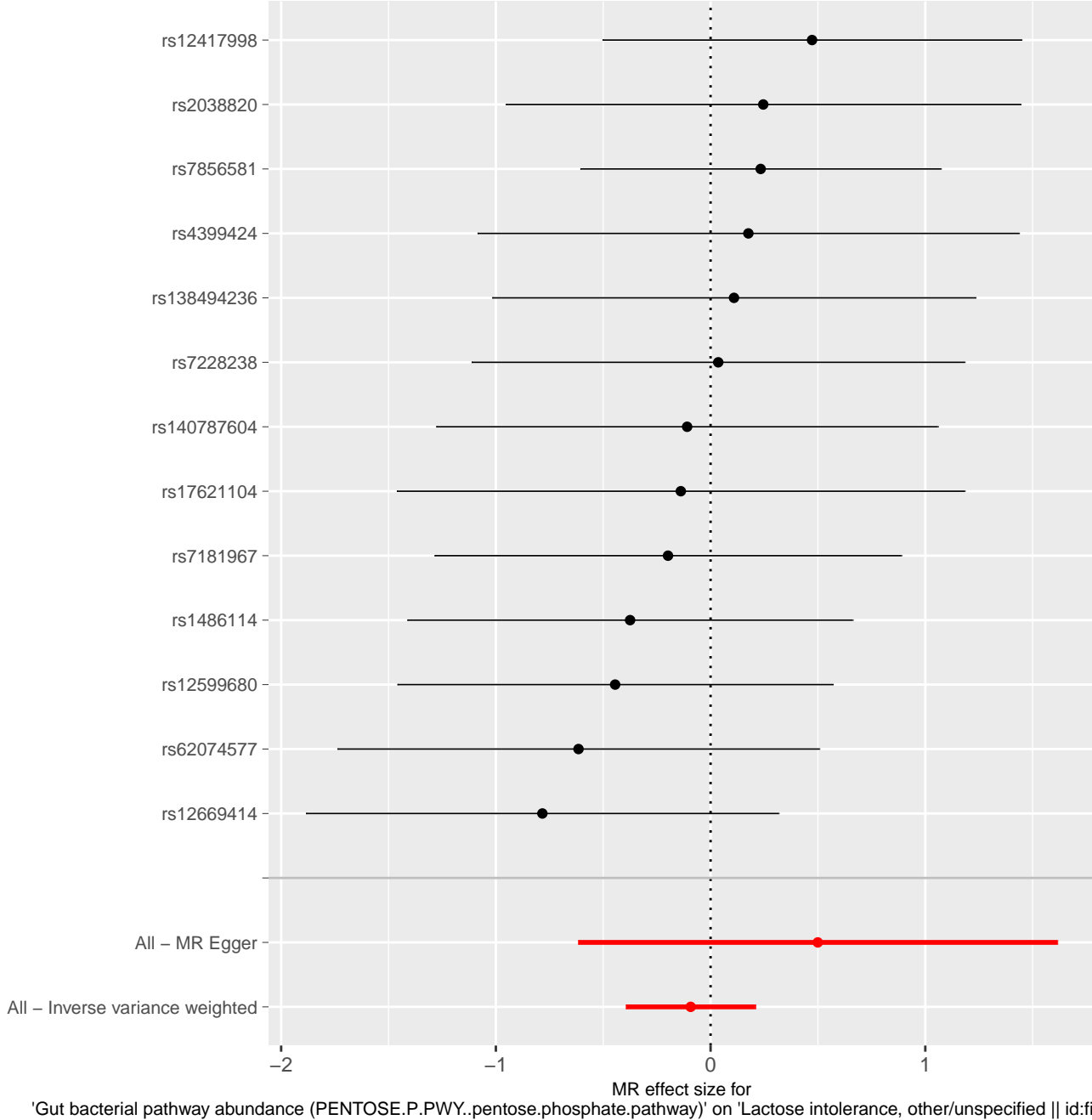

Supplement: Supplementary file 1 [file Data_Sheet_1.zip › supplementary materials/Forward/forest plot/ebi-a-GCST90027506.finngen_R12_E4_LACTONAS.pdf]

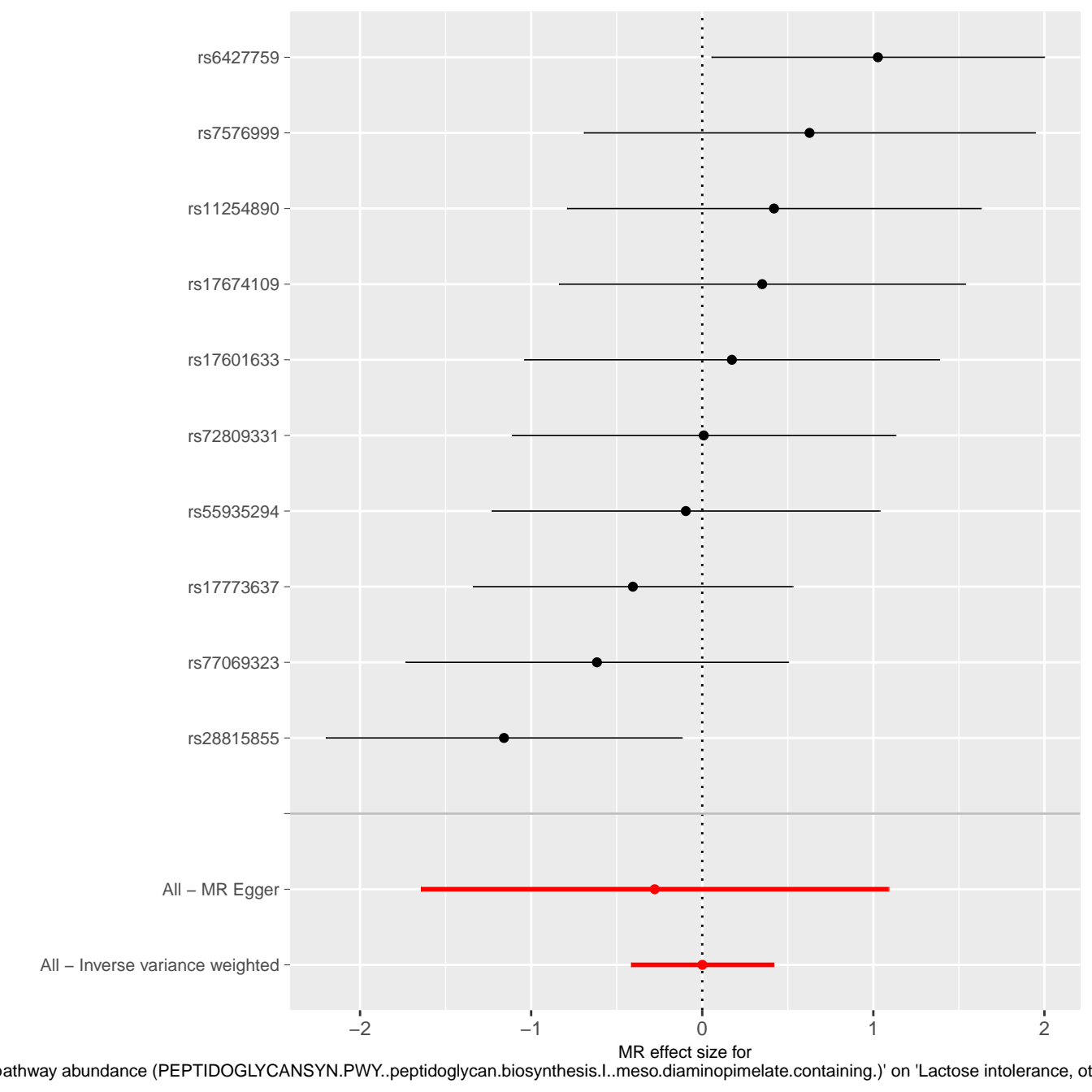

Supplement: Supplementary file 1 [file Data_Sheet_1.zip › supplementary materials/Forward/forest plot/ebi-a-GCST90027507.finngen_R12_E4_LACTONAS.pdf]

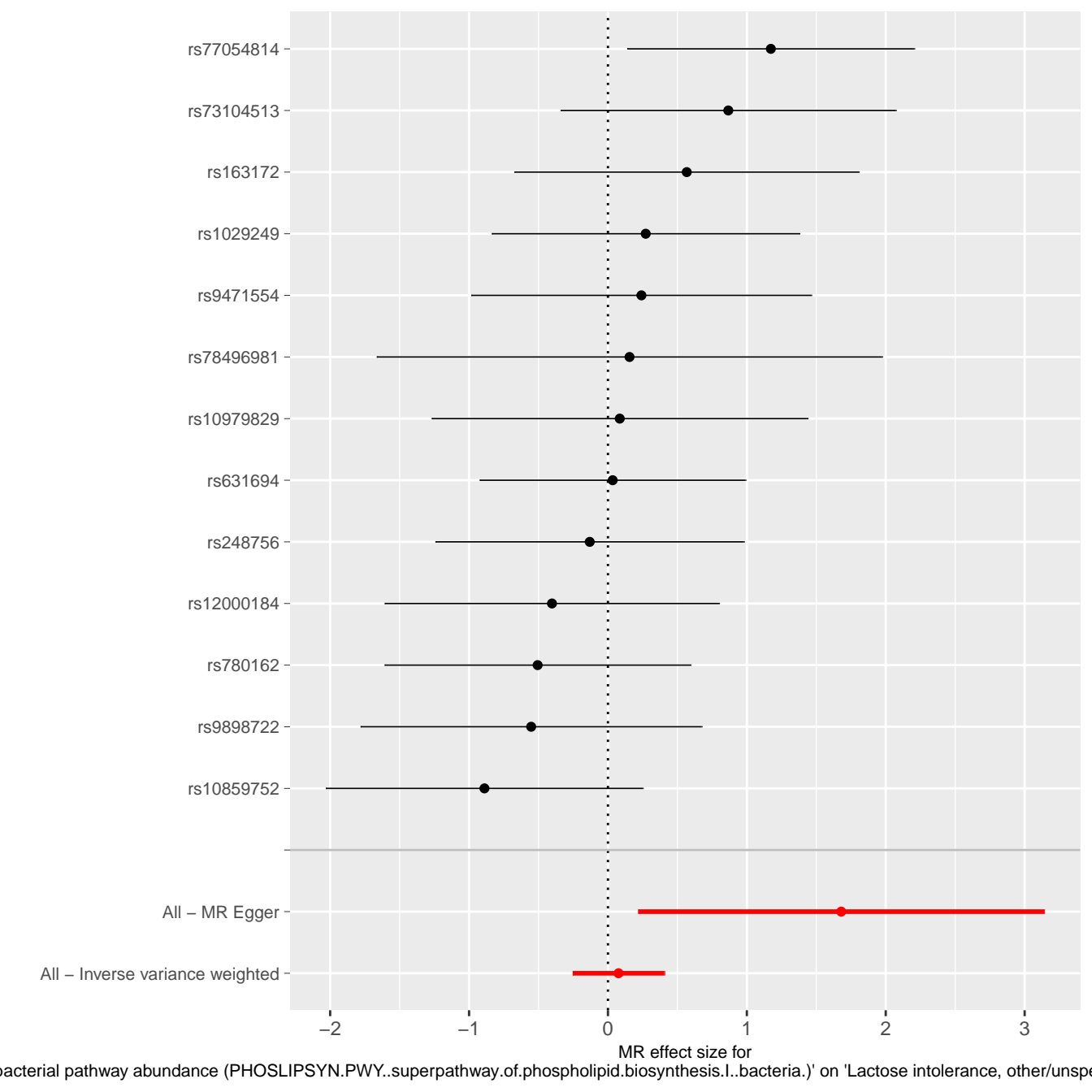

Supplement: Supplementary file 1 [file Data_Sheet_1.zip › supplementary materials/Forward/forest plot/ebi-a-GCST90027508.finngen_R12_E4_LACTONAS.pdf]

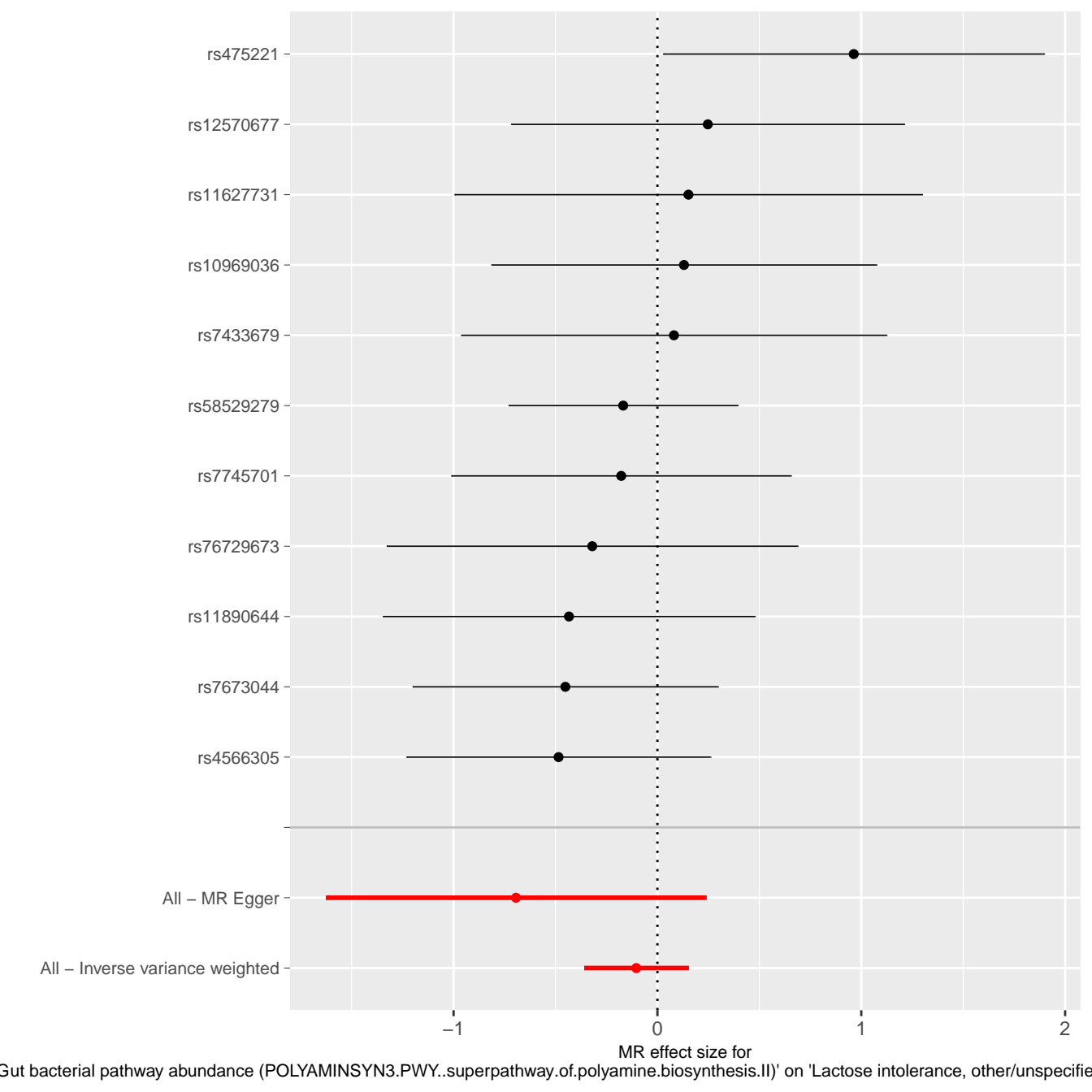

Supplement: Supplementary file 1 [file Data_Sheet_1.zip › supplementary materials/Forward/forest plot/ebi-a-GCST90027509.finngen_R12_E4_LACTONAS.pdf]

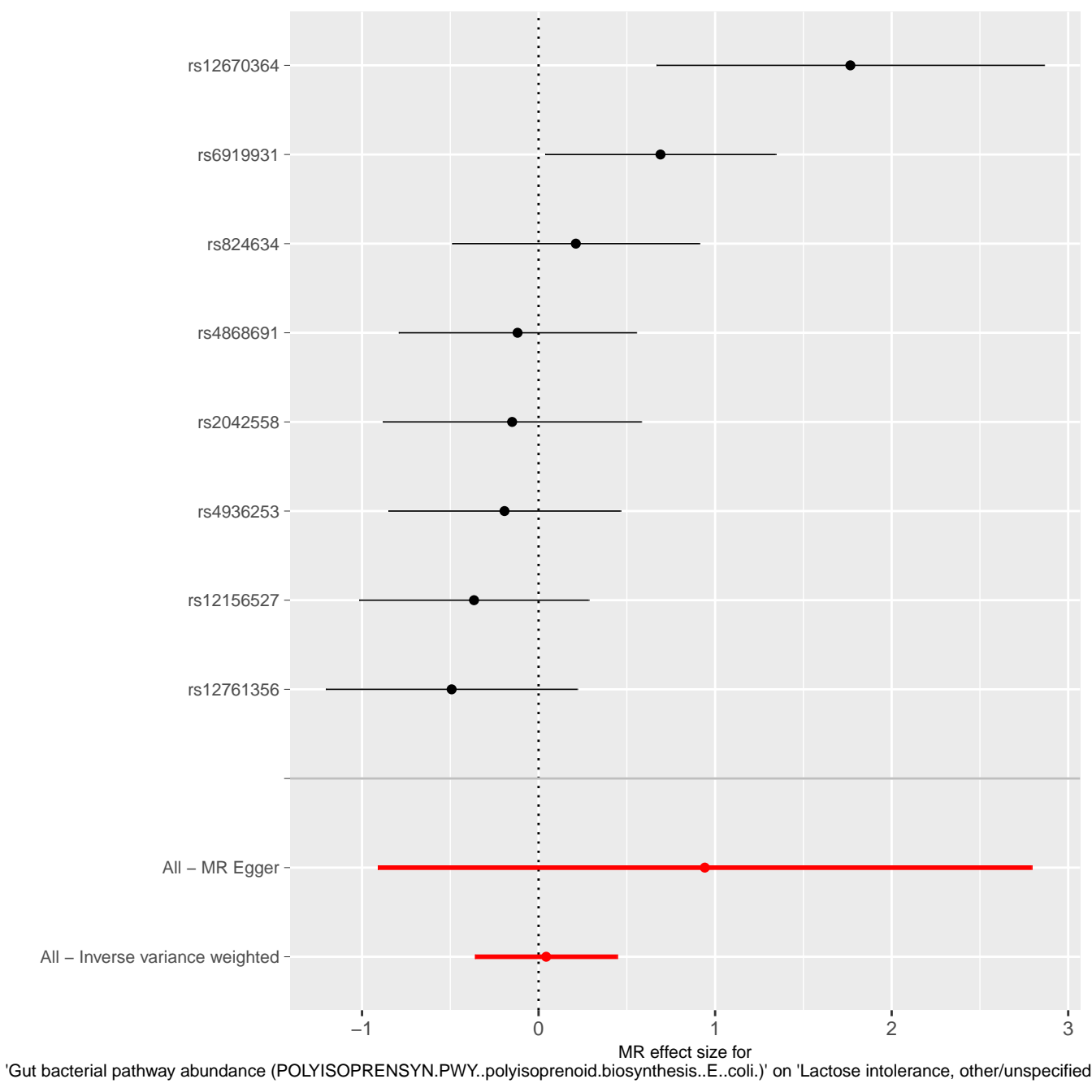

Supplement: Supplementary file 1 [file Data_Sheet_1.zip › supplementary materials/Forward/forest plot/ebi-a-GCST90027511.finngen_R12_E4_LACTONAS.pdf]

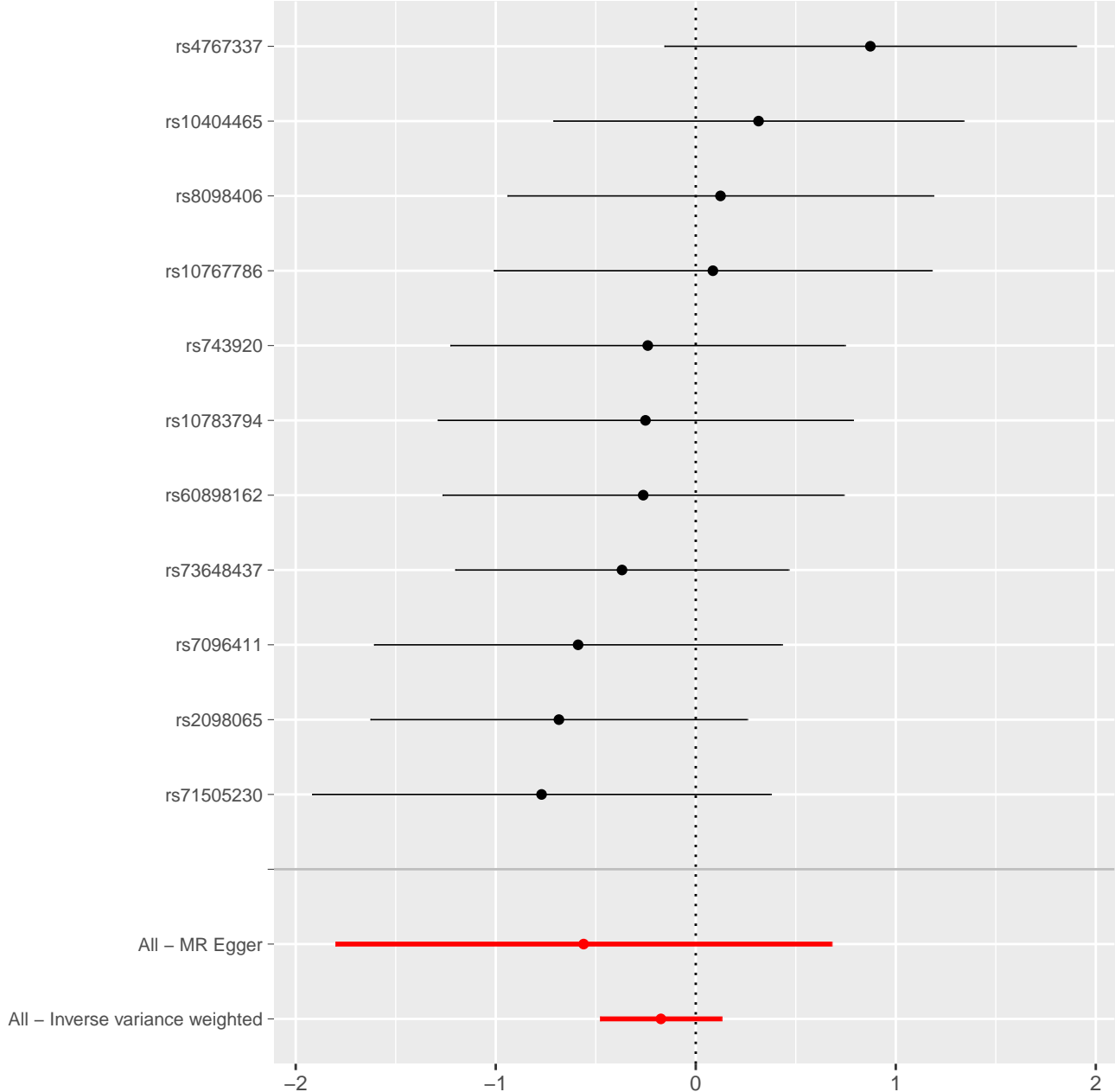

Supplement: Supplementary file 1 [file Data_Sheet_1.zip › supplementary materials/Forward/forest plot/ebi-a-GCST90027512.finngen_R12_E4_LACTONAS.pdf]

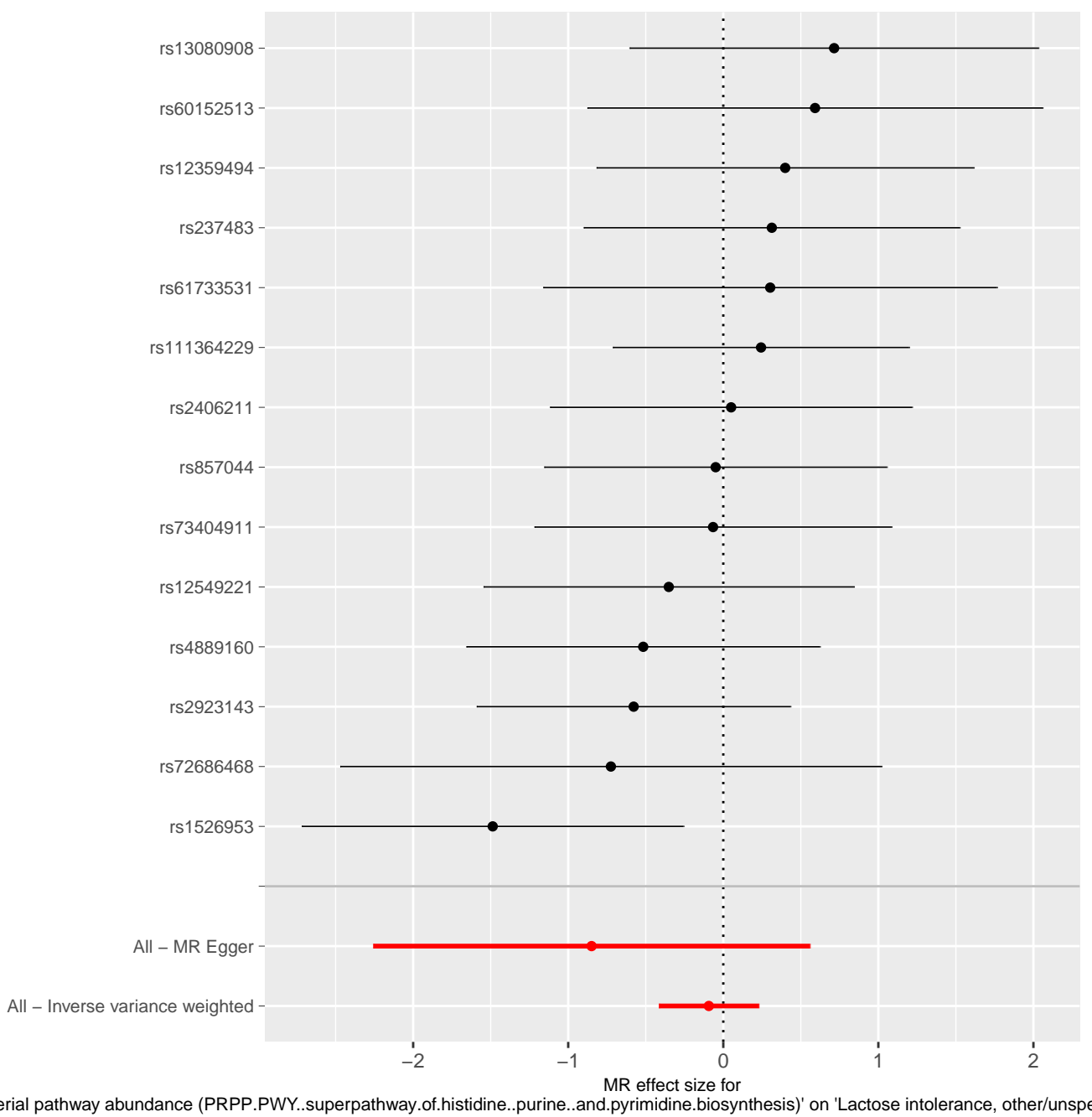

Supplement: Supplementary file 1 [file Data_Sheet_1.zip › supplementary materials/Forward/forest plot/ebi-a-GCST90027513.finngen_R12_E4_LACTONAS.pdf]

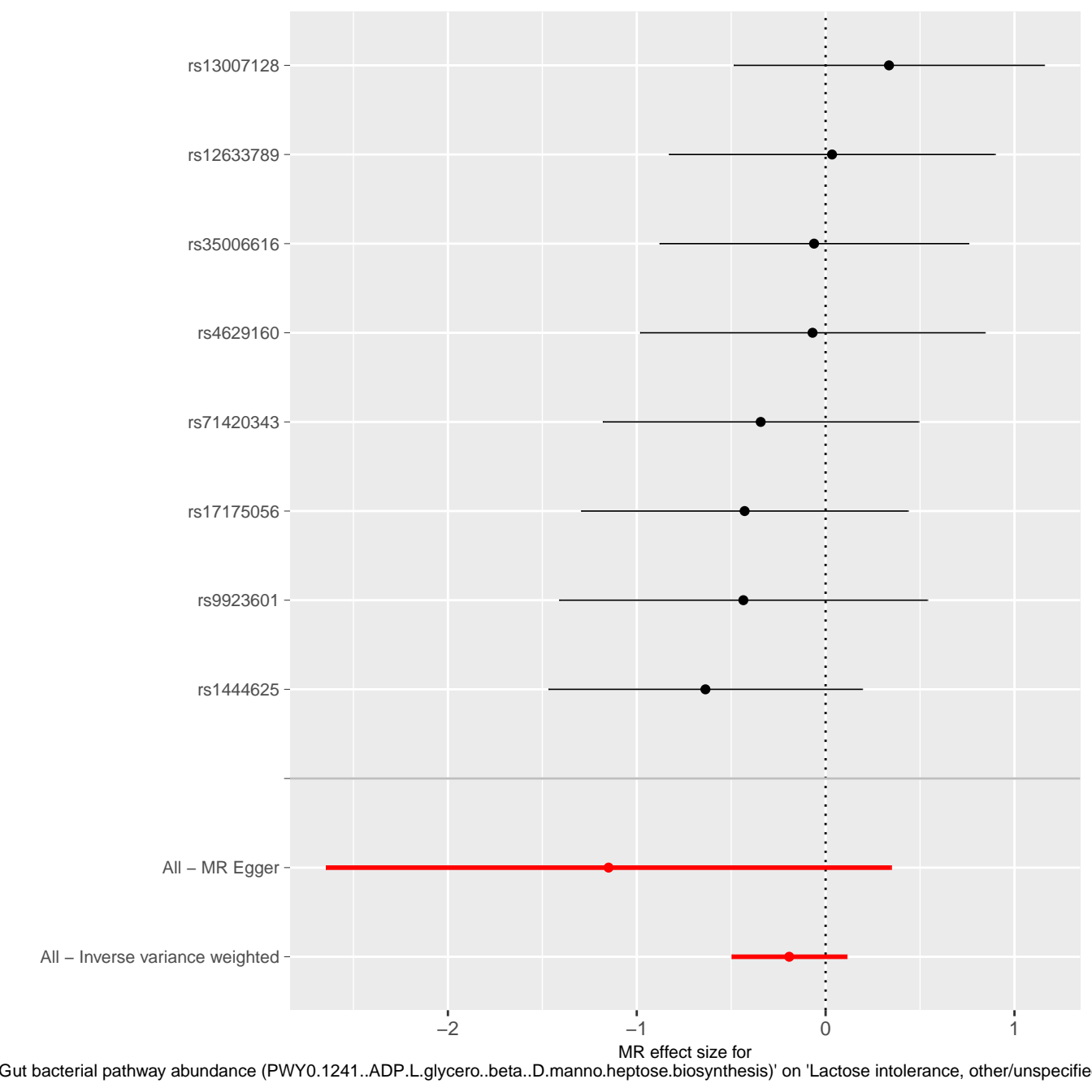

Supplement: Supplementary file 1 [file Data_Sheet_1.zip › supplementary materials/Forward/forest plot/ebi-a-GCST90027515.finngen_R12_E4_LACTONAS.pdf]

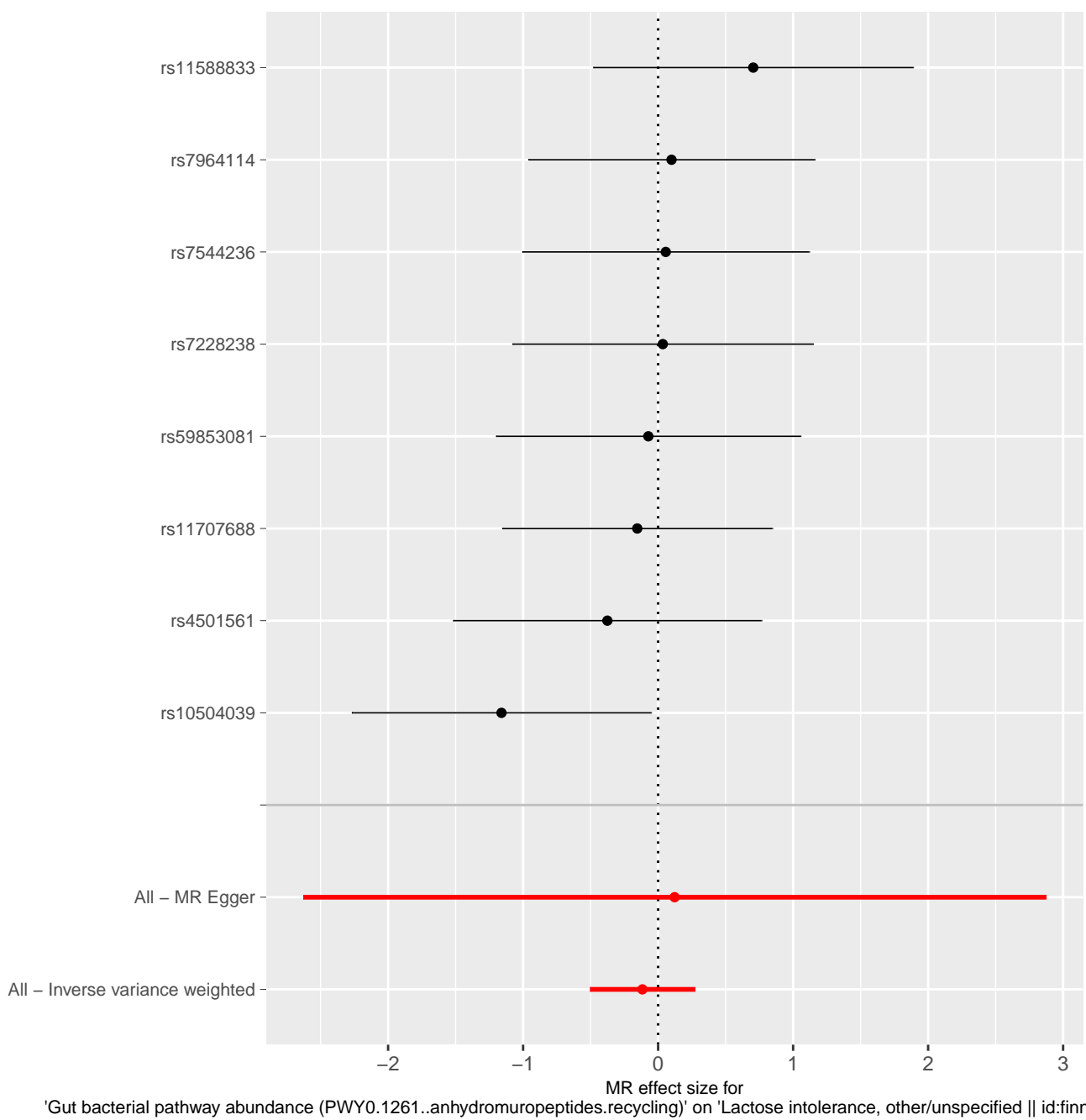

Supplement: Supplementary file 1 [file Data_Sheet_1.zip › supplementary materials/Forward/forest plot/ebi-a-GCST90027516.finngen_R12_E4_LACTONAS.pdf]

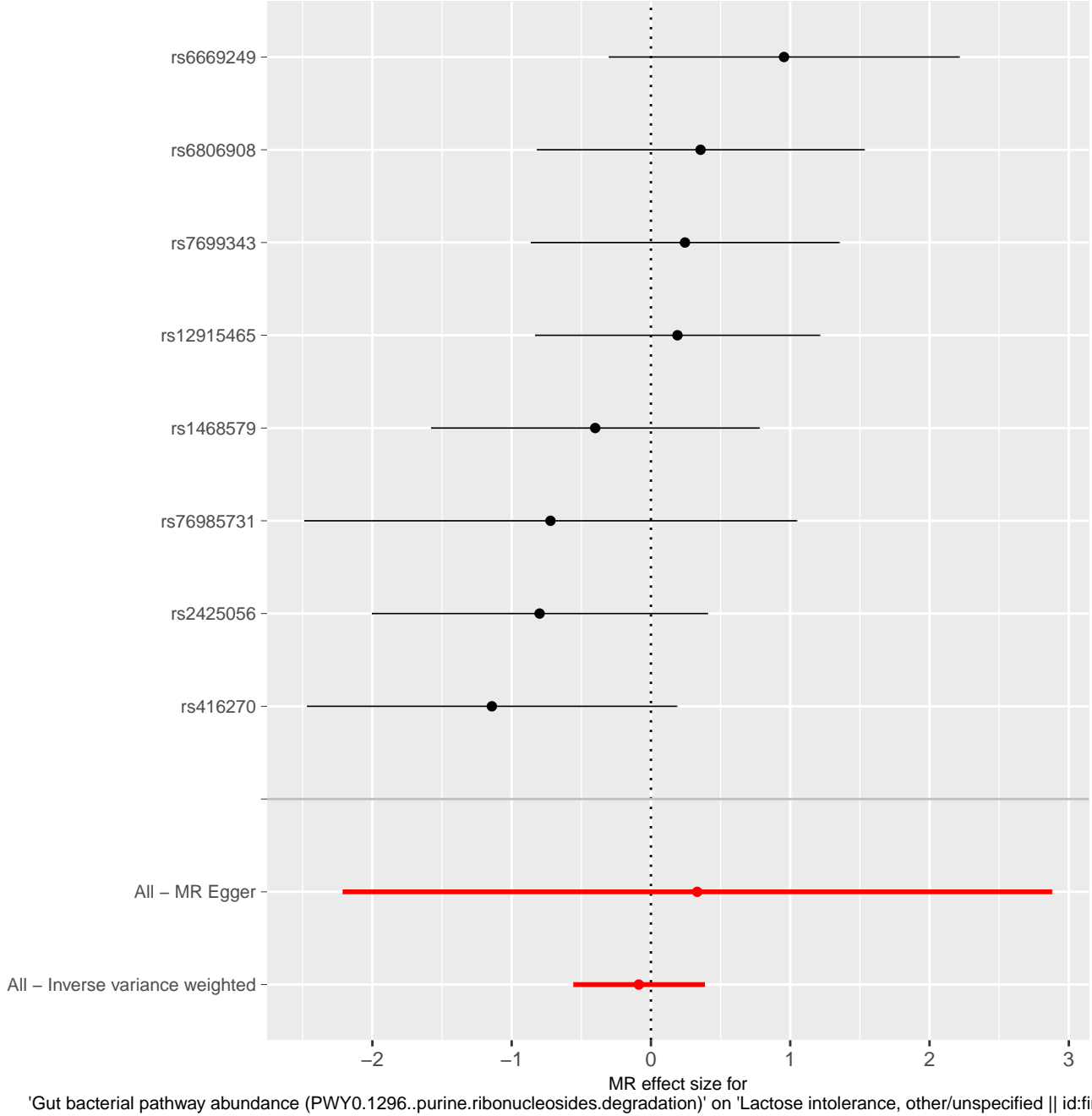

Supplement: Supplementary file 1 [file Data_Sheet_1.zip › supplementary materials/Forward/forest plot/ebi-a-GCST90027517.finngen_R12_E4_LACTONAS.pdf]

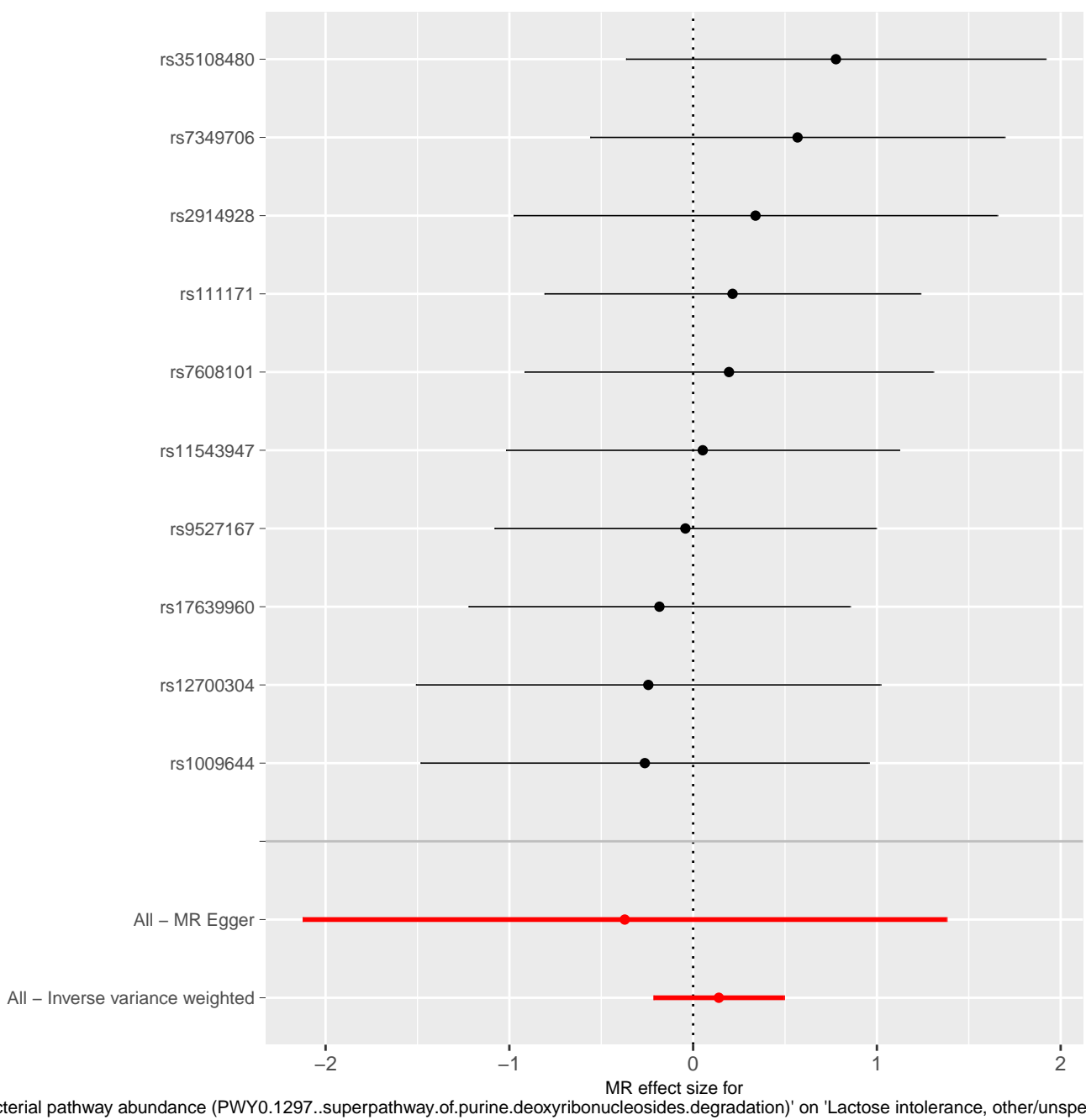

Supplement: Supplementary file 1 [file Data_Sheet_1.zip › supplementary materials/Forward/forest plot/ebi-a-GCST90027518.finngen_R12_E4_LACTONAS.pdf]

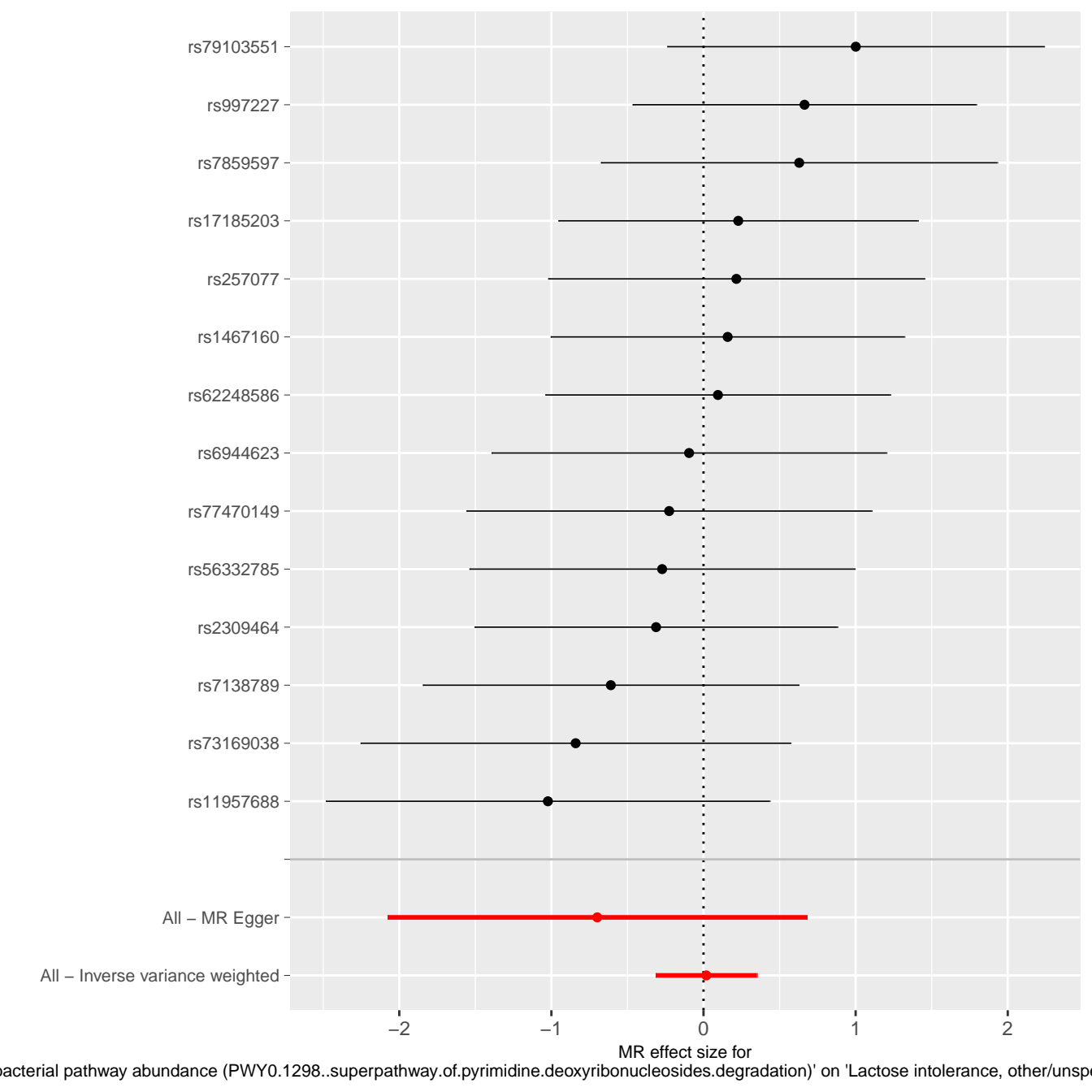

Supplement: Supplementary file 1 [file Data_Sheet_1.zip › supplementary materials/Forward/forest plot/ebi-a-GCST90027519.finngen_R12_E4_LACTONAS.pdf]

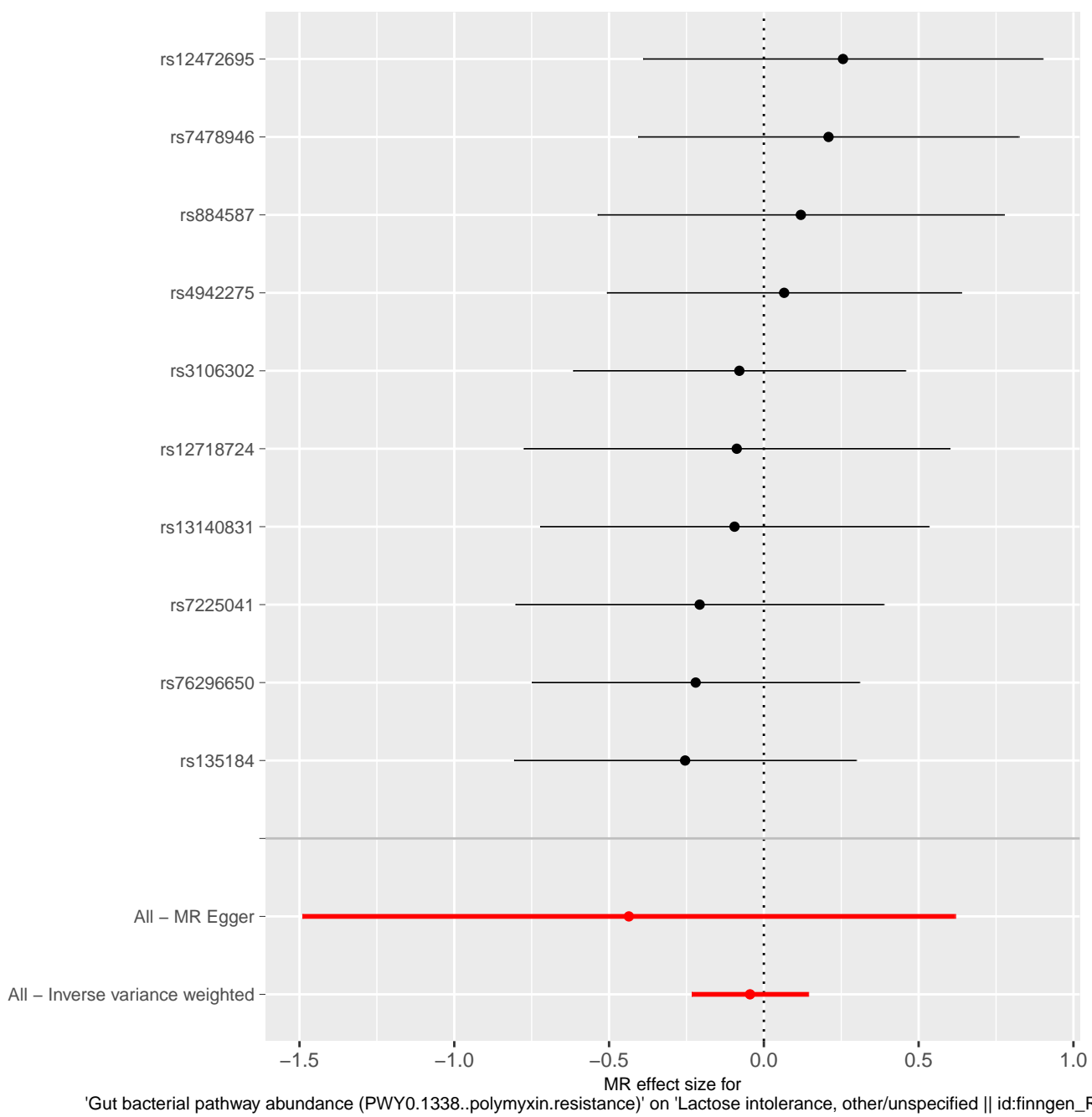

Supplement: Supplementary file 1 [file Data_Sheet_1.zip › supplementary materials/Forward/forest plot/ebi-a-GCST90027520.finngen_R12_E4_LACTONAS.pdf]

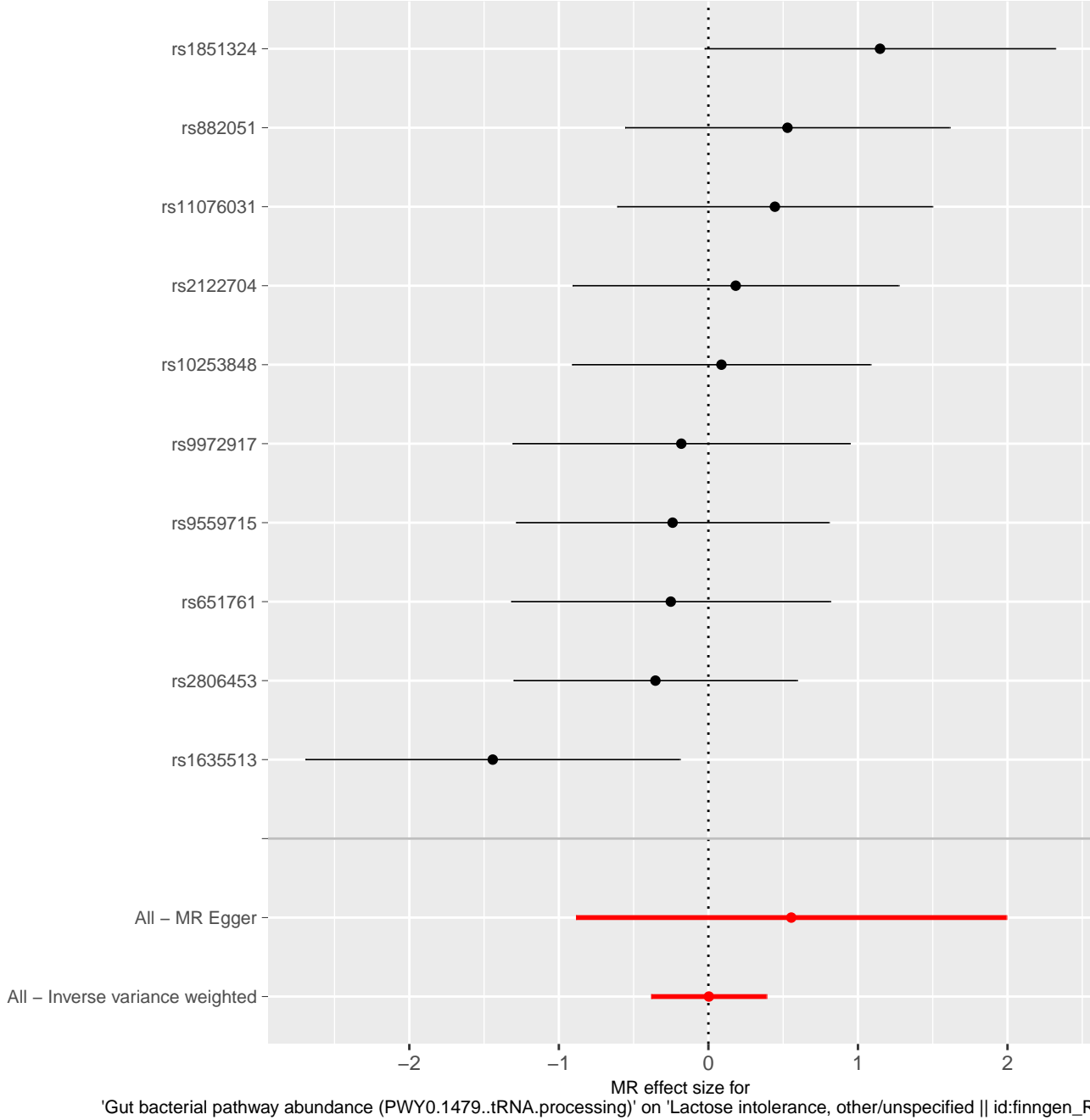

Supplement: Supplementary file 1 [file Data_Sheet_1.zip › supplementary materials/Forward/forest plot/ebi-a-GCST90027522.finngen_R12_E4_LACTONAS.pdf]

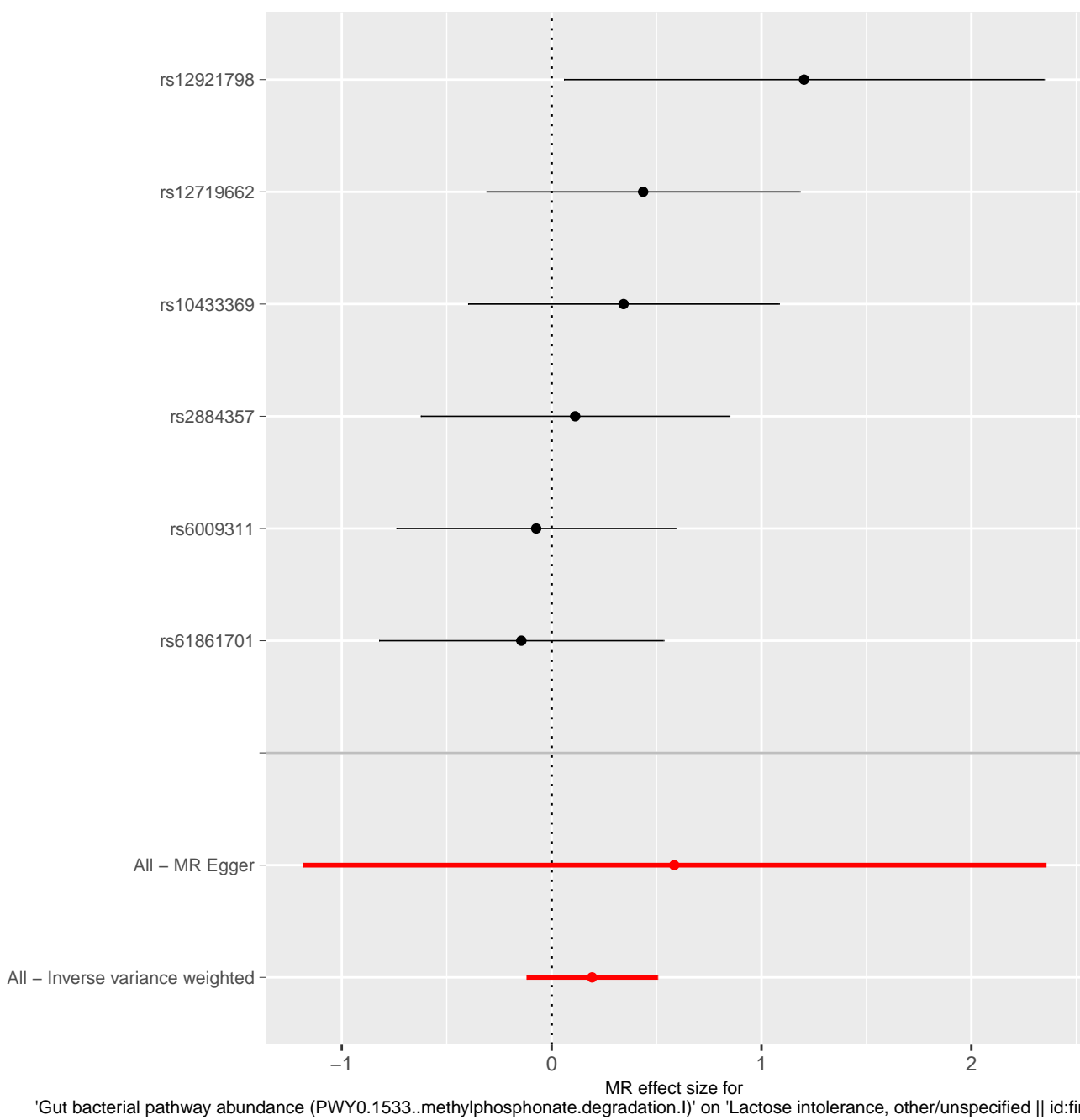

Supplement: Supplementary file 1 [file Data_Sheet_1.zip › supplementary materials/Forward/forest plot/ebi-a-GCST90027523.finngen_R12_E4_LACTONAS.pdf]

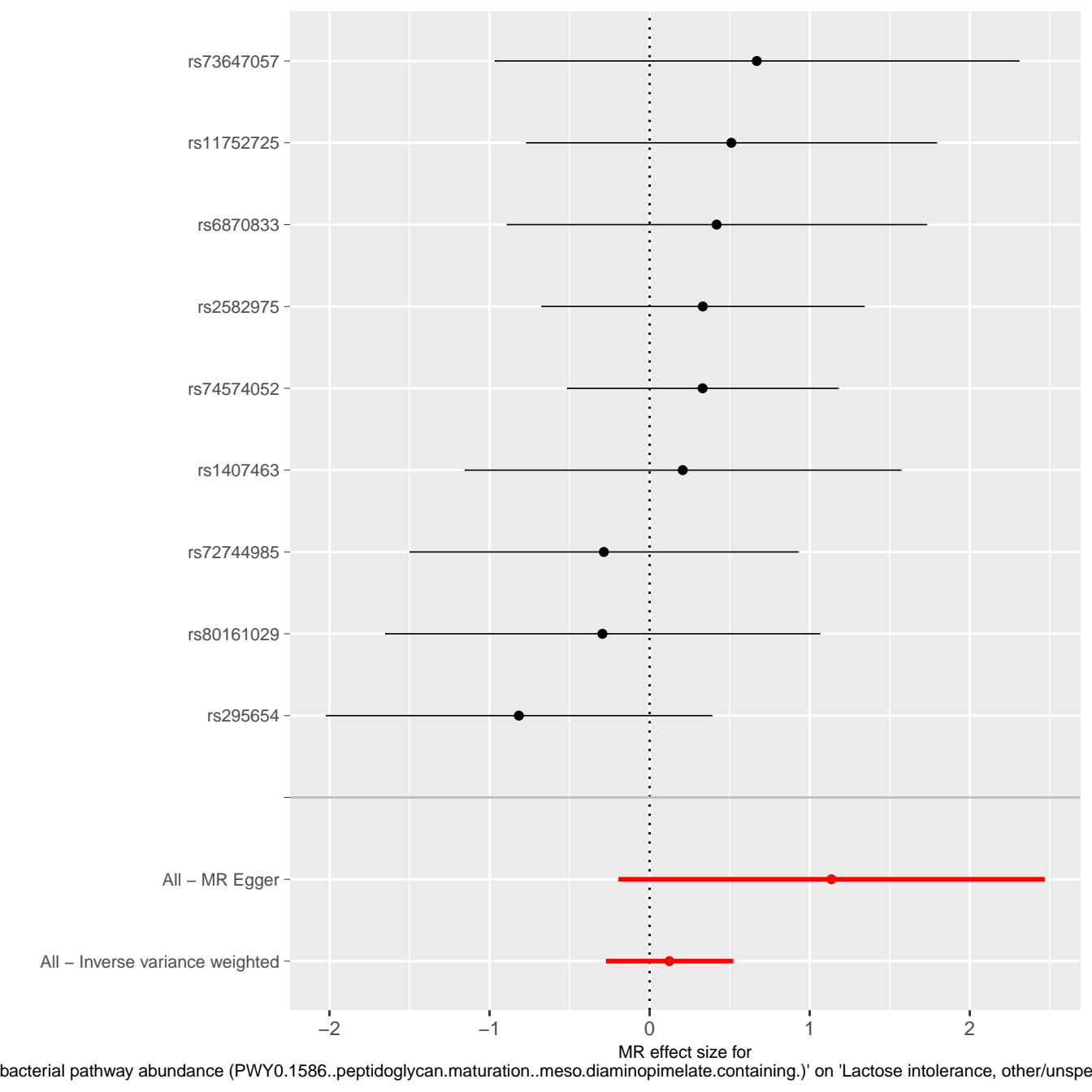

Supplement: Supplementary file 1 [file Data_Sheet_1.zip › supplementary materials/Forward/forest plot/ebi-a-GCST90027524.finngen_R12_E4_LACTONAS.pdf]

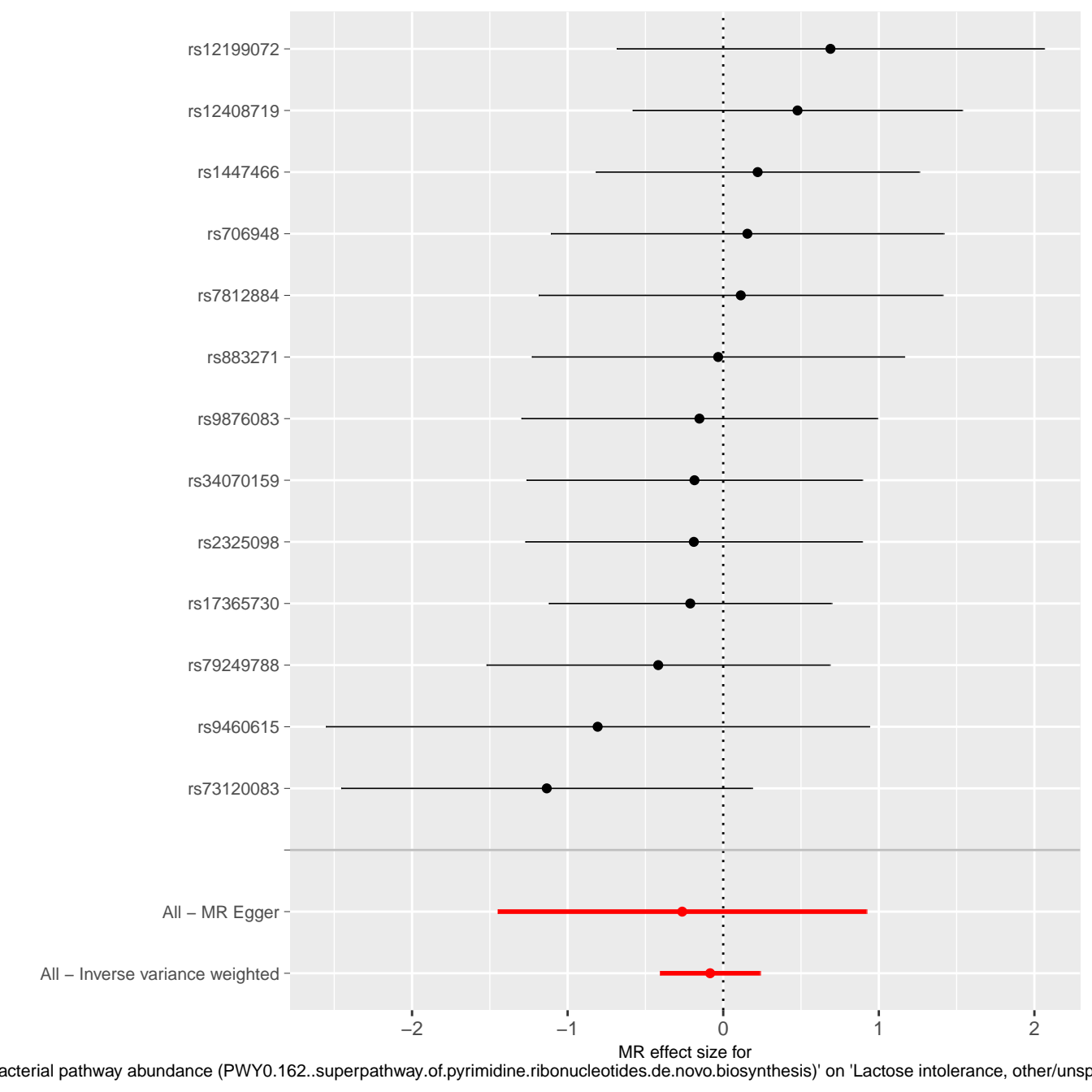

Supplement: Supplementary file 1 [file Data_Sheet_1.zip › supplementary materials/Forward/forest plot/ebi-a-GCST90027525.finngen_R12_E4_LACTONAS.pdf]

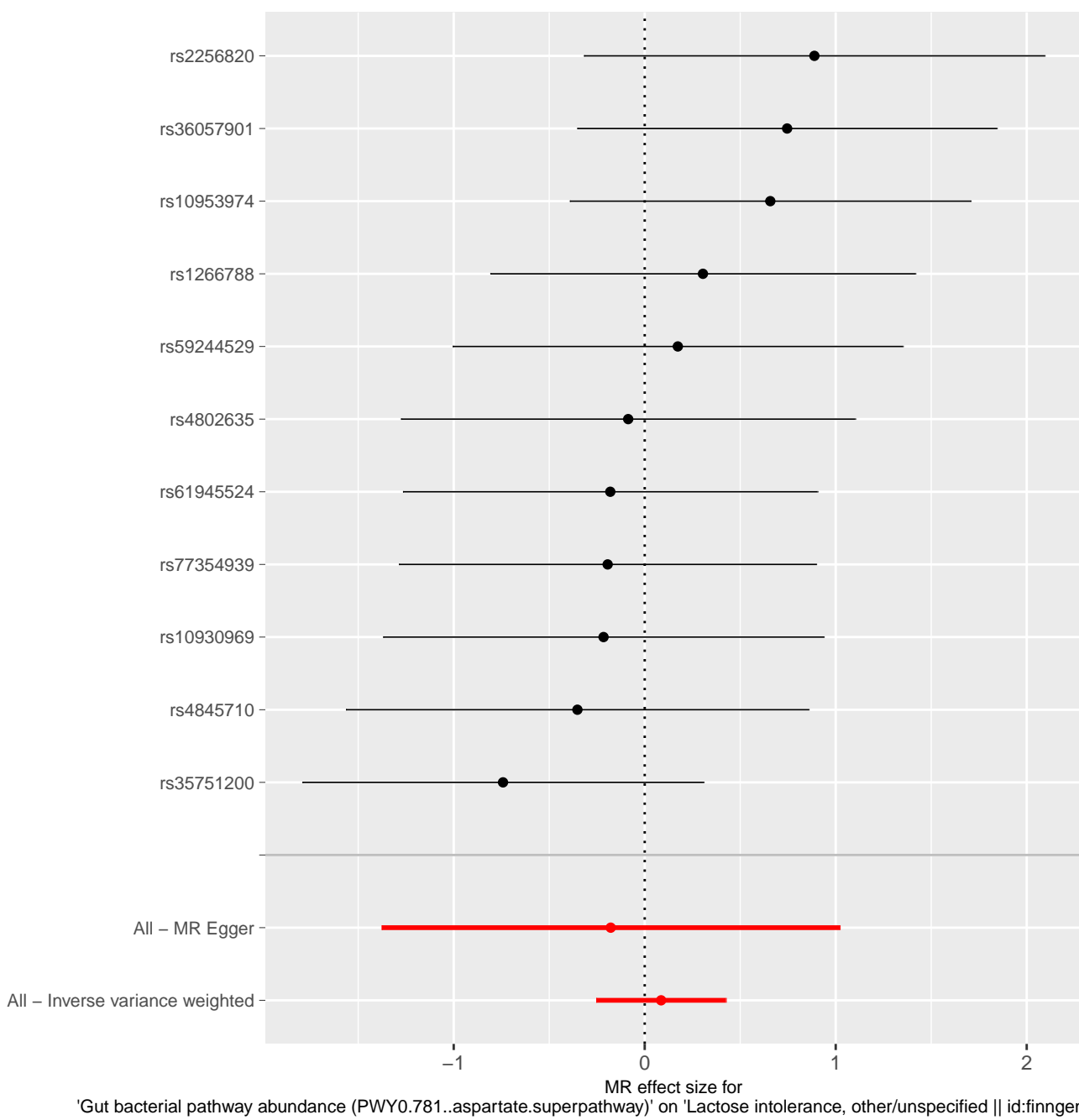

Supplement: Supplementary file 1 [file Data_Sheet_1.zip › supplementary materials/Forward/forest plot/ebi-a-GCST90027526.finngen_R12_E4_LACTONAS.pdf]

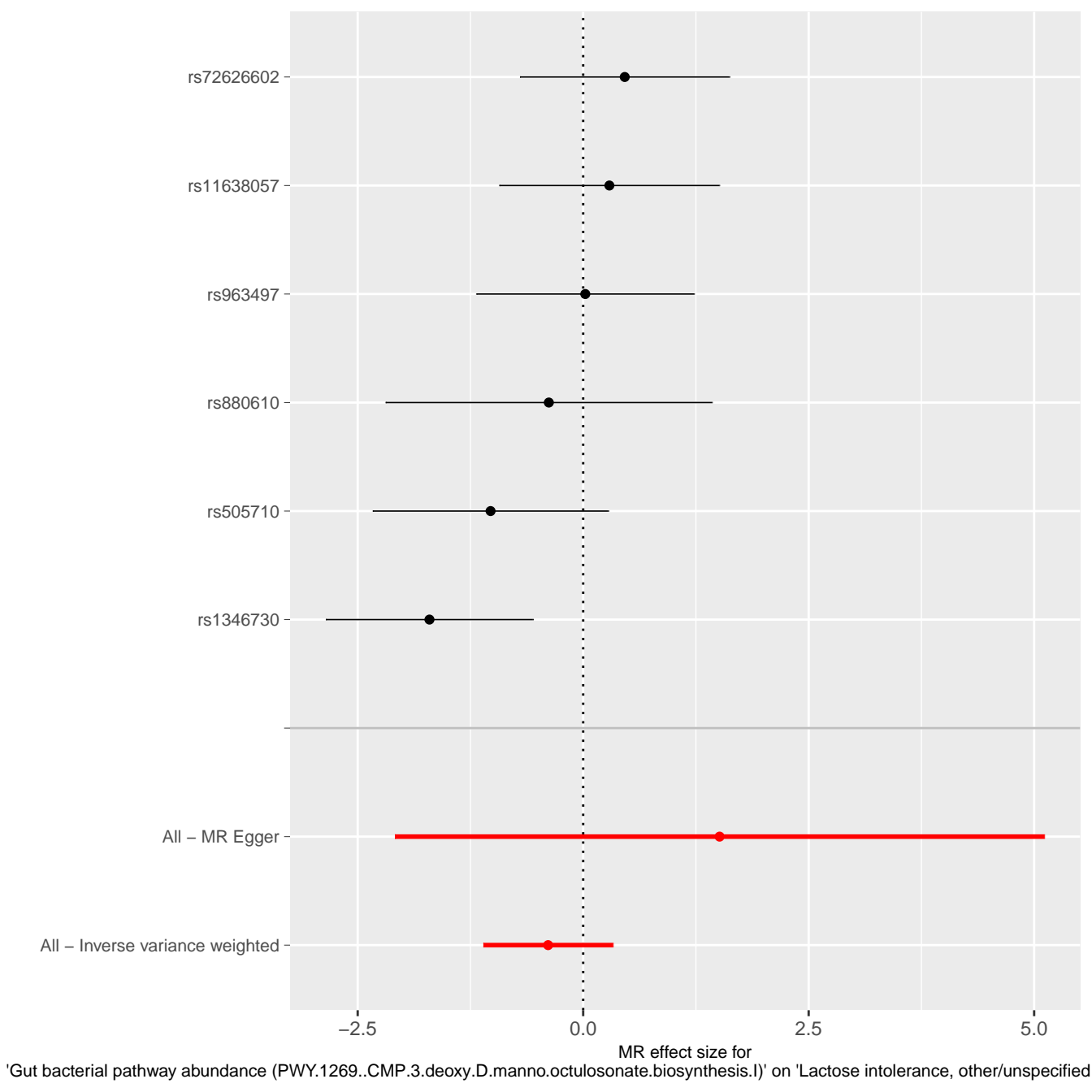

Supplement: Supplementary file 1 [file Data_Sheet_1.zip › supplementary materials/Forward/forest plot/ebi-a-GCST90027528.finngen_R12_E4_LACTONAS.pdf]

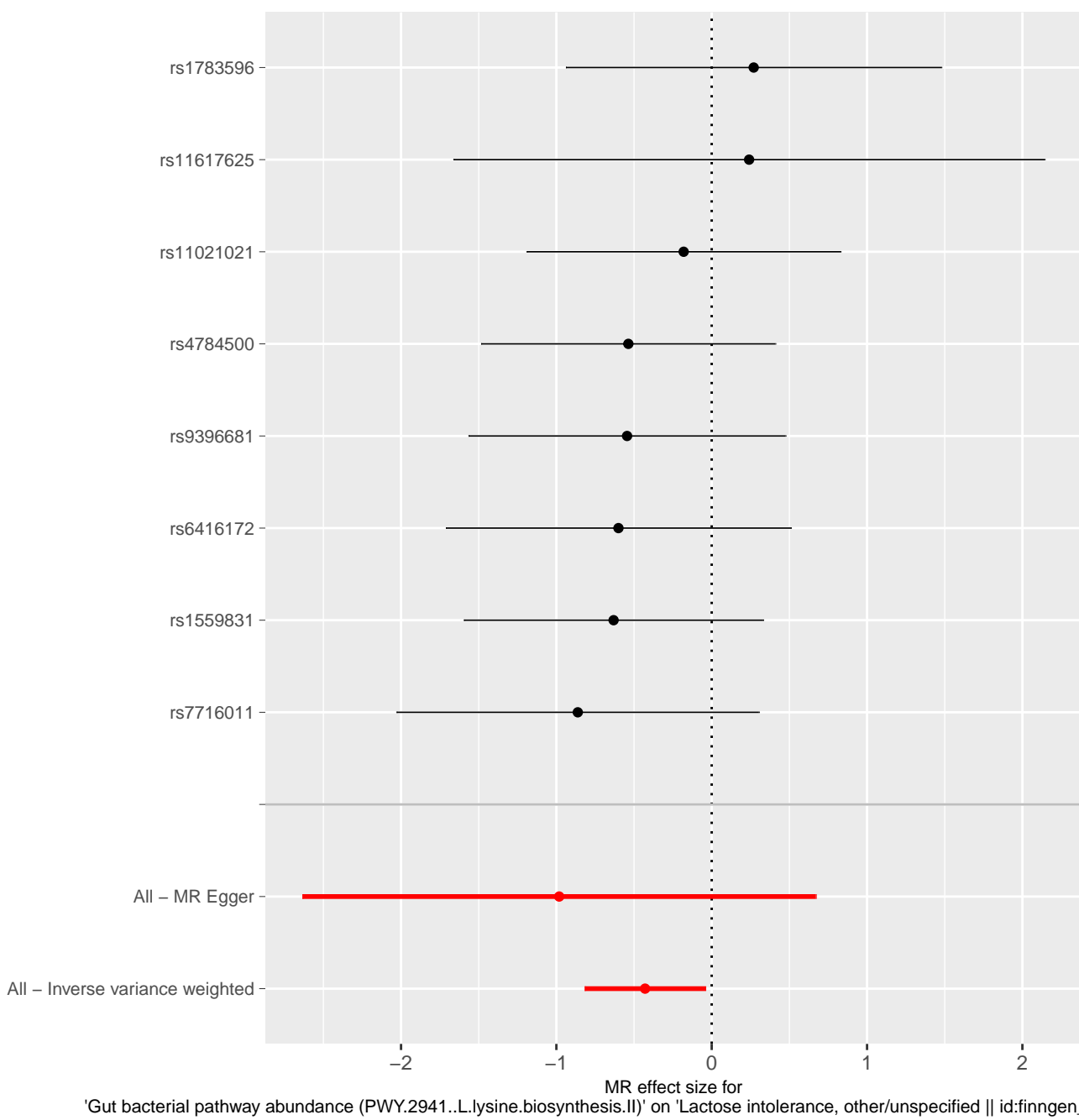

Supplement: Supplementary file 1 [file Data_Sheet_1.zip › supplementary materials/Forward/forest plot/ebi-a-GCST90027529.finngen_R12_E4_LACTONAS.pdf]

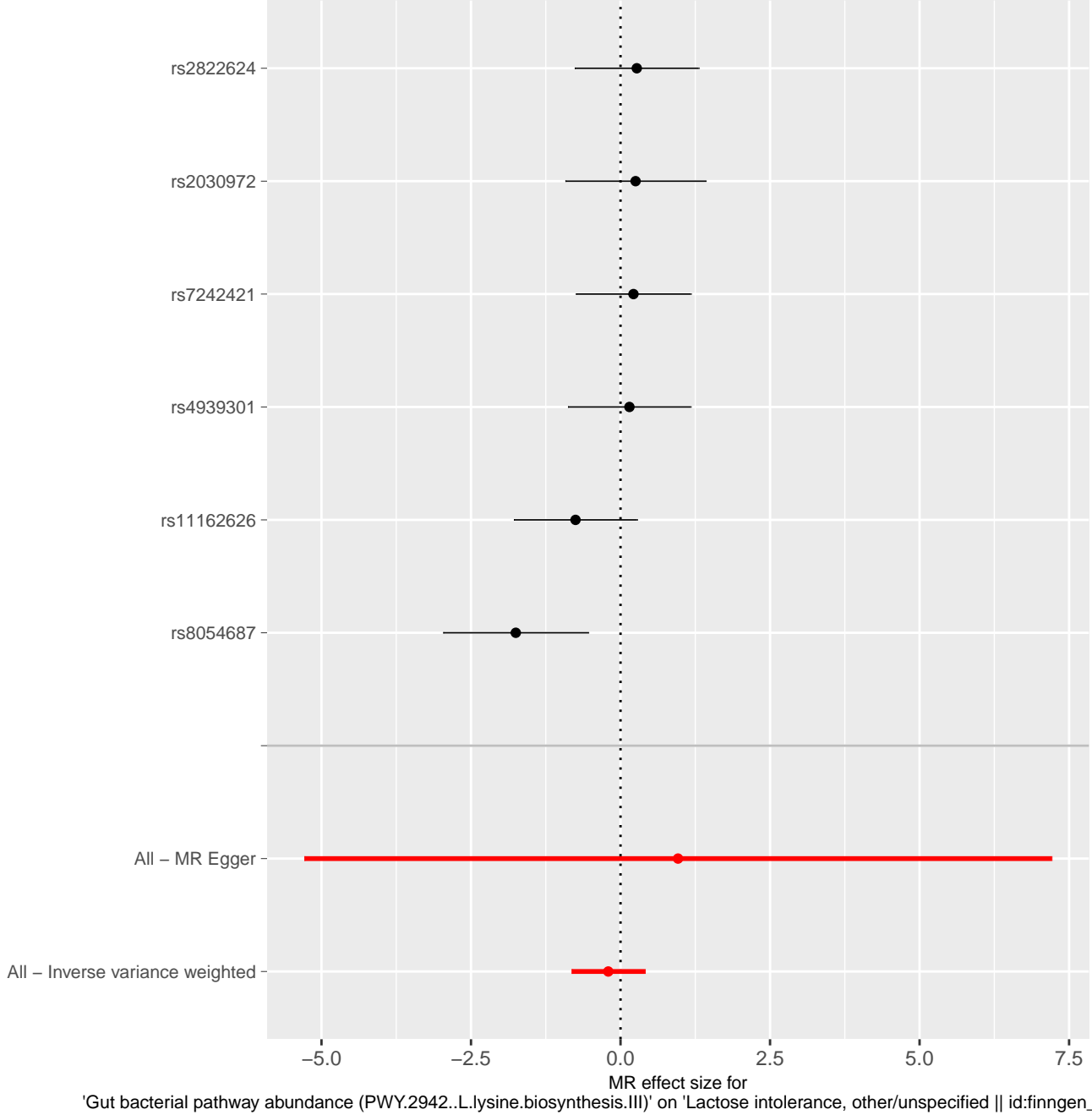

Supplement: Supplementary file 1 [file Data_Sheet_1.zip › supplementary materials/Forward/forest plot/ebi-a-GCST90027530.finngen_R12_E4_LACTONAS.pdf]

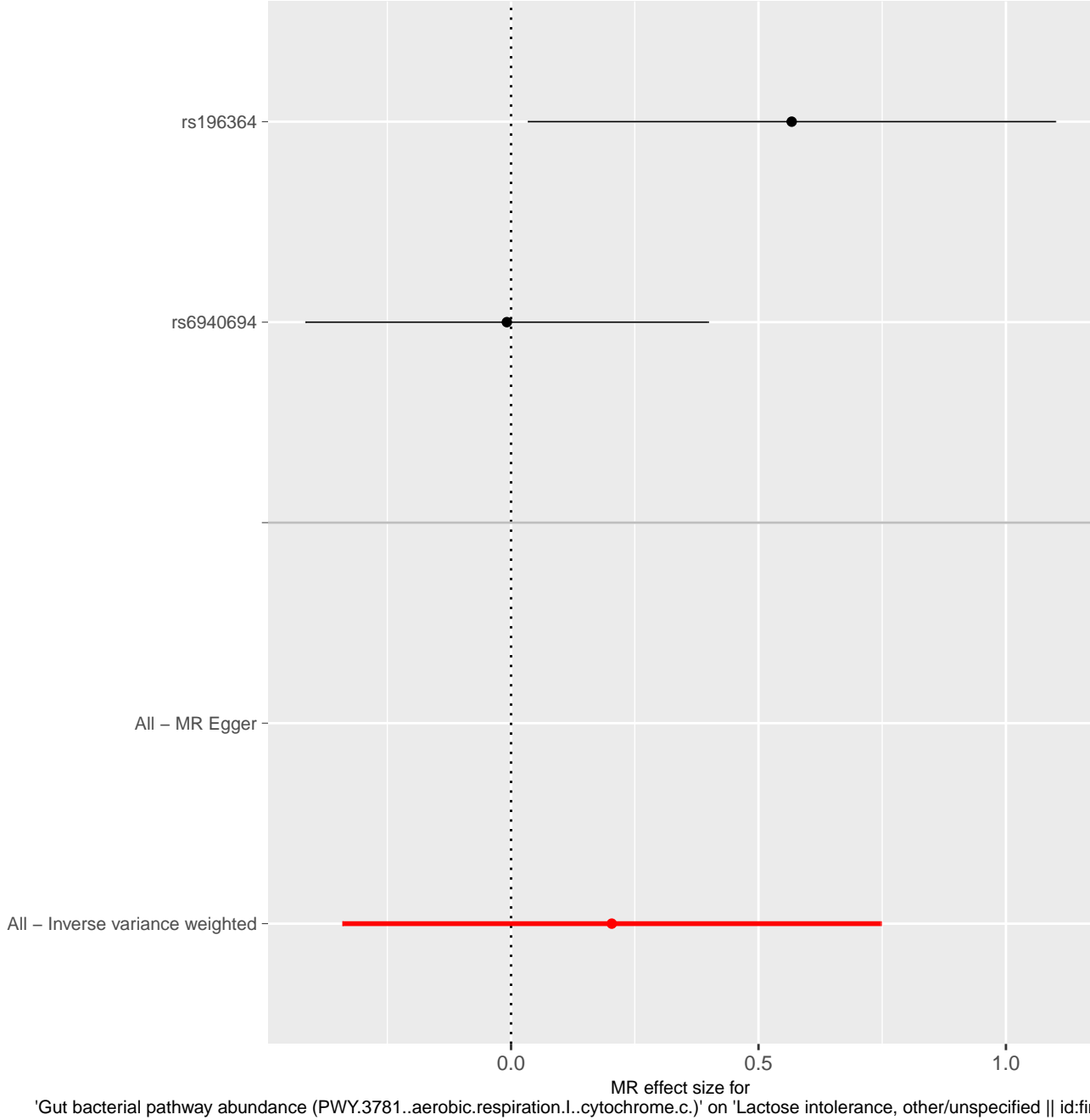

Supplement: Supplementary file 1 [file Data_Sheet_1.zip › supplementary materials/Forward/forest plot/ebi-a-GCST90027532.finngen_R12_E4_LACTONAS.pdf]

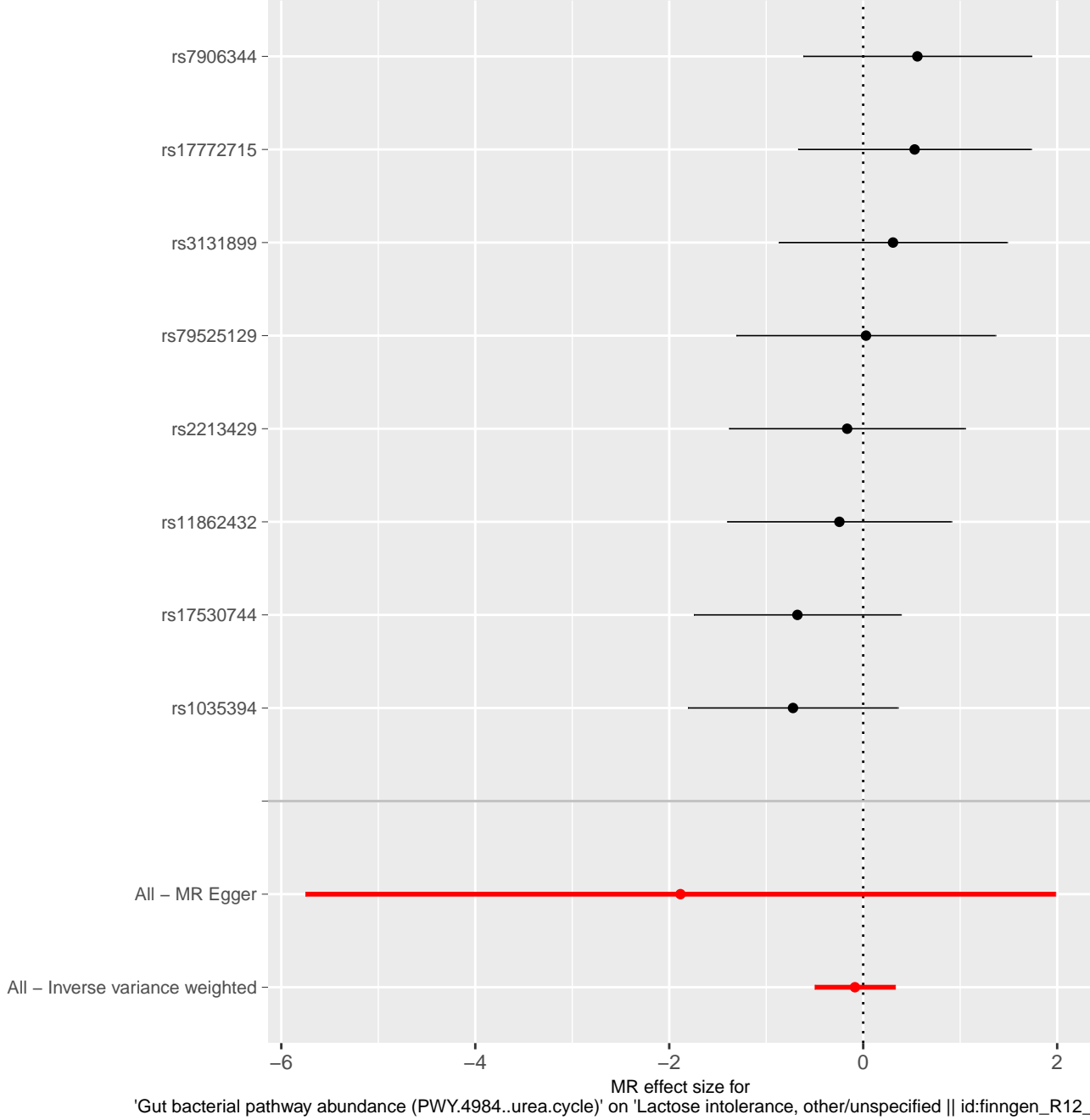

Supplement: Supplementary file 1 [file Data_Sheet_1.zip › supplementary materials/Forward/forest plot/ebi-a-GCST90027534.finngen_R12_E4_LACTONAS.pdf]

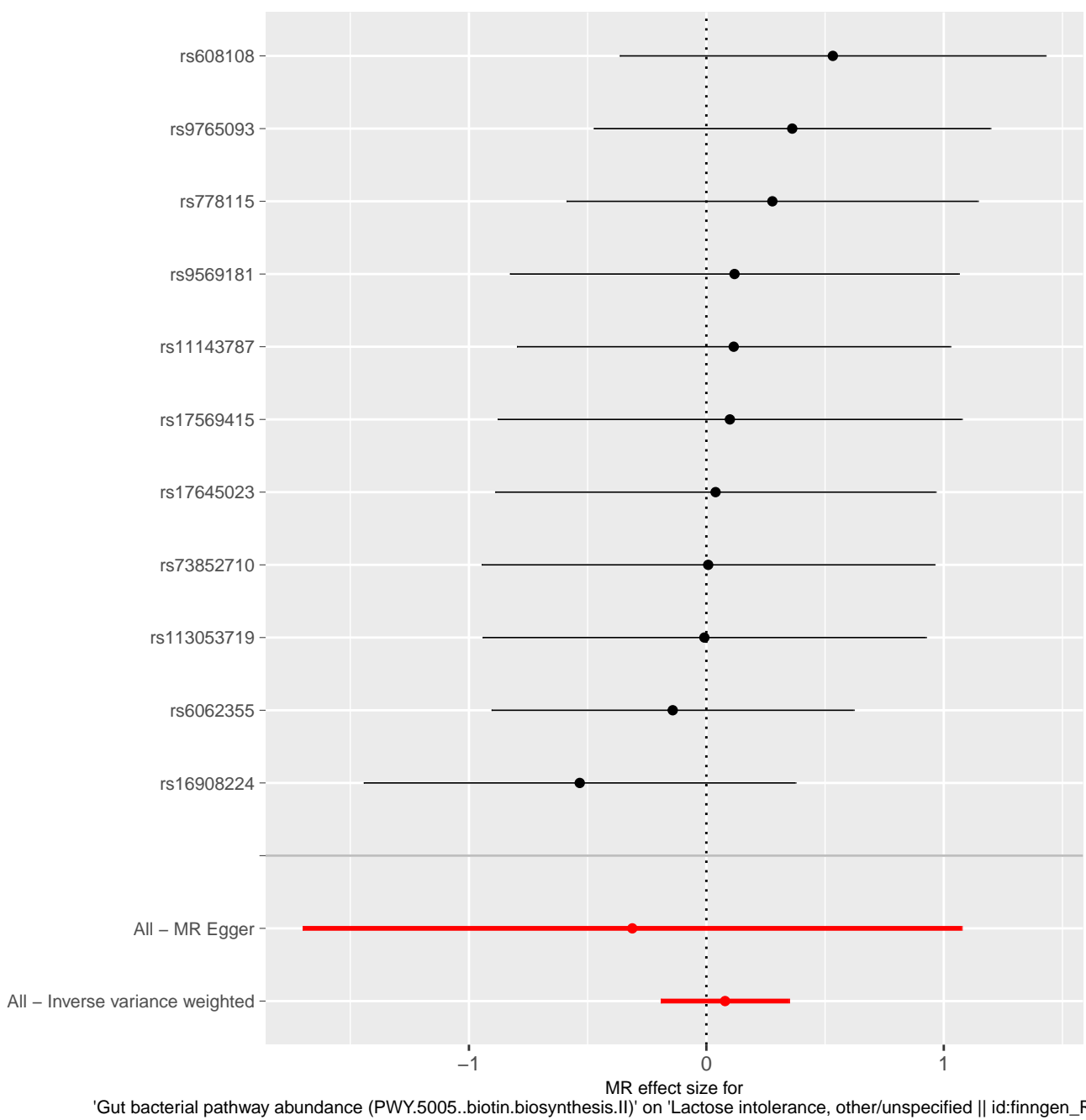

Supplement: Supplementary file 1 [file Data_Sheet_1.zip › supplementary materials/Forward/forest plot/ebi-a-GCST90027536.finngen_R12_E4_LACTONAS.pdf]

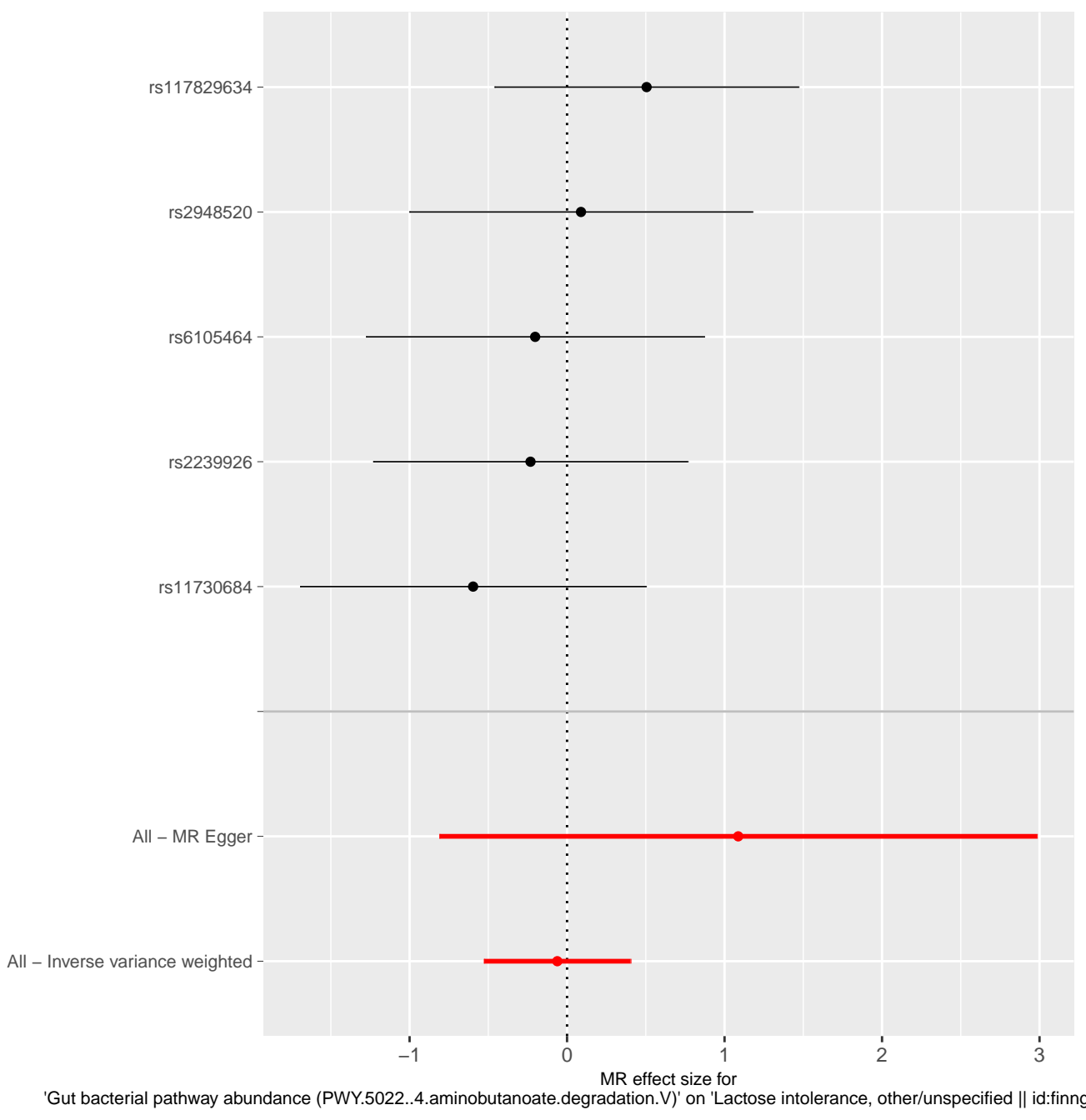

Supplement: Supplementary file 1 [file Data_Sheet_1.zip › supplementary materials/Forward/forest plot/ebi-a-GCST90027537.finngen_R12_E4_LACTONAS.pdf]

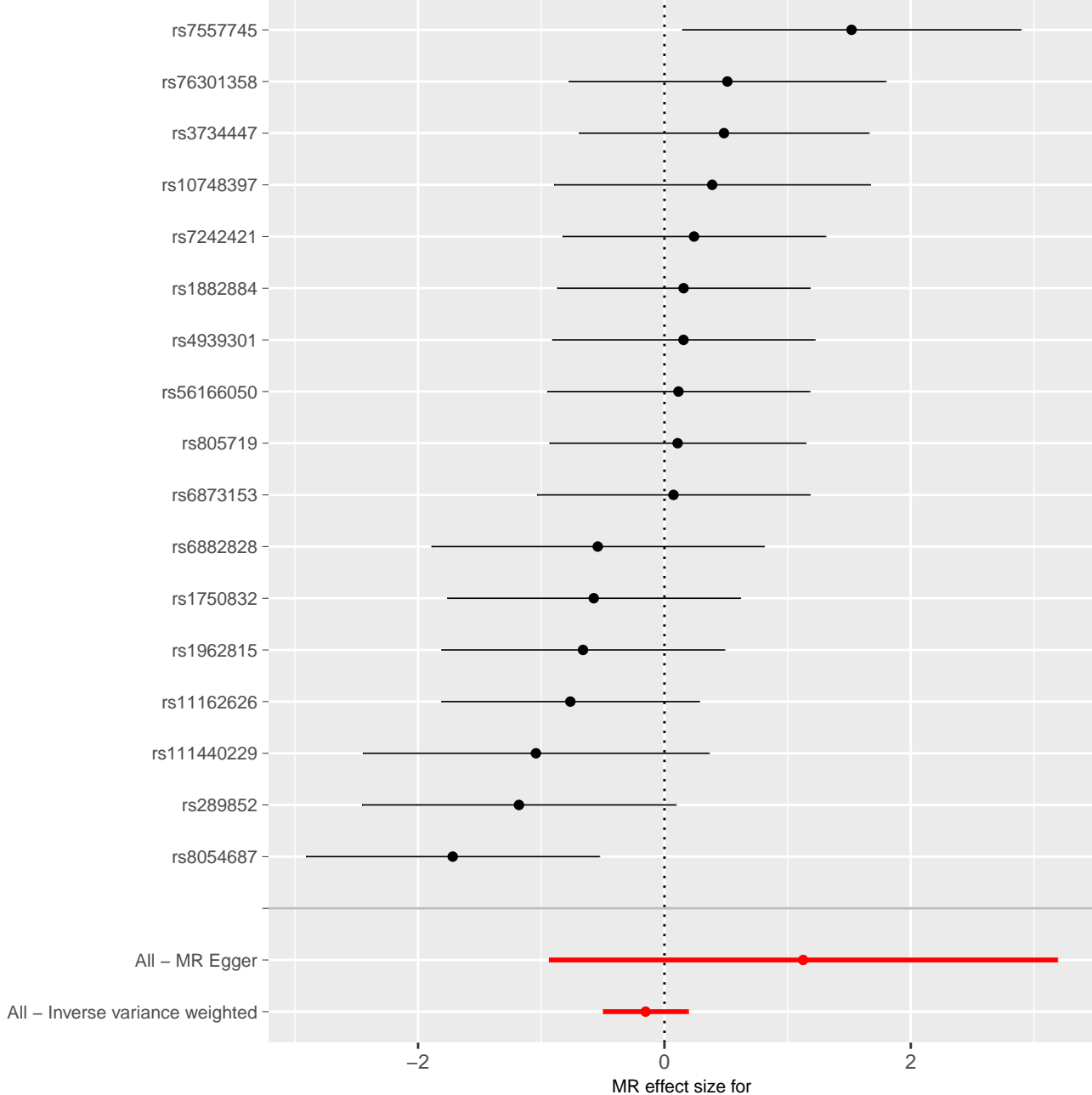

Supplement: Supplementary file 1 [file Data_Sheet_1.zip › supplementary materials/Forward/forest plot/ebi-a-GCST90027539.finngen_R12_E4_LACTONAS.pdf]

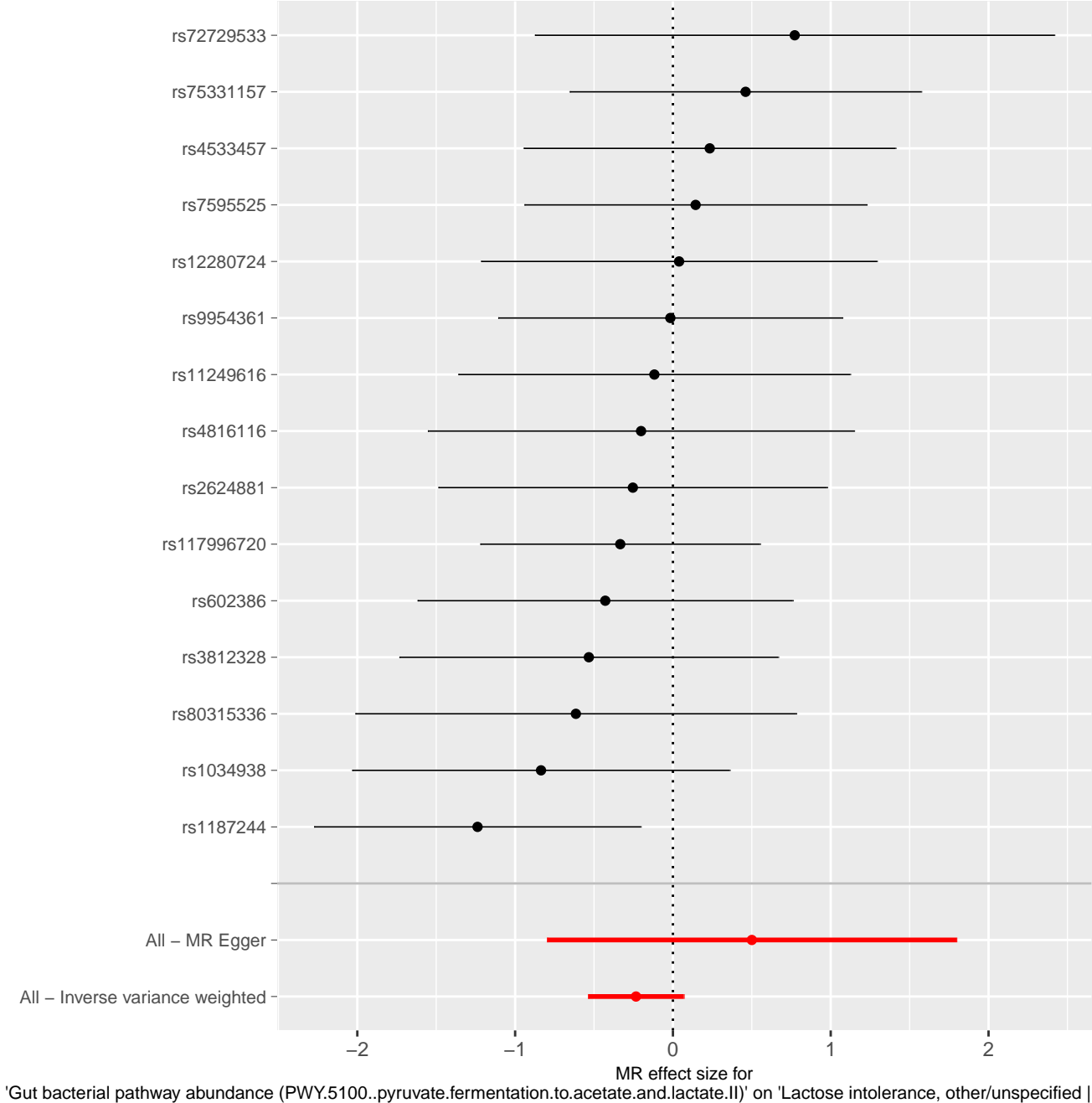

Supplement: Supplementary file 1 [file Data_Sheet_1.zip › supplementary materials/Forward/forest plot/ebi-a-GCST90027540.finngen_R12_E4_LACTONAS.pdf]

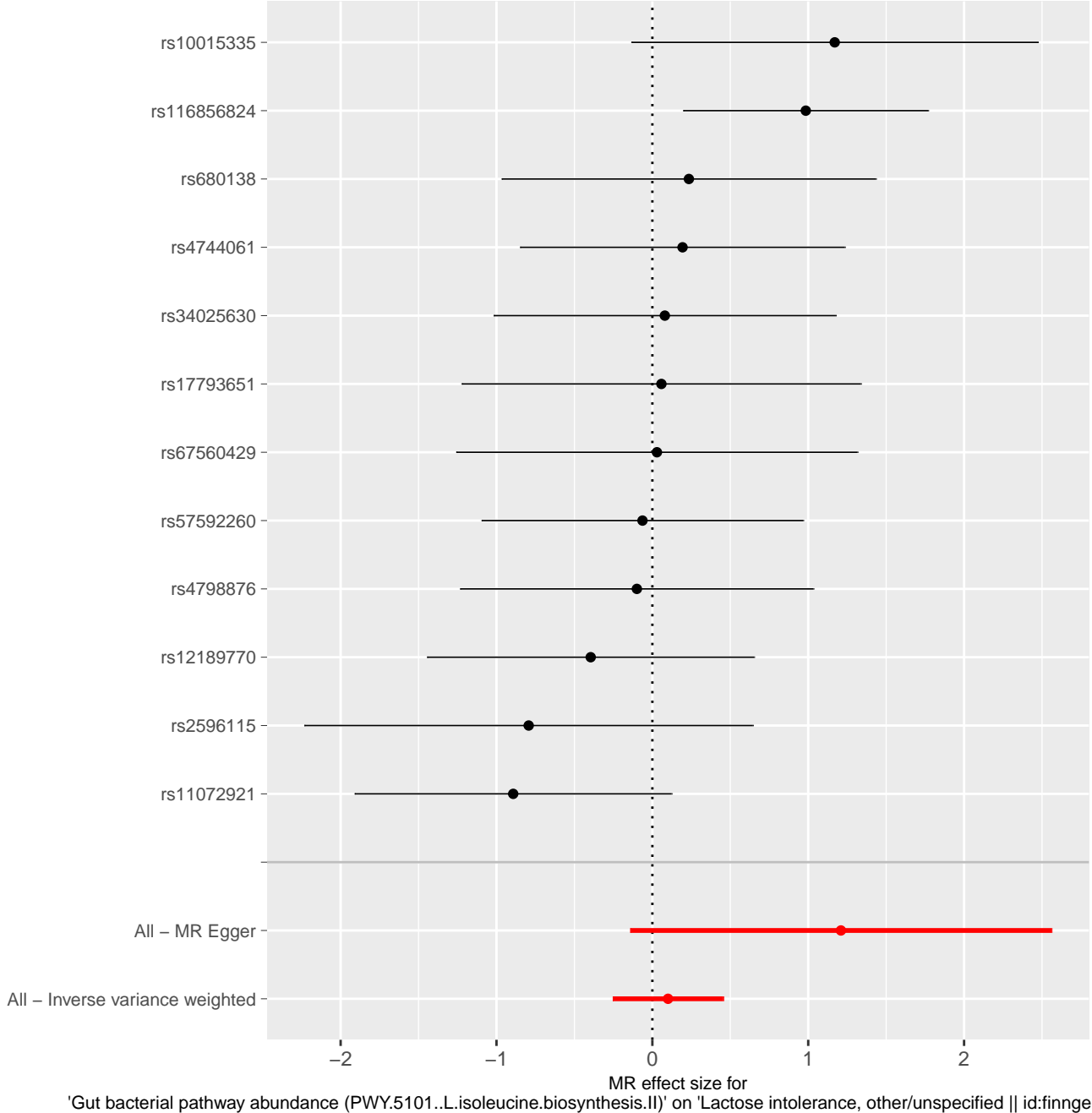

Supplement: Supplementary file 1 [file Data_Sheet_1.zip › supplementary materials/Forward/forest plot/ebi-a-GCST90027541.finngen_R12_E4_LACTONAS.pdf]

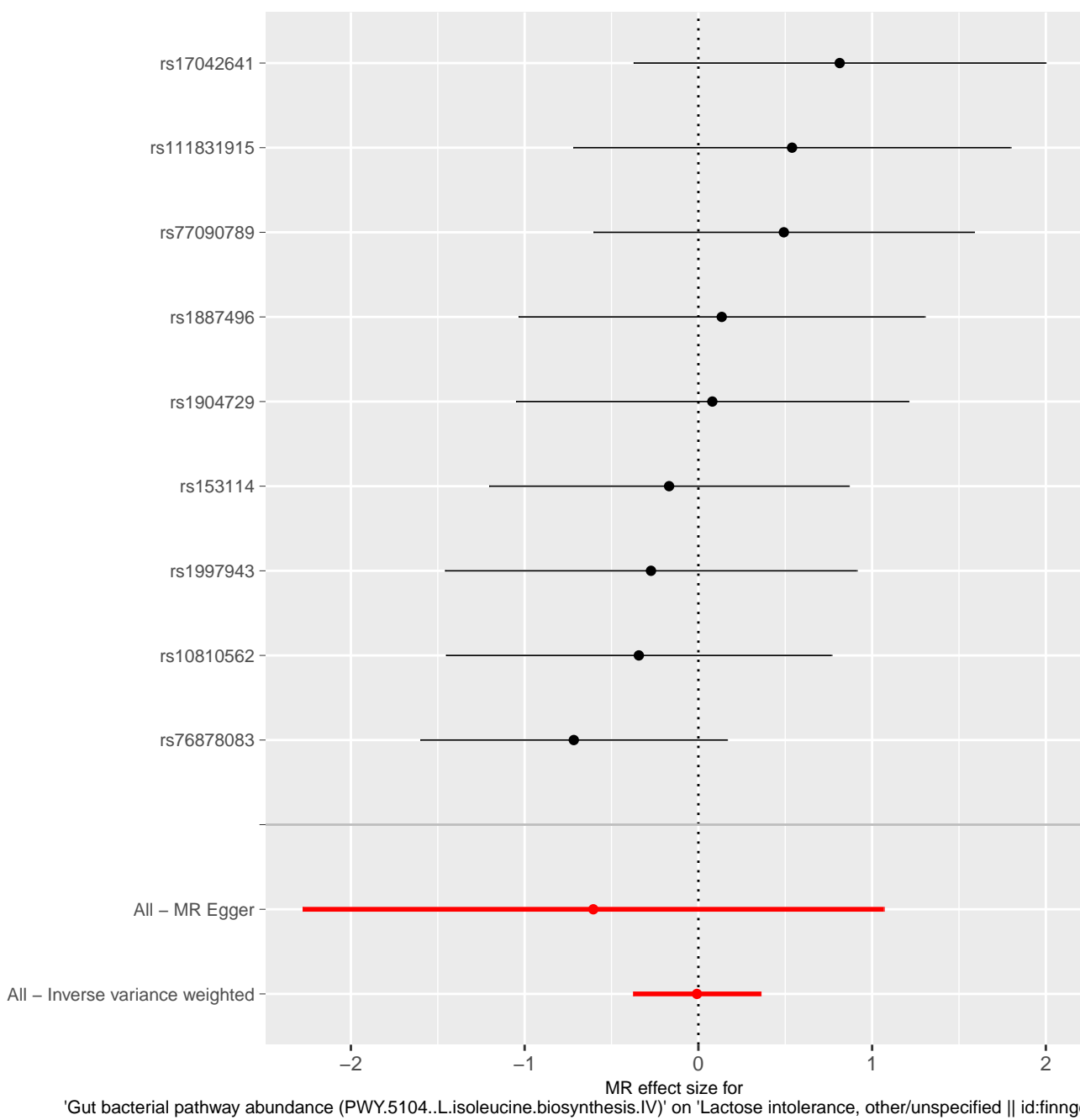

Supplement: Supplementary file 1 [file Data_Sheet_1.zip › supplementary materials/Forward/forest plot/ebi-a-GCST90027542.finngen_R12_E4_LACTONAS.pdf]

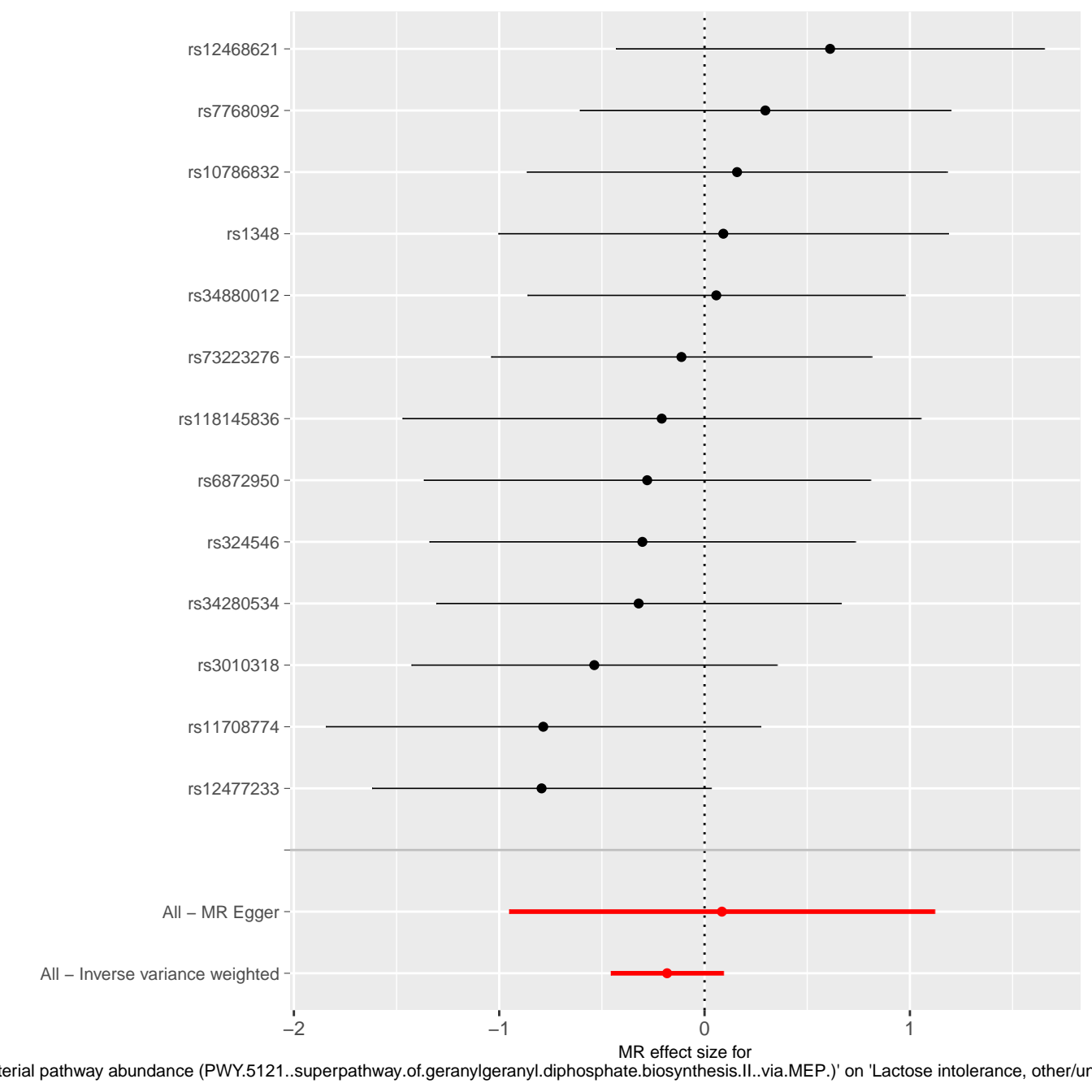

Supplement: Supplementary file 1 [file Data_Sheet_1.zip › supplementary materials/Forward/forest plot/ebi-a-GCST90027543.finngen_R12_E4_LACTONAS.pdf]

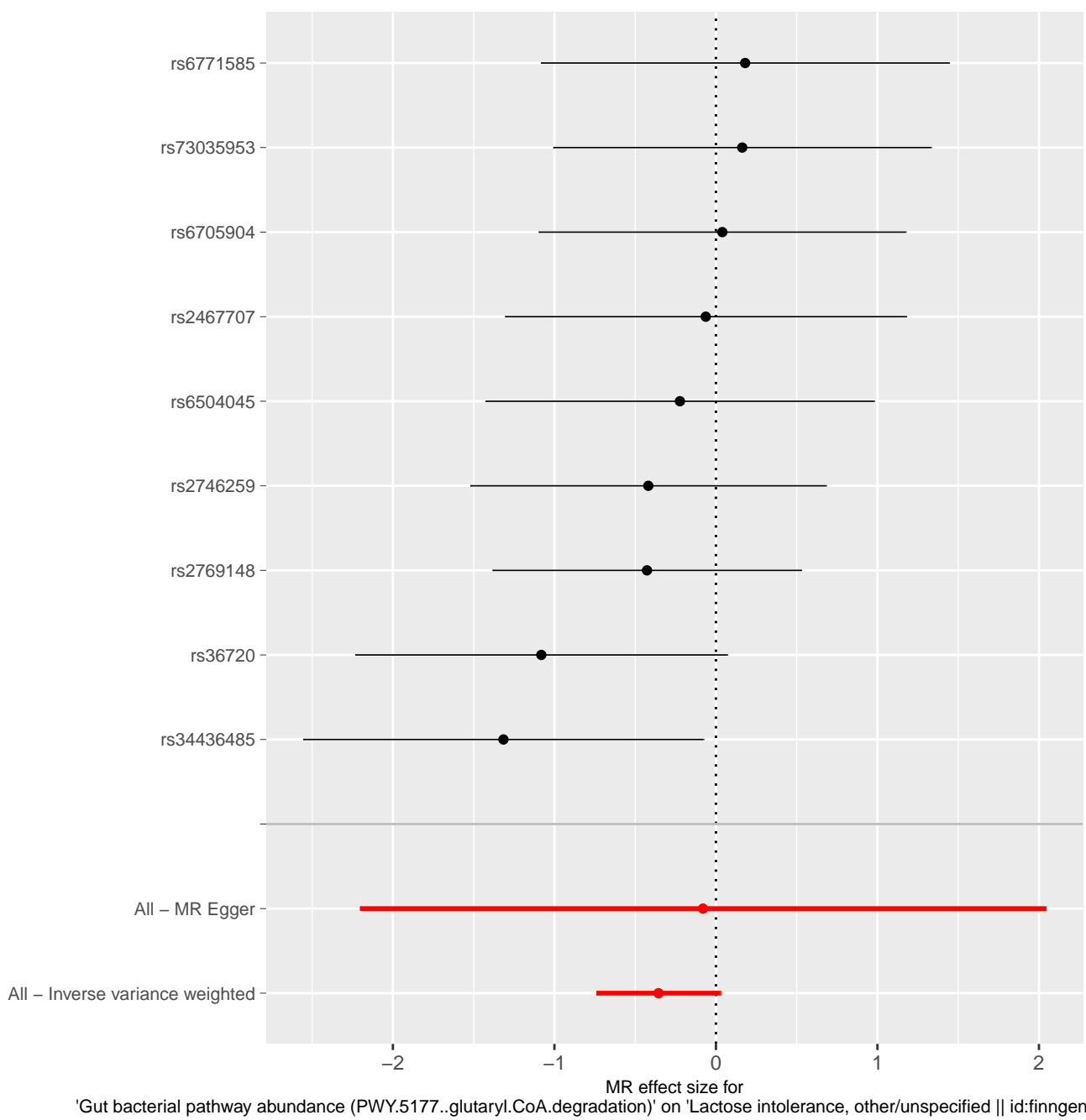

Supplement: Supplementary file 1 [file Data_Sheet_1.zip › supplementary materials/Forward/forest plot/ebi-a-GCST90027545.finngen_R12_E4_LACTONAS.pdf]

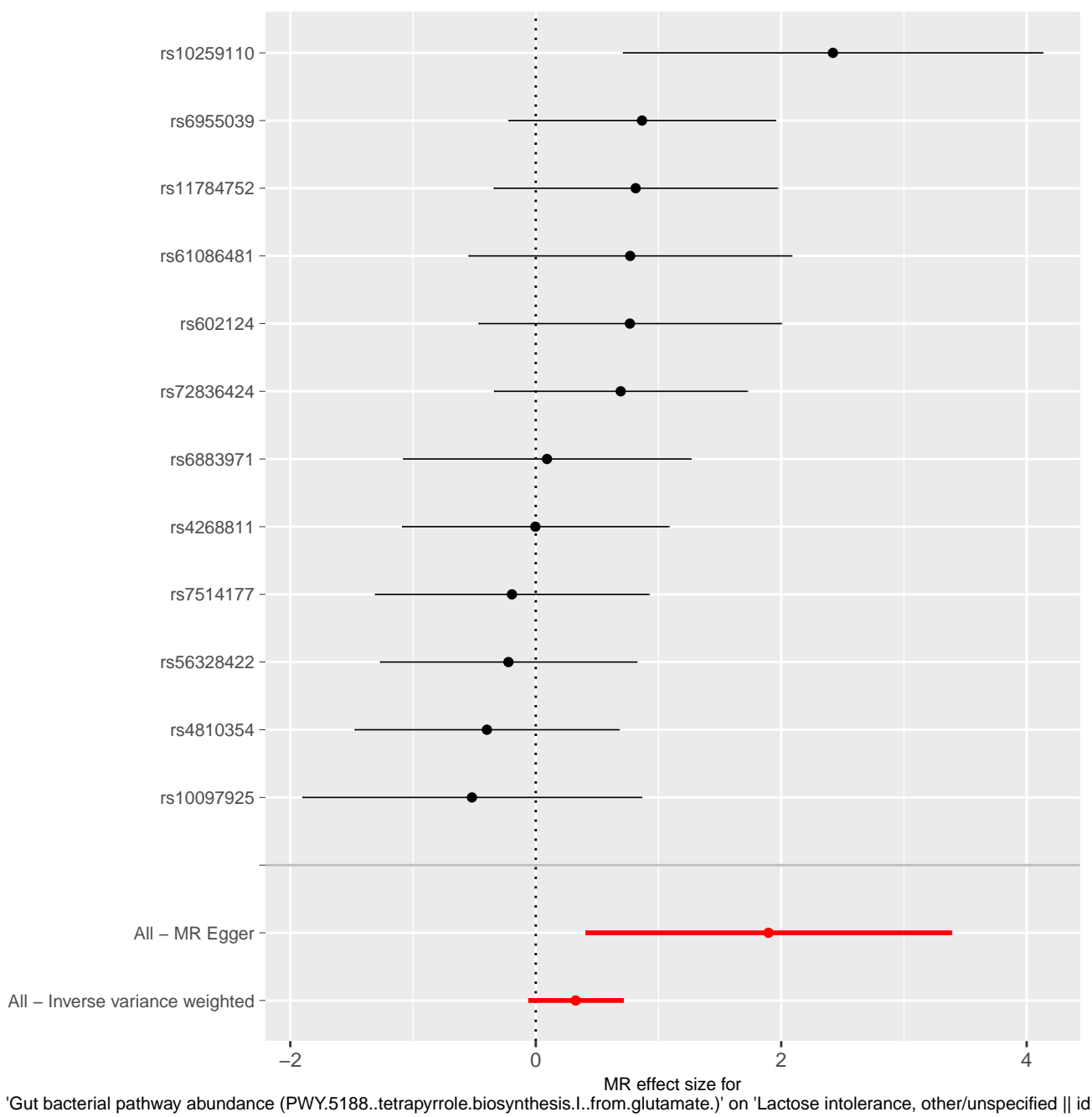

Supplement: Supplementary file 1 [file Data_Sheet_1.zip › supplementary materials/Forward/forest plot/ebi-a-GCST90027546.finngen_R12_E4_LACTONAS.pdf]

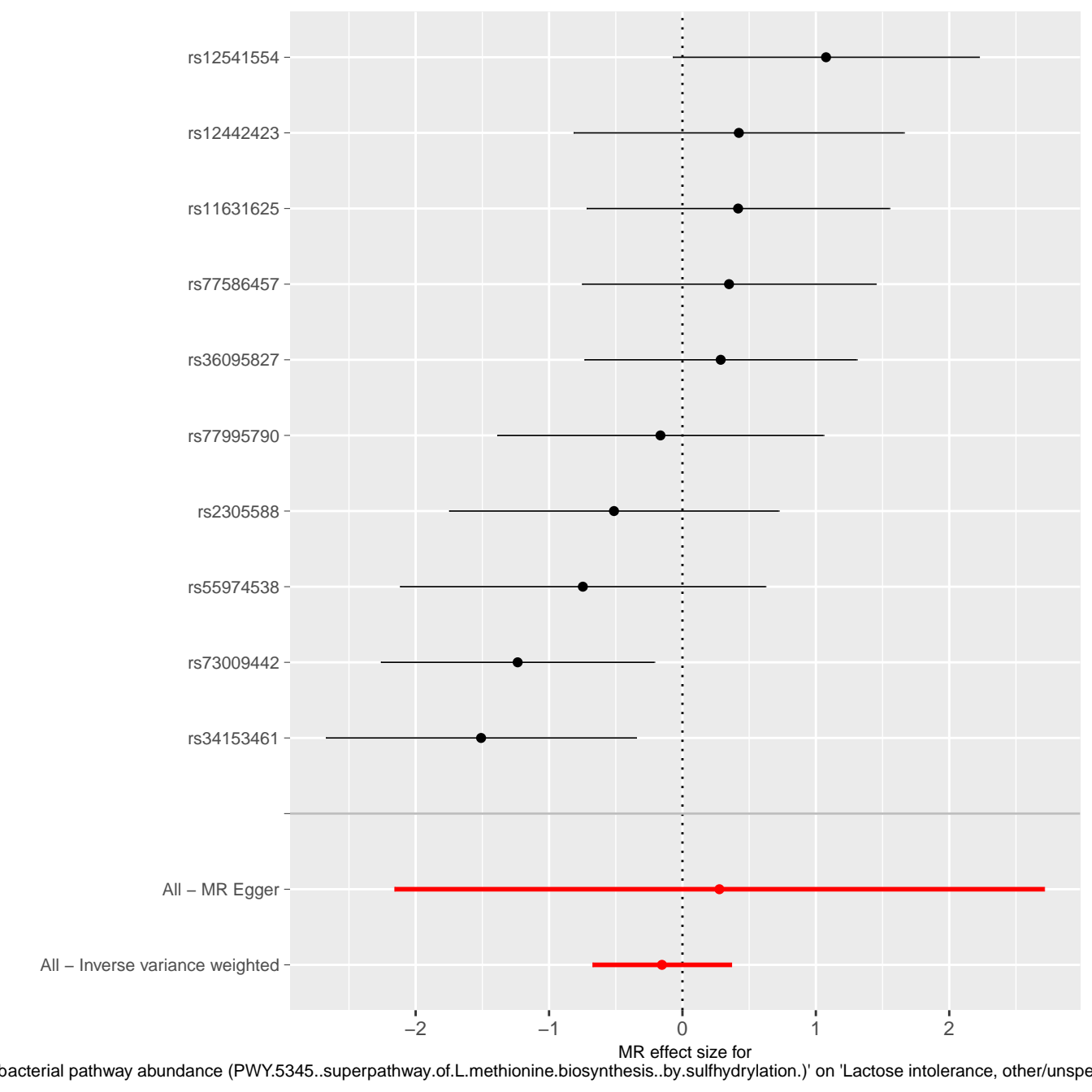

Supplement: Supplementary file 1 [file Data_Sheet_1.zip › supplementary materials/Forward/forest plot/ebi-a-GCST90027548.finngen_R12_E4_LACTONAS.pdf]

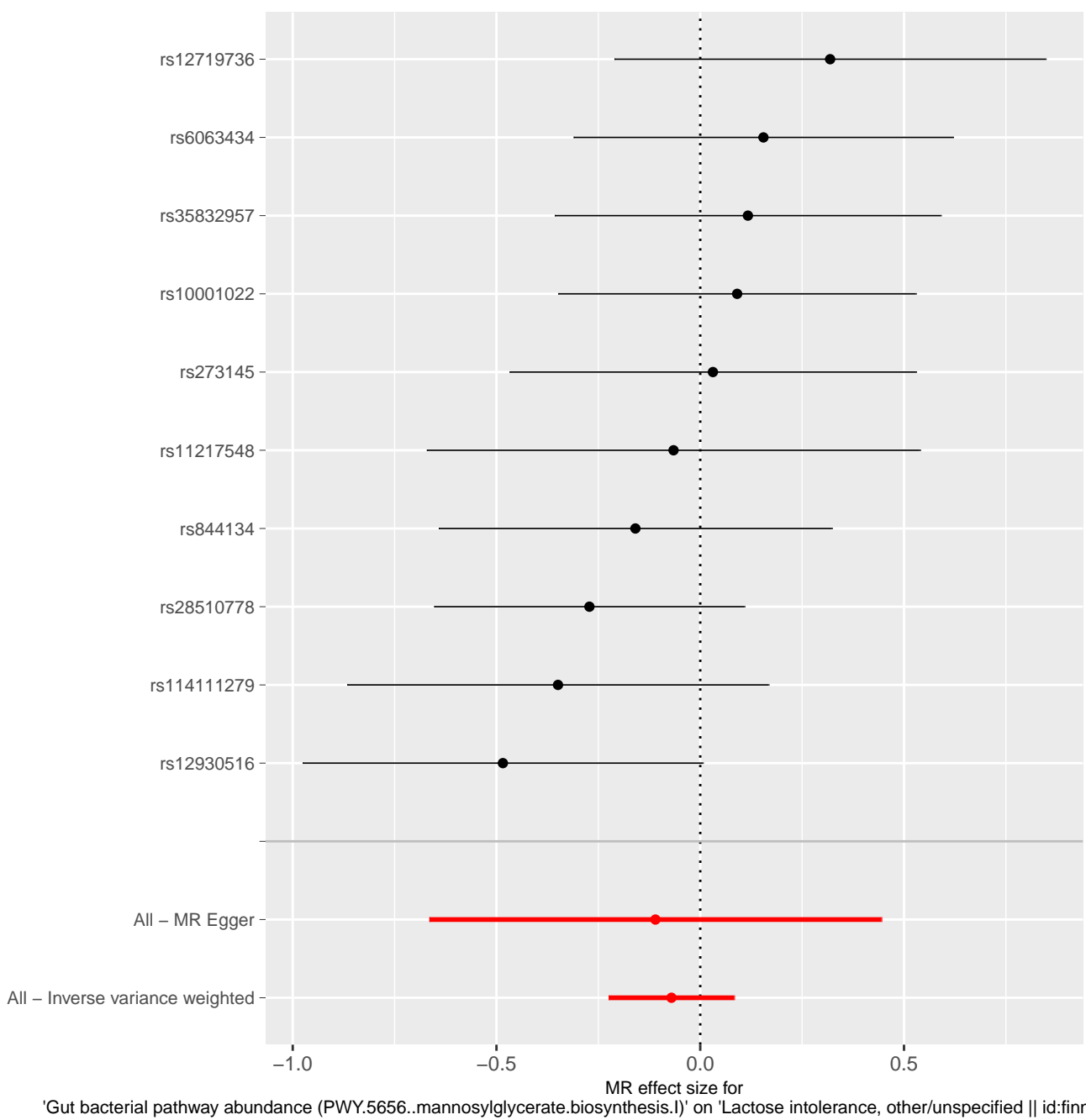

Supplement: Supplementary file 1 [file Data_Sheet_1.zip › supplementary materials/Forward/forest plot/ebi-a-GCST90027551.finngen_R12_E4_LACTONAS.pdf]

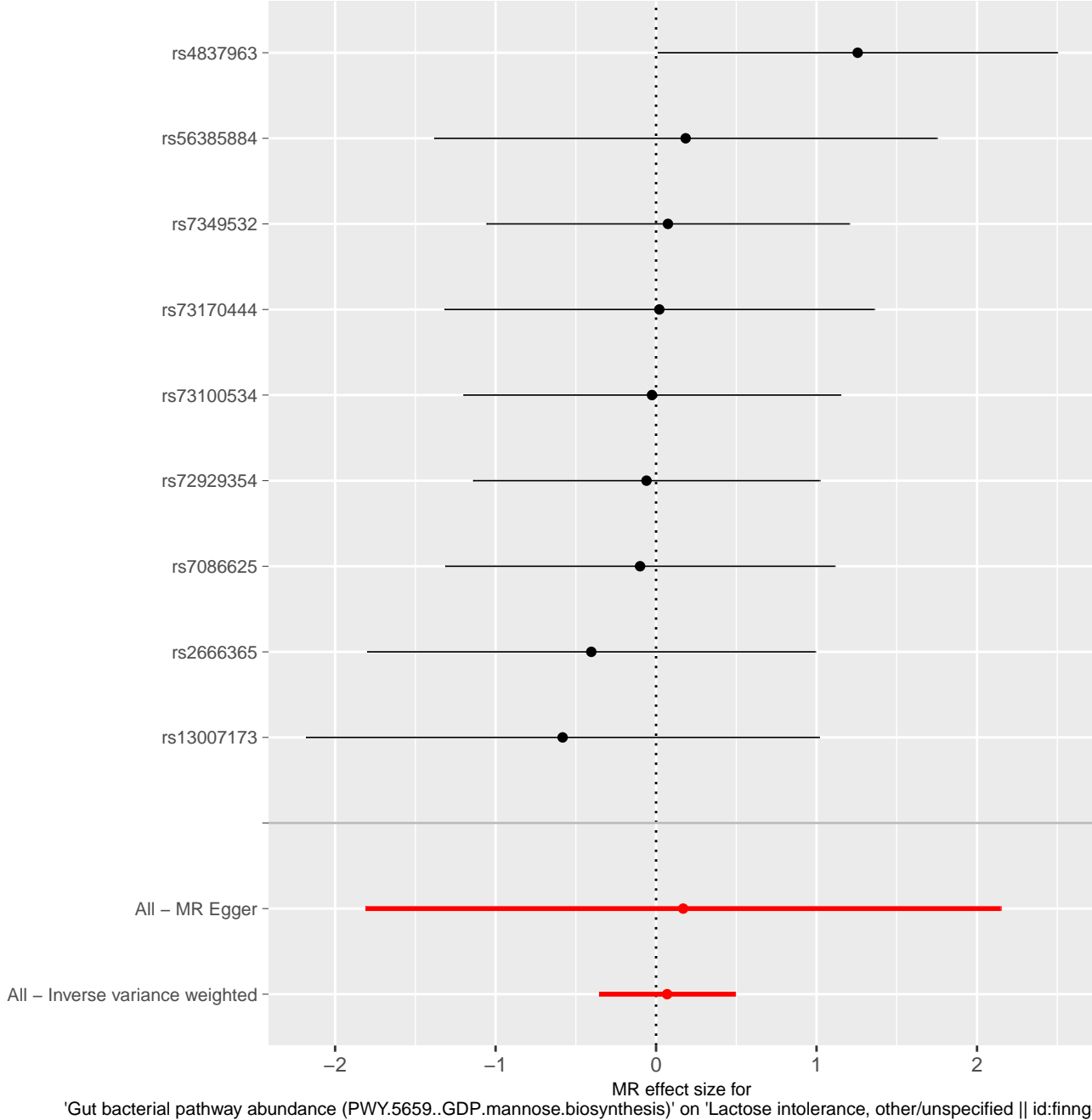

Supplement: Supplementary file 1 [file Data_Sheet_1.zip › supplementary materials/Forward/forest plot/ebi-a-GCST90027552.finngen_R12_E4_LACTONAS.pdf]

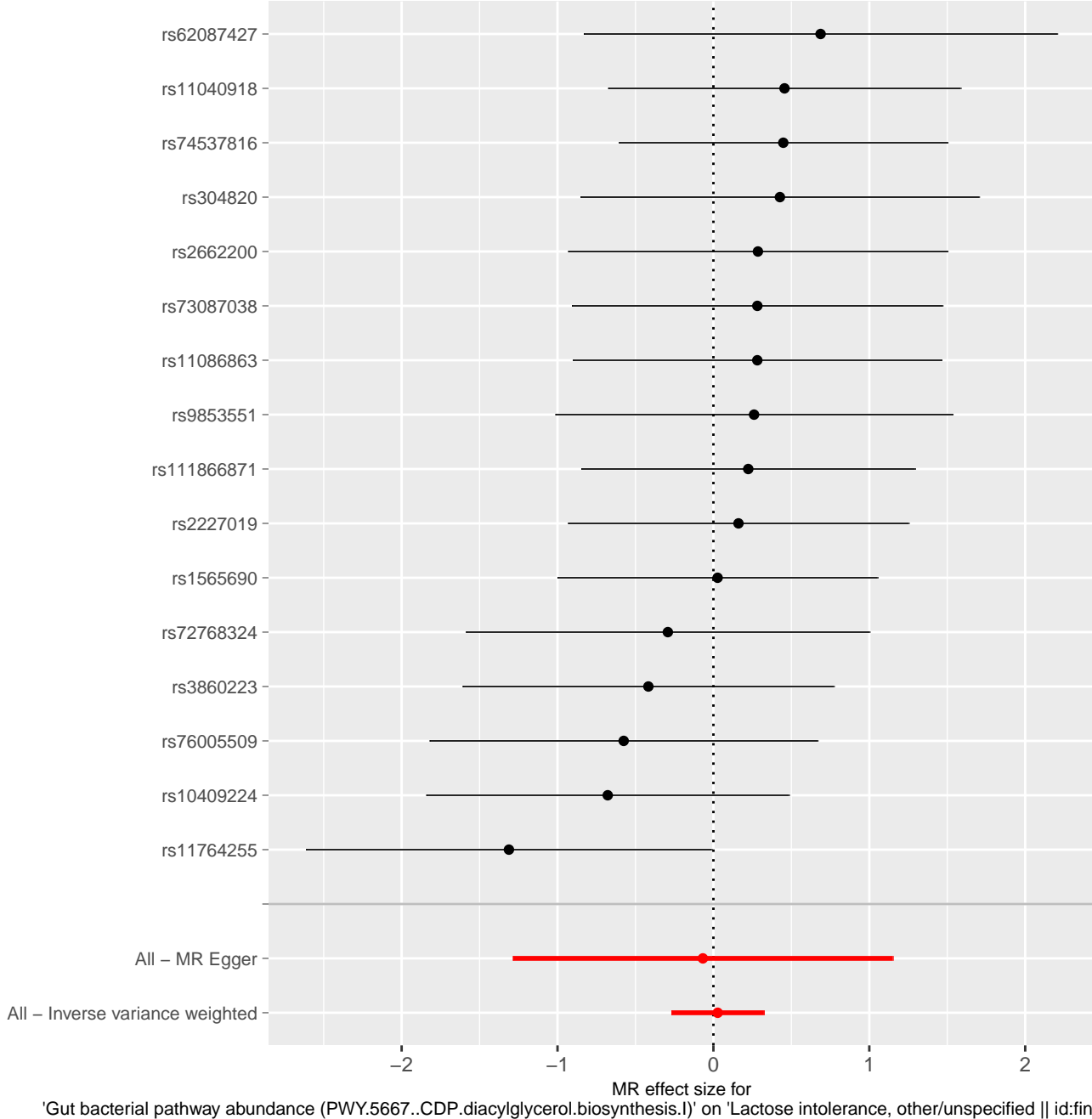

Supplement: Supplementary file 1 [file Data_Sheet_1.zip › supplementary materials/Forward/forest plot/ebi-a-GCST90027553.finngen_R12_E4_LACTONAS.pdf]

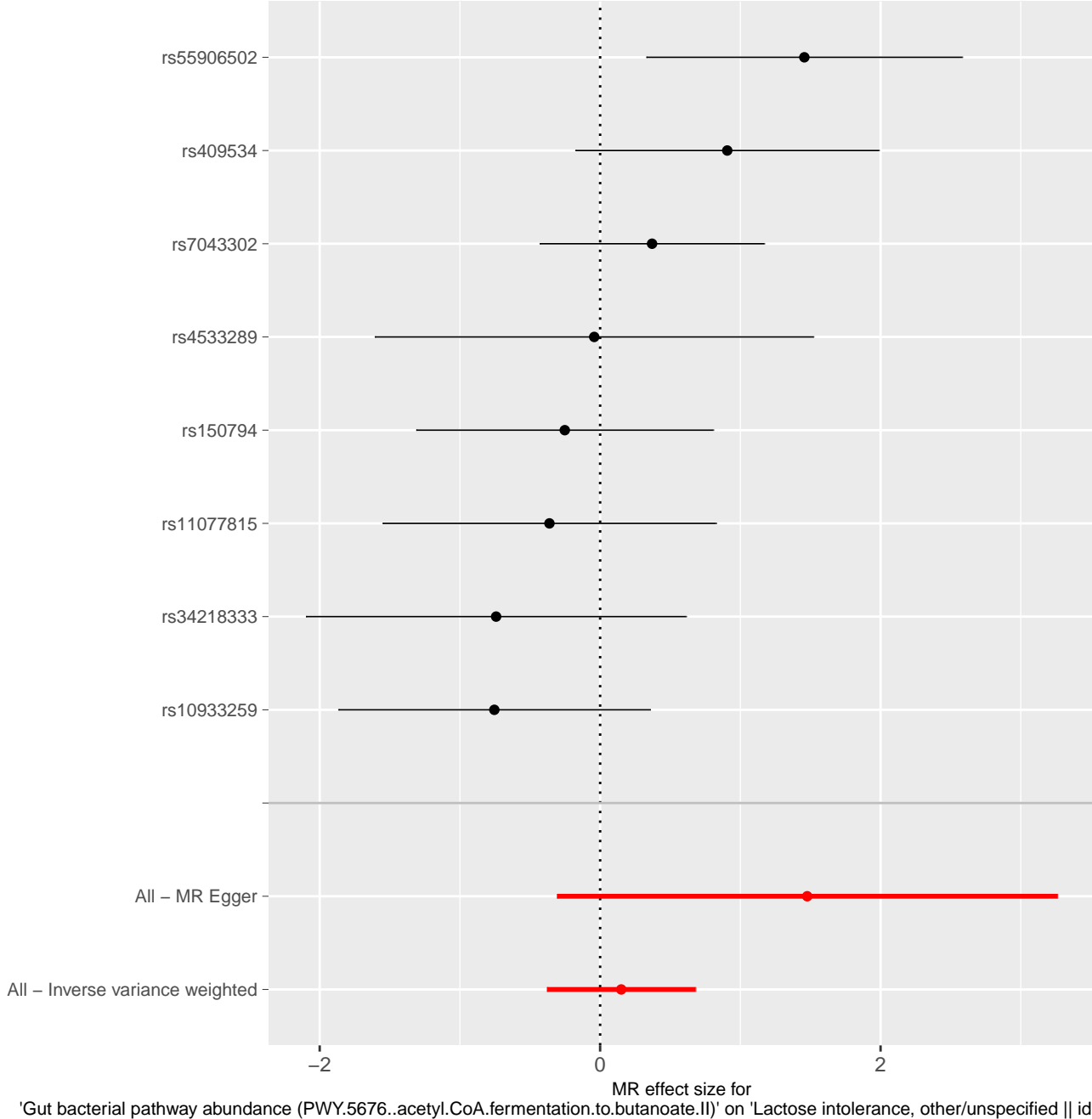

Supplement: Supplementary file 1 [file Data_Sheet_1.zip › supplementary materials/Forward/forest plot/ebi-a-GCST90027554.finngen_R12_E4_LACTONAS.pdf]

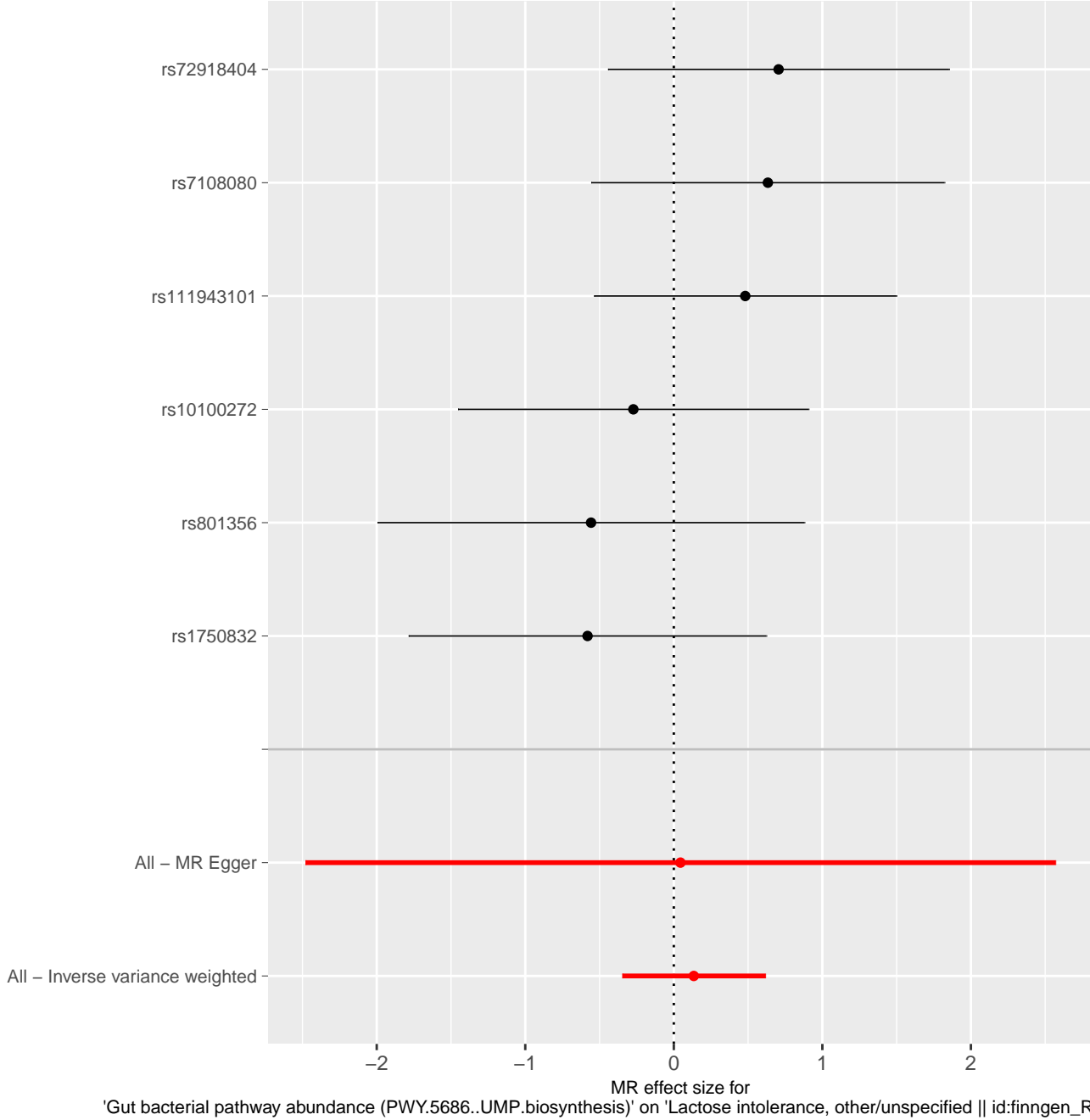

Supplement: Supplementary file 1 [file Data_Sheet_1.zip › supplementary materials/Forward/forest plot/ebi-a-GCST90027555.finngen_R12_E4_LACTONAS.pdf]

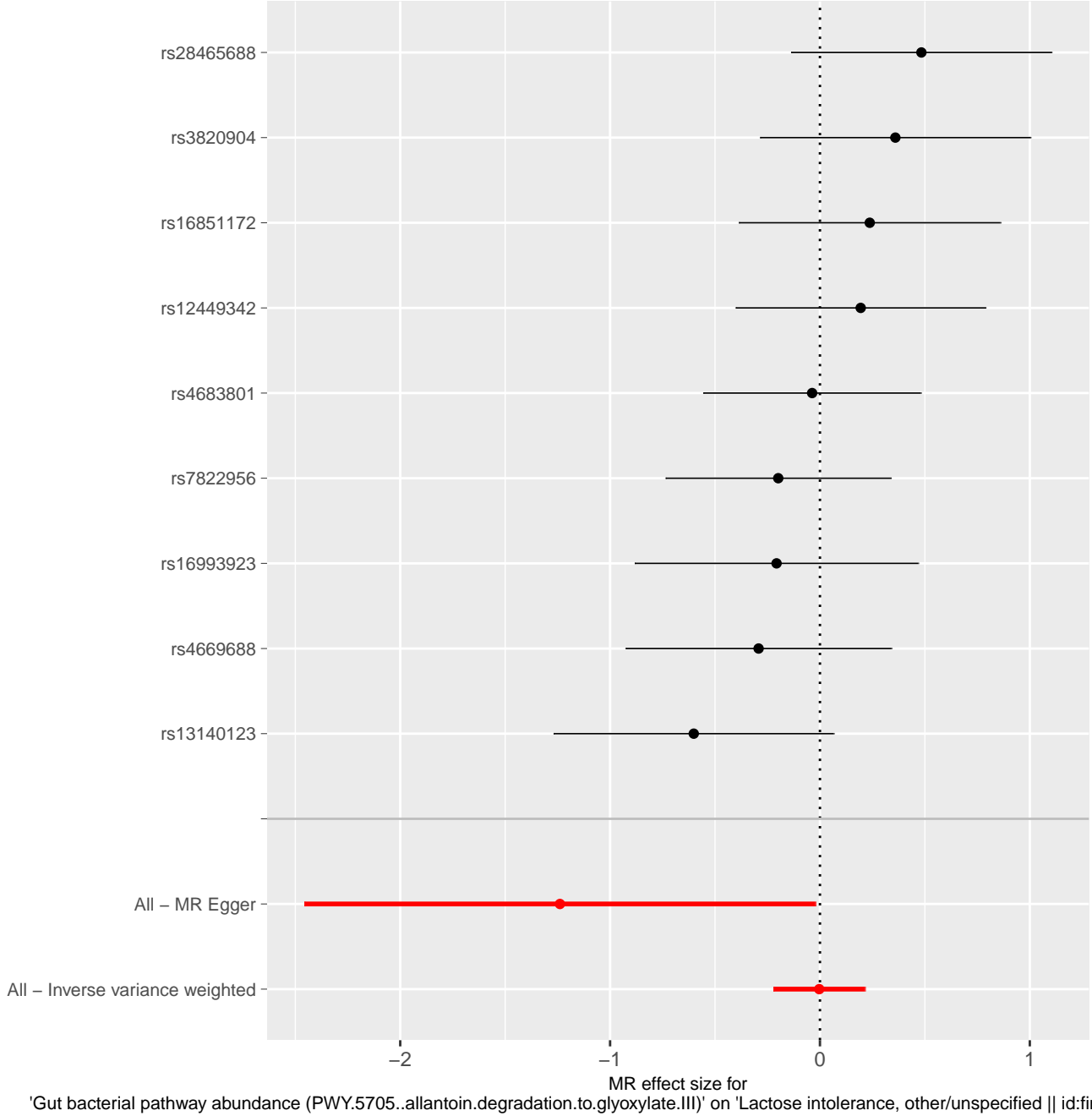

Supplement: Supplementary file 1 [file Data_Sheet_1.zip › supplementary materials/Forward/forest plot/ebi-a-GCST90027557.finngen_R12_E4_LACTONAS.pdf]

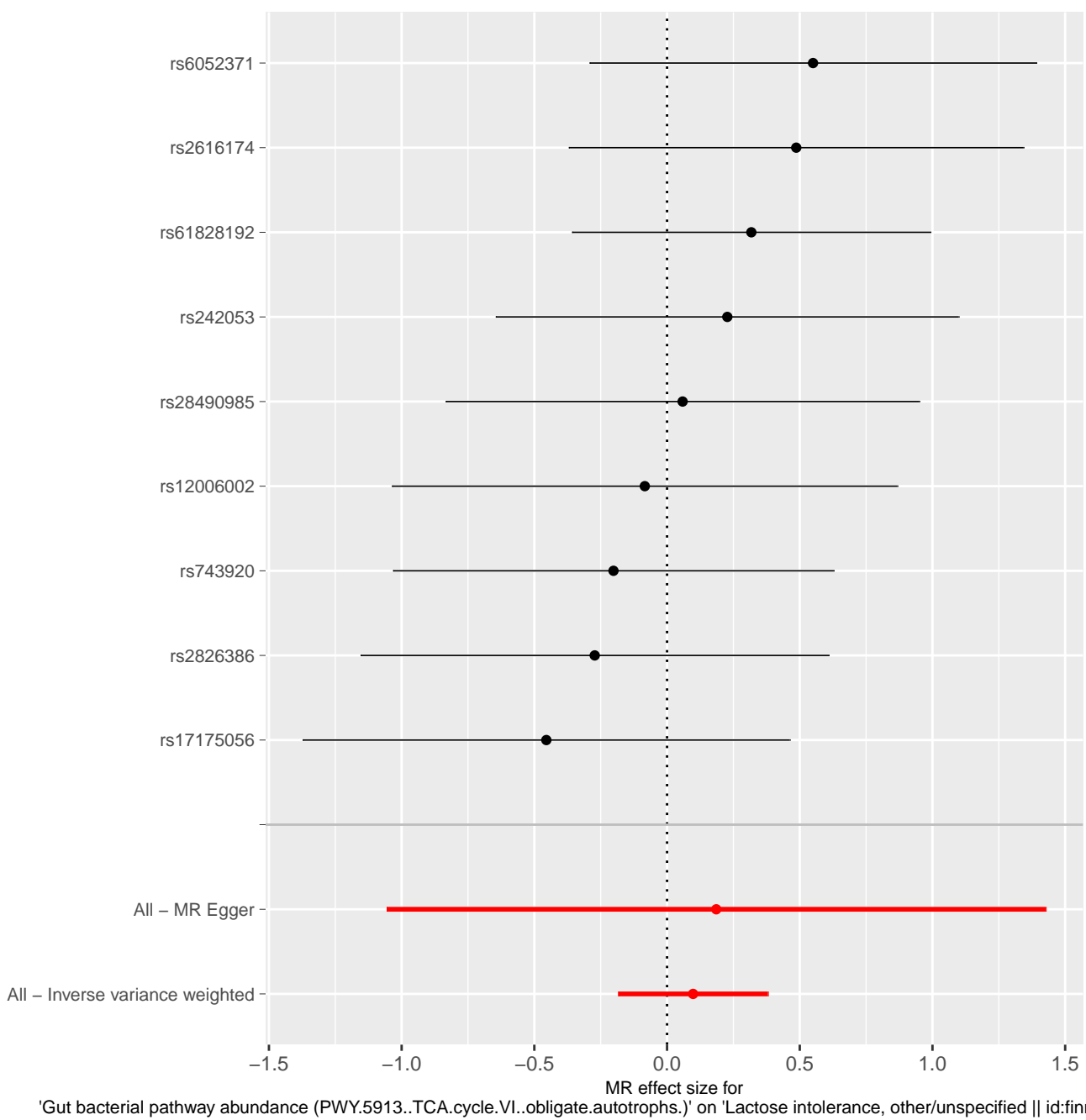

Supplement: Supplementary file 1 [file Data_Sheet_1.zip › supplementary materials/Forward/forest plot/ebi-a-GCST90027561.finngen_R12_E4_LACTONAS.pdf]

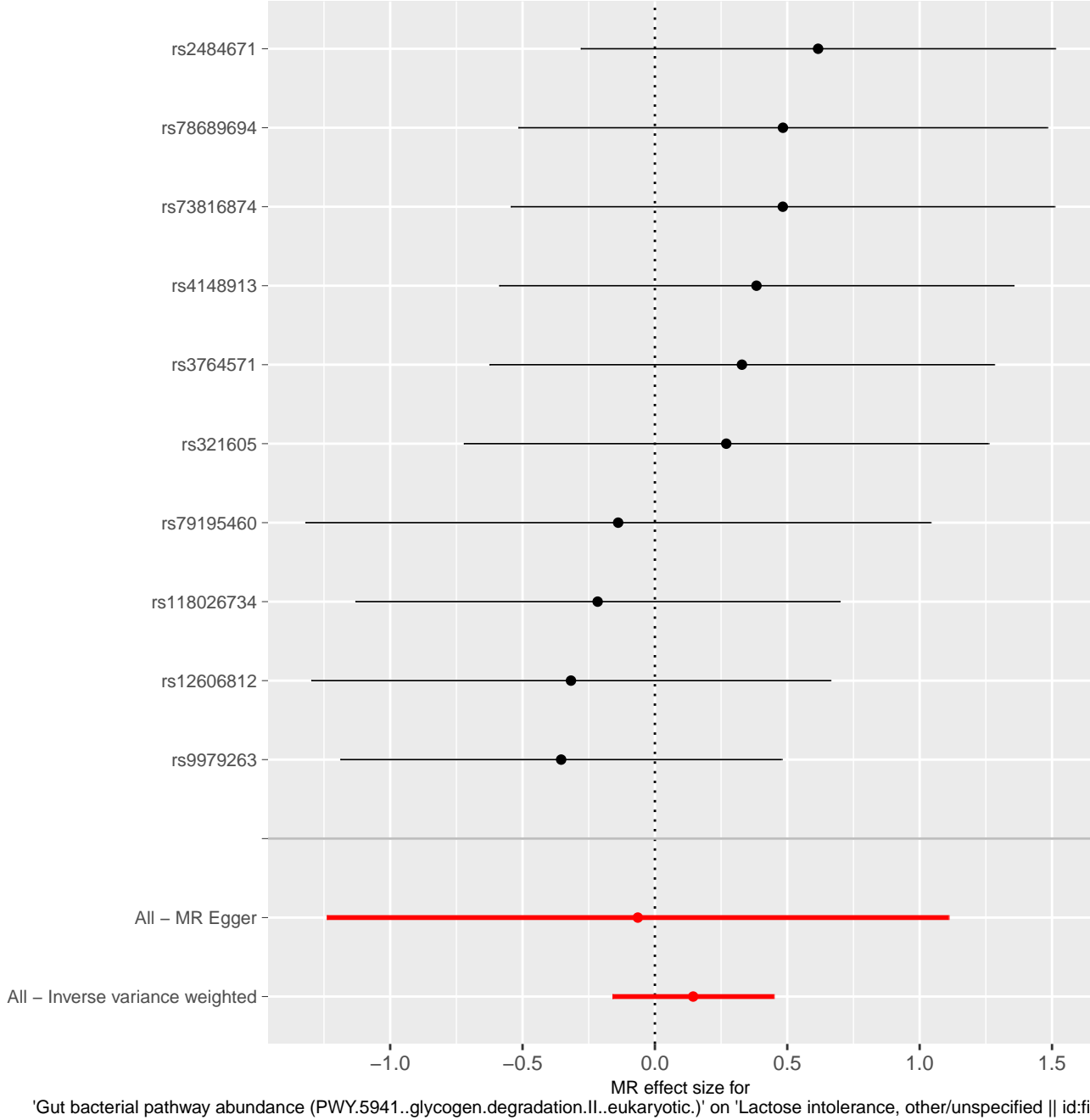

Supplement: Supplementary file 1 [file Data_Sheet_1.zip › supplementary materials/Forward/forest plot/ebi-a-GCST90027564.finngen_R12_E4_LACTONAS.pdf]

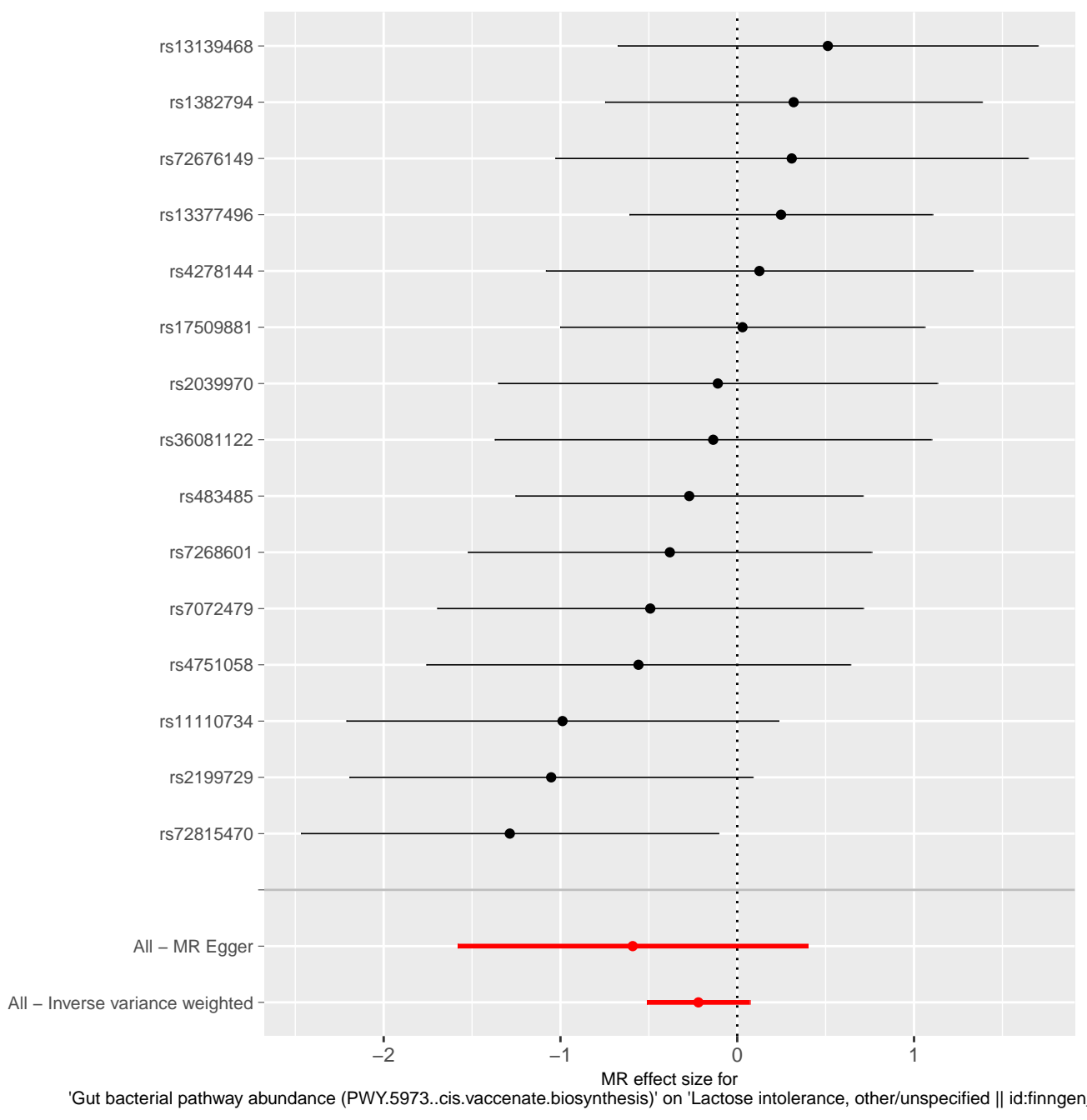

Supplement: Supplementary file 1 [file Data_Sheet_1.zip › supplementary materials/Forward/forest plot/ebi-a-GCST90027566.finngen_R12_E4_LACTONAS.pdf]

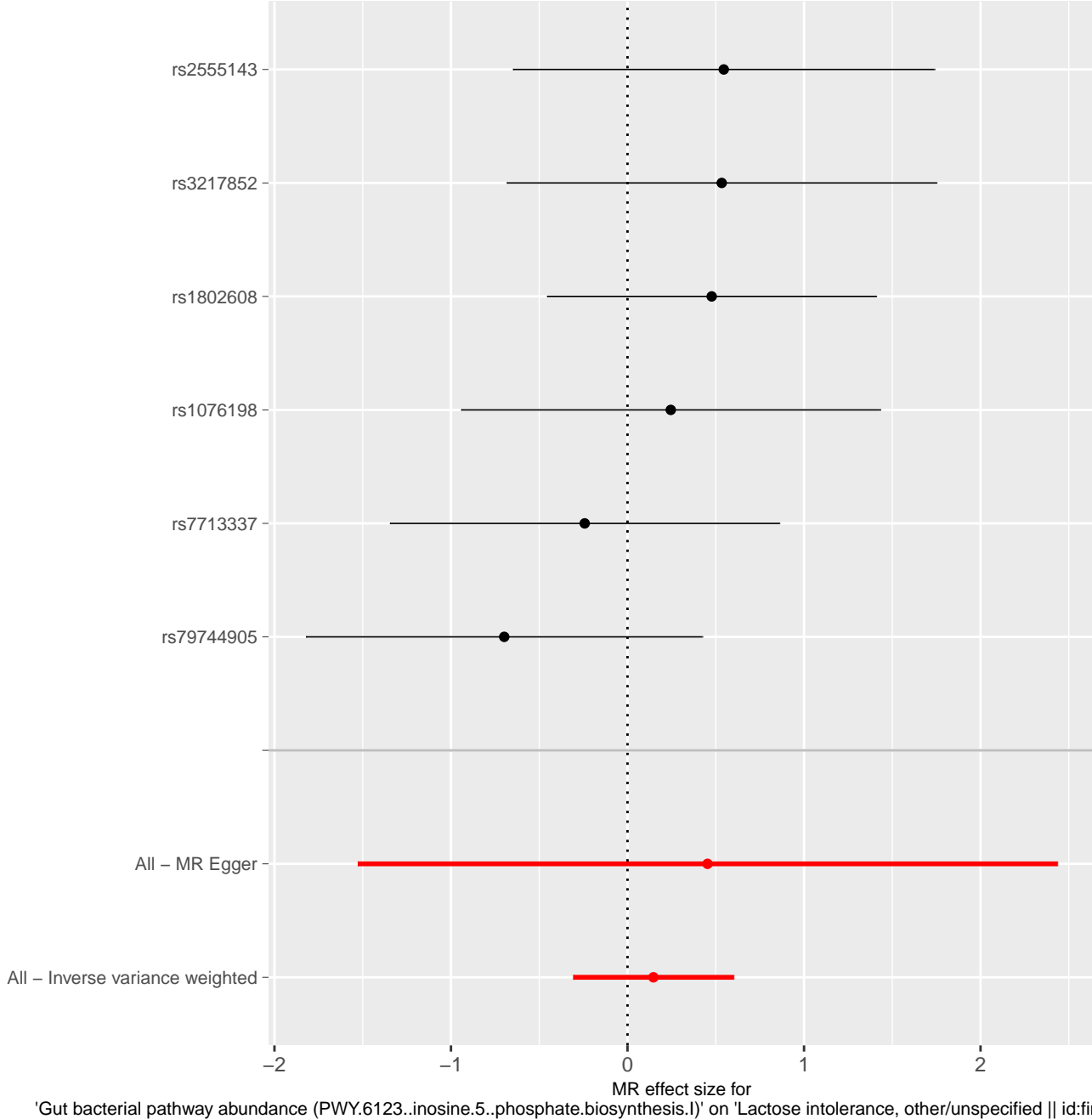

Supplement: Supplementary file 1 [file Data_Sheet_1.zip › supplementary materials/Forward/forest plot/ebi-a-GCST90027569.finngen_R12_E4_LACTONAS.pdf]

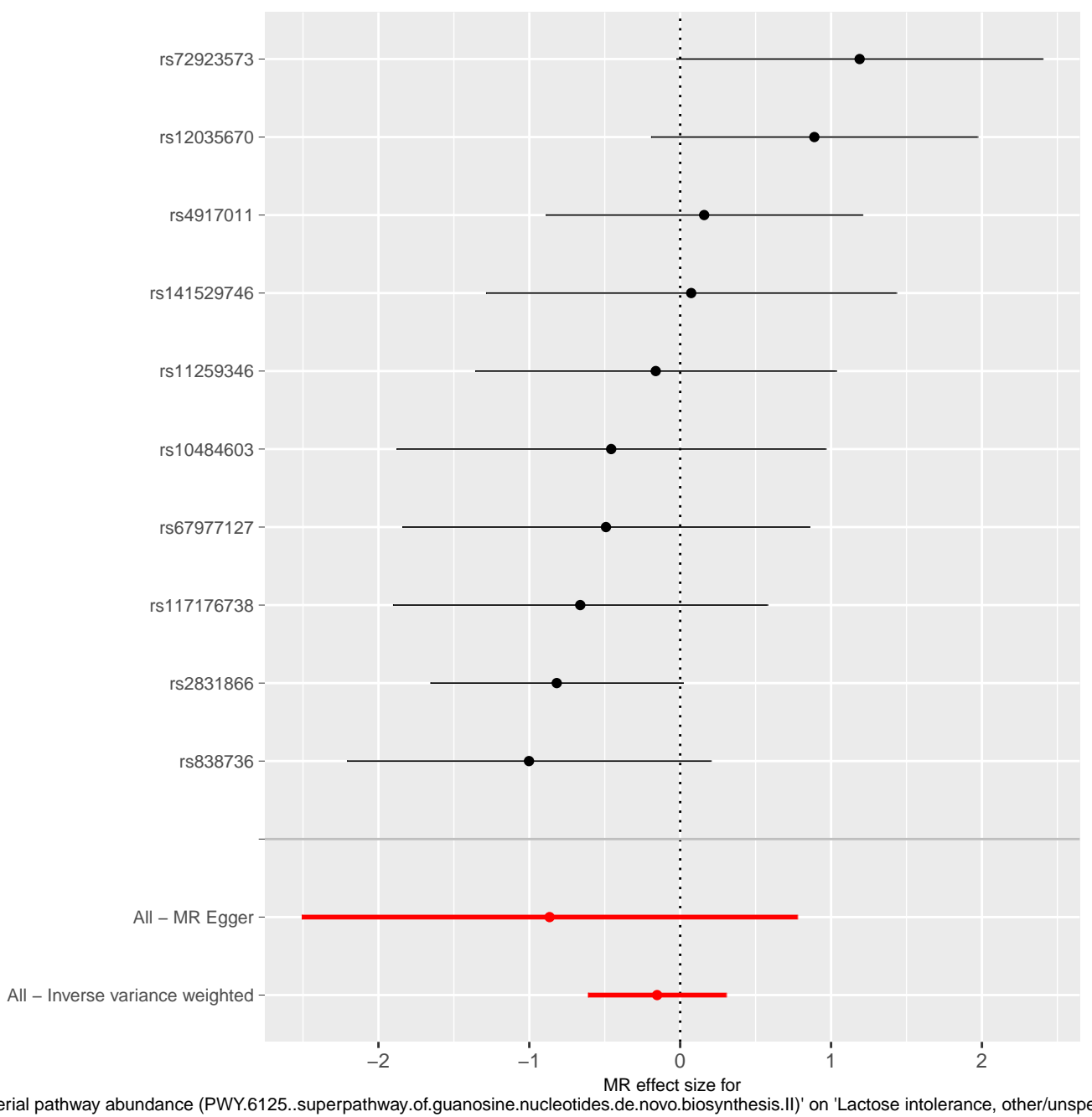

Supplement: Supplementary file 1 [file Data_Sheet_1.zip › supplementary materials/Forward/forest plot/ebi-a-GCST90027570.finngen_R12_E4_LACTONAS.pdf]

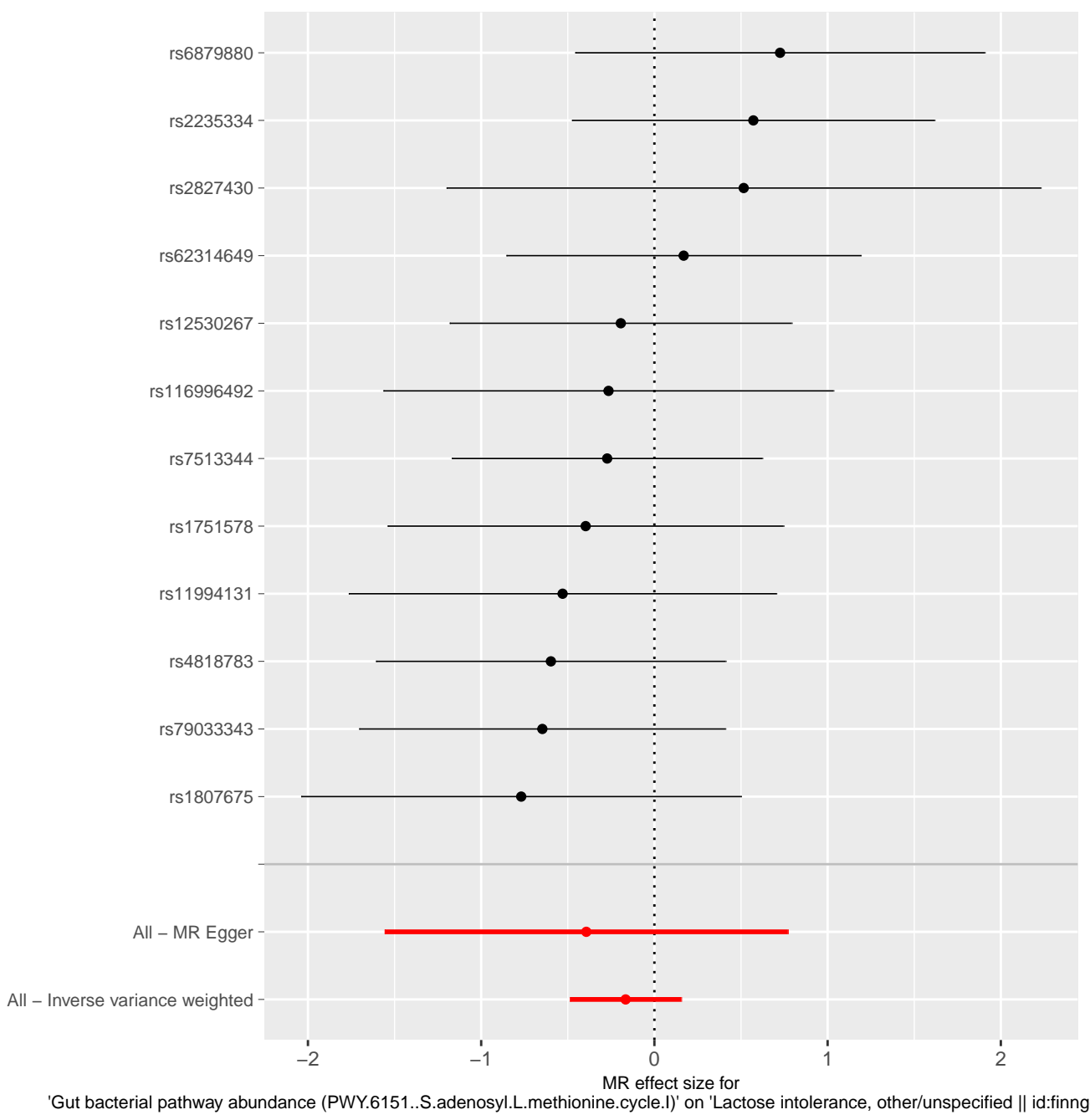

Supplement: Supplementary file 1 [file Data_Sheet_1.zip › supplementary materials/Forward/forest plot/ebi-a-GCST90027572.finngen_R12_E4_LACTONAS.pdf]

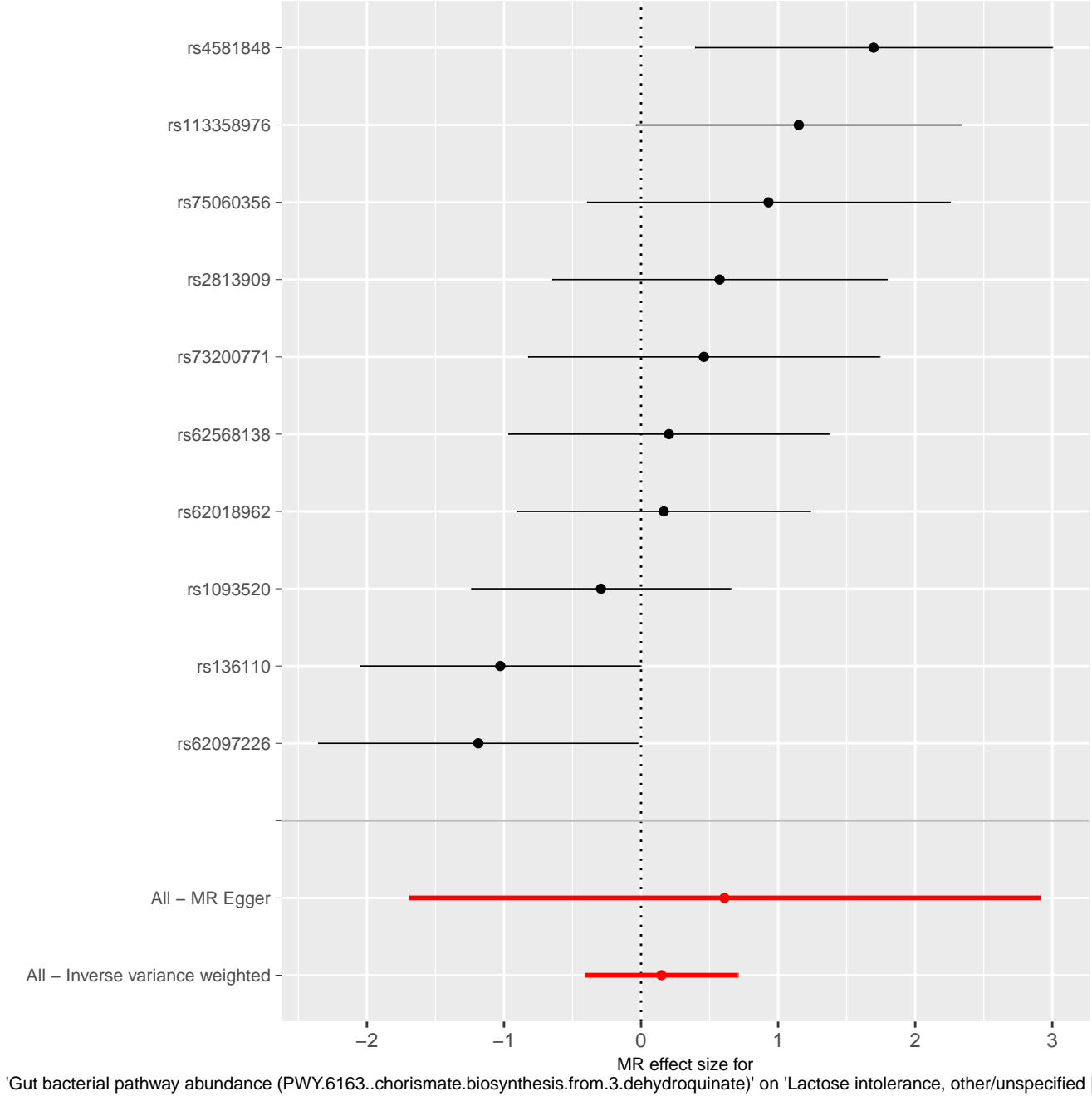

Supplement: Supplementary file 1 [file Data_Sheet_1.zip › supplementary materials/Forward/forest plot/ebi-a-GCST90027573.finngen_R12_E4_LACTONAS.pdf]

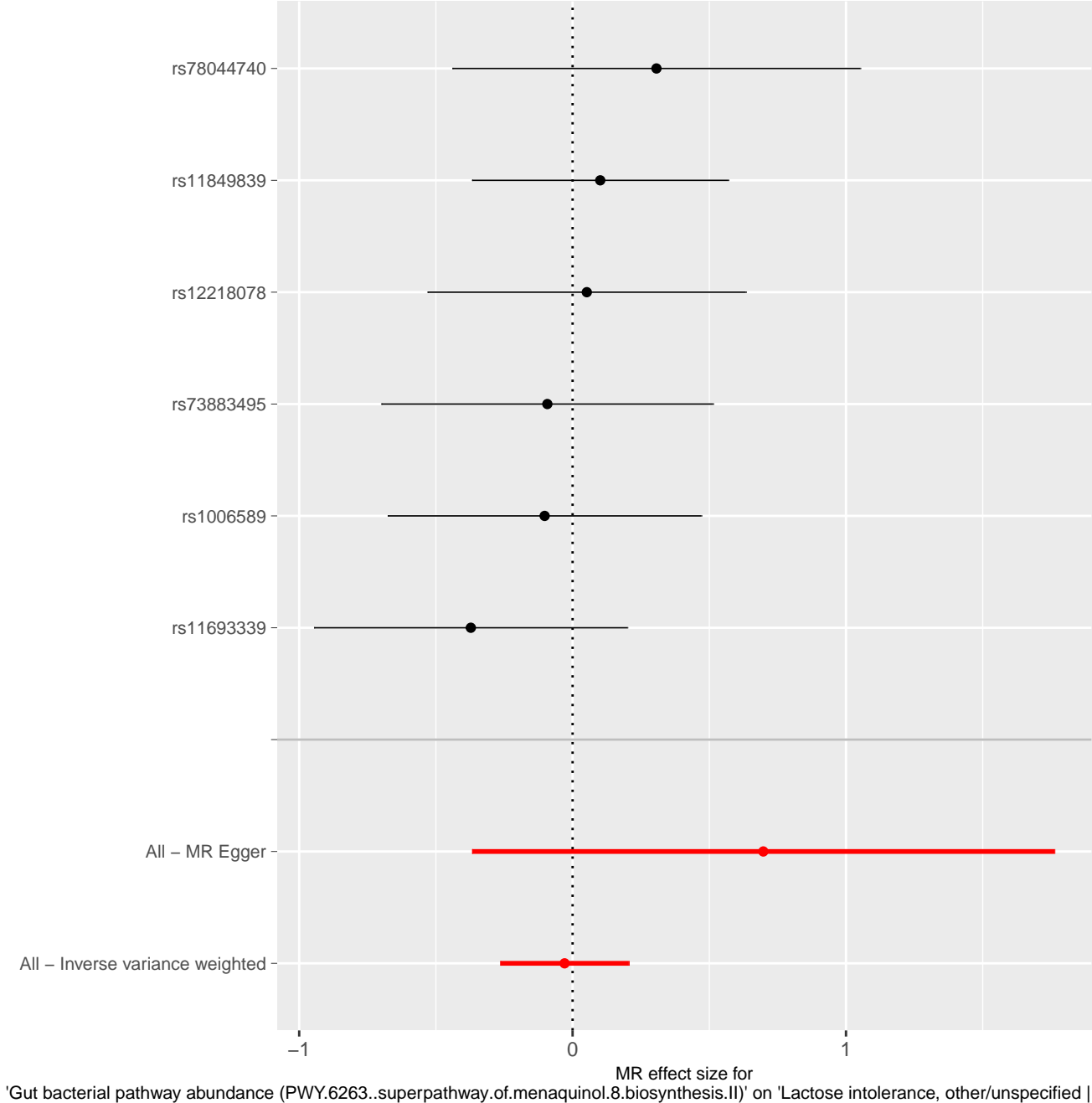

Supplement: Supplementary file 1 [file Data_Sheet_1.zip › supplementary materials/Forward/forest plot/ebi-a-GCST90027575.finngen_R12_E4_LACTONAS.pdf]

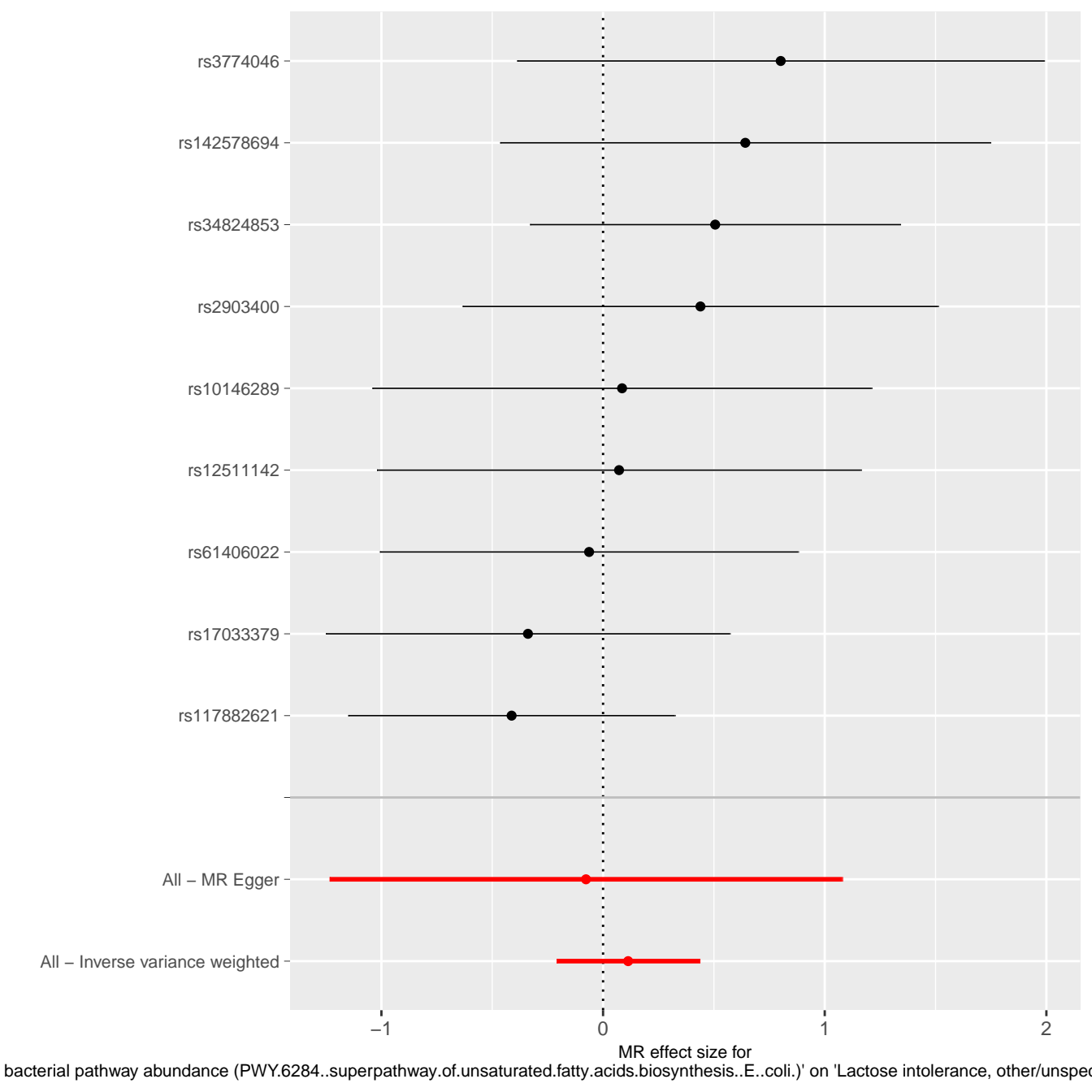

Supplement: Supplementary file 1 [file Data_Sheet_1.zip › supplementary materials/Forward/forest plot/ebi-a-GCST90027576.finngen_R12_E4_LACTONAS.pdf]

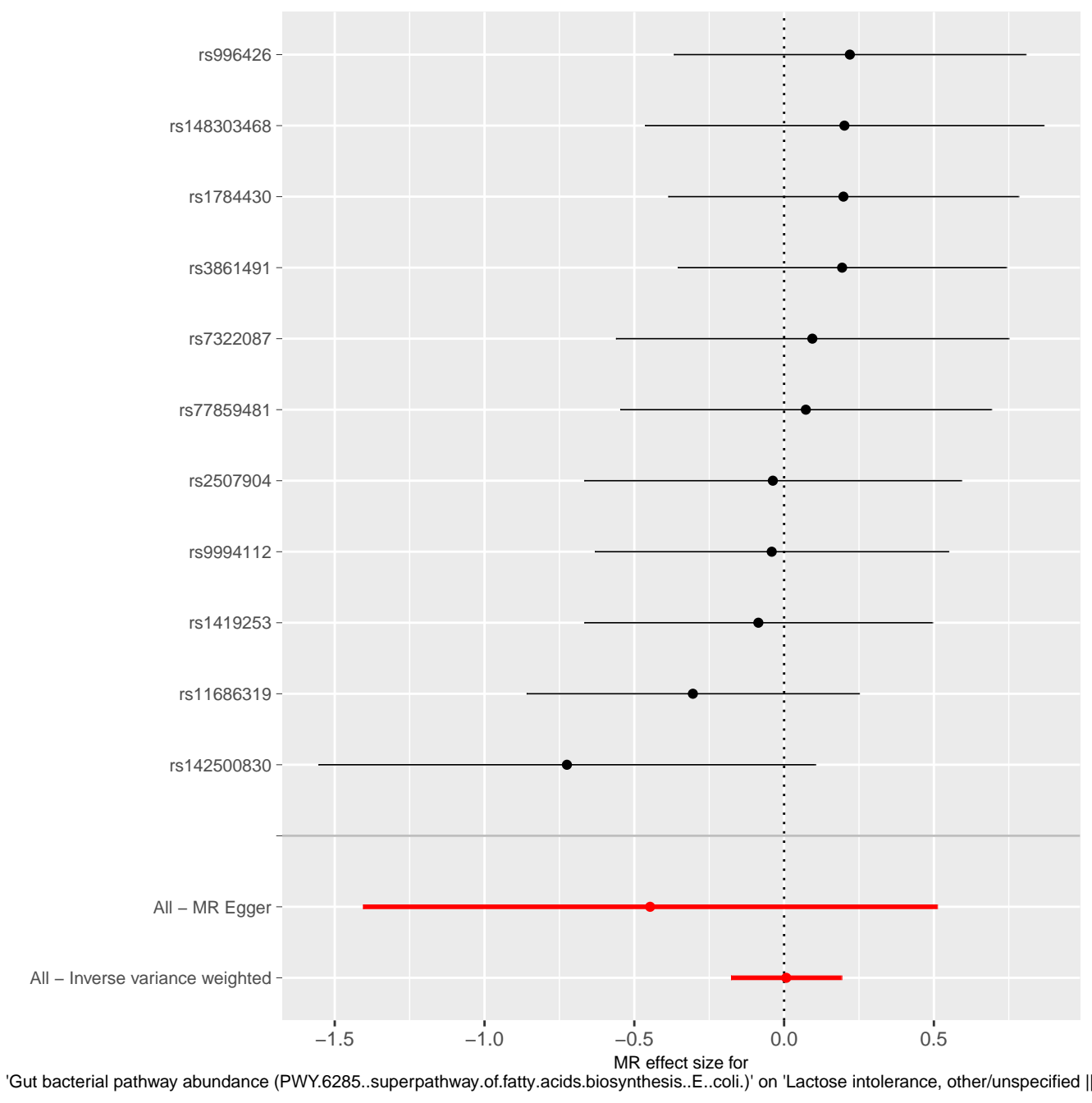

Supplement: Supplementary file 1 [file Data_Sheet_1.zip › supplementary materials/Forward/forest plot/ebi-a-GCST90027577.finngen_R12_E4_LACTONAS.pdf]

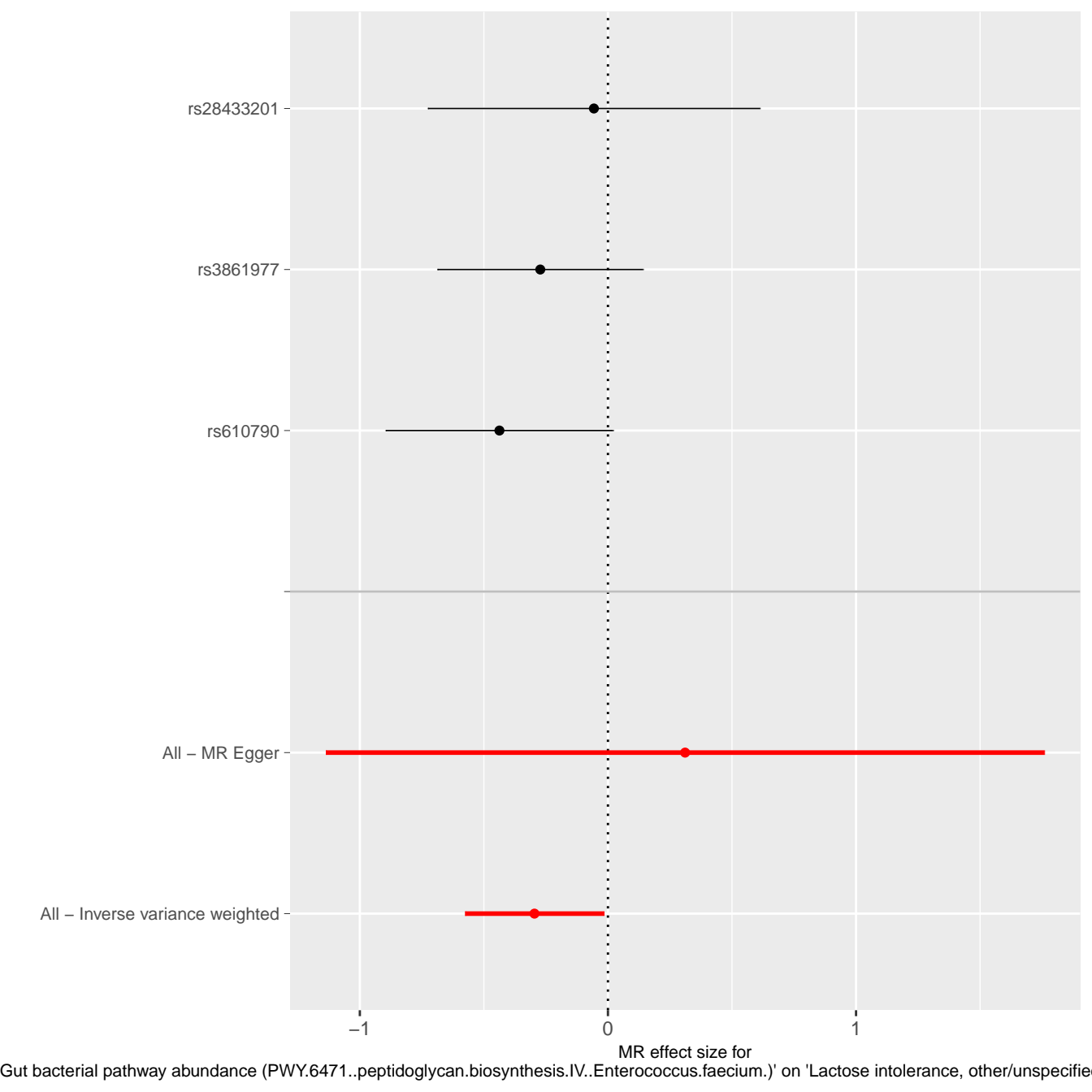

Supplement: Supplementary file 1 [file Data_Sheet_1.zip › supplementary materials/Forward/forest plot/ebi-a-GCST90027580.finngen_R12_E4_LACTONAS.pdf]

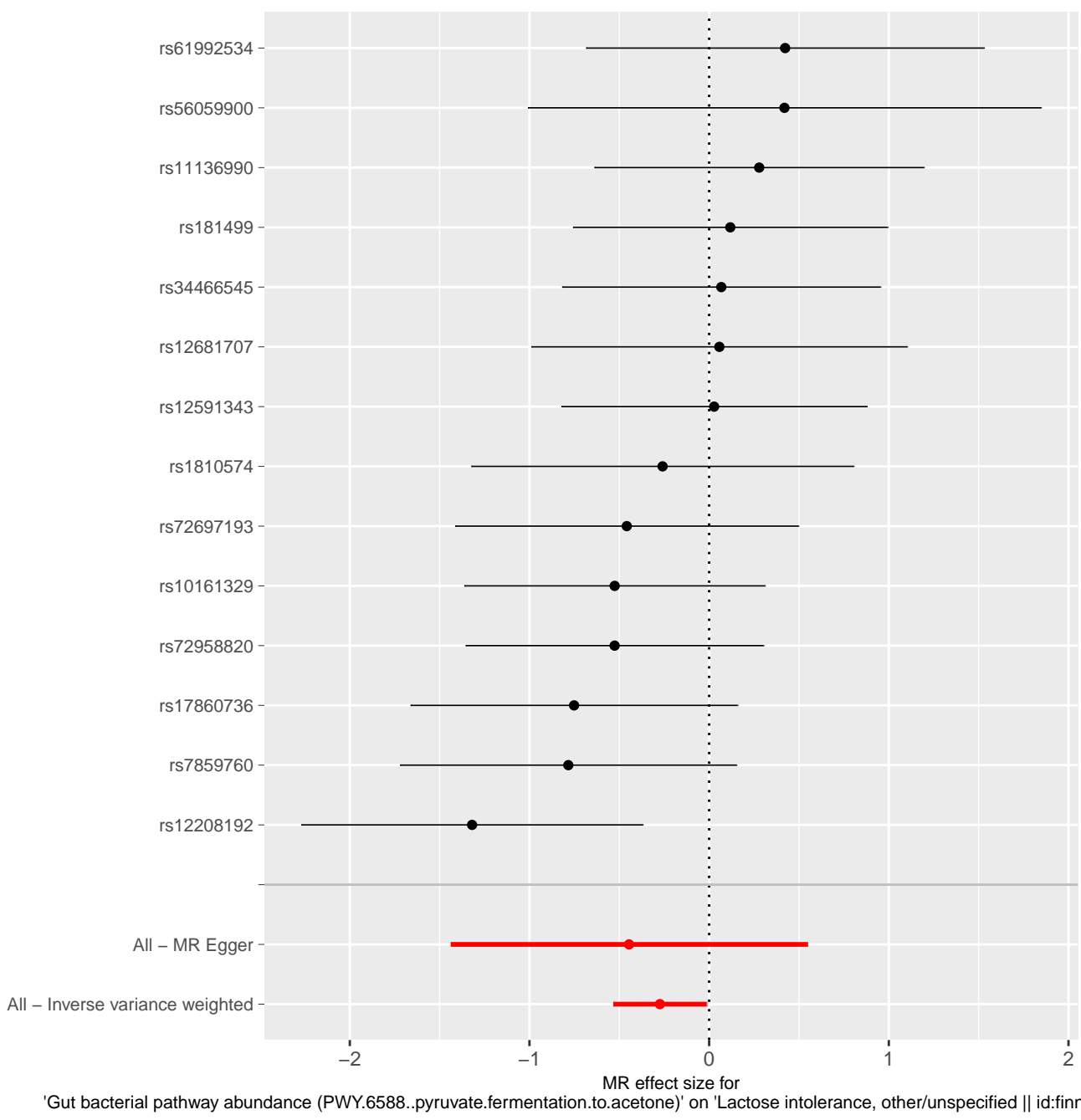

Supplement: Supplementary file 1 [file Data_Sheet_1.zip › supplementary materials/Forward/forest plot/ebi-a-GCST90027584.finngen_R12_E4_LACTONAS.pdf]

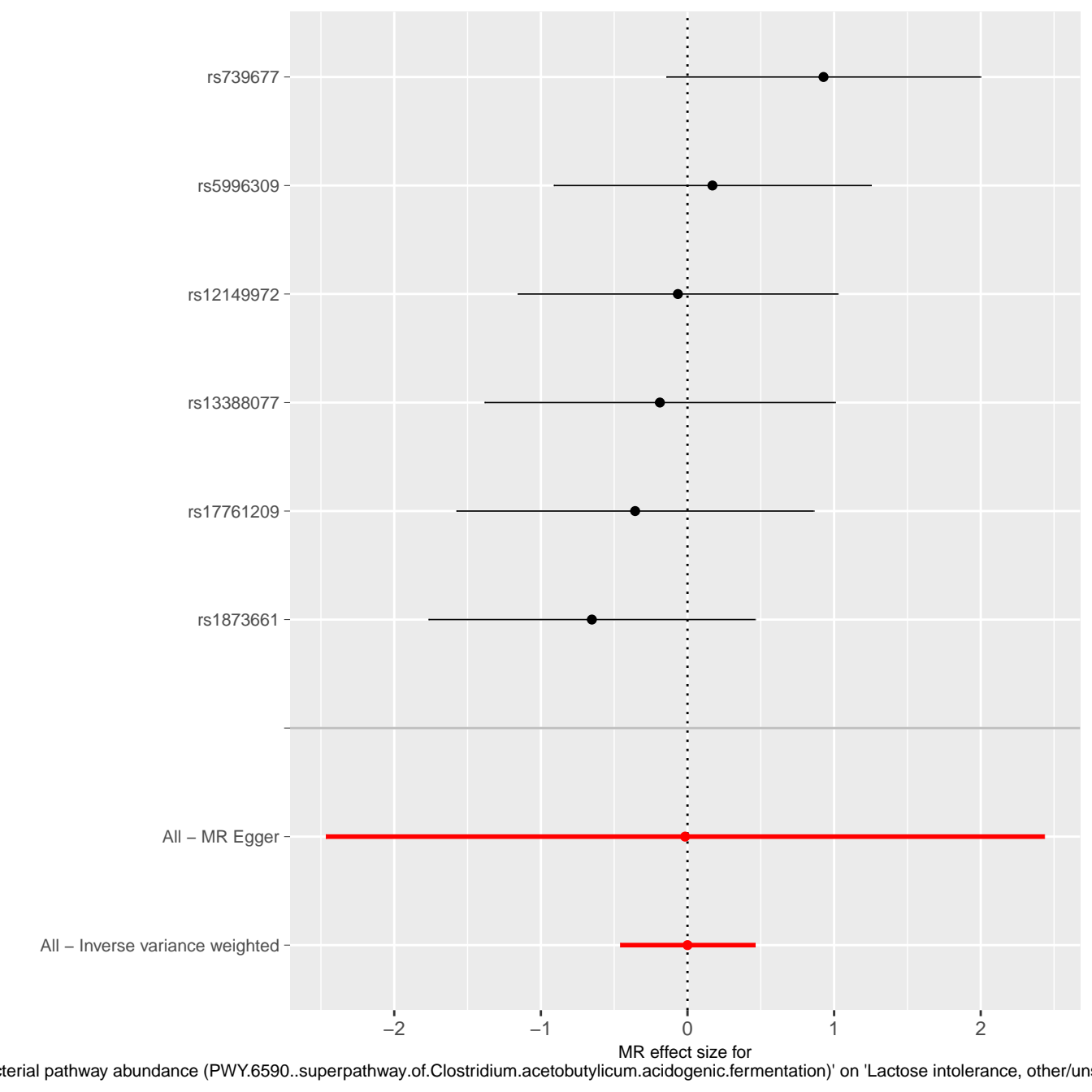

Supplement: Supplementary file 1 [file Data_Sheet_1.zip › supplementary materials/Forward/forest plot/ebi-a-GCST90027585.finngen_R12_E4_LACTONAS.pdf]

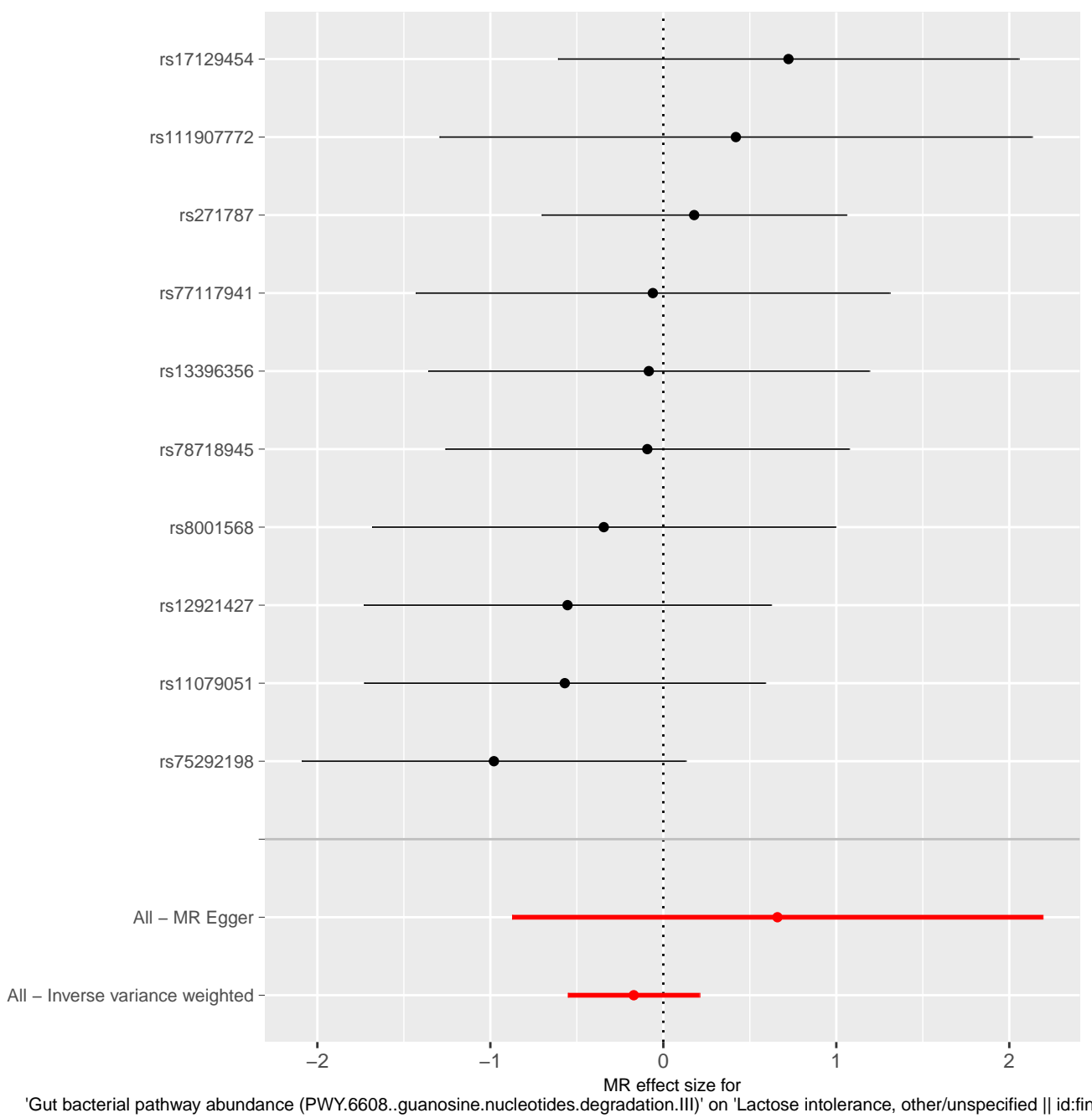

Supplement: Supplementary file 1 [file Data_Sheet_1.zip › supplementary materials/Forward/forest plot/ebi-a-GCST90027586.finngen_R12_E4_LACTONAS.pdf]

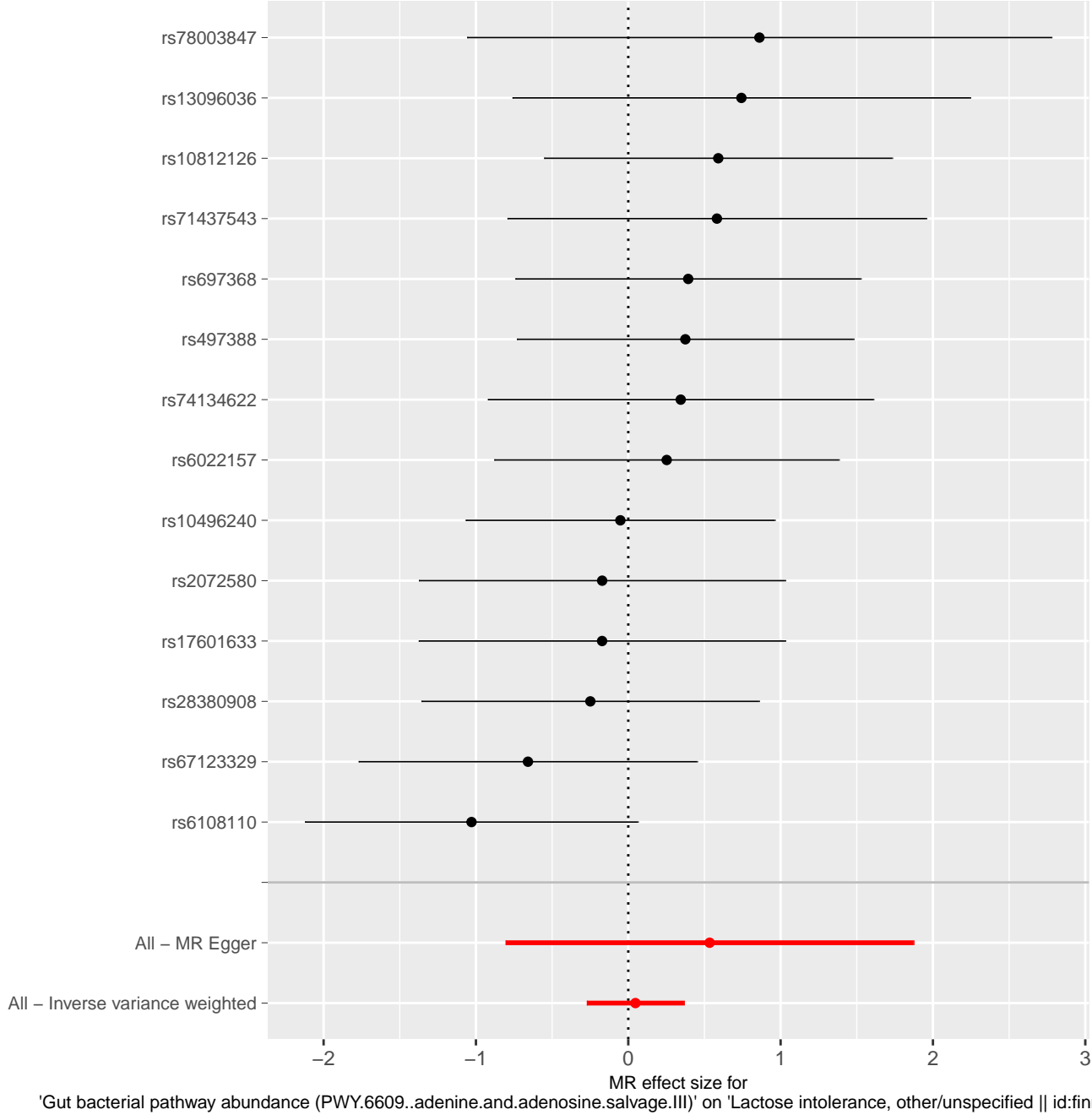

Supplement: Supplementary file 1 [file Data_Sheet_1.zip › supplementary materials/Forward/forest plot/ebi-a-GCST90027587.finngen_R12_E4_LACTONAS.pdf]
