# Supplementary material for: Causal interplay between lactose intolerance and gut microbiota: a combined bidirectional Mendelian randomization and in vivo validation study
Source: Front Nutr. 2026 Jun 1;13:1803337. doi: 10.3389/fnut.2026.1803337 (PMC13265576; doi:10.3389/fnut.2026.1803337)
Supplement: Supplementary file 1 [file ebi-a-GCST90027455.finngen_R12_E4_LACTONAS.pdf › supplementary materials/Forward/forest plot/ebi-a-GCST90027455.finngen_R12_E4_LACTONAS.pdf]

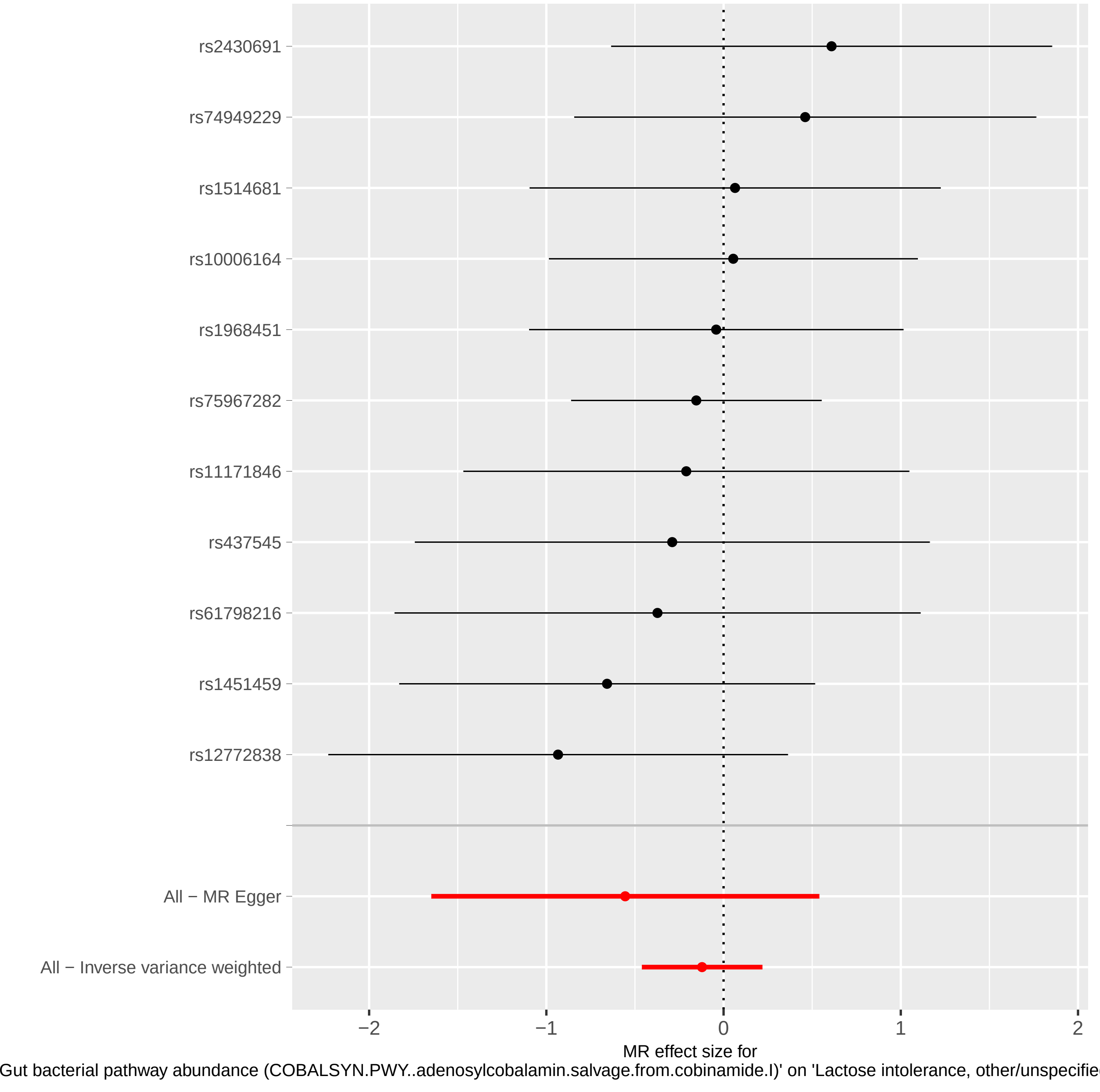

rs2430691
rs74949229
rs1514681
rs10006164
rs1968451
rs75967282
rs11171846
rs437545
rs61798216
rs1451459
rs12772838
All − MR Egger
All − Inverse variance weighted
−2
−1
0
1
2
MR effect size for
Gut bacterial pathway abundance (COBALSYN.PWY..adenosylcobalamin.salvage.from.cobinamide.I)' on 'Lactose intolerance, other/unspecifie
